# Taxonomic and phylogenetic diversity of vascular plants at Ma’anling volcano urban park in tropical Haikou, China: Reponses to soil properties

**DOI:** 10.1371/journal.pone.0198517

**Published:** 2018-06-18

**Authors:** Xia-Lan Cheng, Lang-Xing Yuan, Mir Mohammad Nizamani, Zhi-Xin Zhu, Cynthia Ross Friedman, Hua-Feng Wang

**Affiliations:** 1 Hainan Key Laboratory for Sustainable Utilization of Tropical Bioresources, Institute of Tropical Agriculture and Forestry, Hainan University, Haikou, China; 2 Department of Botany, University of British Columbia, Vancouver, B.C., Canada; Shandong University, CHINA

## Abstract

Anthropogenic processes and socio-economic factors play important roles in shaping plant diversity in urban parks. To investigate how plant diversity of Ma’ anling urban volcano park in Hainan Province, China respond to these factors, we carried out a field investigation on the taxonomic and phylogenetic diversity of vascular plants and soil properties in this area. We found 284 species of vascular plants belonging to 88 families and 241 genera, which included 194 native species, 23 invasive species, 31 naturalized species, 40 cultivars, and 4 rare / endangered plant species. Tree composition and richness significantly varied between different vegetation formations (plantation, secondary forest, and abandoned land). Plant species richness and community composition were significantly affected by elevation (El), soil water content (WC), total soil nitrogen (TN) and soil organic matter (SOM). There were significant diversity differences between plantations and abandoned lands, but not between the plantations and secondary forests. The flora in the study site was tropical in nature, characterized by pantropic distributions. Compared to adjacent areas, floristic composition in the study site was most similar to that of Guangdong, followed by that of Vietnam. Our study revealed the diversity patterns of volcanic plants and provided the basis for future planning of plant conservation, such as preserving plant species, maintaining plant habitats, and coordinating plant management in this region.

## Introduction

Plant diversity is a key property of ecosystems. It includes taxonomic diversity, phylogenetic diversity and functional diversity[1]. Taxonomic diversity accounts for species composition and abundance[2,3]. Phylogenetic diversity (PD) predicts species similarity and is a useful metric for measuring the biodiversity of a group of species [3,4]. This measure is based on the evolutionary distance among the species in the subset [5]. It can reveal the ecological and evolutionary mechanisms underlying the assembly of a community[3]. At present, human activities and many negative environmental impacts threaten plant diversity [6]. Due to over-exploitation and poor management, about 15–47% of the natural forests in tropical Asia exist in highly degraded states[7]. China’s tropical areas suffer from plantation expansion and deforestation [8]. Forests on Hainan Island are an important component of Chinese tropical forests. By the 1990s, deforestation increased at Hainan Island due to plantation expansion[8], which had negative impacts on plant diversity. Plant composition and functional diversity in Haikou, the capital of Hainan, were also influenced by the frequency and the type of land use changes [9]. Besides plantation expansion, nature-based tourism also keeps increasing in Hainan. This type of tourism has a variety of negative environmental impacts, such as alteration to the vegetation structure, habitat fragmentation, changes in soil nutrients and hydrology as well as indirect impacts including those from the spread of weeds and pathogens [10–14]. Phylogenetic diversity and taxonomic diversity are necessary to support the management of natural park ecosystems for the purpose of maintaining or even improving plant diversity.

Plant diversity is critical to ecosystem processes and functioning [15]. Most of the studies that had investigated relationships between plant diversity and ecosystem functioning were focused on the aboveground part of the plants. However, as most plants also have a belowground system, a more holistic approach, taking soils into account, is necessary for us to fully understand ecosystem functioning [16]. Recently, vegetation and soil properties were studied together to reveal how plant diversity responds to soil properties. Studies in grasslands showed that soil properties significantly affect biodiversity [17]. The diversity in coniferous forests was determined by soil total nitrogen and pH [18]. Low soil nutrients and low soil water content prevailed in the coniferous zone [19], constraining most lowland rain forest species with acquisitive traits [18]. There are also studies showing that plant diversity was not strongly related to the variables of soil characteristics, and the positive relationship existed between bulk density and sun woody species [20]. Soil has clearly played a key role in speciation and species coexistence [21]. All of these studies have shown that soil properties are key factors influencing plant diversity. However, little is known about how plant diversity responds to volcanic soil properties. Most studies on volcanic area biodiversity at present generally focus on the diversity of soil bacteria and fungi in volcanic soils[22,23], seed dispersal and plant succession[18,19,24], vegetation structure and species richness on rock outcrops, and prevalent volcanic soils[25]. The response of vascular plant diversity to volcanic soils in tropical regions needs more study.

In this study, we investigated the composition of vegetation as well as the physical and chemical properties of soils in moist evergreen forests at Ma’ anling urban volcano park in Haikou to address the following objectives. Firstly, we quantified current patterns of species composition and richness. Secondly, we compared taxonomic and phylogenetic diversity of vascular plants between different habitats. Thirdly, we examined the relationship between composition of vegetation and soil properties. Our study provided important information about taxonomic and phylogenetic diversity of vascular plants in this park. This information would facilitate the management of the local flora.

## Methods

### Study site

**Fig 1:**
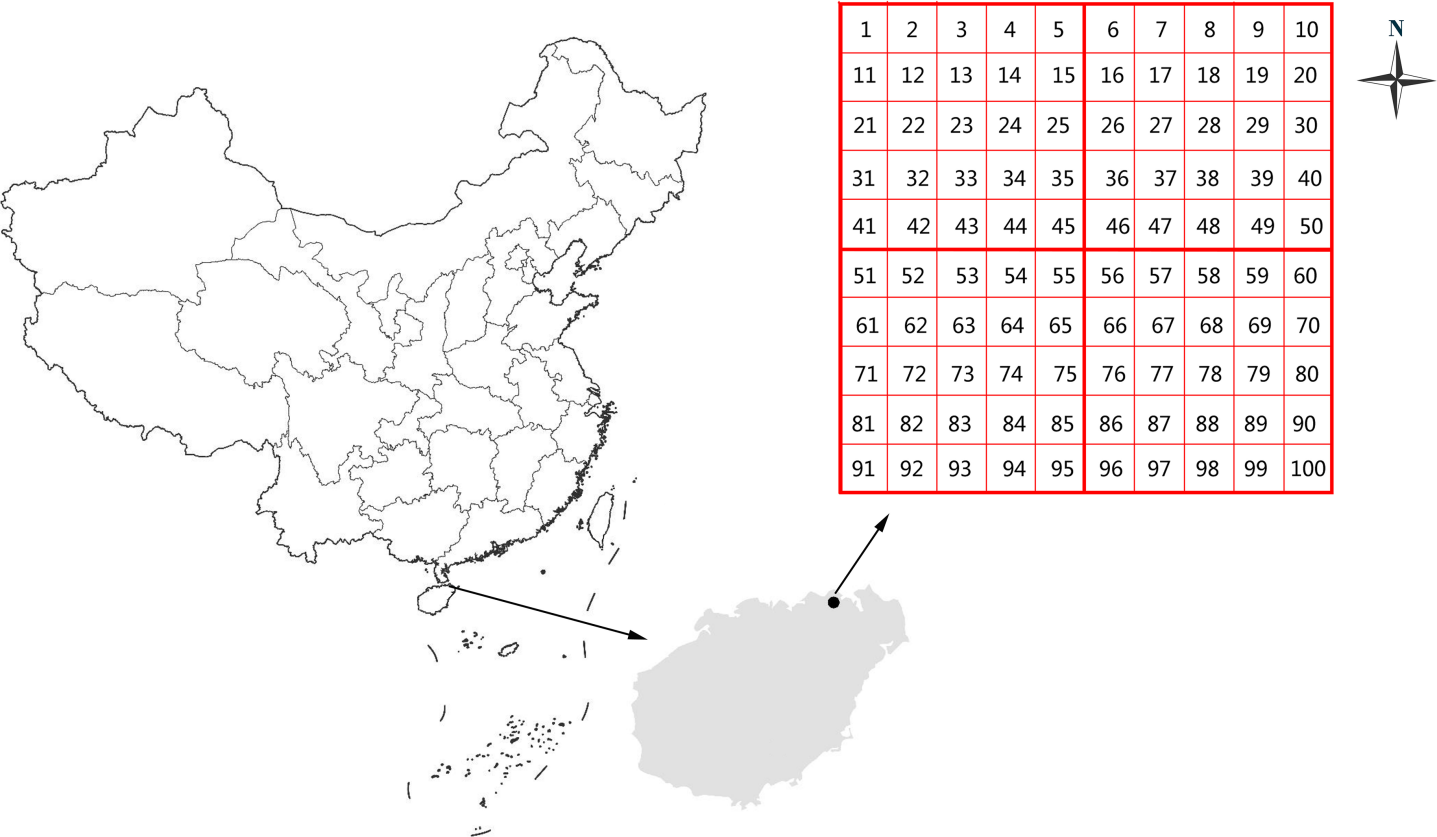
A general overview of Ma’anling volcano park (study site), at Haikou, Hainan Province, China. The grid consisted of 100 quadrats. The length of each quadrat is 1 km.

This study was carried out in the Ma’anling volcano park in the city of Haikou (19°48′-20°01′N, 110°06′-110°27′E), Hainan, China (Fig 1). Hainan is a tropical region in southern China. It is warm throughout the year with abundant rainfall. The mean annual temperature is 23.7 degrees Celsius and the mean annual precipitation is 1,685 mm year^-1^. The precipitation always occurs in summer and autumn. Typhoons often hit this area during rainy seasons. At present, Ma’anling volcano is in a quiescent phase. In the past 30 years, Ma’anling volcano has been subjected to intense tourism. The volcanic soils are shallow, rocky and with a high organic matter. We chose three different vegetation types–plantation forest, secondary forest, and abandoned land–in which to investigate the taxonomic and phylogenetic diversity of the vascular plans as well as the soil properties. Plantation forests are fruit forests that have been recently disturbed; secondary forests are forests that have re-grown after broad scale cutting that occurred twenty years ago; abandoned lands are the areas mainly covered by grass where agricultural activities are no longer viable.

### Field investigation

#### Vegetation sampling

The investigation was performed in a grid of 1 × 1 km quadrats covering the full area of Ma’anling volcano park. It resulted in 100 quadrats in the study site. From each quadrat, we randomly established one 20 × 20 m plot to measure tree species richness, as well tree height, diameter at breast height (DBH, 1.3 m above ground level), and crown width. Within in each of the 20× 20 plots, we randomly established three 5 × 5 m subplots to investigate the shrubs and five 1 × 1 m subplots for the herbs. This way, we set up a total of 100 tree plots, 300 shrub plots and 500 herb plots. We counted the number of species and the abundance of individuals within species. When the coverage of a species was more than 50% in the same plot, we classified it as the dominant species.

The plants species were identified according to *Flora of China* [26]. Nomenclature of family and genus were standardized according to the plant list (www.theplantlist.org) and Angiosperm Phylogeny Group III system [27]. The genus and family distribution types of seed plants were identified by the areal-types of the world families of seed plants and the areal-types of genera and families of Chinese seed plants [28–30]. The distribution types of ferns were identified based on the study by Lu [31].

We considered species as invasive if they are nonnative to the region, introduced by human activities, and lead to ecological problems [32,33]. We classified exotic species based on the study by Yang [34]. Rare / endangered plants were defined based on "China Red List of biological diversity—higher plant volume" evaluation report [35].

#### Soil sampling

The soil samples were collected from the four corners and the center of each of the 20×20 m quadrats. Soil cores were taken from the upper 0–20 cm of the soils using a steel corer with a diameter of 35 mm. The five soil samples within the same quadrats were mixed and then analyzed in a laboratory.

The soil samples were air dried on the surface of envelopes. Large roots, animal and plant residues, litters, and all fine roots were removed from the composite soil samples carefully. Dried soil was sieved through 2 mm mesh screen, and was analyzed for soil pH, soil organic matter (SOM), total soil nitrogen (TN), available phosphor (AP), available potassium (AK) and water content (WC). SOM was analyzed using the method of dichromate oxidation [36]. TN was analyzed using a modified Kjeldahl method [37]. AP was assessed by a modified sequential extraction procedure [38,39]. AK, WC, and soil pH were analyzed according to the procedures specified by the State Forestry Bureau of China [40].

We analyzed the soil property data using Non-metric Multidimensional Scaling (NMDS) analysis and visualized the results through scatter plots using the software R (R version 3.3.3).

#### Building a phylogenetic tree

We assembled a phylogeny of all the angiosperm species at the study site by using Phylomatic3 (http://phylodiversity.net/phylomatic/). The phylogeny was established based on APG III. Branch lengths were adjusted using the Bladj algorithm, with age estimates for the main nodes taken from Wikström et al. [41] and undated nodes spaced evenly among the nodes of known ages as implemented in Phylocom 4.2(http://phylodiversity.net/phylocom/). Since Wikström’s dating does not include ferns, we assigned ages generated by Schuettpelz and Pryer[42] to nodes in the fern part of the phylogeny.

### Data analysis

We quantified taxonomic diversity within each plot using Berger-Parker index (*d*), Shannon index (*He*) and Pielou evenness index (*Je*). Typically, *d* has been used to assess the proportional abundance of species in a community. This index has been used to compare differences of species abundances across communities in the same ecosystem, where community composition may stay relatively constant but individual abundance of each species may vary [43]. Shannon index (*He*) is a common measure of diversity in ecology [44] and sensitive to vegetation structure [45]. Species evenness of a community can be represented by Pielou's evenness index. Values for *d* (1), *He* (2) and *Je* (3) were calculated using the Biodixcel.xlsx program [46] based on the following formulae.

Beger-parkerdd=1nmaxN (1)

d=1nmaxN

$$\mathrm{Shannon}\;\mathrm{index}\;He\;He=-\sum_{i=1}^{s}p_{i}\;\ln\;p_{i}\;p_{i}^{2}=\frac{n_{i}}{N}$$ (2)

$$He=-\sum_{i=1}^{s}p_{i}\;ln\;p_{i}$$

$$p_{i}^{2}=\frac{n_{i}}{N}$$

Pielou'sevennessindexJeJe=HeHemaxHemax=ln⁡s (3)

In the above formulae, *N* refers to the number of all species, *ni* refers to the number of an individual species *i*. *S* refers to the number of species in each plot. *Je* is constrained between 0 and 1. The lower the value of *Je* is, the less evenly the species is distributed in communities.

We also calculated the number of taxa (ntaxa, the number of species), mean phylogenetic distance (MPD), mean nearest taxon distance (MNTD), and PD for each plot. PD is the sum of evolutionary history in millions of years [4]. MNTD is the average distance between an individual and the most closely related (non-conspecific) individual. MNTD is affected by phylogenetic distance in terminal branches [47]. Nearest taxon index (NTI) and net relatedness index (NRI) were calculated using the *picante* package in R [48]. MPD and MNTD values were used to calculate NTI and NRI values, respectively, for each plot.

One Way-ANOVA was used to verify if the type of vegetation formations (i.e., plantation forest, secondary forest and abandoned land) significantly affected plant diversity indices. The NMDS ordination was used to calculate the correlation between physic-chemical properties of soil and taxonomic diversity. Linear regressions were also established between *d*, *He*, *Je*, number of taxa (ntaxa), PD, NRI, NTI, and elevation (El), WC, TN, SOM of the trees, shrubs herbs plots, respectively.

### Ethics statement

Field investigation was carried out in the open air so no specific permission was required. We did not collect plant materials from any privately owned or protected area that required permission. The field study was carried out at Haikou (19°48′-20°01′N, 110°06′-110°27′E) in Hainan Province, China.

## Results

### Species composition

According to our investigation, there were 284 species of vascular plants in the Ma’anling volcano park study site comprising 88 families and 241 genera, although none of these plants were gymnosperms (S1 Table). Plant life forms included trees, shrubs, herbs and vines (contributing to 27.11%, 26.76%, 31.69% and 14.44% of all vascular plant species in this area, respectively). The vegetation was locally dominated by *Dimocarpus longan* Lour., *Melia azedarach* L., *Eucalyptus robusta* Sm., *Diospyros strigose* Hemsl., *Streblus asper* Lour., *Atalantia buxifolia* (Poir.) Oliv., *Lantana camara* L. The herb plots were locally dominated by *Biden spilosa* L., *Chromolaena odorata* (L.) R. M. King & H. Rob., *Pellionia repens* (Loureiro) Merrill, *Saccharum arundinaceum* Retzius, *Cayratia japonica* (Thunberg) Gagnepain, *Tetrastigma pachyphyllum* (Hemsley) Chun, *Merremia vitifolia* (N. L. Burman) H. Hallier. The 14 most species-rich families, where each family had more than 6 species, contributed to 15.91% of the total families, 49.38% of total genera and 53.17% of total species, respectively. Among them, Fabaceae was the most species-rich family (19 genera and 25 species), accounting for 8.80% of all vascular plant species in this area (Table 1).

**Table 1:** Families with more than 6 species.

Family name	Number of genus	Number of species
Fabaceae	19	25
Malvaceae	13	17
Compositae	16	16
Rutaceae	9	12
Moraceae	5	12
Euphorbiaceae	8	10
Rubiaceae	9	9
Apocynaceae	8	9
Gramineae	8	9
Solanaceae	4	8
Phyllanthaceae	6	7
Annonaceae	6	6
Palmae	5	6
Vitaceae	4	6

The 194 native species, belonging to 71 families and 164 genera, accounted for 68.31% of the total species in this area. The 40 cultivated species accounted for another 14.08%. The56 ornamental plants, such as *Allamanda schottii* Pohl, *Canna indica* L., and *Tradescantia zebrine* Bosse, etc., accounted for 19.72% of the total species. There were 23 invasive species, accounting for 8.10% of the total species in this area. There were 4 rare / endangered species: *Aquilaria sinensis* (Lour.) Spreng.,*Dalbergia odorifera* T. Chen., *Dracaena cambodiana* Pierre ex Gagnep. and *Litchi chinensis* Sonn. The first two species have been recorded by the *Chinese species red list*, designated as vulnerable and critically endangered. These two species were also considered vulnerable species in *IUCN Red List* and were designated as second-grade national protected plants in *List of Wild Plants under State Protection*. In addition, *Aquilaria sinensis* (Lour.) Spreng. was recorded at a convention on international trade as an endangered species (S2 Table). *Dracaena cambodiana* Pierre ex Gagnep. was vulnerable and *Litchi chinensis* Sonn was endangered in *List of Wild Plants under State Protection*.

### Floristic elements

In the area, 64.77% of total families are considered tropical elements. Among these, 42 families (47.73%) had pantropic distributions; 6 families (6.82%) had tropical Asia and tropical America disjuncted distributions; 5 families (5.68%) had old world tropics distributions; 2 families (2.27%) had tropical Asia to tropical Australasia distributions; 2 families (2.27%) had tropical Africa and tropical America disjuncted distributions. Temperate elements included 6 families, contributing 6.82% of the flora. The 6 families all had temperate distributions.

**Fig 2:**
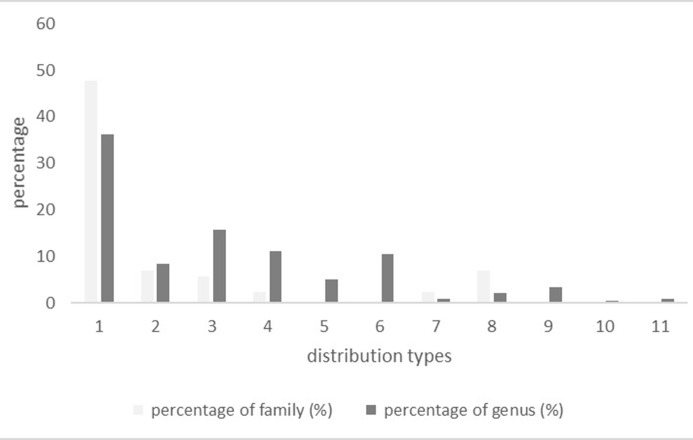
The percentage of plant distribution types at the family and generic levels. 1. Pantropic distribution 2. Tropical Asia and tropical America disjuncted distribution3.Old world tropics distribution 4.TropicalAsia to tropical Australasia distribution 5.Tropical Asia to tropical Africa distribution 6.Tropical Asia (Indo-Malesia) distribution 7.North temperate distribution 8. East Asia and north America disjuncted distribution 9. Center Asia distribution 10. East Asia distribution 11. Tropical Africa and tropical America disjuncted distribution.

At the genus level, tropical elements contributed to 87.55%, and temperate elements contributed to 6.64% of the flora; 87 genera, including *Lantana*, *Ficus*, *Chromolaena*, had pantropic distributions, the most common distribution type among the tropical genera. In contrast, 38 genera, such as *Thunbergia*, *Polyalthia*, *Alangiu*, had old world tropics distributions. Among the rest of the tropical genera, 27, 25, 20, 12, and 2 genera had a tropical Asia to tropical Australasia distribution, a tropical Asia (Indo-Malaysia) distribution, a tropical Asia and tropical America disjuncted distribution, a tropical Asia to tropical Africa distribution, and a tropical African tropical America disjuncted distribution, respectively (Fig 2).

Among temperate genera, 8 genera had east Asia and North America disjuncted distributions, 5 genera had north temperate distributions, 2 genera had east Asia distributions and 1 genus had center Asia distributions. Evidently, the flora in this area was tropical in nature and was characterized by pantropic distributions.

### Plant species diversity and PD

**Fig 3:**
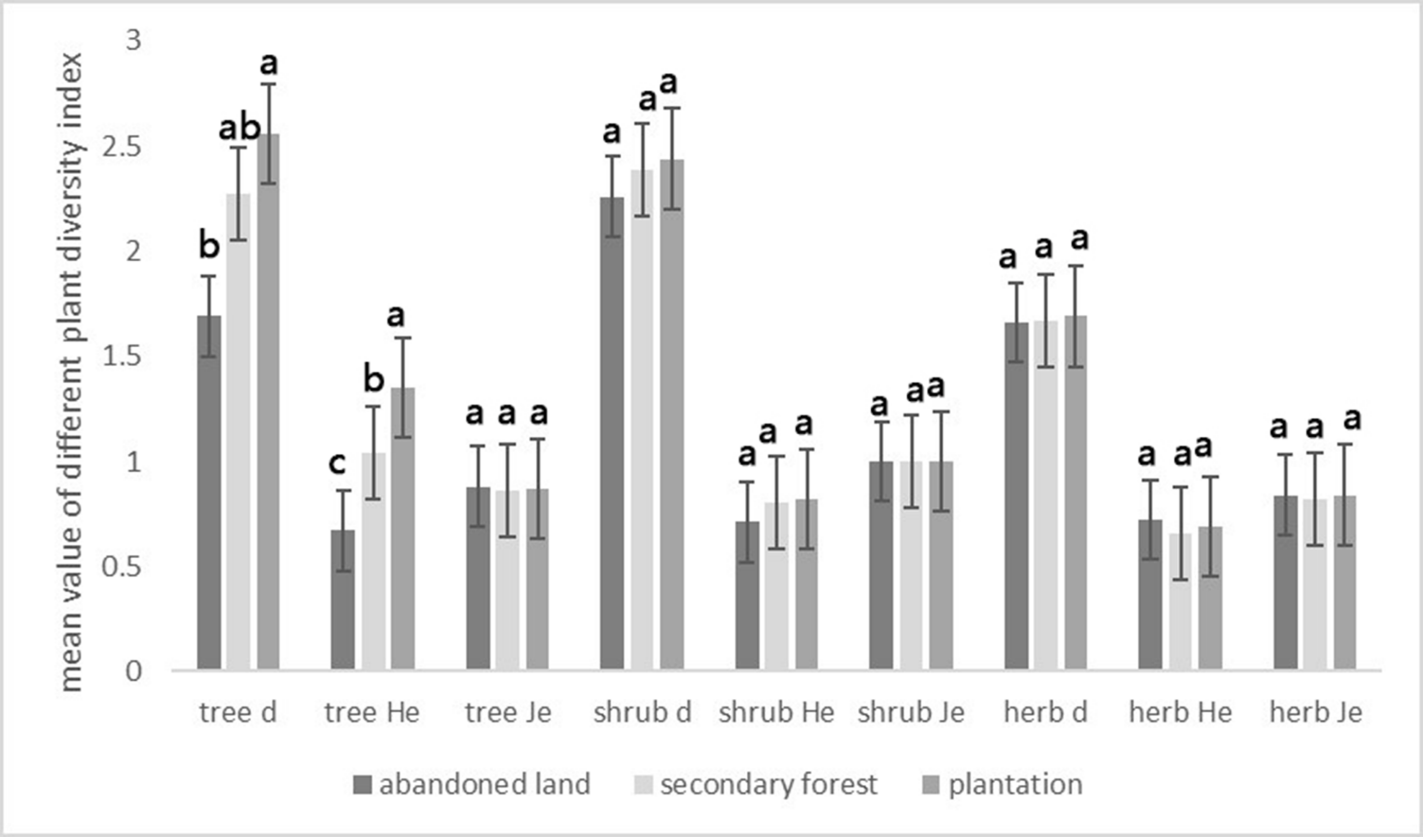
Plant diversity indices of different life forms in different land use types. Error bars were constructed with one standard deviation of the means. Here, *d* refers to Berger-Parker index, *He* refers to Shannon index, *Je* refers to Pielou evenness index. Different letters represented significant differences at *p* = 0.05 among the three different vegetation types.

Plant diversity varied among vegetation formations. The index *d* for trees was significantly different between plantation forest and abandoned land, but not so between secondary forest and plantation forest, or between secondary forest and abandoned land. The difference in index *d* of shrubs and herbs was not significantly different among the three vegetation formations. *He* (Shannon index) of trees was highest in plantations, followed by secondary forest, and was lowest in abandoned land. The differences in *He* of trees among the three vegetation formations were significant: *He* of trees in secondary forest was significantly different from that of plantations and abandoned land (*p*<0.05); and *He* of trees in plantations was also significantly different from that of abandoned land (*p*<0.05). *Je* of all 3 life-styles (trees, shrubs and herbs) among three vegetation formations were not significantly different (Fig 3).

**Fig 4:**
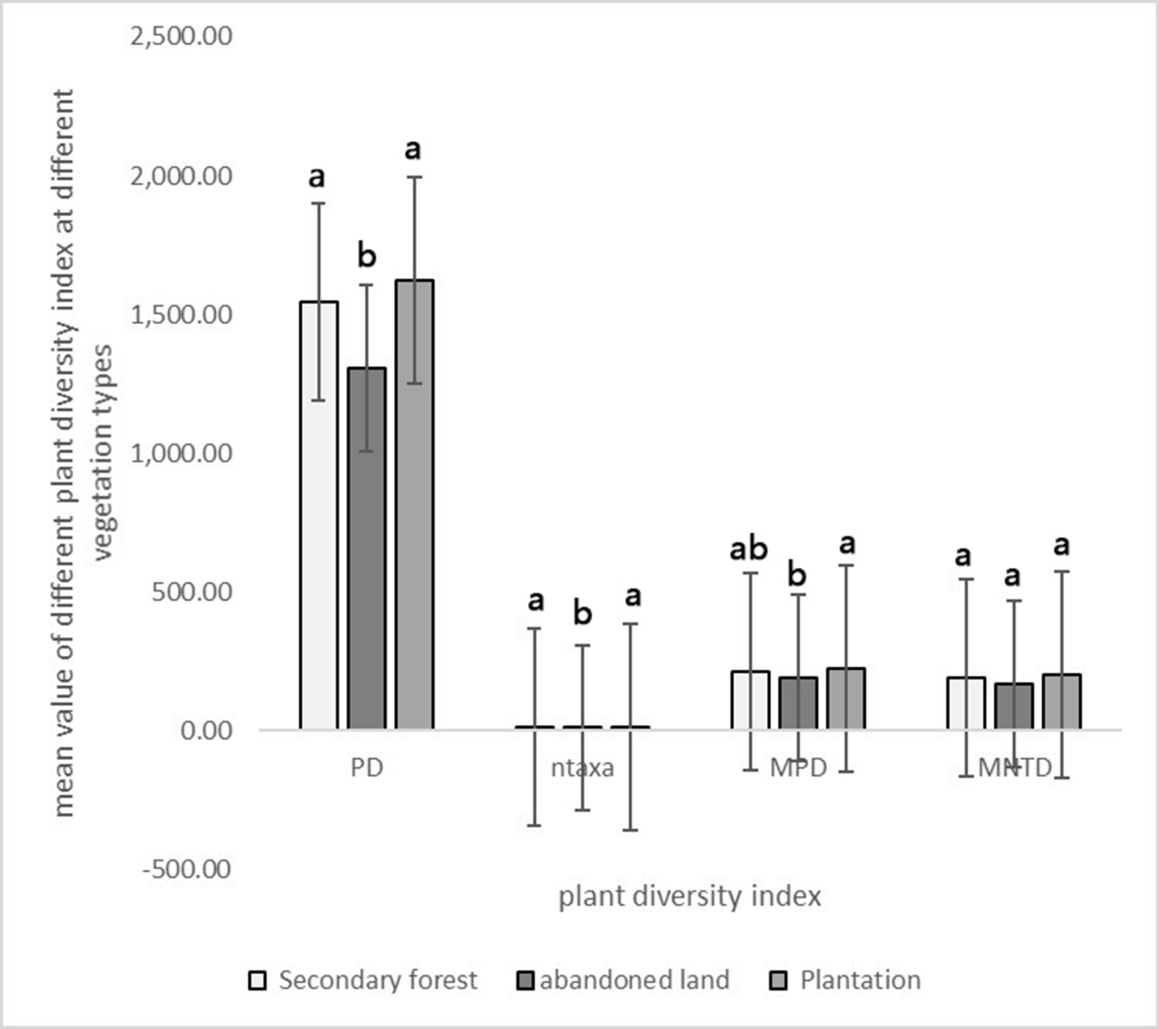
Phylogenetic diversity indices for different vegetation types. Error bars were constructed with one standard deviation of the means. PD refers to phylogenetic diversity. Ntaxa refers to the number of species. MPD refers to mean phylogenetic distance. MNTD refers to mean nearest taxon distance. Different letters represented significant differences at p = 0.05 among three different vegetation types.

The results of our phylogenetic analyses showed that PD, ntaxa, MPD and MNTD of secondary forest, abandoned land and plantation forest were 1,546.33, 130.87, 1,620.81; 12.43, 10.07, 13.20; 213.70, 189.16, 225.38; 190.84, 167.83, 201.65, respectively (Fig 4). PD and ntaxa were highest in plantation forest and lowest in abandoned land. PD and ntaxa in plantation forest and secondary forest were not significantly different from each other; however, they were both significantly different from the PD and ntaxa of abandoned land. MPD in plantation forest was significantly different from abandoned land. MPD in secondary forest was not significantly different from MPD of plantation and abandoned land. MNTD was highest in plantation forest and lowest in abandoned land, but the differences in MNTD between the three vegetation formations were not significant.

### The correlation between plant diversity and soil properties

NMDS ordinations results showed that El, WC, TN and SOM had a highly significant effect on plant diversity (*p* = 0.001). AK had a significant effect on plant diversity (*p* = 0.040). We made scatter plots based on all surveyed plots, including all three plant life forms, all vegetation formations, and all soil physio-chemical properties measured. NRI decreased significantly (*p* < 0.05) with elevation (El) (*p* = 0.005), and water content (WC) (*p* = 0.046), marginally significantly (*p* < 0.1) decreased with total nitrogen (TN). NTI decreased significantly with TN, El and WC, whereas *d* of shrub increased significantly with SOM, marginally significantly increased with WC and TN. PD significantly increased with El and WC, marginally significantly increased with SOM. *He* significantly increased with TN, marginally significantly increased with SOM. Ntaxa significantly increased with El and WC (Table 2, Fig 4).

**Table 2:** Nonmetric Multidimensional Scaling (NMDS) ordination based on soil physic-chemical properties and plant diversity, WC: Water content; TN: Total nitrogen; AP: Available phosphor; AK: Available potassium; SOM: Soil organic matter.

	NMDS1	NMDS2	r2 Pr(>r)	*p*
El	0.987	0.163	0.276	0.001***
WC	0.966	-0.258	0.282	0.001***
TN	0.991	-0.1306	0.221	0.001***
AP	-0.930	-0.368	0.049	0.082
AK	0.955	-0.298	0.061	0.040*
SOM	0.990	-0.143	0.288	0.001 ***
pH	-1.000	-0.026	0.055	0.053

Footnote

* means difference is significant at the 0.05 level and

*** means difference is significant at the 0.001 level.

The WC, TN, AP, AK, SOM and pH across plots ranged between 1.02–39.66%, 1.53–1561.19 μg/g, 0.05–79 μg/g, 91.39–1570.39 μg/g, 0.51–25.29%, 3.92–8.13, respectively (S2 Table). The coefficient of variance (CV) of the physic-chemical parameters ranged from 0 to 1, except that AP had a CV greater than 1.

**Fig 5:**
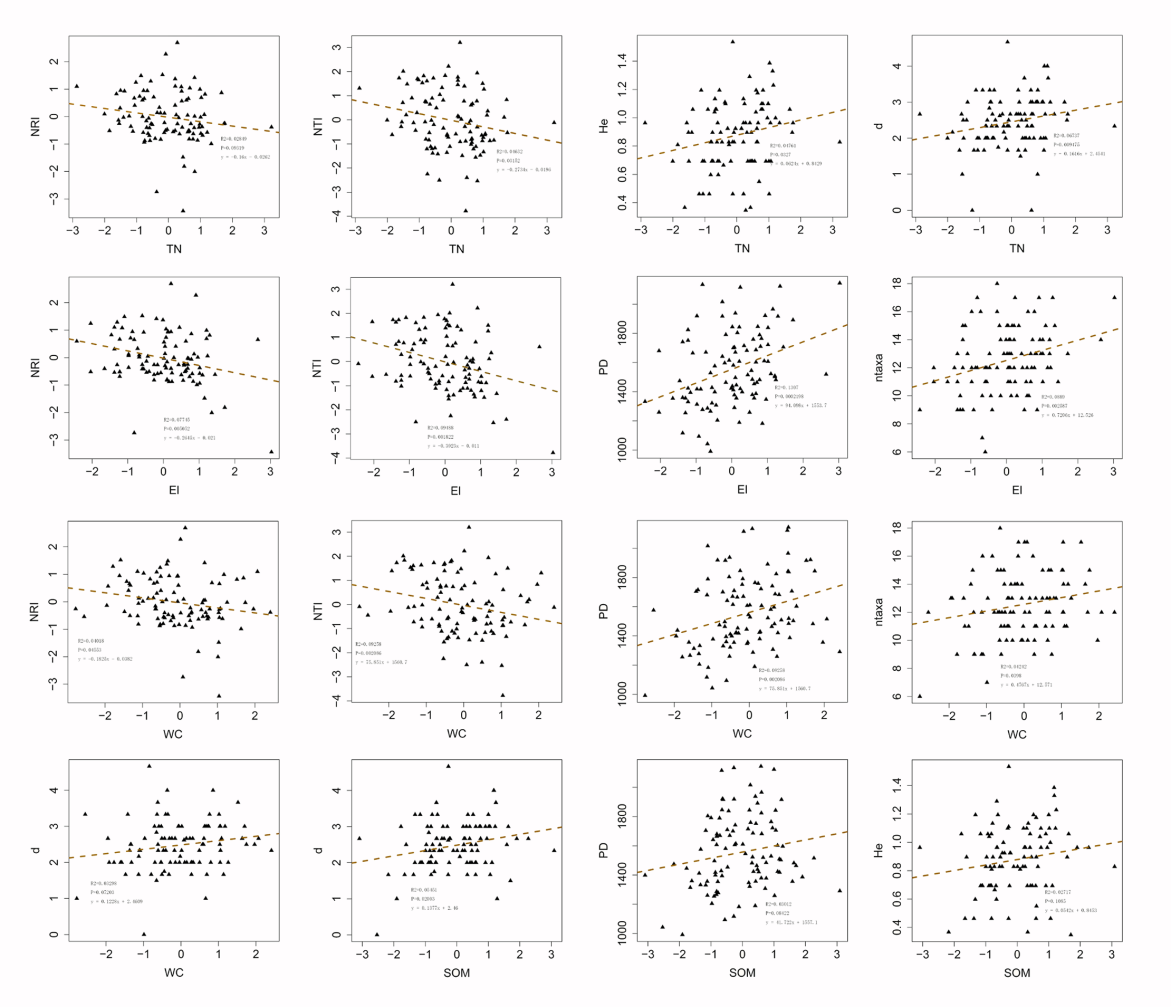
Scatter plots of net relatedness index (NRI), nearest taxon index (NTI), Shannon index (*He*), Beger-parker index (*d*), phylogenetic diversity (PD), number of taxa (ntaxa, the number of species) and soil physic-chemical properties. The dependent variable include: taxonomic and phylogenetic diversity(d, He, Je, PD, ntaxa, NRI, NTI) of different life forms(tree, shrub, herb, all species). The independent variables include: WC, TN and SOM. Single factor regression analysis were established.

The *d* of shrubs was significantly positively correlated with TN, SOM and WC (R^2^ equaled to 0.067, 0.055, and 0.0330, respectively). PD was significantly positively correlated to El and WC. The positive correlation between PD and El was highly significant, with a R^2^ value of 0.131; the correlation between PD and WC was weaker than the correlation between PD and El, with an R^2^ value of 0.093 (Fig 5).

## Discussion

### Comparison of floristic composition to adjacent natural parks and areas

Floristic composition in the study site was compared to four adjacent natural parks: Tongguling Nature Reserve (TNR), Wuzhishan Nature Reserve (WNR), Jianfengling Nature Reserve (JNR) and Diaoluoshan Nature Reserve (DNR), Hainan. The floristic composition was similar to TNR at generic level, which both were dominated by Pantropic distributions [49]. It was different from WNR, DNR and JNR, which were dominated by a Tropical Asian (Indo-Malesian) distribution. No Chinese endemic genus appeared in the study site, and only one appeared in TNR, while DNR, JNR and WNR had 10, 15, 16, respectively. Two factors might have contributed to the floristic differences among the parks. First, we noted that both our study site and the TNR were severely disturbed by human activities. Second, the habitat conditions at DNR, JNR and WNR were very different from the study site and TNR. The altitude of DNR, JNR and WNR are higher than 1,200 m, while the altitude of our study site and TNR are much lower. Therefore, mean annual temperature in the study site and TNR were a bit higher than that of DNR, JNR and WNR.

Comparing floristic composition in the study site to Vietnam and to two adjacent provinces of China, namely Guangdong and Guangxi, it was found that floristic composition in the study site was most similar to Guangdong, followed by Vietnam [50]. The native genera and native species were compared among these four areas. There were 164 native genera in the study site. Genus *Flueggea* was not found in Vietnam. Genus *Dracaena* was not found in Guangdong. Genus *Harrisonia* was not found in Guangxi. At the level of species, there were 194 native species in the study site, 7 of them were not found in Guangdong, 12 of them were not found in Vietnam and 14 of them were not found in Guangxi. Only one species of *Lithocarpus naiadarum* (Hance) Chun was not found in theses 3 areas. This observation suggests that the study site has very low endemism at the generic and species levels, which indicated the continental origin of floristic composition.

### Taxonomic diversity and PD

High *d* values indicate that communities were dominated by a few species. *d* of trees was significantly different between plantation forests and abandoned lands. *He* of trees varied significantly among three different vegetation types. The plant composition in Haikou, China were influenced by the frequency and the type of land use changes[9]. In many Chinese cities and the surrounding area, human management and environmental factors was closely related to plant diversity [51]. Different fertilizer application, pests and pathogen attacks caused different habitat conditions among the vegetation formations [52] and lead to different plant diversity. The plantation forests in the area included fruit trees such as *Litchi chinensis* Sonn., *Dimocarpus longan* Lour. and other commercial trees. This generated the difference of *d* and *He* among the vegetation formations. In contrast, *d* and *He* of shrubs and herbs were not significantly different among the different vegetation formations due to the fact that plantation forests were unmanaged for decades [53]. On the other hand, as one of the important tourist attractions at Haikou, Ma’anling, the volcanic area experienced an influx of tourists, which impacted the plant diversity. Christ et al. pointed out that tourism-related activities lead to loss of biodiversity[54], particularly in the coastal and mountain areas[55]. In the study site, the amount of ornamental plants is large because of tourism. At the same time, exotic species and invasive species were introduced for aesthetics and recreation. For instance, *Oxalis corymbose* DC. and *Axonopus compressus* (Sw.) Beauv. were both invasive species.

PD, ntaxa, MPD and MNTD values were highest in the plantation forests. This showed the plant diversity was high; phylogenetic structure was over-dispersed due to great phylogenetic distance between species (NTI < 0, *p*> 0.975). The plantation forests experienced no management activities for decades. Unmanaged plantation forests are very important for biodiversity maintenance [56]. No management activities improve the conditions for species dispersal [57] and increase species diversity [9]. In the study site, a high abundance of shrubs and herbs caused by the lack of management activities, combined with the fact that commercial trees planted decades ago still remained, have contributed to the high plant diversity. Most commercial trees were introduced from the other regions by humans. These species were more distantly related to at least one of the other commercial species.

Thus, prior artificial intervention, followed by long-term natural growth, led to the emergence of plantation forests, characterized by distant interspecific associations and divergent lineages. The PD, ntaxa, MPD and MNTD values of secondary forests were larger, reflecting the abundance of species richness in secondary forest communities, closer phylogenetic relationship among species, and relative aggregation of lineages structure. The secondary forests have been disturbed significantly through intensive logging and clear-felling in decades. They were dominated by shrubs, and only a few trees and vines. Length and continuance of re-growth time of secondary forests can be critical to the recovery of species diversity and composition. The short restitution time and the shallow and rocky soil impacted both the diversity and composition of the secondary forests. PD, ntaxa, MPD and MNTD were at their minimum in abandoned land, which indicated that the phylogenetic structure is clustered and plant diversity is low. These results could have occurred because abandoned lands are a more stressful environment for plants. Given that species sharing traits within close phylogenetic lineages may have similar responses to the environment, severe environmental conditions are likely to sort species out, leading to communities with low species richness and low PD [58,59]. PD may be related to the distribution pattern of species richness in abandoned land. Lower species richness in a community lead to smaller PD.

### The relationship between soil properties and plant diversity

Soil properties affect plant diversity. AP was highly variable among the 100 samples, with a CV value of 2.68 and stable pH with a CV value of 0.13 (the sample variability is weak when CV≤0.01, medium when 0.01<CV≤1 and strong when CV≥1). Compared to other regions, TN was extremely low, as were AP and SOM. However, AK is very high, because atmospheric deposition at northern Hainan leads to potassium enrichment [60]. Furthermore, the soil is shallow and rocky in the study site (Table 3). These results showed that the soil in the study site was young and low in nutrient availability, which was similar to northern Japan, where the soil was derived from immature volcanic ash [61]. Young soil always lacks nitrogen and Phosphor[62,63]. However, soil at Ma’anling volcanic area was acidic except for five neutral spots and one alkaline spot. Phosphor is easily absorbed or fixed in acidic soil [64], leading to a low AP amount at the study site. Another study in southern China also showed that, compared to natural forests, disturbed forests have lower availabilities of soil nutrients, particularly nitrogen and Phosphor[65–68].Therefore, the plants at Ma’anling area suffer poor soil conditions. A plant community tends to be less diverse on young soils [69].

**Table 3:** Summary statistics of soil physic-chemical properties, WC: Water content; TN: Total nitrogen; AP: Available phosphor; AK: Available potassium; SOM: Soil organic matter, coefficient of variance (CV).

Indicator	Maximum	Minimum	Mean	Standard deviation	CV
WC %	39.66	1.02	18.33	8.10	0.44
TN (μg/g)	1561.19	1.53	359.67	269.35	0.75
AP (μg/g)	79	0.05	3.14	8.41	2.68
AK (μg/g)	1570.39	91.39	698.35	324.90	0.47
SOM %	25.29	0.51	6.55	5.01	0.76
pH	8.13	3.92	5.54	0.73	0.13

#### Plant diversity under poor nutrient soil conditions

Plant diversity at the study site was significantly positively correlated with El, WC, TN, and SOM, and marginally significantly positively correlated with AK. TN, SOM and AK were all important soil nutrients and resources for plants. Two or more limiting resources determine the number of coexistence plant species[70]. Our results showed that plant diversity was significantly impacted by soil nutrition. Soil affected plant species diversity through many different mechanisms[69]. Soil properties and soil nutrition affect the plants in many ways. The surface soil of forest has a strong ability to accumulate nutrients[71]. At our study site, *d* of shrubs was strongly affected by TN and SOM, which means TN and SOM play a key role in the evenness and richness of shrubs. Previous research has shown that nitrogen was positively correlated with SOM [72]. Both TN and SOM were essential elements for plant growth. According to the theory of “productivity-diversity”, nutrient availability and stoichiometry drives primary productivity, which in turn affects the rate at which plant species can be replaced competitively from a community [73,74]. Many studies have documented that low nitrogen content restricts the composition and replacement of the species[75,76]. The nitrogen and organic carbon in soil mainly came from litter, plant roots, soil animal and microorganism residues, and precipitation, found primarily in the surface soil [77,78]. Therefore, the change of nitrogen and SOM content had a great influence on shrubs and other shallow root plants. The effect of plant diversity on the belowground resource availability and particularly the fine characteristics of soil organic matter, has been overlooked; studies on the effects of environmental gradients on soil biodiversity generally disregard the importance of changes in plant diversity on these fine characteristics of soil organic matter [79].

However, not all plant diversity indices were affected by soil properties in the study site. The responses of plant diversity to soil factors varied among plant life forms. Compared to trees and herbs, the *d* of shrubs was more sensitive to soil properties; it was significantly correlated with TN, SOM and WC. The soil samples were collected from soil surface (0–20 cm deep) in this study. Surface soil had little effect on trees since they have deep roots. However, most of the root systems of shrubs were within the range of 0–20 cm. This might explain why the shrubs were more affected by soil properties in this study. Although the distribution of the grass roots was similar to shrubs, the *d* of grass had no significant correlation to soil properties. The difference between herbs and shrubs could be explained by their difference in ecological habits. The dominant herb species at the study site were invasive species such as *Biden spilosa* L. and *Chromolaena odorata* (L.) R. M. King et H. Rob. Invasive plants tend to be more tolerant to barren soil [80], and are apt to form single dominant species communities [81]. Communities with a single dominant species have low diversity, and the diversity index tends to be less sensitive to soil properties. Therefore, the diversity of herbs was likely easily affected by invasive plants.

The NRI and NTI reflects the phylogenetic relatedness between species within a region, echoing recent evolutionary events that structure communities [82,83]. A study on China’s grasslands showed that soil tended to be more important in regulating PD [84]. In our study, NRI and NTI were negatively associated with TN and WC. WC of soil played a more important role in shaping PD within the three different land use types. According to the current study, WC was an important fine-scale factor affecting plant growth, composition and diversity [53,85,86]. Other researchers have similarly determined that WC is the best predictor of plant diversity [87,88].

## Conclusions

We used a combination of approaches to understand taxonomic and phylogenetic diversity of vascular plants in the Ma’anling volcano area of Hainan and how the diversity responded to volcanic soil. In our study site, plant richness and composition were significantly affected by elevation (El), soil water content (WC), total soil nitrogen (TN) and soil organic matter (SOM). Plant diversity significantly varied among different vegetation formations. Further research combining various diversity metrics will benefit our understanding of the ecological and evolutionary processes that shape plant diversity, which in turn, is an important property of ecosystems. Our study revealed the links between diversity, community structure, and key abiotic factors; i.e., soil properties as determined using field studies in tropical natural parks.

## Supporting information

S1 Table List of vascular plants in Ma’anling volcano area of Haikou, China. (XLSX)

S2 Table List of physio-chemical properties of soil in Ma’anling volcano area of Haikou, China. WC: water content; TN: total nitrogen; AP: available phosphor; AK: available potassium; SOM: soil organic matter.
(XLSX)
