# Correction: Taxonomic and phylogenetic diversity of vascular plants at Ma'anling volcano urban park in tropical Haikou, China: Reponses to soil properties

**DOI:** 10.1371/journal.pone.0209520

**Published:** 2018-12-14

**Authors:** Xia-Lan Cheng, Lang-Xing Yuan, Mir Mohammad Nizamani, Zhi-Xin Zhu, Cynthia Ross Friedman, Hua-Feng Wang

S3, S4, S5 and S6 Files are omitted from the list of Supporting Information. All four files can be viewed below.

## Supporting information

S3 File The one hundred plot records in Maanling Valcano area, Haikou, China (1 of 3). (PDF)

S4 File The one hundred plot records in Maanling Valcano area, Haikou, China (2 of 3). (PDF)

S5 File The one hundred plot records in Maanling Valcano area, Haikou, China (3 of 3). (PDF)

S6 File Minimal data set. (ZIP)
