# Supplementary material for: Correction: Taxonomic and phylogenetic diversity of vascular plants at Ma'anling volcano urban park in tropical Haikou, China: Reponses to soil properties
Source: PLoS One. 2018 Dec 14;13(12):e0209520. doi: 10.1371/journal.pone.0209520 (PMC6294358; doi:10.1371/journal.pone.0209520)
Supplement: S3 File — (PDF) [file pone.0209520.s001.pdf]

总表

<table>
<tr><td>群落名称
乔-灌-草
优势种</td><td colspan="4">土坛树—鹊肾树—藿香蓟</td><td>野外编号
(统一编号)</td><td colspan="2">1</td></tr>
<tr><td>记录者</td><td colspan="2"></td><td>日期</td><td>2017.1.7
10：00</td><td>室内编号</td><td colspan="2"></td></tr>
<tr><td>样地面积</td><td colspan="2">20×20 m</td><td>详细地点</td><td colspan="4"></td></tr>
<tr><td>GPS 定位</td><td colspan="2">N：19°57.820′
E：110°10.566′</td><td>海拔高度</td><td colspan="4">48 m</td></tr>
<tr><td>群落高度</td><td colspan="3"></td><td>群落的总盖度</td><td colspan="3">90%</td></tr>
<tr><td>主要层优势种</td><td colspan="7">乔木层：
灌木层：
草本层：</td></tr>
<tr><td>群落外貌特点</td><td colspan="7">杂草丛生，草本植物丰富</td></tr>
<tr><td>小地形及样地周围环境描述</td><td colspan="7">荒废农田池塘</td></tr>
<tr><td rowspan="5">分层及各层的特点</td><td>乔木层</td><td>高度</td><td colspan="5"></td></tr>
<tr><td>灌木层</td><td>高度</td><td colspan="5"></td></tr>
<tr><td>草本层</td><td>高度</td><td colspan="5"></td></tr>
<tr><td>层间植物</td><td>高度</td><td colspan="5"></td></tr>
<tr><td></td><td>高度</td><td colspan="3"></td><td></td><td></td></tr>
<tr><td>备注（之前的土地利用状况）</td><td colspan="7">土壤鲜重：0.16 kg</td></tr>
</table>

说明：数据尽可能填写全面，没有填无

乔木层植物群落调查表

群落名称：木棉　样方面积：20 m × 20 m　野外编号：1

调查时间：2017.01.07　10：00　记录者：　室内编号：

编号	植物名称	高度（m）	胸径（cm）	冠幅（m）	物候期	生活力	备注
1	钝叶鱼木	3	10	3×2	叶	3	
2	木棉	3	8	2×3	叶	3	
3	木棉	2	10	2×3	叶	3	
4	木棉	3	10	3×2	叶	2	
5	番石榴	2.5	8	3×4	叶	2	
6	土坛树	5	10	6×4	叶	2	
7	对叶榕	3	6	3×2	叶	2	
8							
9							
10							
11							
12							
13							
14							
15							
16							
17							
18							
19							
20							
21							
22							
23							
24							
25							
26							
27							
28							
29							
30							
31							
32							
33							
34							
35							

灌丛层植物群落调查表

群落名称：鹊肾树　　样方面积：5 m × 5 m　　野外编号：1

调查时间：2017.01.07　10：00　　记录者：　　室内编号：

编号	植物名称	高度（cm）	冠径（cm）	物候期	生活力	盖度%	株数 / 丛树
1	鹊肾树	150	70	叶	2	20	2
2							
3	鹊肾树	120	50	叶	2	30	1
4	鹊肾树	140	60	叶	2	40	1
5							
6	山小橘	150	50	叶果	2	40	1
7	福建茶	150	60	叶	2	30	1
8							
9							
10							
11							
12							
13							
14							
15							
16							
17							
18							
19							
20							
21							
22							
23							
24							
25							
26							
27							
28							
29							
30							

草本层植物群落调查表

群落名称：　厚叶崖爬藤-藿香蓟　　样方面积 1 m × 1 m　　野外编号：1

调查时间：2017.01.07　10：00　　记录者：　　室内编号:

编号	植物名称	株高(cm)	盖度(%)	物候期	生活力	备注
1	短柄吊球草	20	5	叶果	2	
2	一点红	30	5	叶花	2	
3	藿香蓟	40	60	叶花	1	
4	鬼针草	30	20	叶花	1	
5	空心莲子草	5	30	叶花	2	
6						
7	蛇葡萄	20	30	叶花	1	
8	毛草龙	10	5	叶果	2	
9						
10	叶下珠	15	60	叶果	2	
11	厚叶崖爬藤	5	80	叶	1	
12						
13	倒地铃	20	10	叶果	1	
14	土牛膝	40	50	叶果	2	
15						
16	百花丹	50	60	叶花	2	
17	含羞草	20	50	叶花果	2	
18						
19						
20						
21						
22						
23						
24						
25						
26						
27						
28						
29						
30						

说明：物候期：花、叶、果

生活力：1　良好 2 一般 3 较差

总表

群落名称乔-灌-草优势种	高山榕—马缨丹—斑茅				野外编号(统一编号)	2
记录者			日期	2017.01.07 9:00	室内编号	2
样地面积	20×20 m		详细地点			
GPS 定位	N：19°58.175′ E：110°10.960′	海拔高度	54 m			
群落高度				群落的总盖度	95%	
主要层优势种	乔木层：<br>灌木层：<br>草本层：					
群落外貌特点	乱石场旁，地表贫瘠					
小地形及样地周围环境描述	石矿，村旁，次生林					
分层及各层的特点	乔木层	高度				
	灌木层	高度				
	草本层	高度				
	层间植物	高度				
		高度				
备注（之前的土地利用状况）	土壤鲜重：0.12 kg					

说明：数据尽可能填写全面，没有填无

乔木层植物群落调查表

群落名称：　番石榴　　　样方面积：20 m × 20 m　　野外编号：22
调查时间: 2017.01.07　9:00　记录者：　　　　　室内编号：2

编号	植物名称	高度（m）	胸径（cm）	冠幅（m）	物候期	生活 2 力	备注
1	高山榕	6	40	6×4	叶	2	
2	细基丸	2.5	4	1×2	果	1	
3	毛八角枫	5	6	3×2	叶	2	
4	番石榴	4	4	3×2	叶	2	
5	番石榴	3	4	1×2	叶	2	
6	番石榴	4	5	3×2	叶	2	
7	番石榴	5	6	4×3	叶	2	
8	山黄麻	2	3	1×1	叶	2	
9							
10							
11							
12							
13							
14							
15							
16							
17							
18							
19							
20							
21							
22							
23							
24							
25							
26							
27							
28							
29							
30							
31							
32							
33							
34							
35							

灌丛层植物群落调查表

群落名称：马樱丹　　样方面积：5 m × 5 m　　野外编号：2
调查时间：2017.01.07　9:00　　记录者：　　室内编号：

编号	植物名称	高度（cm）	冠径（cm）	物候期	生活力	盖度%	株数/丛树
1	鸦胆子	600	60	叶	1	10	1
2	马缨丹	200	200	叶花	1	80	2
3	对叶榕	150	100	叶	2	60	1
4							
5	黄牛木	60	40	叶	3	10	1
6	酒饼簕	40	30	叶果	2	10	1
7	马缨丹	60	50	叶花	1	50	1
8							
9	牛筋果	200	300	叶	1	80	1
10	酒饼簕	100	30	叶果	2	20	1
11							
12							
13							
14							
15							
16							
17							
18							
19							
20							
21							
22							
23							
24							
25							
26							
27							
28							
29							
30							

草本层植物群落调查表

群落名称：　白花鬼针草-鸭跖草-无根藤　　样方面积 1 m × 1 m　　野外编号：2
调查时间：2017.01.07　9:00　　记录者：　　室内编号：

编号	植物名称	株高(cm)	盖度(%)	物候期	生活力	备注
1	无根藤	200	60	花果	2	
2	飞机草	150	50	叶花	2	
3	一年蓬	20	10	叶	2	
4						
5	田基黄	20	10	叶花	2	
6	丰花草	10	10	叶花	2	
7						
8	鸭趾草	5	60	叶花	1	
9	独脚金	5	1	叶花	2	
10						
11	白花鬼针草	50	60	叶花	1	
12	墨苜蓿	5	10	叶花	1	
13	蝙蝠草	20	20	叶花	2	
14						
15	天门冬	20	20	叶	1	
16	斑茅	200	80	叶花	1	
17	飞扬草	10	5	叶花	2	
18	长春花	30	20	叶花	1	
19						
20						
21						
22						
23						
24						
25						
26						
27						
28						
29						
30						

说明：物候期：花、叶、果
生活力：1　良好 2　一般　3　较差

总表

群落名称<br>乔-灌-草<br>优势种	荔枝—矮紫金牛—假蒟			野外编号<br>(统一编号)	3
记录者		日期	2017.01.06<br>9:25	室内编号	
样地面积	20×20 m	详细地点			
GPS 定位	N：19°57.864′<br>E：110°11.811′	海拔高度	56 m		
群落高度		群落的总盖度		90%	
主要层优势种	乔木层：<br>灌木层：<br>草本层：				
群落外貌特点	人工林				
小地形及样地周围环境描述	地势复杂，坑地较多，附近有采石场				
分层及各层的特点	乔木层	高度			
	灌木层	高度			
	草本层	高度			
	层间植物	高度			
		高度			
备注（之前的土地利用状况）	土壤鲜重：0.10 kg				

说明：数据尽可能填写全面，没有填无

乔木层植物群落调查表

群落名称：荔枝　　样方面积：20 m × 20 m　　野外编号：3

调查时间：2017.01.06　9:25　　记录者：　　室内编号：

编号	植物名称	高度（m）	胸径（cm）	冠幅（m）	物候期	生活力	备注
1	荔枝	8	20	4×3	叶	1	
2	荔枝	7	18	3×3.5	叶	1	
3	荔枝	8	19	4×3	叶	1	
4	荔枝	8	20	4×4	叶	1	
5	荔枝	9	18	3×4	叶	1	
6	荔枝	8	19	4×4	叶	1	
7	荔枝	8	20	4×3	叶	1	
8	荔枝	7	20	4×3	叶	1	
9	荔枝	7	20	4×3	叶	1	
10	荔枝	8	21	3×4	叶	1	
11	荔枝	8	23	4×4	叶	1	
12	荔枝	8	20	4×4	叶	1	
13	荔枝	8	21	4×4	叶	1	
14	荔枝	8	22	4×3.5	叶	1	
15	荔枝	8	20	3×4	叶	1	
16	荔枝	9	23	4×5	叶	1	
17	荔枝	8	21	4×3	叶	1	
18	荔枝	7	18	3×3	叶	1	
19	荔枝	7	19	3×3.5	叶	1	
20	荔枝	8	20	4×4	叶	1	
21	荔枝	9	22	4×5	叶	1	
22	荔枝	8	21	4×3.5	叶	1	
23	荔枝	8	19	3×4	叶	1	
24	荔枝	8	20	3×4	叶	1	
25	荔枝	8	21	3×3.5	叶	1	
26	荔枝	7	19	4×3	叶	1	
27	荔枝	9	20	4×5	叶	1	
28	荔枝	8	21	4×3	叶	1	
29	荔枝	9	22	3×4	叶	1	
30	荔枝	8	20	3×4	叶	1	
31							
32							
33							
34							
35							

灌丛层植物群落调查表

群落名称：粗糠柴-矮紫金牛　　样方面积：5 m × 5 m　　野外编号：3
调查时间：2017.01.06　9:25　　记录者：　　室内编号：

编号	植物名称	高度（cm）	冠径（cm）	物候期	生活力	盖度%	株数/丛树
1	粗糠柴	180	65	叶	2	30	1
2	粗糠柴	230	60	叶	2	30	1
3	粗糠柴	180	63	叶	2	30	1
4	两面针	160	55	叶	2	30	1
5	两面针	165	53	叶	2	30	1
6	矮紫金牛	50	25	叶	2	50	1
7	土蜜树	172	150	叶	2	60	1
8	大花紫玉盘	180	250	叶	1	10	1
9							
10	矮紫金牛	30	20	叶	2	20	1
11	矮紫金牛	80	53	叶	2	30	1
12	矮紫金牛	70	45	叶	2	30	1
13	矮紫金牛	75	45	叶	2	30	1
14	矮紫金牛	75	40	叶	2	25	1
15	鹊肾树	165	20	叶	1	15	1
16							
17	大管	55	25	叶	2	20	1
18	暗罗	60	20	叶	2	20	1
19	福建茶	20	30	叶	2	10	2
20	潺槁木姜子	60	35	叶	2	20	1
21							
22							
23							
24							
25							
26							
27							
28							
29							
30							

草本层植物群落调查表

群落名称：假蒟-斑茅　　样方面积 1 m × 1 m　　野外编号：3
调查时间：2017.01.0.6　9:25　　记录者：　　室内编号:

编号	植物名称	株高(cm)	盖度(%)	物候期	生活力	备注
1	假蒟	40	80	叶	2	
2	飞机草	65	10	叶	2	
3						
4	厚叶崖爬藤	100	30	叶	2	
5	蜈蚣藤	50	20	叶	2	
6						
7	假蒟	35	80	叶	2	
8	吐烟花	5	20	叶	2	
9						
10	海南茄	80	20	叶	2	
11	斑茅	130	70	叶花	2	
12						
13	假蒟	35	80	叶	1	
14	天门冬	15	20	叶	2	
15						
16						
17						
18						
19						
20						
21						
22						
23						
24						
25						
26						
27						
28						
29						
30						

说明：物候期：花、叶、果
生活力：1　良好 2　一般　3　较差

总表

群落名称<br>乔-灌-草<br>优势种	秋枫—降香-微甘菊			野外编号<br>(统一编号)	4
记录者		日期	2017.01.06<br>11：30	室内编号	
样地面积	20×20 m	详细地点			
GPS 定位	N：19°58.028′<br>E：110°12.210′	海拔高度	53 m		
群落高度		群落的总盖度	70%		
主要层优势种	乔木层：<br>灌木层：<br>草本层：				
群落外貌特点	人工林				
小地形及样地周围环境描述	周边是石料加工厂，砍伐严重，一片荒芜				
分层及各层的特点	乔木层	高度			
	灌木层	高度			
	草本层	高度			
	层间植物	高度			
		高度			
备注（之前的土地利用状况）	土壤鲜重：0.18 kg				

说明：数据尽可能填写全面，没有填无

乔木层植物群落调查表

群落名称：龙眼-秋枫　　样方面积：20 m × 20 m　　野外编号：4
调查时间：2017.01.06　11：30　　记录者：　　室内编号：

编号	植物名称	高度（m）	胸径（cm）	冠幅（m）	物候期	生活力	备注
1	台琼海桐	6	20	4×3	叶	2	
2	龙眼	2.5	10	3×2	叶	1	
3	龙眼	2.5	10	3×3	叶	1	
4	龙眼	3	8	3×2.5	叶	1	
5	短穗鱼尾葵	2.5	12	2.5×3	叶	1	
6	秋枫	7	23	2×3	叶	1	
7	秋枫	5	18	2×3	叶	1	
8	秋枫	5.5	16	2×2	叶	1	
9	银叶树	5	23	3×3	叶	2	
10							
11							
12							
13							
14							
15							
16							
17							
18							
19							
20							
21							
22							
23							
24							
25							
26							
27							
28							
29							
30							
31							
32							
33							
34							
35							

灌丛层植物群落调查表

群落名称：黄花梨　样方面积：5 m × 5 m　野外编号：4

调查时间：2017.01.06　11：35　记录者：　室内编号：

编号	植物名称	高度（cm）	冠径（cm）	物候期	生活力	盖度%	株数 / 从树
1	黄花梨	100	30	叶	2	15	1
2	黄花梨	90	30	叶	2	10	1
3	粗糠柴	60	25	叶	2	10	1
4	对叶榕	80	30	叶	2	15	1
5							
6	鸦胆子	50	30	叶	2	20	1
7	黄花梨	100	20	叶	2	10	1
8	倒吊笔	150	45	叶	2	10	1
9	牛筋果	300	30	叶	2	10	1
10	鹊肾树	20	10	叶	2	10	1
11							
12	簕欓花椒	150	18	叶	1	10	1
13	土坛树	200	80	叶	2	30	4
14							
15							
16							
17							
18							
19							
20							
21							
22							
23							
24							
25							
26							
27							
28							
29							
30							

草本层植物群落调查表

群落名称：斑茅-藿香蓟　样方面积 1 m × 1 m　野外编号：4

调查时间：2017.01.06　11：40　记录者：　室内编号:

编号	植物名称	株高(cm)	盖度(%)	物候期	生活力	备注
1	飞机草	30	30	花	2	
2	红毛草	40	30	花	2	
3						
4	薇甘菊	40	15	花	2	
5	斑茅	60	80	叶	1	
6						
7	十万错	15	15	花	2	
8	鬼针草	15	10	花果	2	
9	土牛膝	20	30	花	2	
10	藿香蓟	15	40	花	2	
11	黄细心	16	10	叶	2	
12						
13	一年蓬	20	30	花	2	
14	含羞草	20	30	花	2	
15						
16	假败酱	40	10	花	2	
17	鸭趾草	5	10	花	2	
18						
19						
20						
21						
22						
23						
24						
25						
26						
27						
28						
29						
30						

说明：物候期：花、叶、果

生活力：1　良好 2 一般 3 较差

总表

群落名称<br>乔-灌-草<br>优势种	木麻黄-木薯-鬼针草			野外编号(统一编号)	5
记录者		日期	2017.01.06 11：30	室内编号	
样地面积		详细地点			
GPS 定位	N：19°57.925′<br>E：110°12.883′	海拔高度	65 m		
群落高度		群落的总盖度		60%	
主要层优势种	乔木层：<br>灌木层：<br>草本层：				
群落外貌特点	次生林				
小地形及样地周围环境描述	群傍村村口旁，荒野				
分层及各层的特点	乔木层	高度			
	灌木层	高度			
	草本层	高度			
	层间植物	高度			
		高度			
备注（之前的土地利用状况）	鲜重：0.14 kg				

说明：数据尽可能填写全面，没有填无

乔木层植物群落调查表

群落名称：桉树-木麻黄　样方面积：20 m × 20 m　野外编号：5
调查时间：　记录者：　室内编号：

编号	植物名称	高度（m）	胸径（cm）	冠幅（m）	物候期	生活力	备注
1	麻疯树	8	18	3×3	叶	2	
2	鹊肾树	5	14	2×2	叶	3	
3	苦楝	10	25	5×5	叶	3	
4	苦楝	10	23	5×4	叶	3	
5	苦楝	10	23	5×5	叶	3	
6	苦楝	95	23	5×5	叶	3	
7	苦楝	9.5	25	5×4	叶	3	
8	菠萝蜜	7	19	3×3	果	2	
9	菠萝蜜	7	19	3×3	叶	2	
10	菠萝蜜	7	18	3×3	果	2	
11	菠萝蜜	7	18	3×3	果	2	
12	桉树	10	14	2.5×2.5	叶	2	
13	桉树	10.5	15	2.5×3	叶	2	
14	桉树	10.5	14	2×2	叶	2	
15	桉树	11	14	2×2	叶	2	
16	桉树	10.5	14	2×2.5	叶	2	
17	桉树	10	15	2×2.5	叶	2	
18	桉树	10	14	2×2.5	叶	2	
19	木麻黄	12	18	2×2	叶	2	
20	木麻黄	11	18	2×2	叶	2	
21	木麻黄	11	19	2×2	叶	2	
22	木麻黄	10.5	18	2×2	叶	2	
23	木麻黄	11	17	2×2	叶	2	
24	木麻黄	11	17	2×2	叶	2	
25	木麻黄	10.5	17	3×2	叶	2	
26	麻疯树	8	16	2×2	叶	2	
27	短穗鱼尾葵	6	12	2×2	叶	2	
28							
29							
30							
31							
32							
33							
34							
35							

灌丛层植物群落调查表

群落名称：麻疯树-越南悬钩子-对叶榕 样方面积：5 m × 5 m　　野外编号：5

调查时间：　　记录者：　　室内编号：

编号	植物名称	高度（cm）	冠径（cm）	物候期	生活力	盖度%	株数 / 丛树
1	潺槁木姜子	150	30	叶	3	10	1
2	麻疯树	180	120	叶	3	40	1
3							
4	越南悬钩子	240	200	叶	3	80	1
5	木薯	300	120	叶	3	60	1
6							
7	赪桐	150	60	叶	3	40	1
8	马缨丹	180	120	叶花果	3	60	1
9	粗糠柴	200	120	叶	3	70	1
10	对叶榕	180	300	叶	3	80	1
11							
12							
13							
14							
15							
16							
17							
18							
19							
20							
21							
22							
23							
24							
25							
26							
27							
28							
29							
30							

草本层植物群落调查表

群落名称：飞机草-毒瓜-鬼针草-青葙　样方面积 1 m × 1 m　　野外编号：5

调查时间：　　记录者：　　室内编号：

编号	植物名称	株高(cm)	盖度(%)	物候期	生活力	备注
1	鸡屎藤	200	10	叶	3	
2	飞机草	180	95	叶花	3	
3	海南茄	80	80	叶	3	
4	藿香蓟	120	60	叶花	3	
5						
6	叶下珠	80	20	叶果	3	
7	辣椒	80	60	叶花果	3	
8	落地生根	60	40	叶	3	
9	毒瓜	120	90	叶果	3	
10						
11	鬼针草	80	95	叶花果	3	
12	金腰箭	60	20	叶花	3	
13						
14	凤尾蕨	30	20	叶	2	
15	一年蓬	60	40	叶	3	
16	决明	80	60	叶果	3	
17						
18	青葙	80	90	叶花	3	
19	红毛草	60	40	叶花	3	
20						
21						
22						
23						
24						
25						
26						
27						
28						
29						
30						

说明：物候期：花、叶、果

生活力：1　良好 2　一般　3　较差

总表

群落名称 乔-灌-草 优势种	龙眼-九节-海芋			野外编号(统一编号)	6
记录者		日期		室内编号	
样地面积		详细地点			
GPS 定位	N：19°57.957′ E：110°13.197′	海拔高度	66 m		
群落高度		群落的总盖度	75%		
主要层优势种	乔木层：<br>灌木层：<br>草本层：				
群落外貌特点	人工林				
小地形及样地周围环境描述	果园，对面为水利工程管理处				
分层及各层的特点	乔木层	高度			
	灌木层	高度			
	草本层	高度			
	层间植物	高度			
		高度			
备注（之前的土地利用状况）	鲜重：0.1 kg				

说明：数据尽可能填写全面，没有填无

乔木层植物群落调查表

群落名称：龙眼　样方面积：20 m × 20 m　野外编号：6

调查时间：　记录者：　室内编号：

编号	植物名称	高度（m）	胸径（cm）	冠幅（m）	物候期	生活力	备注
1	龙眼	8.5	25	4×4	叶	2	
2	龙眼	8	24	4×3.5	叶	2	
3	龙眼	8	24	4×3	叶	2	
4	龙眼	8	22	4×3.5	叶	2	
5	龙眼	8.5	25	3×4	叶	2	
6	龙眼	8	23	3×4	叶	2	
7	龙眼	7.5	22	3.5×3	叶	2	
8	龙眼	7.5	27	3×4	叶	2	
9	龙眼	7	20	3×3	叶	2	
10	龙眼	7.5	24	4×4	叶	2	
11	龙眼	7	22	4×4	叶	2	
12	龙眼	7.5	23	4×4	叶	2	
13	龙眼	7.5	24	4×4	叶	2	
14	龙眼	8	23	4×4	叶	2	
15	龙眼	7.5	23	4×4	叶	2	
16	龙眼	7	22	4×4	叶	2	
17	对叶榕	4	12	2×2	叶果	2	
18	发财树	2.5	13	2×3	叶	2	
19	发财树	2.5	15	2×3	叶	2	
20	龙眼	8	24	4×4	叶	2	
21	龙眼	8	24	4×4	叶	2	
22	龙眼	7.5	23	3×4	叶	2	
23	龙眼	8	24	4×4	叶	2	
24	龙眼	7	23	4×3	叶	2	
25	龙眼	8	25	4×4	叶	2	
26							
27							
28							
29							
30							
31							
32							
33							
34							
35							

灌丛层植物群落调查表

群落名称：九节-华南省藤　样方面积：5 m × 5 m　野外编号：6
调查时间：　记录者：　室内编号：

编号	植物名称	高度（cm）	冠径（cm）	物候期	生活力	盖度%	株数/丛树
1	九节	280	150	叶果	3	80	1
2							
3	对叶榕	220	60	叶	3	40	1
4							
5	鸦胆子	120	60	叶	3	30	1
6	山小橘	160	50	叶	3	40	1
7	华南省藤	300	80	叶	3	60	1
8							
9							
10							
11							
12							
13							
14							
15							
16							
17							
18							
19							
20							
21							
22							
23							
24							
25							
26							
27							
28							
29							
30							

草本层植物群落调查表

群落名称：假蒟-海芋-凤尾蕨　样方面积 1 m × 1 m　野外编号：6
调查时间：　记录者：　室内编号：

编号	植物名称	株高(cm)	盖度(%)	物候期	生活力	备注
1	假蒟	20	90	叶果	3	
2	海芋	40	40	叶	3	
3						
4	海芋	120	90	叶果	3	
5	假蒟	15	60	叶果	3	
6						
7	凤尾蕨	70	60	叶	3	
8	海金沙	300	15	叶	3	
9						
10	海芋	180	90	叶花果	3	
11	假蒟	20	60	叶	3	
12						
13	一年蓬	60	25	叶	3	
14	鸡屎藤	230	20	叶	3	
15	火炭母	30	40	叶花果	3	
16						
17						
18						
19						
20						
21						
22						
23						
24						
25						
26						
27						
28						
29						
30						

说明：物候期：花、叶、果
生活力：1　良好 2　一般 3　较差

总表

群落名称乔-灌-草优势种	秋枫-马缨丹-荨麻			野外编号(统一编号)	7
记录者		日期	2017.01.06 12：30	室内编号	
样地面积	20×20 m	详细地点			
GPS 定位	N：19°57.715′<br>E：110°14.095′	海拔高度	56 m		
群落高度		群落的总盖度	80%		
主要层优势种	乔木层：<br>灌木层：<br>草本层：				
群落外貌特点	次生林				
小地形及样地周围环境描述	杂草丛生 乡野小路旁				
分层及各层的特点	乔木层	高度			
	灌木层	高度			
	草本层	高度			
	层间植物	高度			
		高度			
备注（之前的土地利用状况）	土壤鲜重：0.14 kg				

说明：数据尽可能填写全面，没有填无

乔木层植物群落调查表

群落名称：斜叶榕　　样方面积：20 m × 20 m　　野外编号：7
调查时间：2017.01.06　2：45　　记录者：　　室内编号：

编号	植物名称	高度（m）	胸径（cm）	冠幅（m）	物候期	生活力	备注
1	斜叶榕	8.5	28	5×6	叶	2	
2	秋枫	8	24	5×4	叶	2	
3	对叶榕	3	7	2.5×2	叶	1	
4	榕树	6.5	23	4×3	叶	2	
5							
6							
7							
8							
9							
10							
11							
12							
13							
14							
15							
16							
17							
18							
19							
20							
21							
22							
23							
24							
25							
26							
27							
28							
29							
30							
31							
32							
33							
34							
35							

灌丛层植物群落调查表

群落名称：白饭树-马樱丹　　样方面积：5 m × 5 m　　野外编号：7
调查时间：2017.01.06　12：45　　记录者：　　室内编号：

编号	植物名称	高度（cm）	冠径（cm）	物候期	生活力	盖度%	株数 / 丛树
1	狭叶山蚂蝗	230	60	叶	2	10	3
2	白饭树	250	60	叶	2	40	3
3							
4	马缨丹	80	40	花	2	30	2
5							
6	鸦胆子	40	20	叶	2	25	3
7							
8							
9							
10							
11							
12							
13							
14							
15							
16							
17							
18							
19							
20							
21							
22							
23							
24							
25							
26							
27							
28							
29							
30							

草本层植物群落调查表

群落名称：荨麻　　样方面积 1 m × 1 m　　野外编号：7
调查时间：2017.01.06　12：45　　记录者：　　室内编号:

编号	植物名称	株高(cm)	盖度(%)	物候期	生活力	备注
1	鬼针草	10	50	叶	2	
2	飞机草	8	40	叶	2	
3						
4	刺茄	5	60	叶	2	
5	荨麻	30	80	叶	2	
6						
7	海芋	30	50	叶	2	
8	含羞草	10	0	花	2	
9	赛葵	10	50	果	2	
10	银胶菊	15	30	花	2	
11	土牛膝	20	30	花	2	
12	掌叶鱼黄草	5	20	叶	2	
13						
14	金腰箭	10	20	叶	2	
15	含羞草	5	10	花	2	
16	鬼针草	10	20	花	2	
17						
18	一年蓬	15	20	叶	2	
19	刺蒴麻	35	10	叶	2	
20	紫心牵牛	10	5	叶	2	
21	灰叶	15	10	叶	2	
22	海金沙	5	30	叶	2	
23						
24						
25						
26						
27						
28						
29						
30						

说明：物候期：花、叶、果
生活力：1　良好 2　一般　3　较差

总表

群落名称<br>乔-灌-草<br>优势种	五月茶-破布叶-飞机草				野外编号<br>(统一编号)	8
记录者			日期		室内编号	8
样地面积	20×20 m		详细地点			
GPS 定位	N：19°57.827′<br>E：110°14.680′	海拔高度	46 m			
群落高度				群落的总盖度	95%	
主要层优势种	乔木层：<br>灌木层：<br>草本层：					
群落外貌特点	人工					
小地形及样地周围环境描述	苗圃					
分层及各层的特点	乔木层	高度				
	灌木层	高度				
	草本层	高度				
	层间植物	高度				
		高度				
备注（之前的土地利用状况）	鲜重 0.14 kg					

说明：数据尽可能填写全面，没有填无

乔木层植物群落调查表

群落名称：五月茶　　样方面积：20 m × 20 m　　野外编号：8

调查时间：2017.02.15　10:00　　记录者：　　室内编号：8

编号	植物名称	高度（m）	胸径（cm）	冠幅（m）	物候期	生活力	备注
1	五月茶	10	40	5×4	叶	3	
2	五月茶	9	30	5×3	叶	2	
3	五月茶	10	40	5×4	叶	3	
4	五月茶	8	30	5×5	叶	3	
5	五月茶	6	15	2×2	叶	1	
6	五月茶	10	30	5×3	叶	2	
7	五月茶	9	30	5×3	叶	2	
8	五月茶	10	40	5×4	叶	3	
9							
10	蒲葵	6	60	6×6	叶	3	
11							
12	白兰	7	15	2×3	叶	2	
13							
14	蒲葵	7	65	6×6	叶	3	
15	蒲葵	6	60	6×6	叶	3	
16							
17	白兰	12	25	3×4	叶	3	
18							
19	大花紫薇	5	15	2×1	果	1	
20	大花紫薇	6	20	3×2	果	2	
21							
22							
23							
24							
25							
26							
27							
28							
29							
30							
31							
32							
33							
34							
35							

灌丛层植物群落调查表

群落名称：簕竹-五月茶　　样方面积：5 m × 5 m　　野外编号：8
调查时间：2017.02.15　10:00　　记录者：　　室内编号：

编号	植物名称	高度（cm）	冠径（cm）	物候期	生活力	盖度%	株数/丛树
1	马缨丹	80	60	叶	3	40	
2	五月茶	40	20	叶	3	10	
3	簕竹	120	40	叶	3	60	
4							
5	五月茶	60	40	叶	3	20	
6	五月茶	40	20	叶	3	10	
7	五月茶	20	10	叶	3	5	
8							
9	海南破布叶	180	120	叶	3	60	
10	簕竹	150	40	叶	3	40	
11							
12							
13							
14							
15							
16							
17							
18							
19							
20							
21							
22							
23							
24							
25							
26							
27							
28							
29							
30							

草本层植物群落调查表

群落名称：蔓生莠竹-飞机草　　样方面积 1 m × 1 m　　野外编号：8
调查时间：2017.02.15　10:15　　记录者：　　室内编号:

编号	植物名称	株高(cm)	盖度(%)	物候期	生活力	备注
1	白花鬼针草	40	20	叶	2	
2	含羞草	20	40	叶	2	
3						
4	蔓生莠竹	40	60	叶花	2	
5						
6	飞机草	60	50	叶	3	
7						
8	火炭母	20	20	叶果	3	
9	鸡屎藤	25	20	叶	3	
10						
11	凤尾蕨	20	10	叶	3	
12	火炭母	25	20	叶	3	
13						
14						
15						
16						
17						
18						
19						
20						
21						
22						
23						
24						
25						
26						
27						
28						
29						
30						

说明：物候期：花、叶、果
生活力：1　良好 2　一般　3　较差

总表

群落名称<br>乔-灌-草<br>优势种	印度紫檀-潺槁木姜子-白茅		野外编号<br>(统一编号)	9
记录者		日期	室内编号	9
样地面积	20×20 m	详细地点		
GPS 定位	N：19°57.932′<br>E：110°15.344′	海拔高度	60m	
群落高度		群落的总盖度	90%	
主要层优势种	乔木层：<br>灌木层：<br>草本层：			
群落外貌特点	人工			
小地形及样地周围环境描述	木麻黄种植地<br>杂草众多			
分层及各层的特点	乔木层	高度		
	灌木层	高度		
	草本层	高度		
	层间植物	高度		
		高度		
备注（之前的土地利用状况）	鲜重 0.16 kg			

说明：数据尽可能填写全面，没有填无

乔木层植物群落调查表

群落名称：印度紫檀　　样方面积：20 m × 20 m　　野外编号：9
调查时间：2017.02.15　8:23　　记录者：　　室内编号：

编号	植物名称	高度（m）	胸径（cm）	冠幅（m）	物候期	生活力	备注
1	印度紫檀	5.5	15	3×3	叶	2	
2	印度紫檀	6	15	3×2	叶	1	
3	印度紫檀	2	8	1×1	叶	2	
4	印度紫檀	7	18	4×3	叶	3	
5	印度紫檀	5	15	3×3	叶	2	
6	印度紫檀	4	14	2×2	叶	1	
7	印度紫檀	6	15	3×3	叶	2	
8	印度紫檀	5	15	3×2	叶	2	
9	印度紫檀	6	10	2×2	叶	1	
10	印度紫檀	1.5	5	1×1	叶	3	
11	木麻黄	2	2	0.5 ×0.5	叶	2	
12	木麻黄	3	3	0.5 ×0.5	叶	1	
13	木麻黄	1.5	2	0.3 ×0.5	叶	2	
14	木麻黄	2	2	0.5 ×0.5	叶	2	
15	木麻黄	2.5	3	0.8 ×0.8	叶	2	
16	潺槁木姜子	3	4	3×3	叶	3	
17	潺槁木姜子	3	3	2×2	叶	2	
18	潺槁木姜子	3	4	3×3	叶	3	
19							
20							
21							
22							
23							
24							
25							
26							
27							
28							
29							
30							
31							
32							
33							
34							
35							

灌丛层植物群落调查表

群落名称：露槁木姜子　　样方面积：5 m × 5 m　　野外编号：9
调查时间：2017.02.15　8:23　　记录者：　　室内编号：

编号	植物名称	高度（cm）	冠径（cm）	物候期	生活力	盖度%	株数/丛树
1	排钱树	80	45	叶	2	20	2
2	海南菜豆树	50	20	叶	1	5	1
3							
4	大青	100	30	叶	1	10	1
5	马缨丹	156	120	叶花果	3	60	2
6							
7	潺槁木姜子	100	40	叶	2	10	2
8	潺槁木姜子	180	80	叶	3	20	1
9							
10							
11							
12							
13							
14							
15							
16							
17							
18							
19							
20							
21							
22							
23							
24							
25							
26							
27							
28							
29							
30							

草本层植物群落调查表

群落名称：白茅-鬼针草　　样方面积 1 m × 1 m　　野外编号：9
调查时间：2017.02.15　8:31　　记录者：　　室内编号:

编号	植物名称	株高(cm)	盖度(%)	物候期	生活力	备注
1	飞机草	120	40	叶果	3	
2	白茅	80	80	叶	3	
3	菜豆树	65	10	叶花果	3	
4						
5	红毛草	60	40	叶花果	3	
6	鬼针草	40	20	叶花果	3	
7	一年蓬	20	5	叶	2	
8						
9						
10	飞机草	40	5	叶	3	
11	一年蓬	20	15	叶	3	
12	鬼针草	30	60	叶花果	3	
13						
14	含羞草	10	40	叶	3	
15	蛇婆子	10	30	叶花	2	
16						
17	地旋花	20	35	叶花果	2	
18	金腰箭	15	15	叶花	2	
19	飞机草	30	5	叶	2	
20						
21						
22						
23						
24						
25						
26						
27						
28						
29						
30						

说明：物候期：花、叶、果
生活力：1　良好 2　一般　3　较差

总表

<table>
<tr><td>群落名称
乔-灌-草
优势种</td><td colspan="3">木麻黄-簕欓花椒-蔓生莠竹</td><td>野外编号
(统一编号)</td><td>10</td></tr>
<tr><td>记录者</td><td></td><td>日期</td><td></td><td>室内编号</td><td>10</td></tr>
<tr><td>样地面积</td><td>20×20 m</td><td>详细地点</td><td colspan="3"></td></tr>
<tr><td>GPS 定位</td><td>N：19°57.963′
E：110°15.850′</td><td>海拔高度</td><td colspan="3">71 m</td></tr>
<tr><td>群落高度</td><td colspan="2"></td><td>群落的总盖度</td><td colspan="2">95%</td></tr>
<tr><td>主要层优势种</td><td colspan="5">乔木层：
灌木层：
草本层：</td></tr>
<tr><td>群落外貌特点</td><td colspan="5">次生林</td></tr>
<tr><td>小地形及样地周围环境描述</td><td colspan="5">植被丰富，群落层次明显</td></tr>
<tr><td rowspan="5">分层及各层的特点</td><td>乔木层</td><td>高度</td><td colspan="3"></td></tr>
<tr><td>灌木层</td><td>高度</td><td colspan="3"></td></tr>
<tr><td>草本层</td><td>高度</td><td colspan="3"></td></tr>
<tr><td>层间植物</td><td>高度</td><td colspan="3"></td></tr>
<tr><td></td><td>高度</td><td></td><td></td><td></td></tr>
<tr><td>备注（之前的土地利用状况）</td><td colspan="5">鲜重 0.14 kg</td></tr>
</table>

说明：数据尽可能填写全面，没有填无

乔木层植物群落调查表

群落名称：木麻黄　　样方面积：20 m × 20 m　　野外编号：10

调查时间：2017.02.15　9:13　　记录者：　　室内编号：10

编号	植物名称	高度（m）	胸径（cm）	冠幅（m）	物候期	生活力	备注
1	大王榕	13	45	7×6	叶	1	
2	印度紫檀	8	15	3×4	叶	3	
3							
4	对叶榕	7	20	6×6	叶	2	
5							
6	木麻黄	15	30	4×3	叶	2	
7	木麻黄	14	25	2×4	叶	2	
8	木麻黄	10	20	3×2	叶	2	
9							
10	台湾相思	11	30	8×7	叶	2	
11	台湾相思	10	25	7×6	叶	2	
12							
13							
14							
15							
16							
17							
18							
19							
20							
21							
22							
23							
24							
25							
26							
27							
28							
29							
30							
31							
32							
33							
34							
35							

灌丛层植物群落调查表

群落名称：簕欓花椒-马樱丹-福建茶　　样方面积：5 m × 5 m　　野外编号：10
调查时间：2017.02.15　9:13　　记录者：　　室内编号：

编号	植物名称	高度（cm）	冠径（cm）	物候期	生活力	盖度%	株数 / 丛树
1	两面针	205	120	花、叶	3	30	1
2	雀梅	180	80	叶	3	10	1
3	海南破布叶	230	180	叶	3	75	1
4	越南悬钩子	40	60	叶	3	5	1
5							
6	簕欓花椒	240	180	叶花	3	80	2
7	马缨丹	150	100	叶花果	3	60	2
8	假黄皮	200	60	叶果	2	20	1
9							
10	福建茶	120	160	叶	3	60	1
11	猬实	80	40	叶果	3	20	1
12	酒饼簕	220	60	叶	3	20	1
13							
14							
15							
16							
17							
18							
19							
20							
21							
22							
23							
24							
25							
26							
27							
28							
29							
30							

草本层植物群落调查表

群落名称：蔓生莠竹-飞机草　　样方面积 1 m × 1 m　　野外编号：10
调查时间：2017.02.15　9:27　　记录者：　　室内编号：

编号	植物名称	株高(cm)	盖度(%)	物候期	生活力	备注
1	夜香牛	20	5	叶花	2	
2	大叶油草	5	40	叶	3	
3	酢浆草	5	50	叶花	3	
4						
5	糞箕笃	10	5	叶	3	
6	假杜鹃	15	20	叶花	3	
7	一点红	20	15	叶花	2	
8						
9	蔓生莠竹	20	90	叶花	3	
10	白花鬼针草	15	20	叶花	3	
11						
12	丰花草	5	2	叶花	3	
13	飞机草	60	60	叶果	3	
14						
15	含羞草	15	20	叶花	3	
16	梵天花	40	15	叶果	3	
17						
18						
19						
20						
21						
22						
23						
24						
25						
26						
27						
28						
29						
30						

说明：物候期：花、叶、果
生活力：1　良好 2　一般　3　较差

总表

群落名称<br>乔-灌-草<br>优势种	榄仁-蓖麻-斑茅			野外编号(统一编号)	11
记录者	袁浪兴	日期	2017.01.07<br>10：30	室内编号	11
样地面积	20×20 m	详细地点			
GPS 定位	N：19°57.281′<br>E：110°10.658′	海拔高度	57 m		
群落高度		群落的总盖度	80%		
主要层优势种	乔木层：<br>灌木层：<br>草本层：				
群落外貌特点	次生林				
小地形及样地周围环境描述	杂草，斑茅多，路边，倒垃圾且有一定的坟地				
分层及各层的特点	乔木层	高度			
	灌木层	高度			
	草本层	高度			
	层间植物	高度			
		高度			
备注（之前的土地利用状况）	土壤鲜重 0.14 kg				

说明：数据尽可能填写全面，没有填无

乔木层植物群落调查表

群落名称：榄仁　样方面积：20 m × 20 m　野外编号：11

调查时间：2017.01.07　10：30　记录者:袁浪兴　室内编号：11

编号	植物名称	高度（m）	胸径（cm）	冠幅（m）	物候期	生活力	备注
1	小叶榕	8	40	5×5	叶	3	
2	榄仁	12	40	8×10	叶	3	
3	龙眼	10	60	10×10	叶	3	
4	榄仁	8	40	8×10	叶	3	
5	黄槿	10	40	8×11	叶	3	
6	对叶榕	6	20	6×8	叶果	2	
7	榄仁	8	20	6×8	叶	2	
8	苦楝	8	20	6×7	叶果	1	
9	构树	4	4	2×2	叶	2	
10	苦楝	8	20	6×8	叶果	3	
11							
12							
13							
14							
15							
16							
17							
18							
19							
20							
21							
22							
23							
24							
25							
26							
27							
28							
29							
30							
31							
32							
33							
34							
35							

灌丛层植物群落调查表

群落名称：牛筋果-黄牛木　　样方面积：5 m × 5 m　　野外编号：11
调查时间：2017.01.07　10：30　　记录者：袁浪兴　　室内编号：11

编号	植物名称	高度（cm）	冠径（cm）	物候期	生活力	盖度%	株数 / 丛树
1	土坛树	260	150	叶	3	40	2
2	蓖麻	250	150	叶果	3	50	4
3							
4	牛筋果	300	200	叶果	3	80	3
5	马缨丹	100	120	叶花	3	50	2
6							
7	柞木	120	80	叶	3	20	1
8	鸦胆子	100	50	叶果	2	20	1
9	酒饼簕	80	50	叶	3	20	1
10	黄牛木	210	100	叶	3	60	1
11							
12							
13							
14							
15							
16							
17							
18							
19							
20							
21							
22							
23							
24							
25							
26							
27							
28							
29							
30							

草本层植物群落调查表

群落名称：斑茅-假败酱-南美蟛蜞菊　样方面积 1 m × 1 m　　野外编号：11
调查时间：2017.01.07　10：30　　记录者：袁浪兴　　室内编号：11

编号	植物名称	株高(cm)	盖度(%)	物候期	生活力	备注
1	白花鬼针草	50	20	叶花	3	
2	假败酱	69	60	叶花	3	
3	旱墨莲	5	5	叶花果	3	
4	飞机草	60	10	叶花果	2	
5						
6	金腰箭	60	20	叶花	3	
7	一年蓬	20	10	叶	3	
8	鸭趾草	5	30	叶花	2	
9						
10	土牛膝	20	50	叶果	2	
11	银胶菊	5	10	叶	2	
12						
13	牛筋草	5	5	叶花果	2	
14	热带铁苋菜	20	50	花果	2	
15	大尾摇	30	10	花果	3	
16						
17	含羞草	6	10	叶花	2	
18	斑茅	300	80	叶花	3	
19	南美蟛蜞菊	4	60	叶花	3	
20						
21						
22						
23						
24						
25						
26						
27						
28						
29						
30						

说明：物候期：花、叶、果
生活力：1　良好 2　一般　3　较差

总表

群落名称<br>乔-灌-草<br>优势种	凤凰木-毛柿-假蒟	野外编号<br>(统一编号)	12
记录者		日期	2017.01.07<br>13：50
室内编号			
样地面积	20×20 m	详细地点	
GPS 定位	N：19°57.156′<br>E：110°11.194′	海拔高度	56 m
群落高度		群落的总盖度	95%
主要层优势种	乔木层：<br>灌木层：<br>草本层：		
群落外貌特点	火山石众多，植被丰富		
小地形及样地周围环境描述	古村落旁边		
分层及各层的特点	乔木层	高度	
	灌木层	高度	
	草本层	高度	
	层间植物	高度	
		高度	
备注（之前的土地利用状况）	土壤鲜重：0.1 kg		

说明：数据尽可能填写全面，没有填无

乔木层植物群落调查表

群落名称：凤凰木　　样方面积：20 m × 20 m　　野外编号：12

调查时间：2017.01.07　13：50　　记录者：　　室内编号：

编号	植物名称	高度（m）	胸径（cm）	冠幅（m）	物候期	生活力	备注
1	土坛树	8	10	2×3	叶	1	
2	麻楝	8	8	3×3	叶	1	
3	荔枝	10	40	8×10	叶	1	
4	凤凰木	12	25	8×8	叶果	1	
5	凤凰木	12	10	6×8	叶果	1	
6	凤凰木	12	15	8×8	叶果	1	
7	土坛树	8	10	6×4	叶	1	
8	黄花梨	6	4	1×0.5	叶	2	
9							
10							
11							
12							
13							
14							
15							
16							
17							
18							
19							
20							
21							
22							
23							
24							
25							
26							
27							
28							
29							
30							
31							
32							
33							
34							
35							

灌丛层植物群落调查表

群落名称：毛柿　　样方面积：5 m × 5 m　　野外编号：12
调查时间：2017.01.07　14：00　　记录者：　　室内编号：

编号	植物名称	高度（cm）	冠径（cm）	物候期	生活力	盖度%	株数 / 丛树
1	毛柿	200	120	叶果	1	40	1
2	马缨丹	60	120	叶花果	1	30	1
3							
4	鸦胆子	200	60	叶	1	40	1
5	毛柿	210	180	叶果	2	70	1
6							
7	细基丸	30	60	叶	1	30	1
8	酒饼簕	50	30	叶	1	20	1
9	三角瓣花	80	80	叶	1	40	1
10							
11							
12							
13							
14							
15							
16							
17							
18							
19							
20							
21							
22							
23							
24							
25							
26							
27							
28							
29							
30							

草本层植物群落调查表

群落名称：假蒟-海南山姜　　样方面积 1 m × 1 m　　野外编号：12
调查时间：　2017.01.07　14：00　记录者：　　室内编号：

编号	植物名称	株高(cm)	盖度(%)	物候期	生活力	备注
1	假蒟	30	90	叶	1	
2	鳄嘴花	120	40	叶	1	
3						
4	麦冬	20	10	叶	1	
5	凤尾蕨	50	10	叶	2	
6	海南山姜	150	90	叶果	1	
7						
8	厚叶崖爬藤	40	80	叶	1	
9	红花青藤	150	60	叶	2	
10						
11	扭肚藤	160	40	叶果	2	
12	麒麟尾	300	60	叶	2	
13	扁担藤	400	50	叶	2	
14						
15	土牛膝	40	10	叶果	2	
16	辣椒	30	20	叶花果	3	
17	榛叶黄花稔	120	80	叶花果	1	
18	土人参	20	10	叶	2	
19	海芋	120	80	叶	1	
20						
21						
22						
23						
24						
25						
26						
27						
28						
29						
30						

说明：物候期：花、叶、果
生活力：1　良好 2　一般　3　较差

总表

群落名称<br>乔-灌-草<br>优势种	榕树-牛筋果-飞机草		野外编号(统一编号)	13
记录者		日期	2017.01.06 10：35	室内编号
样地面积	20×20 m	详细地点		
GPS 定位	N：19°57.631′<br>E：110°11.888′	海拔高度	62 m	
群落高度		群落的总盖度	90%	
主要层优势种	乔木层：<br>灌木层：<br>草本层：			
群落外貌特点	次生林			
小地形及样地周围环境描述	乡野小路旁，植被错综复杂			
分层及各层的特点	乔木层	高度		
	灌木层	高度		
	草本层	高度		
	层间植物	高度		
		高度		
备注（之前的土地利用状况）	土壤鲜重：0.10 kg			

说明：数据尽可能填写全面，没有填无

乔木层植物群落调查表

群落名称：龙眼　样方面积：20 m × 20 m　野外编号：13

调查时间：2017.01.06　10：35　记录者：　室内编号：

编号	植物名称	高度（m）	胸径（cm）	冠幅（m）	物候期	生活力	备注
1	桉树	10.5	23	8×10	叶	2	
2	麻楝	2.5	8	0.5×1.5	叶	1	
3	龙眼	2.3	8	2×2	叶	1	
4	龙眼	2.3	10	2.2×2	叶	1	
5	龙眼	2.5	10	2.5×2	叶	1	
6	龙眼	3	8	2×2	叶	1	
7	龙眼	3.2	10	2×2	叶	1	
8	潺槁木姜子	3.7	12	2×2	叶	1	
9							
10							
11							
12							
13							
14							
15							
16							
17							
18							
19							
20							
21							
22							
23							
24							
25							
26							
27							
28							
29							
30							
31							
32							
33							
34							
35							

灌丛层植物群落调查表

群落名称：牛筋果-番石榴　　样方面积：5 m × 5 m　　野外编号：13
调查时间：2017.01.06　10：35　　记录者：　　室内编号：

编号	植物名称	高度（cm）	冠径（cm）	物候期	生活力	盖度%	株数/丛树
1	潺槁木姜子	70	15	花	2	30	2
2	牛筋果	100	30	叶	2	30	2
3	毛柿	50	30	叶	2	20	3
4	鸽子豆	50	30	叶	2	20	2
5	酒饼簕	40	20	叶	2	10	1
6							
7	扭肚藤	60	30	叶	2	10	2
8	福建茶	40	25	叶	2	20	3
9	柞木	55	20	叶	2	20	1
10	假黄皮	70	25	叶	2	20	1
11	番石榴	80	30	果	2	30	2
12							
13	黄牛木	45	15	叶	2	20	1
14	鸦胆子	60	20	叶	2	10	1
15	牛筋果	20	10	叶	2	10	1
16	粗糠柴	30	10	叶	2	10	1
17	细基丸	100	45	果	2	15	1
18							
19							
20							
21							
22							
23							
24							
25							
26							
27							
28							
29							
30							

草本层植物群落调查表

群落名称：马樱丹-斑茅　　样方面积 1 m × 1 m　　野外编号：13
调查时间：2017.01.06　10：35　　记录者：　　室内编号：

编号	植物名称	株高(cm)	盖度(%)	物候期	生活力	备注
1	飞机草	20	20	叶	1	
2	心叶黄花稔	30	10	叶	2	
3	紫心牵牛	30	20	叶	1	
4	假杜鹃	50	30	叶	2	
5	马缨丹	100	35	叶花	1	
6						
7	黄花稔	20	10	花	2	
8	假败酱	25	10	花	2	
9	鬼针草	35	20	花	1	
10	含羞草	25	30	花	2	
11						
12	丰花草	20	20	花	2	
13	天门冬	15	10	叶	2	
14						
15	金腰箭	20	10	花	2	
16	斑茅	150	90	花	1	
17	飞扬草	10	5	花	2	
18						
19	藿香蓟	10	20	叶	1	
20						
21						
22						
23						
24						
25						
26						
27						
28						
29						
30						

说明：物候期：花、叶、果
生活力：1　良好 2　一般　3　较差

总表

群落名称<br>乔-灌-草<br>优势种	荔枝-两面针-吐烟花			野外编号<br>(统一编号)	14
记录者		日期		室内编号	
样地面积		详细地点			
GPS 定位	N：19°7.198′<br>E：110°12.043′	海拔高度	56 m		
群落高度		群落的总盖度	60%		
主要层优势种	乔木层：<br>灌木层：<br>草本层：				
群落外貌特点	人工林				
小地形及样地周围环境描述	果园火山石众多				
分层及各层的特点	乔木层	高度			
	灌木层	高度			
	草本层	高度			
	层间植物	高度			
		高度			
备注（之前的土地利用状况）	鲜重：0.12 kg				

说明：数据尽可能填写全面，没有填无

乔木层植物群落调查表

群落名称：荔枝-黄皮　　样方面积：20 m × 20 m　　野外编号：14
调查时间：　　记录者：　　室内编号：

编号	植物名称	高度（m）	胸径（cm）	冠幅（m）	物候期	生活力	备注
1	高山榕	11.5	100	7×6	叶	2	
2	黄皮	6	21	3×2	叶	2	
3	黄皮	6	23	2×2	叶	2	
4	黄皮	5	20	3×3	叶	2	
5	黄皮	5.5	18	2×2	叶	2	
6	黄皮	6	19	2×2	叶	2	
7	黄皮	6	24	2×2	叶	2	
8	黄皮	6	20	2×2	叶	2	
9	荔枝	9	35	6×6	叶	2	
10	荔枝	9	36	6×5.5	叶	2	
11	荔枝	7	26	5×5	叶	2	
12	荔枝	8	28	5×4	叶	2	
13	荔枝	8	28	5×4	叶	2	
14	荔枝	8	29	4×4	叶	2	
15	荔枝	7	20	3×4	叶	2	
16	荔枝	7	25	3×4	叶	2	
17	荔枝	7.5	25	4×4	叶	2	
18	荔枝	8	24	3×3	叶	2	
19	荔枝	8	25	3×3	叶	2	
20	荔枝	8	24	4×3	叶	2	
21	荔枝	7.5	25	4×3	叶	2	
22	黄皮	6.5	20	3×3	叶	2	
23	黄皮	6	19	3×2	叶	2	
24	黄皮	6	23	3×2	叶	2	
25	黄皮	6.5	22	2×2	叶	2	
26	黄皮	6	22	2×3	叶	2	
27	高山榕	7	19	3×2	叶	3	
28	铁冬青	4.5	5	1×1	叶	3	
29	苦楝	10.5	32	6×6	叶	4	
30	毛八角枫	12	34	7×6	叶	2	
31	潺槁木姜子	3	4	1.5×2	叶	3	
32	海南蒲桃	10	25	5×4	叶	3	
33							
34							
35							

灌丛层植物群落调查表

群落名称：毛柿-两面针-马樱丹-大花紫玉盘　样方面积：5 m × 5 m　野外编号：14
调查时间：　记录者：　室内编号：

编号	植物名称	高度（cm）	冠径（cm）	物候期	生活力	盖度%	株数 / 丛树
1	毛柿	804	200	叶果	3	80	1
2	越南悬钩子	180	20	叶	3	20	1
3							
4	酒饼簕	80	80	叶	3	20	1
5	华南省藤	300	120	叶果	3	20	1
6							
7	两面针	400	300	叶	3	80	1
8	海南茄	300	300	叶	3	20	1
9	牛筋果	80	60	叶	2	15	1
10	马缨丹	120	100	叶花果	3	80	1
11	大花紫玉盘	100	200	叶花	3	90	1
12							
13							
14							
15							
16							
17							
18							
19							
20							
21							
22							
23							
24							
25							
26							
27							
28							
29							
30							

草本层植物群落调查表

群落名称：飞机草-吐烟花-假蒟　样方面积 1 m × 1 m　野外编号：14
调查时间：　记录者：　室内编号：

编号	植物名称	株高(cm)	盖度(%)	物候期	生活力	备注
1	薇甘菊	200	20	叶	3	
2	飞机草	300	90	叶花	3	
3						
4	翼茎白粉藤	40	5	叶	2	
5	吐烟花	20	95	叶	3	
6	薜荔	5	20	叶	3	
7						
8	假蒟	30	80	叶	3	
9	扭肚藤	100	20	叶花	3	
10						
11	落地生根	20	60	叶花	2	
12	鸡屎藤	30	50	叶	2	
13						
14	天门冬	40	20	叶	3	
15	海芋	40	40	叶	2	
16						
17						
18						
19						
20						
21						
22						
23						
24						
25						
26						
27						
28						
29						
30						

说明：物候期：花、叶、果
生活力：1　良好 2　一般 3　较差

总表

群落名称<br>乔-灌-草<br>优势种	桉树-华南省藤-飞机草			野外编号<br>(统一编号)	15
记录者	袁浪兴	日期		室内编号	15
样地面积		详细地点			
GPS 定位	N：19°57.340′<br>E：110°12.972′	海拔高度	60 m		
群落高度			群落的总盖度	30%	
主要层优势种	乔木层：<br>灌木层：<br>草本层：				
群落外貌特点	次生林				
小地形及样地周围环境描述	荒野，小沼泽				
分层及各层的特点	乔木层	高度			
	灌木层	高度			
	草本层	高度			
	层间植物	高度			
		高度			
备注（之前的土地利用状况）	鲜重：0.12 kg				

说明：数据尽可能填写全面，没有填无

乔木层植物群落调查表

群落名称：桉树　　样方面积：20 m × 20 m　　野外编号：15
调查时间：　　记录者：　　室内编号：

编号	植物名称	高度（m）	胸径（cm）	冠幅（m）	物候期	生活力	备注
1	龙眼	7	18	2×3	叶	3	
2	龙眼	7	17	2×3	叶	3	
3	龙眼	7	16	2×2	叶	3	
4	油茶	5	15	2×2	叶	3	
5	油茶	5	15	2×2	叶	3	
6	桉树	8	8	1×0.5	叶	3	
7	桉树	8	10	0.5×1	叶	3	
8	桉树	7	8	1×1	叶	3	
9	桉树	6.5	6	0.5×0.5	叶	3	
10	桉树	7	5	0.5×0.5	叶	3	
11	桉树	8	8	1×1	叶	3	
12	桉树	6.5	6	1×1	叶	3	
13	桉树	6.5	6	0.5×1	叶	3	
14	桉树	6	7	0.5×1	叶	3	
15	桉树	6.5	7	0.5×1	叶	3	
16	桉树	6	6	1×0.5	叶	3	
17	潺槁木姜子	5.5	6	1×0.5	叶	3	
18	倒吊笔	2.5	5	0.5×0.5	果	3	
19	高山榕	13	75	7×7	叶	2	
20	斜叶榕	6	20	3×3	叶	3	
21							
22							
23							
24							
25							
26							
27							
28							
29							
30							
31							
32							
33							
34							
35							

灌丛层植物群落调查表

群落名称：华南省藤-牛筋果　　样方面积：5 m × 5 m　　野外编号：15
调查时间：　　记录者：　　室内编号：

编号	植物名称	高度（cm）	冠径（cm）	物候期	生活力	盖度%	株数/丛树
1	九里香	150	120	叶果	3	20	2
2	酒饼簕	200	180	叶	3	80	2
3							
4	毛柿	120	100	叶果	3	60	2
5	华南省藤	300	200	叶果	3	90	2
6	禾串树	120	80	叶	3	40	2
7							
8	鹊肾树	180	100	叶	3	80	2
9	黑面神	40	30	叶果	3	10	2
10	牛筋果	300	200	叶	3	90	2
11	假杜鹃	400	200	叶花	3	80	2
12	九节	80	100	叶果	3	40	2
13							
14							
15							
16							
17							
18							
19							
20							
21							
22							
23							
24							
25							
26							
27							
28							
29							
30							

草本层植物群落调查表

群落名称：飞机草-厚叶崖爬藤-球兰-斑茅　　样方面积 1 m × 1 m　　野外编号：15
调查时间：　　记录者：　　室内编号：

编号	植物名称	株高(cm)	盖度(%)	物候期	生活力	备注
1	飞机草	300	90	叶花	3	
2	毛草龙	120	30	叶果	3	
3	紫芋	80	40	叶	2	
4						
5	鸭趾草	15	40	叶	3	
6	厚叶崖爬藤	200	90	叶	3	
7						
8	球兰	300	95	叶	3	
9	凤尾蕨	30	40	叶	3	
10						
11	蛇葡萄	40	20	叶	3	
12	马交儿	60	30	叶	3	
13						
14	落地生根	60	40	叶	3	
15	薜荔	10	60	叶	3	
16	火炭母	5	40	叶果	3	
17	斑茅	400	98	叶果	3	
18						
19						
20						
21						
22						
23						
24						
25						
26						
27						
28						
29						
30						

说明：物候期：花、叶、果
生活力：1　良好 2　一般 3　较差

总表

群落名称乔-灌-草优势种	苦楝-破布叶-蔓生莠竹			野外编号(统一编号)	16
记录者		日期		室内编号	
样地面积		详细地点			
GPS定位	N：19°57.469′<br>E：110°13.409′	海拔高度	63 m		
群落高度			群落的总盖度	50%	
主要层优势种	乔木层：<br>灌木层：<br>草本层：				
群落外貌特点	人工林				
小地形及样地周围环境描述	火山口大道两侧				
分层及各层的特点	乔木层	高度			
	灌木层	高度			
	草本层	高度			
	层间植物	高度			
		高度			
备注（之前的土地利用状况）	鲜重：0.1 kg				

说明：数据尽可能填写全面，没有填无

乔木层植物群落调查表

群落名称：苦楝　　样方面积：20 m × 20 m　　野外编号：16
调查时间：　　记录者：　　室内编号：

编号	植物名称	高度（m）	胸径（cm）	冠幅（m）	物候期	生活力	备注
1	苦楝	6	23	3×4	叶	2	
2	苦楝	6	22	3×3	叶	2	
3	苦楝	6	24	3×3	叶	2	
4	洋紫荆	5	8	2×2	叶	2	
5	洋紫荆	5	10	2×2	叶	2	
6	桉树	8	25	3×3	叶	2	
7	黄葛榕	11	180	9×8	叶	1	
8	椰子	9	33	2×2	叶	2	
9	垂叶榕	7	45	4×5	叶	2	
10	苦楝	6.5	23	3×3	叶	2	
11	椰子	5.5	32	2×2	叶	2	
12	榕树	11.5	140	7×7	叶	2	
13	苦楝	5.5	20	3×3	叶	2	
14	苦楝	5.5	21	3×3	叶	2	
15	苦楝	6	23	3×3	叶	2	
16	秋枫	7.5	21	3×3.5	叶	2	
17							
18							
19							
20							
21							
22							
23							
24							
25							
26							
27							
28							
29							
30							
31							
32							
33							
34							
35							

灌丛层植物群落调查表

群落名称：马樱丹-海南破布叶-希茉莉　　样方面积：5 m × 5 m　　野外编号：16
调查时间：　　记录者：　　室内编号：

编号	植物名称	高度（cm）	冠径（cm）	物候期	生活力	盖度%	株数/丛树
1	马缨丹	80	120	叶	3	60	1
2	两面针	40	80	叶	3	15	1
3							
4	海南破布叶	180	120	叶	3	60	1
5	黄葵	80	25	叶	3	30	1
6							
7	希茉莉	60	100	叶	3	80	1
8	黄蝉	60	60	叶花	3	40	1
9							
10							
11							
12							
13							
14							
15							
16							
17							
18							
19							
20							
21							
22							
23							
24							
25							
26							
27							
28							
29							
30							

草本层植物群落调查表

群落名称：海芋-蔓生莠竹　　样方面积 1 m × 1 m　　野外编号：16
调查时间：　　记录者：　　室内编号：

编号	植物名称	株高(cm)	盖度(%)	物候期	生活力	备注
1	夜香牛	30	20	叶花	3	
2	飞机草	40	60	叶	3	
3						
4	野葛	10	60	叶	3	
5	革命菜	20	10	叶	3	
6	海芋	180	90	叶果	3	
7						
8	假败酱	40	20	叶花	3	
9	蔓生莠竹	60	90	叶花	3	
10						
11	大叶油草	10	80	叶	3	
12	墨苜蓿	5	10	叶花	3	
13						
14	一年蓬	40	10	叶	3	
15	酢浆草	5	15	叶	3	
16	丰花草	50	20	叶花	3	
17						
18	金腰箭	60	40	叶花	3	
19	凤尾蕨	20	30	叶	3	
20						
21						
22						
23						
24						
25						
26						
27						
28						
29						
30						

说明：物候期：花、叶、果
生活力：1　良好 2　一般　3　较差

总表

<table>
<tr><td>群落名称
乔-灌-草
优势种</td><td colspan="3">苦楝-麻疯树-飞机草</td><td>野外编号
(统一编
号)</td><td>17</td></tr>
<tr><td>记录者</td><td></td><td>日期</td><td></td><td>室内编号</td><td></td></tr>
<tr><td>样地面积</td><td></td><td>详细地
点</td><td colspan="3"></td></tr>
<tr><td>GPS 定位</td><td>N：19°57.230′
E：110°14.170′</td><td>海
拔
高
度</td><td colspan="3">71 m</td></tr>
<tr><td>群落高度</td><td colspan="2"></td><td>群落的总
盖度</td><td colspan="2">70%</td></tr>
<tr><td>主要层优
势种</td><td colspan="5">乔木层：
灌木层：
草本层：</td></tr>
<tr><td>群落外貌
特点</td><td colspan="5">次生林</td></tr>
<tr><td>小地形及
样地周围
环境描述</td><td colspan="5">杂草从生植物错综复杂旁边有小路</td></tr>
<tr><td rowspan="5">分层及各
层的特点</td><td>乔木层</td><td>高度</td><td colspan="3"></td></tr>
<tr><td>灌木层</td><td>高度</td><td colspan="3"></td></tr>
<tr><td>草本层</td><td>高度</td><td colspan="3"></td></tr>
<tr><td>层间植物</td><td>高度</td><td colspan="3"></td></tr>
<tr><td></td><td>高度</td><td></td><td></td><td></td></tr>
<tr><td>备注（之
前的土地
利用状
况）</td><td colspan="5">鲜重：0.16 kg</td></tr>
</table>

说明：数据尽可能填写全面，没有填无

乔木层植物群落调查表

群落名称：构树-荔枝　　样方面积：20 m × 20 m　　野外编号：17
调查时间：　　记录者：　　室内编号：

编号	植物名称	高度（m）	胸径（cm）	冠幅（m）	物候期	生活力	备注
1	苦楝	10.5	25	5×6	叶	3	
2	苦楝	7.5	20	5×5	叶	3	
3	苦楝	8	23	5×4	叶	3	
4	荔枝	6	18	4×4	叶	2	
5	荔枝	6.5	18	4×4	叶	2	
6	荔枝	8	19	5×4	叶	2	
7	荔枝	6	18	4×4	叶	2	
8	构树	7	16	3×4	叶	2	
9	构树	7.5	15	4×4	叶	2	
10	构树	5.5	18	4×4	叶	2	
11	构树	6	16	3×4	叶	2	
12	构树	5.5	15	3×3	叶	2	
13	龙眼	6	18	4×4	叶	2	
14	龙眼	5	15	4×4	叶	2	
15	木棉	10	35	5×6	叶	3	
16	苦楝	9.5	19	5×6	叶	3	
17							
18							
19							
20							
21							
22							
23							
24							
25							
26							
27							
28							
29							
30							
31							
32							
33							
34							
35							

灌丛层植物群落调查表

群落名称：麻疯树-海南破布叶　　样方面积：5 m × 5 m　　野外编号：17
调查时间：　　记录者：　　室内编号：

编号	植物名称	高度（cm）	冠径（cm）	物候期	生活力	盖度%	株数 / 丛树
1	马缨丹	200	150	叶花果	3	60	2
2	白楸	120	60	叶	2	20	2
3							
4	苎麻	180	80	叶	3	40	1
5							
6	麻疯树	250	300	叶	3	90	1
7	铁包金	180	120	叶	3	60	1
8							
9	海南破布叶	250	300	叶果	3	80	1
10	鸦胆子	200	30	叶	3	20	1
11	簕欓花椒	250	60	叶	3	40	1
12							
13							
14							
15							
16							
17							
18							
19							
20							
21							
22							
23							
24							
25							
26							
27							
28							
29							
30							

草本层植物群落调查表

群落名称：海芋-鬼针草-飞机草-薇甘菊　　样方面积 1 m × 1 m　　野外编号：17
调查时间：　　记录者：　　室内编号：

编号	植物名称	株高(cm)	盖度(%)	物候期	生活力	备注
1	木鳖子	300	70	叶果	3	
2	厚叶崖爬藤	200	40	叶	3	
3	乌蔹梅	180	40	叶	3	
4						
5	海芋	180	80	叶	3	
6	鬼针草	80	70	叶花果	3	
7	鸡屎藤	80	40	叶	3	
8						
9	刺茄	40	60	叶	3	
10	鬼针草	60	80	叶花果	3	
11						
12	野葛	20	20	叶	2	
13	飞机草	300	90	叶	2	
14						
15	薇甘菊	200	80	叶	3	
16	斑茅	300	70	叶花	3	
17						
18						
19						
20						
21						
22						
23						
24						
25						
26						
27						
28						
29						
30						

说明：物候期：花、叶、果
生活力：1　良好 2　一般　3　较差

总表

群落名称<br>乔-灌-草<br>优势种	垂叶榕-苎麻-飞机草			野外编号<br>(统一编号)	18
记录者		日期		室内编号	
样地面积	20 m × 20 m	详细地点			
GPS 定位	N：19°37.480<br>E：110°14.580	海拔高度	48 m		
群落高度			群落的总盖度	95%	
主要层优势种	乔木层：<br>灌木层：<br>草本层：				
群落外貌特点	次生林				
小地形及样地周围环境描述	植被丰富，乔木少，道路旁杂草多				
分层及各层的特点	乔木层	高度			
	灌木层	高度			
	草本层	高度			
	层间植物	高度			
		高度			
备注（之前的土地利用状况）	鲜重 0.16 kg				

说明：数据尽可能填写全面，没有填无

乔木层植物群落调查表

群落名称：垂叶榕　　样方面积：20 m × 20 m　　野外编号：18

调查时间：2017.02.15　10：28　　记录者：　　室内编号：18

编号	植物名称	高度（m）	胸径（cm）	冠幅（m）	物候期	生活力	备注
1	苦楝	7	20	2×3	叶	1	
2							
3	对叶榕	7	15	4×5	叶果	2	
4							
5	鱼尾葵	4	10	2×2	叶	2	
6							
7	垂叶榕	8	20	4×5	叶	2	
8							
9							
10							
11							
12							
13							
14							
15							
16							
17							
18							
19							
20							
21							
22							
23							
24							
25							
26							
27							
28							
29							
30							
31							
32							
33							
34							
35							

灌丛层植物群落调查表

群落名称：苎麻-鹊肾树　　样方面积：5 m × 5 m　　野外编号：18
调查时间：2017.02.15　10：28　　记录者：　　室内编号：

编号	植物名称	高度（cm）	冠径（cm）	物候期	生活力	盖度%	株数 / 丛树
1	鹊肾树	190	120	叶	3	40	1
2	鹊肾树	80	120	叶	3	60	1
3							
4	苎麻	350	360	叶	3	90	1
5							
6	大花紫玉盘	20	80	叶	3	20	1
7	猪肚木	120	120	叶	3	40	1
8	猬实	200	140	叶	3	40	1
9							
10	两面针	120	80	叶	3	20	1
11	菜豆树	200	180	叶花	2	40	1
12							
13							
14							
15							
16							
17							
18							
19							
20							
21							
22							
23							
24							
25							
26							
27							
28							
29							
30							

草本层植物群落调查表

群落名称：飞机草　　样方面积 1 m × 1 m　　野外编号：18
调查时间：2017.02.14　10：37　　记录者：　　室内编号：

编号	植物名称	株高(cm)	盖度(%)	物候期	生活力	备注
1	金腰箭	40	30	叶花	2	
2	土牛膝	20	10	叶花	2	
3						
4	飞机草	80	90	叶	3	
5						
6	白花鬼针草	60	40	叶花	2	
7						
8	斑茅	180	40	叶花	3	
9	乌蔹莓	40	20	叶	3	
10						
11	飞机草	80	90	叶花	3	
12						
13						
14						
15						
16						
17						
18						
19						
20						
21						
22						
23						
24						
25						
26						
27						
28						
29						
30						

说明：物候期：花、叶、果
生活力：1　良好 2　一般 3　较差

总表

群落名称<br>乔-灌-草<br>优势种	苦楝-白饭树-斑茅		野外编号(统一编号)	19
记录者		日期	2017.02.23<br>8：46	室内编号	
样地面积	20×20 m	详细地点		
GPS 定位	N：19°57.308′<br>E：110°15.039′	海拔高度	54 m	
群落高度		群落的总盖度	90%	
主要层优势种	乔木层：<br>灌木层：<br>草本层：			
群落外貌特点	乔木少，斑茅多，杂草多，次生。			
小地形及样地周围环境描述	水库旁			
分层及各层的特点	乔木层	高度		
	灌木层	高度		
	草本层	高度		
	层间植物	高度		
		高度		
备注（之前的土地利用状况）	0.14 kg			

说明：数据尽可能填写全面，没有填无

乔木层植物群落调查表

群落名称：苦楝　　样方面积：20 m × 20 m　　野外编号：19

调查时间：2017.02.23　8：50　　记录者：　　室内编号：

编号	植物名称	高度（m）	胸径（cm）	冠幅（m）	物候期	生活力	备注
1	苦楝	8	8	5×6	叶	1	
2							
3							
4							
5							
6							
7							
8							
9							
10							
11							
12							
13							
14							
15							
16							
17							
18							
19							
20							
21							
22							
23							
24							
25							
26							
27							
28							
29							
30							
31							
32							
33							
34							
35							

灌丛层植物群落调查表

群落名称：山榕　　　样方面积：5 m × 5 m　　　野外编号：19
调查时间：　　　记录者：　　　室内编号：

编号	植物名称	高度（cm）	冠径（cm）	物候期	生活力	盖度%	株数/丛树
1	马缨丹	180	150	花	1	70	2
2	白饭树	400	200	叶	2	70	1
3							
4	钝叶紫金牛	200	150	叶花果	1	60	1
5	山榕	200	200	叶	2	80	1
6							
7	两面针	170	80	叶	2	60	1
8							
9							
10							
11							
12							
13							
14							
15							
16							
17							
18							
19							
20							
21							
22							
23							
24							
25							
26							
27							
28							
29							
30							

草本层植物群落调查表

群落名称：斑茅-飞机草　　　样方面积 1 m × 1 m　　　野外编号：19
调查时间：　　　记录者：　　　室内编号：

编号	植物名称	株高(cm)	盖度(%)	物候期	生活力	备注
1	斑茅	500	80	叶花果	3	
2	飞机草	160	80	叶果	2	
3						
4	一年蓬	20	15	叶	1	
5	酢浆草	3	10	叶	1	
6	含羞草	40	60	叶花	1	
7						
8	掌叶山猪菜	5	5	叶	2	
9	金腰箭	30	10	叶	2	
10	络石	2	40	叶	2	
11						
12	白花鬼针草	20	30	叶花	1	
13	卤蕨	120	5	叶	2	
14	丰花草	15	15	叶花	1	
15	黄花稔	20	5	叶花	2	
16						
17	蛇葡萄	170	5	叶	2	
18	刺茄	60	50	叶	1	
19	倒地铃	200	40	叶花果	1	
20	假败酱	30	20	叶花	1	
21						
22						
23						
24						
25						
26						
27						
28						
29						
30						

说明：物候期：花、叶、果
生活力：1　良好 2　一般　3　较差

总表

群落名称<br>乔-灌-草<br>优势种	桉树-黄牛木-薇甘菊		野外编号<br>(统一编号)	20
记录者		日期	室内编号	20
样地面积	20×20 m	详细地点		
GPS 定位	N：19°57.265′<br>E：110°15.620′	海拔高度	55 m	
群落高度		群落的总盖度	60%	
主要层优势种	乔木层：<br>灌木层：<br>草本层：			
群落外貌特点	人工林			
小地形及样地周围环境描述	荔枝苗人工种植<br>桉树林			
分层及各层的特点	乔木层	高度		
	灌木层	高度		
	草本层	高度		
	层间植物	高度		
		高度		
备注（之前的土地利用状况）	鲜重 0.12 kg			

说明：数据尽可能填写全面，没有填无

乔木层植物群落调查表

群落名称：桉树　　样方面积：20 m × 20 m　　野外编号：20

调查时间：2017.02.15　11：56　　记录者：　　室内编号：20

编号	植物名称	高度（m）	胸径（cm）	冠幅（m）	物候期	生活力	备注
1	大叶榄仁	10	30	10×10	休眠	1	
2	大叶榄仁	11	30	10×10	休眠	1	
3							
4	荔枝	1	3	0.5×0.5	叶	3	
5							
6	桉树	25	40	4×4	叶	3	
7	桉树	15	30	3×3	叶	3	
8	桉树	25	40	4×4	叶	3	
9	桉树	20	35	3×5	叶	2	
10							
11							
12							
13							
14							
15							
16							
17							
18							
19							
20							
21							
22							
23							
24							
25							
26							
27							
28							
29							
30							
31							
32							
33							
34							
35							

灌丛层植物群落调查表

群落名称：阔苞菊-黄牛木　　样方面积：5 m × 5 m　　野外编号：20
调查时间：2017.02.15　11：56　　记录者：　　室内编号：

编号	植物名称	高度（cm）	冠径（cm）	物候期	生活力	盖度%	株数 / 丛树
1	猬实	40	20	叶	3	12	1
2	阔苞菊	60	80	叶	3	20	1
3							
4	酒饼簕	40	30	叶	3	40	1
5	潺槁木姜子	40	30	叶	2	20	1
6							
7	黄牛木	80	60	叶	2	40	1
8	黑面神	60	40	叶	2	10	1
9							
10							
11							
12							
13							
14							
15							
16							
17							
18							
19							
20							
21							
22							
23							
24							
25							
26							
27							
28							
29							
30							

草本层植物群落调查表

群落名称：薇甘菊　　样方面积 1 m × 1 m　　野外编号：20
调查时间：2017.02.15　12：05　　记录者：　　室内编号：

编号	植物名称	株高(cm)	盖度(%)	物候期	生活力	备注
1	少花龙葵	15	20	叶花	3	
2						
3	含羞草	15	20	叶果	2	
4						
5	夜香牛	20	25	叶花	2	
6						
7	一年蓬	20	20	叶	3	
8						
9	藿香蓟	10	15	叶	3	
10	墨苜蓿	5	40	叶	3	
11						
12	一点红	30	20	叶花果	3	
13	墨苜蓿	12	40	叶花	3	
14						
15	飞机草	30	10	叶	3	
16	蛇葡萄	40	20	叶	3	
17	薇甘菊	20	80	叶花	3	
18						
19						
20						
21						
22						
23						
24						
25						
26						
27						
28						
29						
30						

说明：物候期：花、叶、果
生活力：1　良好 2　一般　3　较差

总表

群落名称<br>乔-灌-草<br>优势种	秋枫-鹊肾树-鬼针草			野外编号<br>(统一编号)	21
记录者	袁浪兴	日期	2017.01.07 11：10	室内编号	21
样地面积	20×20 m	详细地点			
GPS 定位	N：19°56.707′<br>E：110°10.569′	海拔高度	61 m		
群落高度		群落的总盖度	95%		
主要层优势种	乔木层：<br>灌木层：<br>草本层：				
群落外貌特点	荒废的农田				
小地形及样地周围环境描述	乔木少，藤本和杂草丛生，地势平坦，土壤层较厚。				
分层及各层的特点	乔木层	高度			
	灌木层	高度			
	草本层	高度			
	层间植物	高度			
		高度			
备注（之前的土地利用状况）	原有农田，现荒废<br>土壤鲜重：0.1 kg				

说明：数据尽可能填写全面，没有填无

乔木层植物群落调查表

群落名称：对叶榕　样方面积：20 m × 20 m　野外编号：21

调查时间：2017.01.07　11：10　记录者：袁浪兴　室内编号：21

编号	植物名称	高度（m）	胸径（cm）	冠幅（m）	物候期	生活力	备注
1	秋枫	6	10	3×3	叶	1	
2	对叶榕	4	5	2×2	叶果	1	
3	对叶榕	5	6	2×3	叶果	1	
4							
5							
6							
7							
8							
9							
10							
11							
12							
13							
14							
15							
16							
17							
18							
19							
20							
21							
22							
23							
24							
25							
26							
27							
28							
29							
30							
31							
32							
33							
34							
35							

灌丛层植物群落调查表

群落名称： 两面针　　样方面积：5 m × 5 m　　野外编号：21
调查时间：2017.01.07　11：10　　记录者：　袁浪兴　　室内编号：21

编号	植物名称	高度（cm）	冠径（cm）	物候期	生活力	盖度%	株数 / 丛树
1	两面针	160	110	叶	3	60	2
2	鹊肾树	200	120	叶	3	50	1
3							
4	酒饼簕	60	40	叶	2	20	1
5	五色梅	30	80	叶花	3	50	1
6							
7	青灰叶下珠	50	30	叶	3	50	3
8	大青	50	15	叶	2	10	1
9							
10							
11							
12							
13							
14							
15							
16							
17							
18							
19							
20							
21							
22							
23							
24							
25							
26							
27							
28							
29							
30							

草本层植物群落调查表

群落名称：蛇葡萄-白花鬼针草-斑茅　　样方面积 1 m × 1 m　　野外编号：21
调查时间：2017.01.07　11：10　　记录者：　袁浪兴　　室内编号：　21

编号	植物名称	株高(cm)	盖度(%)	物候期	生活力	备注
1	火炭母	200	80	叶花果	3	
2	蛇葡萄	300	90	叶果	3	
3						
4	白花鬼针草	60	90	叶花果	3	
5	决明	50	50	叶花果	2	
6						
7	紫金牵牛	220	80	叶花	2	
8	毛草龙	50	15	叶花	2	
9	斑茅	320	94	叶花	3	
10						
11	金腰箭	40	60	叶花	2	
12	夜香牛	20	23	叶花	2	
13	野甘草	18	20	叶花	1	
14						
15	藿香蓟	30	40	叶花	2	
16	土牛膝	40	54	花果	2	
17	叶下珠	24	13	叶花	2	
18						
19						
20						
21						
22						
23						
24						
25						
26						
27						
28						
29						
30						

说明：物候期：花、叶、果
生活力：1　良好 2　一般　3　较差

总表

<table>
<tr><td>群落名称
乔-灌-草
优势种</td><td colspan="4">苦楝-麻疯树-蔓生莠竹</td><td>野外编号(统一编号)</td><td colspan="2">22</td></tr>
<tr><td>记录者</td><td colspan="2"></td><td>日期</td><td>2017.01.07
13：10</td><td>室内编号</td><td colspan="2">22</td></tr>
<tr><td>样地面积</td><td colspan="2">20×20 m</td><td>详细地点</td><td colspan="4"></td></tr>
<tr><td>GPS 定位</td><td>N：19°56.789′
E：110°11.230′</td><td>海拔高度</td><td colspan="5">89 m</td></tr>
<tr><td>群落高度</td><td colspan="3"></td><td>群落的总盖度</td><td colspan="3">99%</td></tr>
<tr><td>主要层优势种</td><td colspan="7">乔木层：
灌木层：
草本层：</td></tr>
<tr><td>群落外貌特点</td><td colspan="7">次生林，杂草丛生</td></tr>
<tr><td>小地形及样地周围环境描述</td><td colspan="7">公路旁</td></tr>
<tr><td rowspan="5">分层及各层的特点</td><td>乔木层</td><td>高度</td><td colspan="5"></td></tr>
<tr><td>灌木层</td><td>高度</td><td colspan="5"></td></tr>
<tr><td>草本层</td><td>高度</td><td colspan="5"></td></tr>
<tr><td>层间植物</td><td>高度</td><td colspan="5"></td></tr>
<tr><td></td><td>高度</td><td colspan="2"></td><td></td><td colspan="2"></td></tr>
<tr><td>备注（之前的土地利用状况）</td><td colspan="7">土壤鲜重：0.12 kg</td></tr>
</table>

说明：数据尽可能填写全面，没有填无

乔木层植物群落调查表

群落名称：苦楝　样方面积：20 m × 20 m　野外编号：22

调查时间：2017.01.7　13：10　记录者：　室内编号：

编号	植物名称	高度（m）	胸径（cm）	冠幅（m）	物候期	生活力	备注
1	苦楝	8	20	6×4	果	2	
2	苦楝	6	18	2×6	叶果	2	
3	秋枫	6	10	6× 4	叶	1	
4	龙眼	8	20	8×6	叶	1	
5	毛八角枫	6	10	4× 3	叶	2	
6							
7							
8							
9							
10							
11							
12							
13							
14							
15							
16							
17							
18							
19							
20							
21							
22							
23							
24							
25							
26							
27							
28							
29							
30							
31							
32							
33							
34							
35							

灌丛层植物群落调查表

群落名称：麻疯树-马樱丹　　样方面积：5 m × 5 m　　野外编号：22

调查时间：2017.01.07　13：10　　记录者：　　室内编号：

编号	植物名称	高度（cm）	冠径（cm）	物候期	生活力	盖度%	株数/丛树
1	木豆	220	90	叶花	1	50	1
2	黄牛木	240	100	叶	2	20	1
3							
4	麻疯树	300	400	叶	2	80	1
5	假杜鹃	180	60	叶花	2	40	1
6	土坛树	180	40	叶	2	10	1
7	鸦胆子	80	40	叶果	2	20	1
8							
9	柞木	160	20	花	2	10	1
10	假鹰爪	150	80	花果	1	60	1
11	马缨丹	120	100	叶花果	1	80	1
12	华南省藤	300	200	叶果	1	40	1
13							
14							
15							
16							
17							
18							
19							
20							
21							
22							
23							
24							
25							
26							
27							
28							
29							
30							

草本层植物群落调查表

群落名称：蔓生莠竹-飞机草　　样方面积 1 m × 1 m　　野外编号：22

调查时间：2017.01.07　13：10　　记录者：　　室内编号：

编号	植物名称	株高(cm)	盖度(%)	物候期	生活力	备注
1	蔓生莠竹	80	90	叶花	1	
2	飞机草	60	60	叶花	1	
3						
4	鬼针草	40	40	叶花果	1	
5	鸭趾草	40	30	叶花	1	
6						
7	斑茅	270	40	叶花	1	
8	蔓生莠竹	60	20	叶花	1	
9						
10	金腰箭	40	20	叶花	2	
11	心叶黄花稔	60	20	叶花	1	
12	广防风	100	40	花果	1	
13						
14	假蒟	30	40	叶	2	
15	百花丹	80	20	叶花	2	
16	土牛膝	60	10	叶果	1	
17	薜荔	5	50	叶花	1	
18						
19						
20						
21						
22						
23						
24						
25						
26						
27						
28						
29						
30						

说明：物候期：花、叶、果

生活力：1　良好 2　一般 3　较差

总表

群落名称 乔-灌-草 优势种	荔枝-山小橘-吐烟花		野外编号 (统一编号)	23
记录者		日期	2017.01.07 14：29	室内编号	
样地面积	20×20 m	详细地点		
GPS 定位	N：19°57.033′ E：110°11.506′	海拔高度	60 m	
群落高度		群落的总盖度	95%	
主要层优势种	乔木层：<br>灌木层：<br>草本层：			
群落外貌特点	荔枝林，人工林			
小地形及样地周围环境描述	火山石多，地表覆盖吐烟花			
分层及各层的特点	乔木层	高度		
	灌木层	高度		
	草本层	高度		
	层间植物	高度		
		高度		
备注（之前的土地利用状况）	土壤鲜重：0.12 kg			

说明：数据尽可能填写全面，没有填无

乔木层植物群落调查表

群落名称：荔枝　　样方面积：20 m × 20 m　　野外编号：23

调查时间：2017.01.07　14：29　　记录者：　　室内编号：

编号	植物名称	高度（m）	胸径（cm）	冠幅（m）	物候期	生活力	备注
1	荔枝	8	60	12×12	叶	1	
2	荔枝	6	30	8×10	叶	1	
3	荔枝	8	40	6×10	叶	1	
4	荔枝	6	30	10×8	叶	1	
5	荔枝	8	40	10×10	叶	1	
6	毛八角枫	10	20	6×5	叶	1	
7	香港樫木	6	6	3×2	叶	1	
8	香港樫木	6	4	2×1	叶	1	
9							
10							
11							
12							
13							
14							
15							
16							
17							
18							
19							
20							
21							
22							
23							
24							
25							
26							
27							
28							
29							
30							
31							
32							
33							
34							
35							

灌丛层植物群落调查表

群落名称：毛柿-马樱丹-山小橘　　样方面积：5 m × 5 m　　野外编号：23
调查时间：2017.01.07　14：29　　记录者：　　室内编号：

编号	植物名称	高度（cm）	冠径（cm）	物候期	生活力	盖度%	株数/丛树
1	细基丸	80	20	叶	1	15	1
2	九节	40	10	叶	1	5	1
3							
4	酒饼簕	40	40	叶果	1	30	1
5	马缨丹	60	60	叶花	1	40	1
6							
7	毛柿	120	100	叶果	1	60	1
8	山小橘	160	80	叶	1	40	1
9	粗糠柴	150	80	叶	1	20	1
10							
11							
12							
13							
14							
15							
16							
17							
18							
19							
20							
21							
22							
23							
24							
25							
26							
27							
28							
29							
30							

草本层植物群落调查表

群落名称：吐烟花-斑茅　　样方面积 1 m × 1 m　　野外编号：23
调查时间：2017.01.07　14：40　　记录者：　　室内编号：

编号	植物名称	株高(cm)	盖度(%)	物候期	生活力	备注
1	假蒟	20	60	叶	1	
2	金腰箭	30	30	叶花	1	
3	吐烟花	5	90	叶	1	
4						
5	飞机草	10	50	叶花	1	
6						
7	厚叶崖爬藤	40	60	叶	1	
8	紫心牵牛	5	30	叶花	1	
9						
10	含羞草	10	20	叶花	1	
11	黄花稔	40	40	叶花	1	
12						
13	斑茅	360	95	叶花	1	
14						
15						
16						
17						
18						
19						
20						
21						
22						
23						
24						
25						
26						
27						
28						
29						
30						

说明：物候期：花、叶、果
生活力：1　良好 2　一般　3　较差

总表

群落名称 乔-灌-草 优势种	荔枝-幌伞枫-假蒟			野外编号 (统一编号)	24
记录者		日期		室内编号	
样地面积		详细地点			
GPS 定位	N：19°56.956′<br>E：110°12.068′	海拔高度	77 m		
群落高度			群落的总盖度	50	
主要层优势种	乔木层：<br>灌木层：<br>草本层：				
群落外貌特点	人工林				
小地形及样地周围环境描述	果菜园				
分层及各层的特点	乔木层	高度			
	灌木层	高度			
	草本层	高度			
	层间植物	高度			
		高度			
备注（之前的土地利用状况）	鲜重：0.14 kg				

说明：数据尽可能填写全面，没有填无

乔木层植物群落调查表

群落名称：菠萝蜜-黄皮　样方面积：20 m × 20 m　野外编号：24

调查时间：　记录者：　室内编号：

编号	植物名称	高度（m）	胸径（cm）	冠幅（m）	物候期	生活力	备注
1	土坛树	4.5	5	1.5×1	叶	2	
2	麻楝	5.5	14	1.5×1.5	叶	2	
3	海南蒲桃	12	75	8×9	叶	2	
4	柚子	5.5	13	2×3	叶	2	
5	荔枝	9	48	8×9	叶	2	
6	荔枝	9.5	50	8×8	叶	2	
7	荔枝	10.5	85	7.5×8	叶	2	
8	黄皮	6	18	3×3	叶	2	
9	黄皮	6.5	15	3×4	叶	2	
10	黄皮	6.5	16	3×3	叶	2	
11	黄皮	6	15	2.5×3	叶	2	
12	黄皮	6	16	2.5×3	叶	2	
13	黄皮	6	15	2×3	叶	2	
14	菠萝蜜	8.5	30	5×6	果	2	
15	菠萝蜜	8	32	5×5	果	2	
16	菠萝蜜	8	32	5×6	果	2	
17	菠萝蜜	8	28	5×4	叶	2	
18	菠萝蜜	8	30	5×5	叶	2	
19	菠萝蜜	7.5	34	5×5	果	2	
20	菠萝蜜	8	30	5×4	叶	2	
21	菠萝蜜	8	28	5×5	果	2	
22	苦楝	7	19	4×5	叶	2	
23	荔枝	5.5	16	3×3	叶	3	
24	木豆	4	15	2×2	叶	3	
25	木豆	4	14	2×2	叶	3	
26							
27							
28							
29							
30							
31							
32							
33							
34							
35							

灌丛层植物群落调查表

群落名称：苎麻　　样方面积：5 m × 5 m　　野外编号：24
调查时间：　　记录者：　　室内编号：

编号	植物名称	高度（cm）	冠径（cm）	物候期	生活力	盖度%	株数 / 丛树
1	倒吊笔	80	60	叶	2	10	1
2	萝芙木	150	60	叶果	3	20	1
3							
4	酒饼簕	80	60	叶	3	40	1
5	苎麻	90	100	叶	3	40	1
6							
7	山小橘	80	40	叶	3	10	1
8	幌伞枫	150	40	叶	3	40	1
9	鹊肾树	80	40	叶	3	15	1
10	毛柿	40	40	叶	3	10	1
11							
12							
13							
14							
15							
16							
17							
18							
19							
20							
21							
22							
23							
24							
25							
26							
27							
28							
29							
30							

草本层植物群落调查表

群落名称：薇甘菊-柊叶　　样方面积 1 m × 1 m　　野外编号：24
调查时间：　　记录者：　　室内编号：

编号	植物名称	株高(cm)	盖度(%)	物候期	生活力	备注
1	薇甘菊	300	90	叶花	3	
2	鸭趾草	30	10	叶花	3	
3	假蒟	30	80	花果	3	
4						
5	火炭母	40	60	叶花果	3	
6	金腰箭	70	40	叶花	3	
7						
8	海芋	80	85	叶	3	
9	柊叶	60	90	叶	3	
10						
11	眼树莲	1000	80	叶	3	
12	吐烟花	75	80	叶	3	
13	翼茎白粉藤	20	15	叶	3	
14						
15	菠萝	60	40	叶	3	
16	红瓜	300	60	叶果	3	
17	麦冬	20	40	叶	3	
18						
19						
20						
21						
22						
23						
24						
25						
26						
27						
28						
29						
30						

说明：物候期：花、叶、果
生活力：1　良好 2　一般　3　较差
