# Supplementary material for: Correction: Taxonomic and phylogenetic diversity of vascular plants at Ma'anling volcano urban park in tropical Haikou, China: Reponses to soil properties
Source: PLoS One. 2018 Dec 14;13(12):e0209520. doi: 10.1371/journal.pone.0209520 (PMC6294358; doi:10.1371/journal.pone.0209520)
Supplement: S4 File — (PDF) [file pone.0209520.s002.pdf]

总表

<table>
<tr><td>群落名称
乔-灌-草
优势种</td><td colspan="4">构树-蓖麻-飞机草</td><td>野外编号
(统一编
号)</td><td colspan="2">25</td></tr>
<tr><td>记录者</td><td colspan="2"></td><td>日期</td><td></td><td>室内编号</td><td colspan="2"></td></tr>
<tr><td>样地面积</td><td colspan="2"></td><td>详细地点</td><td colspan="4"></td></tr>
<tr><td>GPS 定位</td><td>N：19°56.882′
E：110°12.755′</td><td>海拔高度</td><td colspan="5">69 m</td></tr>
<tr><td>群落高度</td><td colspan="3"></td><td>群落的总盖度</td><td colspan="3">10%</td></tr>
<tr><td>主要层优势种</td><td colspan="7">乔木层：
灌木层：
草本层：</td></tr>
<tr><td>群落外貌特点</td><td colspan="7">次生林</td></tr>
<tr><td>小地形及样地周围环境描述</td><td colspan="7">荒野</td></tr>
<tr><td rowspan="5">分层及各层的特点</td><td>乔木层</td><td>高度</td><td colspan="5"></td></tr>
<tr><td>灌木层</td><td>高度</td><td colspan="5"></td></tr>
<tr><td>草本层</td><td>高度</td><td colspan="5"></td></tr>
<tr><td>层间植物</td><td>高度</td><td colspan="5"></td></tr>
<tr><td></td><td>高度</td><td colspan="2"></td><td></td><td colspan="2"></td></tr>
<tr><td>备注（之前的土地利用状况）</td><td colspan="7">鲜重：0.16 kg</td></tr>
</table>

说明：数据尽可能填写全面，没有填无

乔木层植物群落调查表

群落名称：番木瓜　样方面积：20 m × 20 m　野外编号：25
调查时间：　记录者：　室内编号：

编号	植物名称	高度（m）	胸径（cm）	冠幅（m）	物候期	生活力	备注
1	番木瓜	2.5	12	1×1.5	果	3	
2	番木瓜	2.3	10	1×1	叶	3	
3	番木瓜	2.5	8	1×1	果	3	
4	番木瓜	2.5	10	1×1	叶	3	
5	番木瓜	2	10	1×1	叶	3	
6	番木瓜	2	8	1×1	叶	3	
7	番木瓜	2	8	1×1	果	3	
8	番木瓜	2.3	6	0.5×0.5	果	3	
9	番木瓜	2.2	5	0.5×1	叶	3	
10	番木瓜	2.5	6	0.5×0.8	叶	3	
11	对叶榕	3	9	3×2	叶	3	
12	构树	5	13	3×4	叶	2	
13	构树	5	12	3×3	叶	2	
14	构树	5	14	3×3	叶	2	
15							
16							
17							
18							
19							
20							
21							
22							
23							
24							
25							
26							
27							
28							
29							
30							
31							
32							
33							
34							
35							

灌丛层植物群落调查表

群落名称：两面针-蓖麻　　样方面积：5 m × 5 m　　野外编号：25
调查时间：　　记录者：　　室内编号：

编号	植物名称	高度（cm）	冠径（cm）	物候期	生活力	盖度%	株数 / 丛树
1	蓖麻	280	180	叶果	3	50	4
2	鸦胆子	180	30	叶	3	20	3
3	梵天花	80	60	叶果	2	20	2
4							
5	两面针	80	120	叶	3	80	2
6	白饭树	40	120	叶	3	40	3
7	马缨丹	140	120	叶花	3	60	2
8							
9	大青	100	100	叶果	3	40	1
10	大花紫玉盘	120	160	叶果	3	50	1
11							
12							
13							
14							
15							
16							
17							
18							
19							
20							
21							
22							
23							
24							
25							
26							
27							
28							
29							
30							

草本层植物群落调查表

群落名称：飞机草-鬼针草-野葛　　样方面积 1 m × 1 m　　野外编号：25
调查时间：　　记录者：　　室内编号：

编号	植物名称	株高(cm)	盖度(%)	物候期	生活力	备注
1	飞机草	180	95	叶花	3	
2	南美蟛蜞菊	40	20	叶	2	
3						
4	斑茅	310	50	叶花	3	
5	鬼针草	120	95	叶花	3	
6	鸭趾草	20	40	叶	3	
7						
8	野葛	20	90	叶	2	
9	金腰箭	25	40	叶花	3	
10						
11	藿香蓟	15	5	叶花	3	
12	含羞草	10	40	叶花	3	
13						
14	鸡屎藤	10	40	叶	3	
15	叶下珠	20	40	叶果	3	
16	海芋	40	60	叶果	3	
17						
18						
19						
20						
21						
22						
23						
24						
25						
26						
27						
28						
29						
30						

说明：物候期：花、叶、果
生活力：1　良好 2　一般　3　较差

总表

群落名称<br>乔-灌-草<br>优势种	桉树-马缨丹-蔓生莠竹			野外编号<br>(统一编号)	26
记录者		日期		室内编号	
样地面积		详细地点			
GPS 定位	N：19°56.923′<br>E：11°13.357′	海拔高度	80 m		
群落高度		群落的总盖度	65%		
主要层优势种	乔木层：<br>灌木层：<br>草本层：				
群落外貌特点	人工林				
小地形及样地周围环境描述	桉树林，靠近省道				
分层及各层的特点	乔木层	高度			
	灌木层	高度			
	草本层	高度			
	层间植物	高度			
		高度			
备注（之前的土地利用状况）	鲜重：0.12 kg				

说明：数据尽可能填写全面，没有填无

乔木层植物群落调查表

群落名称：桉树　　样方面积：20 m × 20 m　　野外编号：26
调查时间：　　记录者：　　室内编号：

编号	植物名称	高度（m）	胸径（cm）	冠幅（m）	物候期	生活力	备注
1	假苹婆	4.5	16	3×2	叶花	2	
2	假苹婆	4	14	2×2	叶	2	
3	桉树	10	15	1×1.5	叶	3	
4	桉树	10	16	1.5×1	叶	3	
5	桉树	10	16	1.5×1	叶	3	
6	桉树	10	16	1.5×1	叶	3	
7	桉树	10	16	1.5×1	叶	3	
8	桉树	9.5	15	1×1	叶	3	
9	桉树	9.5	15	1×1	叶	3	
10	桉树	9	15	1×1	叶	3	
11	桉树	10	13	1×1	叶	3	
12	桉树	4.5	13	1.5×1	叶	3	
13	桉树	9	15	1.5×1	叶	3	
14	桉树	9	13	1×1.5	叶	3	
15	桉树	9	8	1×1.5	叶	3	
16	桉树	10	8	1×1	叶	3	
17	桉树	10	8	1×1	叶	3	
18	桉树	9	9	1×1	叶	3	
19	桉树	9.5	10	1×1	叶	3	
20	桉树	8.5	13	1×1	叶	3	
21	桉树	8	12	1×1	叶	3	
22	桉树	8	12	1×1	叶	3	
23	桉树	8.5	12	1×1	叶	3	
24	桉树	9	13	1×1	叶	3	
25	桉树	9	9	1×1	叶	3	
26	桉树	10	10	1×1	叶	3	
27	桉树	10	10	1×1	叶	3	
28	桉树	9	10	1×1	叶	3	
29	桉树	9	8	1×1	叶	3	
30	桉树	9	9	1×1	叶	3	
31							
32							
33							
34							
35							

灌丛层植物群落调查表

群落名称：马樱丹-两面针　　样方面积：5 m × 5 m　　野外编号：26
调查时间：　　记录者：　　室内编号：

编号	植物名称	高度（cm）	冠径（cm）	物候期	生活力	盖度%	株数/丛树
1	马缨丹	180	120	叶花果	3	80	1
2	白楸	120	80	叶	3	40	1
3							
4	两面针	80	150	叶	3	90	1
5	潺槁木姜子	200	50	叶	3	20	1
6							
7	苎麻	300	150	叶	3	80	1
8	马缨丹	200	140	叶花果	3	90	1
9							
10							
11							
12							
13							
14							
15							
16							
17							
18							
19							
20							
21							
22							
23							
24							
25							
26							
27							
28							
29							
30							

草本层植物群落调查表

群落名称：紫心牵牛-蔓生莠竹　　样方面积 1 m × 1 m　　野外编号：26
调查时间：　　记录者：　　室内编号：

编号	植物名称	株高(cm)	盖度(%)	物候期	生活力	备注
1	鬼针草	80	80	叶花果	3	
2	紫心牵牛	200	90	叶	3	
3						
4	斑鸠菊	200	80	叶花	3	
5	土牛膝	80	40	叶花果	2	
6						
7	飞机草	120	80	叶花	3	
8	金腰箭	40	30	叶花	2	
9						
10	斑茅	250	80	叶花	3	
11						
12	野葛	250	60	叶	3	
13	丰花草	40	20	叶花	3	
14	蔓生莠竹	80	90	叶花	3	
15	黄花稔	80	40	叶花果	3	
16						
17						
18						
19						
20						
21						
22						
23						
24						
25						
26						
27						
28						
29						
30						

说明：物候期：花、叶、果
生活力：1　良好 2　一般　3　较差

总表

群落名称<br>乔-灌-草<br>优势种	龙眼-山椒子-吐烟花		野外编号<br>(统一编号)	27
记录者		日期		室内编号
样地面积		详细地点		
GPS 定位	N：19°57.041′<br>E：110°14.109′	海拔高度	79 m	
群落高度		群落的总盖度	80%	
主要层优势种	乔木层：<br>灌木层：<br>草本层：			
群落外貌特点	次生林			
小地形及样地周围环境描述	乡道附近			
分层及各层的特点	乔木层	高度		
	灌木层	高度		
	草本层	高度		
	层间植物	高度		
		高度		
备注（之前的土地利用状况）	鲜重：0.12 kg			

说明：数据尽可能填写全面，没有填无

乔木层植物群落调查表

群落名称：龙眼　　样方面积：20 m × 20 m　　野外编号：27
调查时间：　　记录者：　　室内编号：

编号	植物名称	高度（m）	胸径（cm）	冠幅（m）	物候期	生活力	备注
1	龙眼	7	17	4×4	叶	2	
2	龙眼	7	16	4×4	叶	2	
3	龙眼	7	16	4×4	叶	2	
4	龙眼	7	15	4×4	叶	2	
5	龙眼	7	17	4×4	叶	2	
6	龙眼	7	16	4×3	叶	2	
7	菠萝蜜	8	18	4×4	果	2	
8	菠萝蜜	7	20	4×4	果	2	
9	菠萝蜜	7.5	18	4×5	果	2	
10	菠萝蜜	7.5	19	4×4	果	3	
11	苦楝	8	23	5×5	叶	3	
12	苦楝	9	25	5×6	叶	3	
13	苦楝	8.5	23	5×5	叶	3	
14	苦楝	8.5	24	5×5	叶	3	
15	麻楝	7.5	15	3×3	叶	2	
16	香蕉	4	9	4×3	叶	2	
17	香蕉	4.5	8	3×4	叶	2	
18	香蕉	4	8	3×3	叶	2	
19	龙眼	6.5	15	3×3	叶	2	
20	龙眼	7	16	4×4	叶	2	
21	龙眼	7	16	4×4	叶	2	
22	土坛树	5	5	2×2	叶	3	
23	倒吊笔	6.5	13	2×3	果	3	
24	麻疯树	6.5	13	2×3	果	3	
25	麻疯树	5.5	12	2×3	叶	3	
26							
27							
28							
29							
30							
31							
32							
33							
34							
35							

灌丛层植物群落调查表

群落名称：大花紫玉盘-斑鸠菊-海南茄-假杜鹃　样方面积：5 m × 5 m　野外编号：27
调查时间：　记录者：　室内编号：

编号	植物名称	高度（cm）	冠径（cm）	物候期	生活力	盖度%	株数 / 丛树
1	大花紫玉盘	400	300	叶花果	3	80	3
2	白楸	150	80	叶	3	40	2
3	斑鸠菊	300	200	叶	3	80	3
4							
5	两面针	80	30	叶	3	20	3
6	马缨丹	200	180	叶花果	3	60	3
7	海南茄	80	100	叶花果	3	80	2
8							
9	假杜鹃	60	80	叶花	3	60	1
10	苎麻	120	40	叶	3	80	4
11	鸦胆子	80	200	叶	2	20	1
12							
13							
14							
15							
16							
17							
18							
19							
20							
21							
22							
23							
24							
25							
26							
27							
28							
29							
30							

草本层植物群落调查表

群落名称：鬼针草-厚叶崖爬藤-假蒟-吐烟花　样方面积 1 m × 1 m　野外编号：27
调查时间：　记录者：　室内编号：

编号	植物名称	株高(cm)	盖度(%)	物候期	生活力	备注
1	金腰箭	60	20	叶花	3	
2	鬼针草	80	80	叶花果	3	
3	飞机草	120	40	叶花	3	
4						
5	鸭趾草	10	40	叶	2	
6	夜香牛	30	10	叶花	2	
7						
8	黄花稔	40	20	叶花	2	
9	含羞草	20	30	叶花	3	
10						
11	火炭母	40	60	叶花果	3	
12	土牛膝	40	10	叶花果	2	
13	厚叶崖爬藤	300	80	叶	3	
14						
15	假蒟	40	80	叶果	2	
16	凤尾蕨	30	20	叶	2	
17	吐烟花	20	80	叶	3	
18						
19						
20						
21						
22						
23						
24						
25						
26						
27						
28						
29						
30						

说明：物候期：花、叶、果
生活力：1　良好 2　一般　3　较差

总表

群落名称乔-灌-草优势种	山楝-土坛树-飞机草		野外编号(统一编号)	28
记录者		日期	室内编号	28
样地面积	20 m × 20 m	详细地点		
GPS 定位	N：19°57.048′<br>E：110°14.674′	海拔高度	55m	
群落高度		群落的总盖度		
主要层优势种	乔木层：<br>灌木层：<br>草本层：			
群落外貌特点	次生林			
小地形及样地周围环境描述	石头工厂周边，火山石丛中，建筑垃圾，杂草丛生			
分层及各层的特点	乔木层	高度		
	灌木层	高度		
	草本层	高度		
	层间植物	高度		
		高度		
备注（之前的土地利用状况）	鲜重 0.12 kg			

说明：数据尽可能填写全面，没有填无

乔木层植物群落调查表

群落名称：山楝-麻疯树　　样方面积：20 m × 20 m　　野外编号：28

调查时间：2017.02.15　10：58　　记录者：　　室内编号：28

编号	植物名称	高度（m）	胸径（cm）	冠幅（m）	物候期	生活力	备注
1	山楝	7	15	4×4	叶	3	
2	山楝	5	10	4×3	叶	2	
3	麻风树	7	25	4×3	叶	2	
4	麻风树	5	15	2×3	叶	2	
5							
6							
7							
8							
9							
10							
11							
12							
13							
14							
15							
16							
17							
18							
19							
20							
21							
22							
23							
24							
25							
26							
27							
28							
29							
30							
31							
32							
33							
34							
35							

灌丛层植物群落调查表

群落名称：苎麻-猬实　　样方面积：5 m × 5 m　　野外编号：28
调查时间：2017.02.15　10：58　　记录者：　　室内编号：

编号	植物名称	高度（cm）	冠径（cm）	物候期	生活力	盖度%	株数 / 丛树
1	土坛树	240	180	叶	2	60	1
2	猬实	230	190	叶	2	80	1
3	牛筋果	300	40	叶	3	20	1
4							
5	苎麻	280	180	叶	3	90	1
6							
7	苎麻	180	40	叶	3	40	1
8	马缨丹	120	80	叶花	3	60	1
9							
10							
11							
12							
13							
14							
15							
16							
17							
18							
19							
20							
21							
22							
23							
24							
25							
26							
27							
28							
29							
30							

草本层植物群落调查表

群落名称：蔓生莠竹-飞机草-斑茅　　样方面积 1 m × 1 m　　野外编号：28
调查时间：2017.02.15　11：00　　记录者：　　室内编号：

编号	植物名称	株高(cm)	盖度(%)	物候期	生活力	备注
1	蔓生莠竹	120	90	叶	3	
2						
3	飞机草	150	80	叶花	3	
4						
5	斑茅	300	80	叶花	3	
6						
7	乌蔹莓	20	30	叶	3	
8	飞机草	40	60	叶	3	
9						
10	野葛	30	20	叶	3	
11	南美蟛蜞菊	10	10	叶	2	
12						
13						
14						
15						
16						
17						
18						
19						
20						
21						
22						
23						
24						
25						
26						
27						
28						
29						
30						

说明：物候期：花、叶、果
生活力：1　良好 2 一般 3 较差

总表

群落名称乔-灌-草优势种	苦楝-鹊肾树-飞机草			野外编号(统一编号)	29
记录者		日期		室内编号	29
样地面积	20×20 m	详细地点			
GPS 定位	N：19°56.963′<br>E：110°15.236′	海拔高度	54 m		
群落高度		群落的总盖度	90%		
主要层优势种	乔木层：<br>灌木层：<br>草本层：				
群落外貌特点	次生林				
小地形及样地周围环境描述	杂草多，道路边，植被丰富				
分层及各层的特点	乔木层	高度			
	灌木层	高度			
	草本层	高度			
	层间植物	高度			
		高度			
备注（之前的土地利用状况）	鲜重 0.14 kg				

说明：数据尽可能填写全面，没有填无

乔木层植物群落调查表

群落名称：苦楝-秋枫　　样方面积：20 m × 20 m　　野外编号：29
调查时间：2017.02.15　11：20　　记录者：　　室内编号：29

编号	植物名称	高度（m）	胸径（cm）	冠幅（m）	物候期	生活力	备注
1	秋枫	12	40	5×5	叶	2	
2	秋枫	10	30	5×4	叶	2	
3	秋枫	4	12	3×2	叶	2	
4	秋枫	12	40	5×3	叶	2	
5							
6	苦楝	14	30	3×5	休眠	1	
7	苦楝	10	20	3×3	休眠	1	
8	木槿	8	20	4×4	叶	2	
9							
10	苦楝	10	20	5×4	休眠	1	
11	苦楝	12	30	5×5	叶	2	
12	苦楝	7	15	4×2	休眠	1	
13	苦楝	13	30	5×5	叶	2	
14							
15							
16							
17							
18							
19							
20							
21							
22							
23							
24							
25							
26							
27							
28							
29							
30							
31							
32							
33							
34							
35							

灌丛层植物群落调查表

群落名称：鹊肾树　　样方面积：5 m × 5 m　　野外编号：29
调查时间：2017.02.15　11：20　　记录者：　　室内编号：

编号	植物名称	高度（cm）	冠径（cm）	物候期	生活力	盖度%	株数 / 丛树
1	马缨丹	150	100	叶花果	3	40	1
2	海南破布叶	60	40	叶	3	20	1
3							
4	水茄	120	80	叶	2	40	1
5							
6	鹊肾树	240	150	叶	3	80	1
7							
8	猬实	200	80	叶	2	40	1
9							
10	猪肚木	80	40	叶	2	10	1
11							
12							
13							
14							
15							
16							
17							
18							
19							
20							
21							
22							
23							
24							
25							
26							
27							
28							
29							
30							

草本层植物群落调查表

群落名称：淡竹叶-飞机草　　样方面积 1 m × 1 m　　野外编号：29
调查时间：2017.02.15　11：26　　记录者：　　室内编号：

编号	植物名称	株高(cm)	盖度(%)	物候期	生活力	备注
1	夜香牛	20	5	叶花	3	
2	丰花草	15	20	叶花	3	
3						
4	刺茄	20	20	叶	3	
5	斑茅	80	40	叶	3	
6						
7	海芋	20	20	叶	3	
8	禾本科淡竹叶	20	80	叶花	3	
9						
10	鸡屎藤	40	15	叶	3	
11	飞机草	120	80	叶花	3	
12						
13	假蒟	20	60	叶	3	
14	白花鬼针草	40	50	叶花果	3	
15						
16						
17						
18						
19						
20						
21						
22						
23						
24						
25						
26						
27						
28						
29						
30						

说明：物候期：花、叶、果
生活力：1　良好 2　一般　3　较差

总表

群落名称<br>乔-灌-草<br>优势种	苦楝-两面针-地毯草			野外编号<br>(统一编号)	30
记录者		日期		室内编号	30
样地面积	20×20 m	详细地点			
GPS 定位	N：19°56.940′<br>E：110°15.607′	海拔高度	51 m		
群落高度		群落的总盖度	80%		
主要层优势种	乔木层：<br>灌木层：<br>草本层：				
群落外貌特点	次生林				
小地形及样地周围环境描述	道路旁，杂草多，乔木少				
分层及各层的特点	乔木层	高度			
	灌木层	高度			
	草本层	高度			
	层间植物	高度			
		高度			
备注（之前的土地利用状况）	鲜重 0.14 kg				

说明：数据尽可能填写全面，没有填无

乔木层植物群落调查表

群落名称：苦楝-番石榴　　样方面积：20 m × 20 m　　野外编号：30

调查时间：2017.02.15　12：12　　记录者：　　室内编号：30

编号	植物名称	高度（m）	胸径（cm）	冠幅（m）	物候期	生活力	备注
1	番石榴	3	7	2×2	叶	3	
2							
3	苦楝	4	5	1×1	休眠	1	
4							
5							
6							
7							
8							
9							
10							
11							
12							
13							
14							
15							
16							
17							
18							
19							
20							
21							
22							
23							
24							
25							
26							
27							
28							
29							
30							
31							
32							
33							
34							
35							

灌丛层植物群落调查表

群落名称：两面针　　样方面积：5 m × 5 m　　野外编号：30
调查时间：2017.02.15　12：12　　记录者：　　室内编号：

编号	植物名称	高度（cm）	冠径（cm）	物候期	生活力	盖度%	株数/丛树
1	水茄	150	120	花果	3	40	
2	三角梅	80	100	叶花	3	40	
3							
4	大青	80	20	叶	2	10	
5	马缨丹	40	40	叶花	3	20	
6	黑面神	20	10	叶	3	2	
7							
8	两面针	150	120	叶花	3	60	
9	雀梅	80	80	叶	3	20	
10	铁包金	30	15	叶	3	3	
11	酒饼簕	60	20	叶	3	5	
12	黑面神	100	40	叶	3	20	
13							
14							
15							
16							
17							
18							
19							
20							
21							
22							
23							
24							
25							
26							
27							
28							
29							
30							

草本层植物群落调查表

群落名称：蟛蜞菊-含羞草-大叶油草　　样方面积 1 m × 1 m　　野外编号：30
调查时间：　　记录者：　　室内编号：

编号	植物名称	株高(cm)	盖度(%)	物候期	生活力	备注
1	蟛蜞菊	5	90	叶	3	
2	白茅	15	60	叶花	3	
3						
4	含羞草	20	90	叶果	3	
5	夜香牛	10	5	叶花	3	
6						
7	假败酱	20	15	叶花	3	
8	平花草	15	40	叶花	3	
9						
10	大叶油草	5	90	叶	3	
11	一年蓬	15	10	叶	3	
12						
13	酢浆草	5	80	叶花	3	
14	文殊兰	80	60	叶花	3	
15	飞机草	40	20	叶	3	
16						
17						
18						
19						
20						
21						
22						
23						
24						
25						
26						
27						
28						
29						
30						

说明：物候期：花、叶、果
生活力：1　良好 2　一般　3　较差

总表

<table>
<tr><td>群落名称
乔-灌-草
优势种</td><td colspan="4">荔枝-鹊肾树-鬼针草</td><td>野外编号(统一编号)</td><td>31</td></tr>
<tr><td>记录者</td><td colspan="2"></td><td>日期</td><td>2017.01.07
11：30</td><td>室内编号</td><td></td></tr>
<tr><td>样地面积</td><td colspan="2">20×20 m</td><td>详细地点</td><td colspan="3"></td></tr>
<tr><td>GPS 定位</td><td>N：19°56.408′
E：110°10.605′</td><td>海拔高度</td><td colspan="4">63 m</td></tr>
<tr><td>群落高度</td><td colspan="3"></td><td>群落的总盖度</td><td colspan="2">80%</td></tr>
<tr><td>主要层优势种</td><td colspan="6">乔木层：
灌木层：
草本层：</td></tr>
<tr><td>群落外貌特点</td><td colspan="6">荔枝园</td></tr>
<tr><td>小地形及样地周围环境描述</td><td colspan="6">果园，荔枝番石榴等植物</td></tr>
<tr><td rowspan="5">分层及各层的特点</td><td>乔木层</td><td>高度</td><td colspan="4"></td></tr>
<tr><td>灌木层</td><td>高度</td><td colspan="4"></td></tr>
<tr><td>草本层</td><td>高度</td><td colspan="4"></td></tr>
<tr><td>层间植物</td><td>高度</td><td colspan="4"></td></tr>
<tr><td></td><td>高度</td><td colspan="2"></td><td></td><td></td></tr>
<tr><td>备注（之前的土地利用状况）</td><td colspan="6">土壤鲜重：0.14 kg</td></tr>
</table>

说明：数据尽可能填写全面，没有填无

乔木层植物群落调查表

群落名称：荔枝　　样方面积：20 m × 20 m　　野外编号：31

调查时间：2017.01.07　11：30　　记录者：　　室内编号：

编号	植物名称	高度（m）	胸径（cm）	冠幅（m）	物候期	生活力	备注
1	荔枝	6	15	6×8	叶	1	
2	龙眼	5	10	5×6	叶	1	
3	荔枝	5	15	8×8	叶	2	
4	荔枝	6	12	6×5	叶	1	
5	黄花梨	5	6	1×2	叶	1	
6	黄花梨	5	4	2×2	叶	2	
7	番石榴	4	6	3×2	叶	2	
8	苦楝	6	10	5×3	叶果	2	
9	苦楝	8	8	3×4	叶果	2	
10	荔枝	6	10	6×8	叶	1	
11	荔枝	6	12	8×7	叶	1	
12							
13							
14							
15							
16							
17							
18							
19							
20							
21							
22							
23							
24							
25							
26							
27							
28							
29							
30							
31							
32							
33							
34							
35							

灌丛层植物群落调查表

群落名称：鹊肾树-光荚含羞草　　样方面积：5 m × 5 m　　野外编号：31
调查时间：　2017.01.07　11：30　　记录者：　　室内编号：

编号	植物名称	高度（cm）	冠径（cm）	物候期	生活力	盖度%	株数 / 丛树
1	鹊肾树	200	120	叶	2	40	1
2	倒吊笔	160	60	叶	3	20	1
3	铁包金	120	120	叶花	2	60	1
4							
5	鹊肾树	300	100	叶	1	80	1
6	鹊肾树	150	100	叶	2	40	1
7	破布叶	200	150	叶果	2	50	1
8							
9	福建茶	150	100	叶	2	40	1
10	山小橘	160	200	叶花果	1	50	1
11	牛筋果	160	150	叶果	1	60	1
12	光荚含羞草	400	300	叶	1	80	1
13							
14							
15							
16							
17							
18							
19							
20							
21							
22							
23							
24							
25							
26							
27							
28							
29							
30							

草本层植物群落调查表

群落名称：鬼针草-三点金　　样方面积 1 m × 1 m　　野外编号：31
调查时间：2017.01.07　11：30　　记录者：　　室内编号：

编号	植物名称	株高(cm)	盖度(%)	物候期	生活力	备注
1	倒地铃	120	80	果	1	
2	鬼针草	80	40	花果	1	
3						
4	红瓜	300	60	花果	1	
5	毒瓜	10	40	花果	1	
6	鸭趾草	5	30	花	2	
7						
8	斑茅	310	80	叶花	1	
9	香蕉	340	70	叶果	2	
10						
11	鬼针草	40	90	叶花果	1	
12	链荚豆	5	20	叶果	2	
13						
14	飞扬草	15	5	叶花	2	
15	三点金	5	90	叶	1	
16	飞机草	50	80	叶花	1	
17						
18						
19						
20						
21						
22						
23						
24						
25						
26						
27						
28						
29						
30						

说明：物候期：花、叶、果
生活力：1　良好 2 一般 3 较差

总表

群落名称<br>乔-灌-草<br>优势种	荔枝-毛柿-飞机草			野外编号(统一编号)	32
记录者		日期	2017.01.07<br>12：30	室内编号	
样地面积	20×20 m	详细地点			
GPS 定位	N：19°56.420′<br>E：110°11.081′	海拔高度	69 m		
群落高度		群落的总盖度	80%		
主要层优势种	乔木层：<br>灌木层：<br>草本层：				
群落外貌特点	荔枝林，多斑茅				
小地形及样地周围环境描述	地表火山石多				
分层及各层的特点	乔木层	高度			
	灌木层	高度			
	草本层	高度			
	层间植物	高度			
		高度			
备注（之前的土地利用状况）	土壤鲜重：0.14 kg				

说明：数据尽可能填写全面，没有填无

乔木层植物群落调查表

群落名称：荔枝　　样方面积：20 m × 20 m　　野外编号：32

调查时间：2017.01.07　12：30　　记录者：　　室内编号：

编号	植物名称	高度（m）	胸径（cm）	冠幅（m）	物候期	生活力	备注
1	荔枝	8	60	8×6	叶	1	
2	荔枝	8	60	5×6	叶	1	
3	荔枝	9	40	6×5	叶	1	
4	荔枝	6	50	6×7	叶	1	
5	荔枝	7	50	7×8	叶	1	
6	荔枝	8	40	8×6	叶	1	
7	荔枝	8	40	7×5	叶	1	
8	荔枝	9	60	8×7	叶	1	
9							
10							
11							
12							
13							
14							
15							
16							
17							
18							
19							
20							
21							
22							
23							
24							
25							
26							
27							
28							
29							
30							
31							
32							
33							
34							
35							

灌丛层植物群落调查表

群落名称：假杜鹃-破布叶　　样方面积：5 m × 5 m　　野外编号：32

调查时间：2017.01.07　12：30　　记录者：　　室内编号：

编号	植物名称	高度（cm）	冠径（cm）	物候期	生活力	盖度%	株数 / 丛树
1	山小橘	70	20	叶	1	10	1
2	酒饼簕	80	15	叶	1	10	1
3	鸦胆子	50	10	叶	2	5	1
4	假杜鹃	180	100	叶花	1	60	1
5	裸花紫珠	150	150	叶	1	50	1
6	毛柿	80	60	叶果	2	40	1
7							
8	福建茶	160	80	叶	1	40	1
9	毛柿	180	40	叶果	1	30	1
10	两面针	160	80	叶	1	20	1
11							
12	破布叶	120	100	叶果	2	60	1
13	马缨丹	120	60	叶	2	40	1
14	倒吊笔	160	20	叶	1	20	1
15							
16							
17							
18							
19							
20							
21							
22							
23							
24							
25							
26							
27							
28							
29							
30							

草本层植物群落调查表

群落名称：斑茅-飞机草　　样方面积 1 m × 1 m　　野外编号：32

调查时间：2017.01.07　12：40　记录者：　　室内编号：

编号	植物名称	株高(cm)	盖度(%)	物候期	生活力	备注
1	飞机草	60	80	叶花	1	
2	少花龙葵	40	20	叶花	1	
3	一点红	20	5	叶花	1	
4						
5	革命菜	15	10	叶花	2	
6	鸡屎藤	5	40	叶	2	
7	酢浆草	15	5	叶	2	
8	落地生根	20	15	叶	2	
9						
10	毒瓜	50	60	叶花果	1	
11	藿香蓟	60	50	叶花	2	
12	厚叶崖爬藤	10	60	叶果	1	
13						
14	金腰箭	30	15	叶花	1	
15	土牛膝	35	40	叶果	1	
16						
17	斑茅	300	90	叶花	1	
18	鸭趾草	5	30	叶花	1	
19	苦蘵	10	40	叶花果	1	
20						
21						
22						
23						
24						
25						
26						
27						
28						
29						
30						

说明：物候期：花、叶、果

生活力：1　良好 2　一般　3　较差

总表

群落名称乔-灌-草优势种	荔枝-破布叶-吐烟花		野外编号(统一编号)	33
记录者		日期		室内编号
样地面积	20×20 m	详细地点		
GPS 定位	N：19°56.173′<br>E：110°11.643′	海拔高度	98 m	
群落高度		群落的总盖度	95%	
主要层优势种	乔木层：<br>灌木层：<br>草本层：			
群落外貌特点	火山石多，地表灌木多，分层明显			
小地形及样地周围环境描述	道路边，房边有果园			
分层及各层的特点	乔木层	高度		
	灌木层	高度		
	草本层	高度		
	层间植物	高度		
		高度		
备注（之前的土地利用状况）	0.12 kg			

说明：数据尽可能填写全面，没有填无

乔木层植物群落调查表

群落名称：荔枝　　样方面积：20 m × 20 m　　野外编号：33
调查时间：　　记录者：　　室内编号：

编号	植物名称	高度（m）	胸径（cm）	冠幅（m）	物候期	生活力	备注
1	高山榕	10	180	1210	叶	1	
2	木麻黄	12	20	46	叶	1	
3	荔枝	10	25	87	叶	1	
4	荔枝	12	35	108	叶	1	
5	荔枝	10	20	68	叶	1	
6	土坛树	6	15	65	叶	2	
7	八角枫	10	20	65	休眠	2	
8							
9							
10							
11							
12							
13							
14							
15							
16							
17							
18							
19							
20							
21							
22							
23							
24							
25							
26							
27							
28							
29							
30							
31							
32							
33							
34							
35							

灌丛层植物群落调查表

群落名称：九节-毛柿-破布叶　　样方面积：5 m × 5 m　　野外编号：33
调查时间：　　记录者：　　室内编号：

编号	植物名称	高度（cm）	冠径（cm）	物候期	生活力	盖度%	株数/丛树
1	毛柿	180	50	叶	1	70	2
2	酒饼簕	100	20	叶	2	10	1
3							
4	鸦胆子	120	70	叶	2	20	1
5	九节	200	100	叶果	1	80	2
6	假鹰爪	120	70	叶	2	60	2
7							
8	破布叶	300	120	叶	1	70	2
9	马缨丹	300	40	叶	2	20	1
10							
11							
12							
13							
14							
15							
16							
17							
18							
19							
20							
21							
22							
23							
24							
25							
26							
27							
28							
29							
30							

草本层植物群落调查表

群落名称：吐烟花-飞机草　　样方面积 1 m × 1 m　　野外编号：33
调查时间：　　记录者：　　室内编号：

编号	植物名称	株高(cm)	盖度(%)	物候期	生活力	备注
1	薇甘菊	40	10	叶	1	
2	飞机草	120	60	叶	1	
3						
4	金腰箭	30	5	叶花	2	
5	淡竹叶	20	5	叶	2	
6	麦冬	30	10	叶	1	
7						
8	翼茎白粉藤	100	10	叶	2	
9	扭肚藤	70	40	叶	1	
10						
11	吐烟花	3	60	叶	2	
12	肾蕨	20	10	叶	2	
13	铁线蕨	20	5	叶	2	
14						
15	吐烟花	5	70	叶	1	
16	厚叶崖爬藤	120	20	叶	2	
17						
18						
19						
20						
21						
22						
23						
24						
25						
26						
27						
28						
29						
30						

说明：物候期：花、叶、果
生活力：1　良好 2　一般　3　较差

总表

<table>
<tr><td>群落名称
乔-灌-草
优势种</td><td colspan="4">木麻黄-马缨丹-海芋</td><td>野外编号
(统一编号)</td><td>34</td></tr>
<tr><td>记录者</td><td colspan="2"></td><td>日期</td><td></td><td>室内编号</td><td></td></tr>
<tr><td>样地面积</td><td colspan="2">20×20 m</td><td>详细地点</td><td colspan="3"></td></tr>
<tr><td>GPS 定位</td><td>N：19°56.313′
E：110°12.177′</td><td>海拔高度</td><td colspan="4">113 m</td></tr>
<tr><td>群落高度</td><td colspan="3"></td><td>群落的总盖度</td><td colspan="2">90%</td></tr>
<tr><td>主要层优势种</td><td colspan="6">乔木层：
灌木层：
草本层：</td></tr>
<tr><td>群落外貌特点</td><td colspan="6">杂草众多、土壤湿润，乔木少</td></tr>
<tr><td>小地形及样地周围环境描述</td><td colspan="6">村房、道路边</td></tr>
<tr><td rowspan="5">分层及各层的特点</td><td>乔木层</td><td>高度</td><td colspan="4"></td></tr>
<tr><td>灌木层</td><td>高度</td><td colspan="4"></td></tr>
<tr><td>草本层</td><td>高度</td><td colspan="4"></td></tr>
<tr><td>层间植物</td><td>高度</td><td colspan="4"></td></tr>
<tr><td></td><td>高度</td><td colspan="2"></td><td></td><td></td></tr>
<tr><td>备注（之前的土地利用状况）</td><td colspan="6">0.12 kg</td></tr>
</table>

说明：数据尽可能填写全面，没有填无

乔木层植物群落调查表

群落名称：荔枝-木麻黄　　样方面积：20 m × 20 m　　野外编号：34
调查时间：　　记录者：　　室内编号：

编号	植物名称	高度（m）	胸径（cm）	冠幅（m）	物候期	生活力	备注
1	木麻黄	18	15	2×2	叶	1	
2	苦楝	12	20	4×3	叶	1	
3	土坛树	15	25	2×3	叶	1	
4	芒果	15	35	3×2	叶	1	
5	荔枝	12	60	4×6	叶	1	
6	荔枝	10	40	3×3	叶	1	
7	木麻黄	15	15	2×2	叶	1	
8	秋枫	10	18	2×2	叶	1	
9							
10							
11							
12							
13							
14							
15							
16							
17							
18							
19							
20							
21							
22							
23							
24							
25							
26							
27							
28							
29							
30							
31							
32							
33							
34							
35							

灌丛层植物群落调查表

群落名称：马樱丹-倒吊笔-对叶榕-潺槁木姜子 样方面积：5 m × 5 m 野外编号：34
调查时间： 记录者： 室内编号：

编号	植物名称	高度（cm）	冠径（cm）	物候期	生活力	盖度%	株数/丛树
1	白饭树	170	30	叶	1	20	1
2	大青	120	30	叶	2	10	1
3							
4	马缨丹	140	60	叶花	1	40	2
5	菜豆树	110	70	叶	2	20	1
6	倒吊笔	180	30	叶	2	40	1
7							
8	对叶榕	170	40	叶	2	40	1
9	潺槁木姜子	150	40	叶	2	40	2
10	麻风树	120	80	叶	2	20	1
11							
12							
13							
14							
15							
16							
17							
18							
19							
20							
21							
22							
23							
24							
25							
26							
27							
28							
29							
30							

草本层植物群落调查表

群落名称：竹节草-斑茅-海芋-紫芋 样方面积 1 m × 1 m 野外编号：34
调查时间： 记录者： 室内编号：

编号	植物名称	株高(cm)	盖度(%)	物候期	生活力	备注
1	竹节草	100	80	叶花	2	
2	斑茅	300	80	叶花果	3	
3						
4	飞机草	120	70	叶果	2	
5	刺茄	80	20	叶	2	
6						
7	粪箕笃	50	10	叶	2	
8	刺茄	60	60	叶	2	
9						
10	假蒟	40	70	叶	1	
11	麒麟尾	100	60	叶	2	
12	海芋	140	80	叶	1	
13						
14	金腰箭	150	50	叶	2	
15	紫芋	120	80	叶	1	
16	落葵	170	5	叶花果	1	
17						
18						
19						
20						
21						
22						
23						
24						
25						
26						
27						
28						
29						
30						

说明：物候期：花、叶、果
生活力：1 良好 2 一般 3 较差

总表

群落名称乔-灌-草优势种	秋枫-木豆-海芋			野外编号(统一编号)	35
记录者		日期		室内编号	
样地面积	20×20 m	详细地点			
GPS 定位	N：19°56.188′<br>E：110°12.900′	海拔高度	120 m		
群落高度		群落的总盖度	85%		
主要层优势种	乔木层：<br>灌木层：<br>草本层：				
群落外貌特点	乔木较多，地表较裸露，草本少				
小地形及样地周围环境描述	村旁，荒废果园				
分层及各层的特点	乔木层	高度			
	灌木层	高度			
	草本层	高度			
	层间植物	高度			
		高度			
备注（之前的土地利用状况）	0.14 kg				

说明：数据尽可能填写全面，没有填无

乔木层植物群落调查表

群落名称：秋枫　　样方面积：20 m × 20 m　　野外编号：35

调查时间：　　记录者：　　室内编号：

编号	植物名称	高度（m）	胸径（cm）	冠幅（m）	物候期	生活力	备注
1	土坛树	16	30	5×5	叶	1	
2	杨桃	12	35	3×5	叶	1	
3	秋枫	12	30	4×4	叶	1	
4	秋枫	12	30	3×3	叶	1	
5	秋枫	15	25	3×2	叶	1	
6	秋枫	12	20	3×3	叶	1	
7	秋枫	13	40	4×4	叶花	1	
8	猫尾木	18	40	4×3	果	1	
9	龙眼	15	40	5×4	叶	1	
10	山楝	15	40	5×5	叶	1	
11	猫尾木	15	35	4×4	果	1	
12	毛八角枫	18	45	4×4	休眠	3	
13							
14							
15							
16							
17							
18							
19							
20							
21							
22							
23							
24							
25							
26							
27							
28							
29							
30							
31							
32							
33							
34							
35							

灌丛层植物群落调查表

群落名称：鹊肾树-番木瓜　　样方面积：5 m × 5 m　　野外编号：35
调查时间：　　记录者：　　室内编号：

编号	植物名称	高度（cm）	冠径（cm）	物候期	生活力	盖度%	株数/丛树
1	粗糠柴	100	30	叶	2	20	1
2	番木瓜	50	60	叶	1	40	2
3							
4	赪桐	120	40	叶	2	20	1
5	鹊肾树	170	60	叶	1	50	1
6							
7	木豆	190	30	叶	1	20	2
8	异木患	140	20	叶	2	10	1
9	苎麻	50	30	叶	2	20	1
10							
11							
12							
13							
14							
15							
16							
17							
18							
19							
20							
21							
22							
23							
24							
25							
26							
27							
28							
29							
30							

草本层植物群落调查表

群落名称：海芋-华南毛蕨　　样方面积 1 m × 1 m　　野外编号：35
调查时间：　　记录者：　　室内编号：

编号	植物名称	株高(cm)	盖度(%)	物候期	生活力	备注
1	凤尾蕨	20	20	叶	1	
2	丰花草	10	5	叶花	2	
3						
4	海芋	200	90	叶	1	
5	倒地铃	15	5	叶	1	
6						
7	叶下珠	30	10	叶果	1	
8	糞箕笃	2	5	叶	2	
9						
10	薜荔	1	5	叶	1	
11	马瓞儿	2	20	叶花果	1	
12	华南毛蕨	30	60	叶	1	
13	黄鹌菜	40	10	叶花果	2	
14						
15	金腰箭	25	5	叶花果	2	
16	火炭母	20	5	叶花果	1	
17	革命菜	15	5	叶花	2	
18	藿香蓟	30	10	叶花	1	
19	飞机草	50	10	叶花	1	
20	假蒟	10	5	叶	2	
21						
22						
23						
24						
25						
26						
27						
28						
29						
30						

说明：物候期：花、叶、果
生活力：1　良好 2　一般　3　较差

总表

群落名称<br>乔-灌-草<br>优势种	荔枝-毛柿-吐烟花			野外编号<br>(统一编号)	36
记录者		日期		室内编号	36
样地面积	20 m×20 m	详细地点			
GPS 定位	N：19 °56.397′<br>E：110 °13.370′	海拔高度	112 m		
群落高度			群落的总盖度	95％	
主要层优势种	乔木层：<br>灌木层：<br>草本层：				
群落外貌特点	人工林				
小地形及样地周围环境描述	荔枝龙眼园植物状况良好				
分层及各层的特点	乔木层	高度			
	灌木层	高度			
	草本层	高度			
	层间植物	高度			
		高度			
备注（之前的土地利用状况）	鲜重：0.12 kg				

说明：数据尽可能填写全面，没有填无

乔木层植物群落调查表

群落名称：龙眼-荔枝　　样方面积：20 m × 20 m　　野外编号：36
调查时间：2017.02.16.　8：21　　记录者：　　室内编号：36

编号	植物名称	高度（m）	胸径（cm）	冠幅（m）	物候期	生活力	备注
1	番石榴	10	20	5×2	叶	2	
2							
3	龙眼	12	30	5×6	叶	3	
4	龙眼	10	30	6×6	叶	3	
5	龙眼	14	40	6×6	叶	3	
6	龙眼	10	20	5×5	叶	2	
7	龙眼	5	10	2×3	叶	1	
8	龙眼	12	15	4×5	叶	2	
9							
10	荔枝	13	40	3×4	叶	1	
11	荔枝	10	20	3×3	叶	2	
12	荔枝	10	20	3×3	叶	2	
13	荔枝	6	15	2×3	叶	2	
14	荔枝	15	45	5×3	叶	2	
15							
16							
17	麻楝	10	15	2×4	叶	2	
18							
19	毛八角枫	12	30	4×5	休眠	1	
20							
21							
22							
23							
24							
25							
26							
27							
28							
29							
30							
31							
32							
33							
34							
35							

灌丛层植物群落调查表

群落名称：秋枫-越南悬钩子　　样方面积：1 m × 1 m　　野外编号：36
调查时间：2017.02.16　.8:22　　记录者：　　室内编号：

编号	植物名称	高度（cm）	冠径（cm）	物候期	生活力	盖度%	株数/丛树
1	毛柿	40	30	叶	2	5	
2	阴香	25	10	叶	2	5	
3	九节	30	15	叶	2	2	
4							
5	越南悬钩子	60	15	叶	3	10	
6	秋枫	60	40	叶	2	15	
7							
8	鸦胆子	20	25	叶	2	4	
9							
10							
11							
12							
13							
14							
15							
16							
17							
18							
19							
20							
21							
22							
23							
24							
25							
26							
27							
28							
29							
30							

草本层植物群落调查表

群落名称：吐烟花　　样方面积 1 m × 1 m　　野外编号：36
调查时间：　　记录者：　　室内编号：

编号	植物名称	株高(cm)	盖度(%)	物候期	生活力	备注
1	吐烟花	5	90	叶	3	
2	假蒟	10	1	叶	2	
3						
4	吐烟花	4	90	叶	3	
5	凤尾蕨	20	10	叶	3	
6						
7	吐烟花	5	80	叶	3	
8	厚叶崖爬藤	12	20	叶	2	
9						
10	吐烟花	5	90	叶	3	
11						
12	吐烟花	4	95	叶	2	
13	火炭母	20	5	叶	3	
14						
15						
16						
17						
18						
19						
20						
21						
22						
23						
24						
25						
26						
27						
28						
29						
30						

说明：物候期：花、叶、果
生活力：1　良好 2　一般　3　较差

总表

群落名称<br>乔-灌-草<br>优势种	菠萝蜜-毛柿-鬼针草			野外编号<br>(统一编号)	37
记录者		日期		室内编号	37
样地面积	20×20 m	详细地点			
GPS 定位	N：19°56.209′<br>E：110°13.835′	海拔高度	93 m		
群落高度		群落的总盖度	90%		
主要层优势种	乔木层：<br>灌木层：<br>草本层：				
群落外貌特点	人工林				
小地形及样地周围环境描述	果园，石斛种植地，火山石丰富				
分层及各层的特点	乔木层	高度			
	灌木层	高度			
	草本层	高度			
	层间植物	高度			
		高度			
备注（之前的土地利用状况）	鲜重 0.10 kg				

说明：数据尽可能填写全面，没有填无

乔木层植物群落调查表

群落名称：龙眼-荔枝　　样方面积：20 m × 20 m　　野外编号：37
调查时间：2017.02.16　12：45　　记录者：　　室内编号：

编号	植物名称	高度（m）	胸径（cm）	冠幅（m）	物候期	生活力	备注
1	龙眼	10	40	5×4	叶	3	
2	龙眼	8	30	4×4	叶	3	
3	龙眼	10	40	5×5	叶	3	
4							
5	菠萝蜜	15	40	5×5	叶花果	3	
6	菠萝蜜	12	30	2×4	叶花果	2	
7							
8	荔枝	10	30	4×4	叶	3	
9	荔枝	10	30	4×3	叶	3	
10	荔枝	8	25	4×3	叶	3	
11							
12							
13							
14							
15							
16							
17							
18							
19							
20							
21							
22							
23							
24							
25							
26							
27							
28							
29							
30							
31							
32							
33							
34							
35							

灌丛层植物群落调查表

群落名称：禾串树-大花紫玉盘　　样方面积：5 m × 5 m　　野外编号：37
调查时间：2017.02.16　12：28　　记录者：　　室内编号：

编号	植物名称	高度（cm）	冠径（cm）	物候期	生活力	盖度%	株数 / 丛树
1	牛筋果	130	100	叶	1	10	
2	白楸	200	100	叶	2	10	
3	两面针	40	50	叶	2	20	
4							
5	禾串树	130	50	叶	1	50	
6	破布簕	200	70	叶	2	20	
7							
8	毛柿	190	100	叶	1	20	
9	大花紫玉盘	160	150	叶花	1	30	
10	白饭树	50	40	叶	2	20	
11							
12							
13							
14							
15							
16							
17							
18							
19							
20							
21							
22							
23							
24							
25							
26							
27							
28							
29							
30							

草本层植物群落调查表

群落名称：白花鬼针草　　样方面积 1 m × 1 m　　野外编号：37
调查时间：2017.02.16　　记录者：　　室内编号：

编号	植物名称	株高(cm)	盖度(%)	物候期	生活力	备注
1	飞机草	50	50	花	1	
2	白花鬼针草	40	60	花	1	
3						
4	藿香蓟	20	10	花	2	
5	薇甘菊	160	40	叶	2	
6						
7	斑鸠菊	200	20	花	1	
8	粪箕笃	20	40	叶	1	
9						
10	斑茅	180	10	花	2	
11	鸭趾草	20	20	叶花	2	
12	墨苜蓿	5	20	花	2	
13						
14	金腰箭	50	10	花	2	
15	红毛菜	60	20	花	2	
16	酢浆草	15	10	花果	2	
17						
18						
19						
20						
21						
22						
23						
24						
25						
26						
27						
28						
29						
30						

说明：物候期：花、叶、果
生活力：1　良好 2　一般　3　较差

总表

群落名称乔-灌-草优势种	木麻黄-潺槁木姜子-飞机草			野外编号(统一编号)	38
记录者		日期		室内编号	38
样地面积	20×20 m	详细地点			
GPS 定位	N：19°56.165′<br>E：110°14.701′	海拔高度	87 m		
群落高度			群落的总盖度	90%	
主要层优势种	乔木层：<br>灌木层：<br>草本层：				
群落外貌特点	人工林				
小地形及样地周围环境描述	木麻黄林，菜野间，无杂草，土松软				
分层及各层的特点	乔木层	高度			
	灌木层	高度			
	草本层	高度			
	层间植物	高度			
		高度			
备注（之前的土地利用状况）	鲜重 0.16 kg				

说明：数据尽可能填写全面，没有填无

乔木层植物群落调查表

群落名称：木麻黄　　样方面积：20 m × 20 m　　野外编号：38
调查时间：2017.02.16　13：36　　记录者：　　室内编号：

编号	植物名称	高度（m）	胸径（cm）	冠幅（m）	物候期	生活力	备注
1	木麻黄	7	10	2×2	叶	3	
2	木麻黄	7	10	2×3	叶	3	
3	木麻黄	8	10	2×3	叶	3	
4	木麻黄	4	8	2×2	叶	3	
5							
6	菠萝蜜	10	40	4×4	叶果	2	
7	菠萝蜜	12	40	4×5	叶果	3	
8							
9	龙眼	5	20	2×3	叶	2	
10							
11							
12							
13							
14							
15							
16							
17							
18							
19							
20							
21							
22							
23							
24							
25							
26							
27							
28							
29							
30							
31							
32							
33							
34							
35							

灌丛层植物群落调查表

群落名称：两面针　　样方面积：5 m × 5 m　　野外编号：38
调查时间：2017.02.16　13：40　　记录者：　　室内编号：

编号	植物名称	高度（cm）	冠径（cm）	物候期	生活力	盖度%	株数 / 丛树
1	毛柿	60	30	叶	2	20	
2	华南省藤	190	100	叶	2	20	
3	潺槁木姜子	180	60	叶	2	20	
4							
5	鹊肾树	90	40	叶	3	10	
6							
7	破布簕	50	40	果	2	20	
8	两面针	40	20	叶	2	30	
9							
10							
11							
12							
13							
14							
15							
16							
17							
18							
19							
20							
21							
22							
23							
24							
25							
26							
27							
28							
29							
30							

草本层植物群落调查表

群落名称：金腰箭-飞机草　　样方面积 1 m × 1 m　　野外编号：38
调查时间：2017.02.16　13：49　　记录者：　　室内编号：

编号	植物名称	株高(cm)	盖度(%)	物候期	生活力	备注
1	金腰箭	110	50	叶	2	
2	飞机草	100	50	叶	2	
3	丰花草	20	30	叶花	2	
4						
5	鸭趾草	15	40	叶	2	
6	一点红	10	20	叶花	2	
7						
8	夜香牛	30	30	叶花	2	
9	藿香蓟	20	20	叶花	2	
10						
11	革命菜	40	10	叶花	2	
12	少花龙葵	30	20	花	2	
13						
14	酢浆草	5	30	叶花	1	
15	一年蓬	20	10	叶	2	
16	香附子	5	40	叶花	1	
17						
18						
19						
20						
21						
22						
23						
24						
25						
26						
27						
28						
29						
30						

说明：物候期：花、叶、果
生活力：1　良好 2 一般 3 较差

总表

<table>
<tr><td>群落名称
乔-灌-草
优势种</td><td colspan="3">木棉-白饭树-南美蟛蜞菊</td><td>野外编号
(统一编号)</td><td>39</td></tr>
<tr><td>记录者</td><td colspan="2"></td><td>日期</td><td></td><td>室内编号</td><td></td></tr>
<tr><td>样地面积</td><td colspan="2">20×20 m</td><td>详细地点</td><td colspan="3"></td></tr>
<tr><td>GPS 定位</td><td>N：19°56.324′
E：110°15.107′</td><td>海拔高度</td><td colspan="4">88 m</td></tr>
<tr><td>群落高度</td><td colspan="3"></td><td>群落的总盖度</td><td colspan="2">90%</td></tr>
<tr><td>主要层优势种</td><td colspan="6">乔木层：
灌木层：
草本层：</td></tr>
<tr><td>群落外貌特点</td><td colspan="6">次生林</td></tr>
<tr><td>小地形及样地周围环境描述</td><td colspan="6">路边草从众多，无多少乔木</td></tr>
<tr><td rowspan="5">分层及各层的特点</td><td>乔木层</td><td>高度</td><td colspan="4"></td></tr>
<tr><td>灌木层</td><td>高度</td><td colspan="4"></td></tr>
<tr><td>草本层</td><td>高度</td><td colspan="4"></td></tr>
<tr><td>层间植物</td><td>高度</td><td colspan="4"></td></tr>
<tr><td></td><td>高度</td><td></td><td></td><td colspan="2"></td></tr>
<tr><td>备注（之前的土地利用状况）</td><td colspan="6">鲜重 0.12 kg</td></tr>
</table>

说明：数据尽可能填写全面，没有填无

乔木层植物群落调查表

群落名称：木棉　　样方面积：20 m × 20 m　　野外编号：39
调查时间：2017.02.16　14：05　　记录者：　　室内编号：39

编号	植物名称	高度（m）	胸径（cm）	冠幅（m）	物候期	生活力	备注
1	木棉	15	50	4×4	休眠	1	
2	木棉	14	50	4×4	休眠	1	
3	木棉	12	45	3×4	休眠	1	
4							
5	鱼尾葵	7	15	3×3	叶	3	
6							
7							
8							
9							
10							
11							
12							
13							
14							
15							
16							
17							
18							
19							
20							
21							
22							
23							
24							
25							
26							
27							
28							
29							
30							
31							
32							
33							
34							
35							

灌丛层植物群落调查表

群落名称：倒吊笔-越南悬钩子　　样方面积：5 m × 5 m　　野外编号：39
调查时间：2017.02.16　14：08　　记录者：　　室内编号：

编号	植物名称	高度（cm）	冠径（cm）	物候期	生活力	盖度%	株数/丛树
1	麻风树	140	15	叶	2	10	
2							
3	越南悬钩子	160	200	叶	2	70	
4	倒吊笔	50	100	叶	2	80	
5	潺槁木姜子	130	50	叶	2	30	
6	破布簕	80	50	叶	2	10	
7	鹊肾树	60	30	叶	2	10	
8	马缨丹	140	40	叶花	2	20	
9	白饭树	300	300	叶	3	60	
10							
11	两面针	200	50	叶	2	30	
12	福建茶	50	20	叶	2	10	
13							
14							
15							
16							
17							
18							
19							
20							
21							
22							
23							
24							
25							
26							
27							
28							
29							
30							

草本层植物群落调查表

群落名称：蟛蜞菊-斑茅-白花鬼针草　　样方面积 1 m × 1 m　　野外编号：39
调查时间：　　记录者：　　室内编号：

编号	植物名称	株高(cm)	盖度(%)	物候期	生活力	备注
1	斑茅	250	80	叶花果	3	
2	蟛蜞菊	40	90	叶	1	
3						
4	白花鬼针草	50	70	叶花	1	
5	火炭母	40	20	叶	2	
6						
7	墨苜蓿	15	20	叶花	2	
8	平花草	10	20	叶花	2	
9						
10	黄花稔	15	20	叶花	2	
11	含羞草	10	10	叶	3	
12	叶下珠	25	5	花果	2	
13						
14	飞机草	100	40	花果	2	
15	酢浆草	5	20	花	2	
16	紫心牵牛	5	5	叶花	2	
17						
18						
19						
20						
21						
22						
23						
24						
25						
26						
27						
28						
29						
30						

说明：物候期：花、叶、果
生活力：1　良好 2　一般　3　较差

总表

群落名称乔-灌-草优势种	木麻黄-蓴竹-薇甘菊			野外编号(统一编号)	40
记录者		日期		室内编号	
样地面积	20×20 m	详细地点			
GPS 定位	N：19°56.519′<br>E：110°15.626′	海拔高度	58 m		
群落高度		群落的总盖度	90%		
主要层优势种	乔木层：<br>灌木层：<br>草本层：				
群落外貌特点	人工林				
小地形及样地周围环境描述	竹山，杂草众多，竹子多				
分层及各层的特点	乔木层	高度			
	灌木层	高度			
	草本层	高度			
	层间植物	高度			
		高度			
备注（之前的土地利用状况）	鲜重 0.10 kg				

说明：数据尽可能填写全面，没有填无

乔木层植物群落调查表

群落名称： 木麻黄-对叶榕　　样方面积：20 m × 20 m　　野外编号：40

调查时间：2017.02.16　14：40　　记录者：　　室内编号：40

编号	植物名称	高度（m）	胸径（cm）	冠幅（m）	物候期	生活力	备注
1	对叶榕	8	25	6×3	叶	2	
2	对叶榕	7	20	5×4	叶	2	
3							
4	苦楝	10	40	6×6	叶	1	
5							
6	木麻黄	14	30	2×2	叶	1	
7	木麻黄	10	35	4×3	叶	3	
8	木麻黄	8	20	3×3	叶	2	
9							
10							
11							
12							
13							
14							
15							
16							
17							
18							
19							
20							
21							
22							
23							
24							
25							
26							
27							
28							
29							
30							
31							
32							
33							
34							
35							

灌丛层植物群落调查表

群落名称：白饭树　　样方面积：5 m × 5 m　　野外编号：40
调查时间：2017.02.16　14：43　　记录者：　　室内编号：

编号	植物名称	高度（cm）	冠径（cm）	物候期	生活力	盖度%	株数 / 丛树
1	潺槁木姜子	40	30	叶	2	5	
2	马缨丹	150	40	花果	1	40	
3							
4	白饭树	300	100	叶	3	80	
5	木薯	150	40	叶	2	50	
6							
7							
8							
9							
10							
11							
12							
13							
14							
15							
16							
17							
18							
19							
20							
21							
22							
23							
24							
25							
26							
27							
28							
29							
30							

草本层植物群落调查表

群落名称：节毛乌蔹莓-白花鬼针草　　样方面积 1 m × 1 m　　野外编号：40
调查时间：2017.02.16　14：43　　记录者：　　室内编号：

编号	植物名称	株高(cm)	盖度(%)	物候期	生活力	备注
1	薇甘菊	5	40	叶	1	
2	火炭母	15	5	叶花果	2	
3	一点红	30	5	叶花	2	
4	丰花草	10	5	叶花	2	
5						
6	叶下珠	30	5	叶花果	2	
7	节毛乌蔹梅	200	80	叶花	1	
8						
9	金腰箭	40	5	叶花	2	
10	藿香蓟	10	5	叶花	2	
11						
12	少花龙葵	30	30	叶花果	2	
13	毒爪	40	10	叶花果	2	
14	一年蓬	20	10	叶	1	
15						
16	夜香牛	30	10	叶花	2	
17	鸭趾草	40	40	叶	2	
18	白花鬼针草	120	80	叶花	1	
19						
20						
21						
22						
23						
24						
25						
26						
27						
28						
29						
30						

说明：物候期：花、叶、果
生活力：1　良好 2　一般　3　较差

总表

群落名称<br>乔-灌-草<br>优势种	苎麻-掌叶鱼黄草		野外编号<br>(统一编号)	41
记录者		日期		室内编号	
样地面积	20×20 m	详细地点			
GPS 定位	N：19°55.606′<br>E：110°10.669′	海拔高度	68 m		
群落高度		群落的总盖度	90%		
主要层优势种	乔木层：<br>灌木层：<br>草本层：				
群落外貌特点	无乔木，废弃垃圾堆边，多草本、灌木				
小地形及样地周围环境描述	开发区旁，杂草多				
分层及各层的特点	乔木层	高度			
	灌木层	高度			
	草本层	高度			
	层间植物	高度			
		高度			
备注（之前的土地利用状况）	0.14 kg				

说明：数据尽可能填写全面，没有填无

乔木层植物群落调查表

群落名称：　　样方面积：20 m × 20 m　　野外编号：41

调查时间：　　记录者：　　室内编号：

编号	植物名称	高度（m）	胸径（cm）	冠幅（m）	物候期	生活力	备注
1							
2							
3							
4							
5							
6							
7							
8							
9							
10							
11							
12							
13							
14							
15							
16							
17							
18							
19							
20							
21							
22							
23							
24							
25							
26							
27							
28							
29							
30							
31							
32							
33							
34							
35							

灌丛层植物群落调查表

群落名称：苎麻-马樱丹　　样方面积：5 m × 5 m　　野外编号：41
调查时间：　　记录者：　　室内编号：

编号	植物名称	高度（cm）	冠径（cm）	物候期	生活力	盖度%	株数/丛树
1	苎麻	140	30	叶	1	80	2
2							
3	马缨丹	160	150	叶花	1	70	2
4							
5	肖梵天花	75	80	叶花果	1	40	1
6							
7							
8							
9							
10							
11							
12							
13							
14							
15							
16							
17							
18							
19							
20							
21							
22							
23							
24							
25							
26							
27							
28							
29							
30							

草本层植物群落调查表

群落名称：掌叶山猪菜-白花鬼针草　　样方面积 1 m × 1 m　　野外编号：41
调查时间：　　记录者：　　室内编号：

编号	植物名称	株高(cm)	盖度(%)	物候期	生活力	备注
1	掌叶山猪菜	120	80	叶花	1	
2	飞机草	120	40	叶果	1	
3	红毛草	80	60	叶花	2	
4						
5	白花鬼针草	40	80	叶花	1	
6	地旋花	50	40	叶花果	2	
7						
8	斑茅	300	40	叶花果	2	
9	含羞草	40	5	叶花	2	
10	革命菜	60	10	叶花	2	
11						
12	少花龙葵	20	10	叶花	2	
13	羽芒菊	30	5	叶花	1	
14						
15	土牛膝	140	40	叶果	2	
16	假败酱	60	20	叶花	1	
17	叶下珠	5	20	叶果	2	
18						
19						
20						
21						
22						
23						
24						
25						
26						
27						
28						
29						
30						

说明：物候期：花、叶、果
生活力：1　良好 2　一般　3　较差

总表

群落名称<br>乔-灌-草<br>优势种	荔枝-华南省藤-假蒟			野外编号<br>(统一编号)	42
记录者		日期		室内编号	
样地面积	20×20 m	详细地点			
GPS 定位	N：19°55.896′<br>E：110°11.011′	海拔高度	77 m		
群落高度			群落的总盖度	98%	
主要层优势种	乔木层：<br>灌木层：<br>草本层：				
群落外貌特点	植被丰富，分层明显				
小地形及样地周围环境描述	荔枝林荒废				
分层及各层的特点	乔木层	高度			
	灌木层	高度			
	草本层	高度			
	层间植物	高度			
		高度			
备注（之前的土地利用状况）	0.08 kg				

说明：数据尽可能填写全面，没有填无

乔木层植物群落调查表

群落名称：荔枝　　样方面积：20 m × 20 m　　野外编号：42
调查时间：　　记录者：　　室内编号：

编号	植物名称	高度（m）	胸径（cm）	冠幅（m）	物候期	生活力	备注
1	荔枝	12	40	44	叶	1	
2	荔枝	10	40	34	叶	1	
3	荔枝	15	50	44	叶	1	
4	荔枝	12	30	33	叶	1	
5	荔枝	14	50	55	叶	1	
6	荔枝	15	45	44	叶	1	
7	荔枝	12	60	44	叶	1	
8	荔枝	10	30	33	叶	1	
9	荔枝	12	50	44	叶	1	
10	荔枝	10	30	43	叶	1	
11	荔枝	12	35	33	叶	1	
12	荔枝	10	20	43	叶	1	
13	荔枝	13	30	33	叶	1	
14	毛八角枫	15	25	33	休眠	3	
15	榄仁	12	30	66	叶	2	
16	假柿木姜子	10	10	23	叶	1	
17	土坛树	9	12	11	叶	1	
18	苦楝	15	30	23	花	1	
19							
20							
21							
22							
23							
24							
25							
26							
27							
28							
29							
30							
31							
32							
33							
34							
35							

灌丛层植物群落调查表

群落名称：白藤-矮紫金牛　　样方面积：5 m × 5 m　　野外编号：42
调查时间：　　记录者：　　室内编号：

编号	植物名称	高度（cm）	冠径（cm）	物候期	生活力	盖度%	株数/丛树
1	白藤	180	60	叶	2	80	1
2	毛柿	80	120	叶果	2	60	2
3							
4	矮紫金牛	150	60	叶	1	80	2
5	牛筋藤	120	50	叶	2	40	2
6							
7	潺槁木姜子	110	30	叶	1	20	1
8	鹰爪花	150	160	叶	1	60	1
9	鹊肾树	80	120	叶	2	40	1
10							
11							
12							
13							
14							
15							
16							
17							
18							
19							
20							
21							
22							
23							
24							
25							
26							
27							
28							
29							
30							

草本层植物群落调查表

群落名称：吊竹梅-假蒟-海芋-吐烟花　　样方面积 1 m × 1 m　　野外编号：42
调查时间：　　记录者：　　室内编号：

编号	植物名称	株高(cm)	盖度(%)	物候期	生活力	备注
1	假蒟	30	50	叶	1	
2	吊竹梅	40	70	叶	1	
3	海芋	20	20	叶	1	
4						
5	吐烟花	5	60	叶	2	
6	扭肚藤	100	20	叶	1	
7	假蒟	20	70	叶	1	
8						
9	海南山姜	140	60	叶	2	
10	海芋	200	70	叶	1	
11	麦冬	30	40	叶	2	
12						
13	掌叶山猪菜	30	20	叶	1	
14	乌蔹莓	140	20	叶	2	
15	薜荔	20	10	叶	2	
16						
17	吐烟花	3	70	叶	1	
18	假蒟	20	40	叶	2	
19						
20						
21						
22						
23						
24						
25						
26						
27						
28						
29						
30						

说明：物候期：花、叶、果
生活力：1　良好 2　一般　3　较差

总表

群落名称<br>乔-灌-草<br>优势种	毛八角枫-苎麻-吐烟花		野外编号<br>(统一编号)	43
记录者		日期		室内编号
样地面积	20×20 m	详细地点		
GPS 定位	N：19°55.906′<br>E：110°11.484′	海拔高度	88 m	
群落高度		群落的总盖度	95%	
主要层优势种	乔木层：<br>灌木层：<br>草本层：			
群落外貌特点	次生林			
小地形及样地周围环境描述	火山石多，杂草、灌木多			
分层及各层的特点	乔木层	高度		
	灌木层	高度		
	草本层	高度		
	层间植物	高度		
		高度		
备注（之前的土地利用状况）	0.10 kg			

说明：数据尽可能填写全面，没有填无

乔木层植物群落调查表

群落名称：毛八角枫　　样方面积：20 m × 20 m　　野外编号：43
调查时间：　　记录者：　　室内编号：

编号	植物名称	高度（m）	胸径（cm）	冠幅（m）	物候期	生活力	备注
1	毛八角枫	12	20	8×6	休眠	2	
2	楤木	6	4	2×1.5	叶	1	
3	土坛树	8	6	3×2	叶	2	
4	毛八角枫	12	15	8×6	休眠	1	
5	毛八角枫	10	15	8×6	休眠	1	
6	毛八角枫	8	10	6×7	休眠	1	
7	土坛树	6	4	3×2	叶	1	
8							
9							
10							
11							
12							
13							
14							
15							
16							
17							
18							
19							
20							
21							
22							
23							
24							
25							
26							
27							
28							
29							
30							
31							
32							
33							
34							
35							

灌丛层植物群落调查表

群落名称：马樱丹-两面针　　样方面积：5 m × 5 m　　野外编号：43
调查时间：　　记录者：　　室内编号：

编号	植物名称	高度（cm）	冠径（cm）	物候期	生活力	盖度%	株数/丛树
1	假杜鹃	120	40	叶花	1	20	1
2	九节	200	30	叶	1	20	1
3							
4	酒饼簕	90	50	叶	2	30	1
5	两面针	140	100	叶	1	50	1
6							
7	马缨丹	190	80	叶花	2	50	1
8	牛筋果	180	100	叶	2	40	1
9	苎麻	200	80	叶	1	40	2
10							
11							
12							
13							
14							
15							
16							
17							
18							
19							
20							
21							
22							
23							
24							
25							
26							
27							
28							
29							
30							

草本层植物群落调查表

群落名称：吐烟花-厚叶崖爬藤　　样方面积 1 m × 1 m　　野外编号：43
调查时间：　　记录者：　　室内编号：

编号	植物名称	株高(cm)	盖度(%)	物候期	生活力	备注
1	海南茄	160	10	叶	2	
2	薇甘菊	10	20	叶	2	
3						
4	翼茎白粉藤	140	20	叶	1	
5	蔓生莠竹	100	40	叶	1	
6						
7	掌叶山猪菜	150	40	叶	1	
8	飞机草	120	10	叶	1	
9						
10	肾蕨	20	5	叶	2	
11	厚叶崖爬藤	170	60	叶果	1	
12						
13	吐烟花	5	70	叶	1	
14	山牵牛	120	5	叶	2	
15	粪箕笃	100	10	叶	1	
16	蔓草虫豆	150	5	叶	2	
17						
18						
19						
20						
21						
22						
23						
24						
25						
26						
27						
28						
29						
30						

说明：物候期：花、叶、果
生活力：1　良好 2　一般　3　较差

总表

群落名称乔-灌-草优势种	荔枝-麻疯树-薇甘菊			野外编号(统一编号)	44
记录者		日期		室内编号	
样地面积	20×20 m	详细地点			
GPS 定位	N：19°55.863′<br>E：110°12.389′	海拔高度	118 m		
群落高度		群落的总盖度	95%		
主要层优势种	乔木层：<br>灌木层：<br>草本层：				
群落外貌特点	分层明显，盖度大，植被丰富				
小地形及样地周围环境描述	村旁				
分层及各层的特点	乔木层	高度			
	灌木层	高度			
	草本层	高度			
	层间植物	高度			
		高度			
备注（之前的土地利用状况）	0.12 kg				

说明：数据尽可能填写全面，没有填无

乔木层植物群落调查表

群落名称：乌墨-荔枝　　样方面积：20 m × 20 m　　野外编号：44
调查时间：　　记录者：　　室内编号：

编号	植物名称	高度（m）	胸径（cm）	冠幅（m）	物候期	生活力	备注
1	乌墨	12	45	12×10	叶	1	
2	土坛树	8	15	3×5	叶	2	
3	荔枝	15	50	7×8	叶	1	
4	秋枫	15	35	3×3	叶	1	
5	苦楝	15	25	2×3	叶	1	
6							
7							
8							
9							
10							
11							
12							
13							
14							
15							
16							
17							
18							
19							
20							
21							
22							
23							
24							
25							
26							
27							
28							
29							
30							
31							
32							
33							
34							
35							

灌丛层植物群落调查表

群落名称：楸木-麻疯树　　样方面积：5 m × 5 m　　野外编号：44
调查时间：　　记录者：　　室内编号：

编号	植物名称	高度（cm）	冠径（cm）	物候期	生活力	盖度%	株数/丛树
1	榛叶黄花稔	60	40	花果	1	40	4
2	蓖麻	180	80	叶花	1	50	1
3							
4	番木瓜	200	120	叶花果	1	60	2
5	假杜鹃	60	40	叶花	1	60	2
6							
7	楸木	200	160	叶	2	70	1
8	马缨丹	140	100	花	2	60	2
9	麻风树	250	160	叶	2	70	1
10							
11							
12							
13							
14							
15							
16							
17							
18							
19							
20							
21							
22							
23							
24							
25							
26							
27							
28							
29							
30							

草本层植物群落调查表

群落名称：薇甘菊-紫心牵牛-光荚含羞草-斑茅　　样方面积 1 m × 1 m　　野外编号：44
调查时间：　　记录者：　　室内编号：

编号	植物名称	株高(cm)	盖度(%)	物候期	生活力	备注
1	落葵	40	40	叶花	1	
2	薇甘菊	50	70	叶	1	
3						
4	紫心牵牛	30	70	叶花	1	
5	毒瓜	300	10	叶果	2	
6						
7	光荚含羞草	80	70	叶花	1	
8	金腰箭	70	60	叶果	3	
9	革命菜	60	40	叶花	1	
10						
11	罗勒	50	20	叶	2	
12	假蒟	20	60	叶	1	
13						
14	少花龙葵	60	60	叶花	2	
15	斑茅	300	70	叶	2	
16	假败酱	40	20	叶花	2	
17	竹节草	60	40	叶	1	
18	曼陀罗	60	20	叶花果	1	
19						
20						
21						
22						
23						
24						
25						
26						
27						
28						
29						
30						

说明：物候期：花、叶、果
生活力：1 良好 2 一般 3 较差

总表

<table>
<tr><td>群落名称
乔-灌-草
优势种</td><td colspan="4">水仙柯-酒饼簕-吐烟花</td><td>野外编号
(统一编号)</td><td>45</td></tr>
<tr><td>记录者</td><td colspan="2"></td><td>日期</td><td></td><td>室内编号</td><td></td></tr>
<tr><td>样地面积</td><td colspan="2">20×20 m</td><td>详细地点</td><td colspan="3"></td></tr>
<tr><td>GPS 定位</td><td>N：19°55.611′
E：110°13.032′</td><td>海拔高度</td><td colspan="4">169 m</td></tr>
<tr><td>群落高度</td><td colspan="3"></td><td>群落的总盖度</td><td colspan="2">89%</td></tr>
<tr><td>主要层优势种</td><td colspan="6">乔木层：
灌木层：
草本层：</td></tr>
<tr><td>群落外貌特点</td><td colspan="6">植被丰富</td></tr>
<tr><td>小地形及样地周围环境描述</td><td colspan="6">火山口边</td></tr>
<tr><td rowspan="5">分层及各层的特点</td><td>乔木层</td><td>高度</td><td colspan="4"></td></tr>
<tr><td>灌木层</td><td>高度</td><td colspan="4"></td></tr>
<tr><td>草本层</td><td>高度</td><td colspan="4"></td></tr>
<tr><td>层间植物</td><td>高度</td><td colspan="4"></td></tr>
<tr><td></td><td>高度</td><td colspan="2"></td><td></td><td></td></tr>
<tr><td>备注（之前的土地利用状况）</td><td colspan="6">0.10 kg</td></tr>
</table>

说明：数据尽可能填写全面，没有填无

乔木层植物群落调查表

群落名称：水仙柯　　样方面积：20 m × 20 m　　野外编号：45
调查时间：　　记录者：　　室内编号：

编号	植物名称	高度（m）	胸径（cm）	冠幅（m）	物候期	生活力	备注
1	水仙柯	8	5	1×1	叶	1	
2	水仙柯	7	5	1×1	叶	1	
3	水仙柯	6	4	1×1	叶	1	
4	水仙柯	7	4	2×2	叶	1	
5	水仙柯	6	8	2×2	叶	1	
6	水仙柯	8	12	2×2	叶	1	
7	水仙柯	7	10	2×2	叶	1	
8	水仙柯	6	8	1×1	叶	1	
9	柚子	6	4	1×3	叶	1	
10	柑橘	7	8	1×1	叶	1	
11	麻楝	12	12	1×1	叶	1	
12	柚子	12	10	3×2	花	1	
13	芒果	12	18	3×3	叶	1	
14	木麻黄	18	50	3×5	叶	2	
15							
16							
17							
18							
19							
20							
21							
22							
23							
24							
25							
26							
27							
28							
29							
30							
31							
32							
33							
34							
35							

灌丛层植物群落调查表

群落名称：苎麻-酒饼簕　　样方面积：5 m × 5 m　　野外编号：45
调查时间：　　记录者：　　室内编号：

编号	植物名称	高度（cm）	冠径（cm）	物候期	生活力	盖度%	株数/丛树
1	油茶	140	30	叶	1	20	2
2	九节	150	40	叶	2	20	3
3							
4	苎麻	130	100	叶	1	50	3
5	海南茄	150	40	叶	1	40	1
6							
7	大管	160	40	叶	2	20	1
8	酒饼簕	180	70	叶	2	50	2
9							
10							
11							
12							
13							
14							
15							
16							
17							
18							
19							
20							
21							
22							
23							
24							
25							
26							
27							
28							
29							
30							

草本层植物群落调查表

群落名称：吐烟花-蔓生莠竹　　样方面积 1 m × 1 m　　野外编号：45
调查时间：　　记录者：　　室内编号：

编号	植物名称	株高(cm)	盖度(%)	物候期	生活力	备注
1	华南毛蕨	30	50	叶	1	
2	吐烟花	3	80	叶	1	
3						
4	假蒟	20	40	叶	2	
5	麦冬	40	20	叶	2	
6						
7	淡竹叶	15	10	叶	2	
8	薇甘菊	10	20	叶	2	
9						
10	鸭趾草	20	20	叶	1	
11	海芋	30	10	叶	1	
12						
13	大叶开唇兰	40	5	叶花	1	
14	蔓生莠竹	20	60	叶	1	
15						
16						
17						
18						
19						
20						
21						
22						
23						
24						
25						
26						
27						
28						
29						
30						

说明：物候期：花、叶、果
生活力：1　良好 2　一般 3　较差

总表

群落名称乔-灌-草优势种	苦楝-九节-蔓生莠竹	野外编号(统一编号)	46
记录者		日期	
		室内编号	
样地面积	20×20 m	详细地点	
GPS 定位	N：19°55.656′<br>E：110°13.179′	海拔高度	165 m
群落高度		群落的总盖度	89%
主要层优势种	乔木层：<br>灌木层：<br>草本层：		
群落外貌特点	果园，杂草众多，地被丰富		
小地形及样地周围环境描述	火山口下村旁		
分层及各层的特点	乔木层	高度	
	灌木层	高度	
	草本层	高度	
	层间植物	高度	
		高度	
备注（之前的土地利用状况）	0.10 kg		

说明：数据尽可能填写全面，没有填无

乔木层植物群落调查表

群落名称：苦楝　　样方面积：20 m × 20 m　　野外编号：46

调查时间：　　记录者：　　室内编号：

编号	植物名称	高度（m）	胸径（cm）	冠幅（m）	物候期	生活力	备注
1	苦楝	15	20	8×6	叶	1	
2	苦楝	15	18	8×7	叶	1	
3	毛八角枫	18	25	8×6	叶	1	
4	荔枝	15	30	12×10	叶	1	
5	麻楝	10	15	4×3	叶	1	
6	苦楝	15	25	8×6	叶花	1	
7	苦楝	15	15	8×7	叶花	1	
8	麻楝	12	16	8×6	叶	1	
9	菠萝蜜	8	6	6×5	叶果	1	
10	楤木	8	6	4×5	叶	1	
11							
12							
13							
14							
15							
16							
17							
18							
19							
20							
21							
22							
23							
24							
25							
26							
27							
28							
29							
30							
31							
32							
33							
34							
35							

灌丛层植物群落调查表

群落名称：九节-马樱丹-破布叶　　样方面积：5 m × 5 m　　野外编号：46
调查时间：　　记录者：　　室内编号：

编号	植物名称	高度（cm）	冠径（cm）	物候期	生活力	盖度%	株数/丛树
1	海南茄	30	20	花	1	5	1
2	马缨丹	140	150	花果	1	60	1
3							
4	九节	250	200	花	1	70	1
5	假杜鹃	30	30	果	2	30	1
6							
7	土蜜树	200	160	叶	2	50	1
8	破布叶	170	160	叶	2	60	1
9	潺槁木姜子	80	20	叶	2	5	1
10							
11							
12							
13							
14							
15							
16							
17							
18							
19							
20							
21							
22							
23							
24							
25							
26							
27							
28							
29							
30							

草本层植物群落调查表

群落名称：蔓生莠竹-海芋-吐烟花　　样方面积 1 m × 1 m　　野外编号：46
调查时间：　　记录者：　　室内编号：

编号	植物名称	株高(cm)	盖度(%)	物候期	生活力	备注
1	蔓生莠竹	50	70	叶	1	
2	海芋	60	70	叶	1	
3						
4	粪箕笃	3	40	叶	1	
5	假蒟	30	40	叶	1	
6	飞机草	70	30	叶	1	
7						
8	马瓟儿	150	60	花果	1	
9	吐烟花	5	70	叶	2	
10	肾蕨	20	10	叶	1	
11						
12	一年蓬	20	5	叶	2	
13	苦蘵	25	10	叶花	1	
14						
15	酢浆草	4	40	叶	1	
16	白粉藤	180	30	叶	2	
17	广防风	150	50	叶花果	2	
18						
19						
20						
21						
22						
23						
24						
25						
26						
27						
28						
29						
30						

说明：物候期：花、叶、果
生活力：1　良好 2　一般　3　较差

总表

群落名称<br>乔-灌-草<br>优势种	荔枝-楤木-龙葵			野外编号<br>(统一编号)	47
记录者		日期		室内编号	47
样地面积	20×20 m	详细地点			
GPS 定位	N：19°55.647′<br>E：110°13.912′	海拔高度	109 m		
群落高度		群落的总盖度	90%		
主要层优势种	乔木层：<br>灌木层：<br>草本层：				
群落外貌特点	人工林				
小地形及样地周围环境描述	道路旁，火山石多，荔枝园				
分层及各层的特点	乔木层	高度			
	灌木层	高度			
	草本层	高度			
	层间植物	高度			
		高度			
备注（之前的土地利用状况）	鲜重 0.10 kg				

说明：数据尽可能填写全面，没有填无

乔木层植物群落调查表

群落名称：荔枝-黄皮　　样方面积：20 m × 20 m　　野外编号：47
调查时间：2017.02.16　12：03　　记录者：　　室内编号：47

编号	植物名称	高度（m）	胸径（cm）	冠幅（m）	物候期	生活力	备注
1	黄皮	8	30	2×3	叶	3	
2	黄皮	7	25	2×3	叶	3	
3	黄皮	6	25	3×2	叶	3	
4							
5	荔枝	10	15	2×3	叶	2	
6	荔枝	12	20	3×4	叶	3	
7	荔枝	5	10	2×2	叶	2	
8	荔枝	7	12	2×3	叶	2	
9							
10							
11							
12							
13							
14							
15							
16							
17							
18							
19							
20							
21							
22							
23							
24							
25							
26							
27							
28							
29							
30							
31							
32							
33							
34							
35							

灌丛层植物群落调查表

群落名称：番木瓜　　样方面积：5 m × 5 m　　野外编号：47
调查时间：2017.02.16　11：47　　记录者：　　室内编号：

编号	植物名称	高度（cm）	冠径（cm）	物候期	生活力	盖度%	株数 / 丛树
1	番木瓜	300	150	叶果	1	50	
2							
3	长杆木姜子	50	20	叶	2	10	
4							
5	楤木	140	120	叶	3	10	
6							
7	酒饼簕	40	20	叶	3	20	
8							
9							
10							
11							
12							
13							
14							
15							
16							
17							
18							
19							
20							
21							
22							
23							
24							
25							
26							
27							
28							
29							
30							

草本层植物群落调查表

群落名称：革命菜-酢浆草　　样方面积 1 m × 1 m　　野外编号：47
调查时间：　　记录者：　　室内编号：

编号	植物名称	株高(cm)	盖度(%)	物候期	生活力	备注
1	飞机草	150	30	叶	2	
2	海芋	30	50	叶	1	
3	十万错	30	10	叶	2	
4						
5	鸭趾草	15	10	花	2	
6	少花龙葵	50	40	花	2	
7						
8	藿香蓟	40	30	叶花	3	
9	微甘菊	60	40	叶花	2	
10						
11	土八参	20	10	果	3	
12	革命菜	60	80	叶花果	3	
13						
14	丰花草	40	20	叶花	3	
15	酢浆草	15	60	叶花	2	
16	毒瓜	5	20	果	2	
17						
18	紫茉莉	20	10	花	2	
19						
20						
21						
22						
23						
24						
25						
26						
27						
28						
29						
30						

说明：物候期：花、叶、果
生活力：1　良好 2　一般　3　较差

总表

群落名称乔-灌-草优势种	龙眼-破布叶-薇甘菊			野外编号(统一编号)	48
记录者		日期		室内编号	48
样地面积	20×20 m	详细地点			
GPS 定位	N：19°55.845′<br>E：110°14.397′	海拔高度	86 m		
群落高度			群落的总盖度	80%	
主要层优势种	乔木层：<br>灌木层：<br>草本层：				
群落外貌特点	次生林				
小地形及样地周围环境描述	公路旁荒地，杂草枯死，已打药				
分层及各层的特点	乔木层	高度			
	灌木层	高度			
	草本层	高度			
	层间植物	高度			
		高度			
备注（之前的土地利用状况）	鲜重 0.10 kg				

说明：数据尽可能填写全面，没有填无

乔木层植物群落调查表

群落名称：龙眼　样方面积：20 m × 20 m　野外编号：48

调查时间：2017.02.16　13：05　记录者：　室内编号：48

编号	植物名称	高度（m）	胸径（cm）	冠幅（m）	物候期	生活力	备注
1	文定果	7	20	4×4	叶	2	
2							
3	龙眼	15	60	8×10	叶	3	
4	龙眼	12	50	8×8	叶	3	
5							
6	楤木	4	10	2×2	叶	1	
7							
8	苦楝	12	20	4×3	叶	1	
9							
10	毛八角枫	6	15	3×3	叶	2	
11							
12							
13							
14							
15							
16							
17							
18							
19							
20							
21							
22							
23							
24							
25							
26							
27							
28							
29							
30							
31							
32							
33							
34							
35							

灌丛层植物群落调查表

群落名称：马樱丹-破布叶　　样方面积：5 m × 5 m　　野外编号：48
调查时间：2017.02.16　13：08　　记录者：　　室内编号：

编号	植物名称	高度（cm）	冠径（cm）	物候期	生活力	盖度%	株数 / 丛树
1	猪肚木	40	50	叶	2	10	
2	毛柿	30	30	叶	3	10	
3							
4	马缨丹	170	50	叶花	2	20	
5							
6	鹊肾树	30	40	叶	2	10	
7	破布叶	160	70	叶	2	20	
8							
9	麻风树	140	50	叶	3	10	
10							
11							
12							
13							
14							
15							
16							
17							
18							
19							
20							
21							
22							
23							
24							
25							
26							
27							
28							
29							
30							

草本层植物群落调查表

群落名称：海芋　　样方面积 1 m × 1 m　　野外编号：48
调查时间：2017.02.16　　记录者：　　室内编号：

编号	植物名称	株高(cm)	盖度(%)	物候期	生活力	备注
1	扭肚藤	20	10	叶	2	
2						
3	薇甘菊	100	10	叶	3	
4	绿萝	30	20	叶	3	
5	麒麟尾	60	20	叶	2	
6						
7	鸡屎藤	150	10	叶	3	
8	飞机草	160	40	叶	2	
9						
10	假菊	30	30	叶	2	
11	海芋	40	50	叶	2	
12	刺茄	10	10	叶果	2	
13						
14	吐烟花	5	20	叶	2	
15						
16						
17						
18						
19						
20						
21						
22						
23						
24						
25						
26						
27						
28						
29						
30						

说明：物候期：花、叶、果
生活力：1　良好 2　一般 3　较差

总表

群落名称<br>乔-灌-草<br>优势种	莲雾-热欀花椒-鬼针草			野外编号(统一编号)	49
记录者		日期	2017.01.06<br>13：30	室内编号	
样地面积	20×20 m	详细地点			
GPS 定位	N：19°55.604′<br>E：110°15.377′	海拔高度	87 m		
群落高度		群落的总盖度	80%		
主要层优势种	乔木层：<br>灌木层：<br>草本层：				
群落外貌特点	次生林				
小地形及样地周围环境描述	采石场附近，地势陡，杂草多。				
分层及各层的特点	乔木层	高度			
	灌木层	高度			
	草本层	高度			
	层间植物	高度			
		高度			
备注（之前的土地利用状况）	土壤鲜重：0.14 kg				

说明：数据尽可能填写全面，没有填无

乔木层植物群落调查表

群落名称：黄皮　　样方面积：20 m × 20 m　　野外编号：49
调查时间：2017.01.06　13：32　　记录者：　　室内编号：

编号	植物名称	高度（m）	胸径（cm）	冠幅（m）	物候期	生活力	备注
1	番石榴	3.5	10	3×2	叶	1	
2	黄皮	3.5	10	2×2.5	叶	1	
3	莲雾	4	9	2×2	叶	1	
4	人心果	4	9	2×3	叶	1	
5	槟榔	3.5	10	2×3	叶	1	
6	芒果	3.5	10	2×2	叶	1	
7	黄皮	3.5	8	2×2.5	叶	1	
8	黄皮	3.5	8	2×3	叶	1	
9	荔枝	4	10	2×2	叶	1	
10	荔枝	4	8	2×2	叶	1	
11	番石榴	3.5	10	2×3	叶	1	
12	莲雾	5.5	20	4×3	叶	2	
13	莲雾	5.5	23	4×3	叶	2	
14	番荔枝	4	13	2×2	叶	2	
15	番荔枝	4	10	2×2	叶	2	
16	苦楝	3.5	13	2×3	叶	2	
17							
18							
19							
20							
21							
22							
23							
24							
25							
26							
27							
28							
29							
30							
31							
32							
33							
34							
35							

灌丛层植物群落调查表

群落名称：簕欓花椒-木薯　　样方面积：5 m × 5 m　　野外编号：49

调查时间：2017.01.06　13：40　　记录者：　　室内编号：

编号	植物名称	高度（cm）	冠径（cm）	物候期	生活力	盖度%	株数/丛树
1	簕欓花椒	60	35	叶	2	20	2
2							
3	木薯	100	25	叶	2	25	1
4							
5							
6							
7							
8							
9							
10							
11							
12							
13							
14							
15							
16							
17							
18							
19							
20							
21							
22							
23							
24							
25							
26							
27							
28							
29							
30							

草本层植物群落调查表

群落名称：马樱丹-丰花草　　样方面积 1 m × 1 m　　野外编号：49

调查时间：2017.01.06　13：45　　记录者：　　室内编号：

编号	植物名称	株高(cm)	盖度(%)	物候期	生活力	备注
1	藿香蓟	30	20	花	2	
2	番木瓜	300	20	叶	2	
3	马缨丹	40	30	花	2	
4	丰花草	30	30	花	2	
5	蛇婆子	20	20	叶	2	
6	红毛草	10	20	叶	2	
7						
8	一年蓬	10	10	花	2	
9	鬼针草	15	20	花	2	
10	柔毛山蚂蝗	30	10	叶	2	
11	飞机草	20	10	叶	2	
12	苦蘵	20	5	叶	2	
13						
14	土人参	10	10	叶	2	
15	蝙蝠草	30	20	叶	2	
16	革命菜	25	10	花	2	
17						
18	地旋花	30	15	叶	2	
19	掌叶鱼黄草	20	15	叶	2	
20	一点红	10	5	花	2	
21						
22	梵天花	25	5	花	2	
23	墨苜蓿	15	10	花	2	
24						
25						
26						
27						
28						
29						
30						

说明：物候期：花、叶、果

生活力：1　良好 2　一般　3　较差

总表

群落名称乔-灌-草优势种	菠萝蜜-鹊肾树-假蒟			野外编号(统一编号)	50
记录者		日期		室内编号	
样地面积		详细地点			
GPS 定位	N：19°55.489′<br>E：110°15.806′	海拔高度	90 m		
群落高度			群落的总盖度	80%	
主要层优势种	乔木层：<br>灌木层：<br>草本层：				
群落外貌特点	人工林，荔枝园，火山石众多，地表薄				
小地形及样地周围环境描述	有一定的小菜地，红壤				
分层及各层的特点	乔木层	高度			
	灌木层	高度			
	草本层	高度			
	层间植物	高度			
		高度			
备注（之前的土地利用状况）	鲜重：0.14 kg				

说明：数据尽可能填写全面，没有填无

乔木层植物群落调查表

群落名称：荔枝　　样方面积：20 m × 20 m　　野外编号：50
调查时间：　　记录者：　　室内编号：

编号	植物名称	高度（m）	胸径（cm）	冠幅（m）	物候期	生活力	备注
1	菠萝蜜	10	20	6×4	花果	2	
2	菠萝蜜	8	15	4×3	花果	2	
3	菠萝蜜	6	4	1×1	叶	2	
4	黄皮	4	4	2×1	叶	1	
5	荔枝	2.5	6	3×2	叶	2	
6	荔枝	2	4	3×2	叶	2	
7	荔枝	3	5	4×3	叶	3	
8	荔枝	2.5	5	3×2	叶	3	
9	荔枝	2.8	6	5×3	叶	2	
10	龙眼	6	10	5×3	叶	1	
11	麻楝	7	6	4×3	叶	1	
12	麻楝	8	7	5×3	叶	1	
13	短穗鱼尾葵	4	3	0.6×1	叶	1	
14							
15							
16							
17							
18							
19							
20							
21							
22							
23							
24							
25							
26							
27							
28							
29							
30							
31							
32							
33							
34							
35							

灌丛层植物群落调查表

群落名称：鹊肾树-木豆　　样方面积：5 m × 5 m　　野外编号：50
调查时间：　　记录者：　　室内编号：

编号	植物名称	高度（cm）	冠径（cm）	物候期	生活力	盖度%	株数 / 丛树
1	木豆	280	200	叶花	3	50	3
2							
3	鹊肾树	180	200	叶	3	60	1
4							
5	鹊肾树	120	100	叶	2	40	1
6							
7							
8							
9							
10							
11							
12							
13							
14							
15							
16							
17							
18							
19							
20							
21							
22							
23							
24							
25							
26							
27							
28							
29							
30							

草本层植物群落调查表

群落名称：厚叶崖爬藤-野葛-假蒟　　样方面积 1 m × 1 m　　野外编号：50
调查时间：　　记录者：　　室内编号：

编号	植物名称	株高(cm)	盖度(%)	物候期	生活力	备注
1	厚叶崖爬藤	300	80	叶	2	
2	黄鹌菜	30	5	花	2	
3	牛筋果	200	40	叶	2	
4	藿香蓟	30	50	花	2	
5	丰花草	20	10	花	2	
6	苦蘵	30	5	叶花果	2	
7						
8	小酸浆	20	10	花	3	
9	皱子白花菜	15	10	叶	2	
10	芋	30	40	叶	3	
11	一年蓬	20	10	叶	2	
12						
13	野葛	300	80	叶	2	
14	马缨丹	50	40	花	2	
15	飞扬草	10	5	花	2	
16						
17	掌叶鱼黄草	200	40	叶	2	
18	金腰箭	40	30	花	2	
19	贴生石韦	5	30	叶	2	
20	酢浆草	10	30	叶	2	
21	叶下珠	10	20	花	2	
22						
23	革命菜	20	10	叶	2	
24	夜香牛	30	20	花	2	
25	假蒟	20	80	叶	2	
26						
27						
28						
29						
30						

说明：物候期：花、叶、果
生活力：1　良好 2　一般 3　较差

总表

群落名称<br>乔-灌-草<br>优势种	木棉-马缨丹-鬼针草			野外编号<br>(统一编号)	51
记录者		日期		室内编号	
样地面积	20×20 m	详细地点			
GPS 定位	N：19°55.185′<br>E：110°10.419′	海拔高度	68 m		
群落高度		群落的总盖度	90%		
主要层优势种	乔木层：<br>灌木层：<br>草本层：				
群落外貌特点	沟渠旁次生林，杂草丛生				
小地形及样地周围环境描述	杂草多				
分层及各层的特点	乔木层	高度			
	灌木层	高度			
	草本层	高度			
	层间植物	高度			
		高度			
备注（之前的土地利用状况）	0.10 kg				

说明：数据尽可能填写全面，没有填无

乔木层植物群落调查表

群落名称：木棉　　样方面积：20 m × 20 m　　野外编号：51
调查时间：　　记录者：　　室内编号：

编号	植物名称	高度（m）	胸径（cm）	冠幅（m）	物候期	生活力	备注
1	木棉	12	20	6×8	休眠	2	
2	木棉	10	18	6×3	休眠	2	
3	木棉	9	15	7×8	休眠	3	
4	木棉	8	12	6×5	休眠	2	
5	木棉	11	19	8×9	休眠	2	
6	木棉	10	15	8×9	休眠	2	
7	木棉	8	12	8×5	休眠	2	
8	木棉	7	10	8×7	休眠	2	
9	木棉	8	12	6×3	休眠	2	
10	木棉	9	13	7×6	休眠	2	
11	木棉	10	16	8×10	休眠	2	
12	木棉	11	18	8×8	休眠	2	
13	苦楝	5	4	1×1	叶	1	
14	对叶榕	7	12	4×4	叶	1	
15	鱼尾葵	8	15	3×3	叶	1	
16							
17							
18							
19							
20							
21							
22							
23							
24							
25							
26							
27							
28							
29							
30							
31							
32							
33							
34							
35							

灌丛层植物群落调查表

群落名称：苎麻-马樱丹　　样方面积：5 m × 5 m　　野外编号：51
调查时间：　　记录者：　　室内编号：

编号	植物名称	高度（cm）	冠径（cm）	物候期	生活力	盖度%	株数/从树
1	马缨丹	200	140	叶花	1	70	1
2	破布叶	170	120	叶	2	50	1
3							
4	苎麻	160	100	叶	1	80	2
5	假杜鹃	120	60	叶	1	40	1
6							
7	牛筋果	200	120	叶	2	40	1
8	白饭树	180	80	叶	2	20	1
9							
10							
11							
12							
13							
14							
15							
16							
17							
18							
19							
20							
21							
22							
23							
24							
25							
26							
27							
28							
29							
30							

草本层植物群落调查表

群落名称：白花鬼针草-斑茅　　样方面积 1 m × 1 m　　野外编号：51
调查时间：　　记录者：　　室内编号：

编号	植物名称	株高(cm)	盖度(%)	物候期	生活力	备注
1	白花鬼针草	40	80	叶花	2	
2	羽芒菊	10	20	叶花	2	
3						
4	斑茅	400	80	叶花果	3	
5	蔓生莠竹	40	60	叶	2	
6	一年蓬	10	10	叶	2	
7						
8	薇甘菊	2	15	叶	2	
9	地旋花	15	5	叶花	2	
10	飞机草	150	60	叶果	3	
11						
12	夜香牛	30	5	叶花	2	
13	赛葵	100	10	叶果	2	
14						
15	火炭母	160	10	叶	2	
16	紫心牵牛	90	20	叶花	2	
17	山牵牛	200	40	叶	1	
18	假败酱	100	50	叶花	1	
19						
20						
21						
22						
23						
24						
25						
26						
27						
28						
29						
30						

说明：物候期：花、叶、果
生活力：1　良好 2　一般　3　较差

总表

群落名称<br>乔-灌-草<br>优势种	构树-马缨丹-斑茅			野外编号<br>(统一编号)	52
记录者		日期		室内编号	
样地面积	20×20 m	详细地点			
GPS 定位	N：19°55.155′<br>E：110°11.174′	海拔高度	73m		
群落高度		群落的总盖度	98%		
主要层优势种	乔木层：<br>灌木层：<br>草本层：				
群落外貌特点	次生林				
小地形及样地周围环境描述	杂草多、斑茅多				
分层及各层的特点	乔木层	高度			
	灌木层	高度			
	草本层	高度			
	层间植物	高度			
		高度			
备注（之前的土地利用状况）	0.10 kg				

说明：数据尽可能填写全面，没有填无

乔木层植物群落调查表

群落名称：构树　　样方面积：20 m × 20 m　　野外编号：52
调查时间：　　记录者：　　室内编号：

编号	植物名称	高度（m）	胸径（cm）	冠幅（m）	物候期	生活力	备注
1	构树	7	10	6×5	叶	2	
2	构树	6	8	5×4	叶	2	
3	构树	7	10	6×5	叶	2	
4	构树	8	12	6×6	叶	2	
5	构树	5	10	5×6	叶	2	
6	构树	6	10	6×5	叶	2	
7	构树	7	11	6×5	叶	2	
8	构树	7	11	6×5	叶	2	
9	构树	8	12	6×6	叶	2	
10	构树	6	10	5×4	叶	2	
11	构树	5	8	5×5	叶	2	
12	构树	7	10	5×6	叶	2	
13	构树	8	12	6×5	叶	2	
14	构树	7	13	6×6	叶	2	
15	构树	8	12	6×6	叶	2	
16	构树	6	10	6×5	叶	2	
17	苦楝	6	10	3×2	叶	1	
18	苦楝	8	8	4×3	叶	1	
19							
20							
21							
22							
23							
24							
25							
26							
27							
28							
29							
30							
31							
32							
33							
34							
35							

灌丛层植物群落调查表

群落名称：马樱丹　　样方面积：5 m × 5 m　　野外编号：52
调查时间：　　记录者：　　室内编号：

编号	植物名称	高度（cm）	冠径（cm）	物候期	生活力	盖度%	株数/丛树
1	白饭树	120	40	叶	1	20	1
2	苎麻	200	30	叶	2	20	1
3							
4	马缨丹	200	160	叶花	1	70	1
5	桑树	170	30	叶	2	10	1
6	鹊肾树	160	40	叶	2	20	1
7							
8	对叶榕	60	20	叶	2	20	1
9							
10							
11							
12							
13							
14							
15							
16							
17							
18							
19							
20							
21							
22							
23							
24							
25							
26							
27							
28							
29							
30							

草本层植物群落调查表

群落名称：斑茅-白花鬼针草-蔓生莠竹　　样方面积 1 m × 1 m　　野外编号：52
调查时间：　　记录者：　　室内编号：

编号	植物名称	株高(cm)	盖度(%)	物候期	生活力	备注
1	斑茅	300	80	叶果	2	
2	蔓生莠竹	150	60	叶	2	
3						
4	白花鬼针草	60	70	叶花	2	
5	含羞草	5	10	叶花	2	
6	掌叶山猪菜	5	5	叶	2	
7						
8	蛇葡萄	2	40	叶	1	
9	藿香蓟	30	20	叶花	1	
10						
11	野甘草	20	10	叶花果	2	
12	紫心牵牛	60	40	叶花	2	
13						
14	山牵牛	200	40	叶	2	
15	火炭母	20	30	叶花	2	
16						
17						
18						
19						
20						
21						
22						
23						
24						
25						
26						
27						
28						
29						
30						

说明：物候期：花、叶、果
生活力：1　良好 2　一般　3　较差

总表

群落名称<br>乔-灌-草<br>优势种	荔枝-倒吊笔-假蒟				野外编号<br>(统一编号)	53
记录者			日期		室内编号	
样地面积	20×20 m		详细地点			
GPS 定位	N：19°55.258′<br>E：110°11.587′	海拔高度	89m			
群落高度				群落的总盖度	89%	
主要层优势种	乔木层：<br>灌木层：<br>草本层：					
群落外貌特点	村落边，植被丰富					
小地形及样地周围环境描述	地被多为假蒟					
分层及各层的特点	乔木层	高度				
	灌木层	高度				
	草本层	高度				
	层间植物	高度				
		高度				
备注（之前的土地利用状况）	0.10 kg					

说明：数据尽可能填写全面，没有填无

乔木层植物群落调查表

群落名称：土坛树-荔枝　　样方面积：20 m × 20 m　　野外编号：53
调查时间：　　记录者：　　室内编号：

编号	植物名称	高度（m）	胸径（cm）	冠幅（m）	物候期	生活力	备注
1	大叶野樱	12	40	6×8	叶	1	
2	荔枝	18	60	8×8	叶	1	
3	荔枝	16	50	7×8	叶	1	
4	荔枝	9	25	5×5	叶	1	
5	土坛树	15	30	4×5	叶	2	
6	土坛树	12	18	3×3	叶	2	
7	土坛树	12	20	3×3	叶	2	
8	土坛树	10	20	4×5	叶	2	
9	椰子	18	4	3×3	叶果	1	
10							
11							
12							
13							
14							
15							
16							
17							
18							
19							
20							
21							
22							
23							
24							
25							
26							
27							
28							
29							
30							
31							
32							
33							
34							
35							

灌丛层植物群落调查表

群落名称：蓖麻-倒吊笔　　样方面积：5 m × 5 m　　野外编号：53
调查时间：　　记录者：　　室内编号：

编号	植物名称	高度（cm）	冠径（cm）	物候期	生活力	盖度%	株数/丛树
1	鸦胆子	170	60	叶	1	40	1
2	蓖麻	200	150	叶果	1	70	2
3							
4	倒吊笔	300	160	叶果	1	70	1
5	山小橘	180	100	叶	2	50	1
6	对叶榕	170	120	叶	2	50	1
7							
8	鹊肾树	180	50	叶	1	40	1
9	酒饼簕	140	70	叶	2	40	2
10	毛柿	160	40	叶	2	40	2
11	矮紫金牛	80	40	叶	1	50	2
12	猪肚木	130	50	叶	2	30	1
13							
14							
15							
16							
17							
18							
19							
20							
21							
22							
23							
24							
25							
26							
27							
28							
29							
30							

草本层植物群落调查表

群落名称：海芋-绿萝-假蒟-鸭跖草　　样方面积 1 m × 1 m　　野外编号：53
调查时间：　　记录者：　　室内编号：

编号	植物名称	株高(cm)	盖度(%)	物候期	生活力	备注
1	海芋	60	80	叶花果	1	
2	绿萝	80	80	叶	1	
3	磨盘草	160	40	叶	1	
4						
5	假蒟	40	80	叶	1	
6	麒麟尾	200	60	叶	2	
7						
8	飞机草	120	40	叶	2	
9	厚叶崖爬藤	170	70	叶	1	
10						
11	假蒟	30	80	叶	1	
12	山牵牛	20	10	叶	2	
13						
14	扭肚藤	80	40	叶	1	
15	鸭跖草	30	80	叶	1	
16	蔓生莠竹	50	60	叶果	2	
17						
18						
19						
20						
21						
22						
23						
24						
25						
26						
27						
28						
29						
30						

说明：物候期：花、叶、果
生活力：1　良好 2 一般 3 较差

总表

群落名称乔-灌-草优势种	乌墨-雀梅-蔓生莠竹	野外编号(统一编号)	54
记录者		日期	
		室内编号	
样地面积	20×20 m	详细地点	
GPS 定位	N：19°55.313′<br>E：110°12.181′	海拔高度	115 m
群落高度		群落的总盖度	90%
主要层优势种	乔木层：<br>灌木层：<br>草本层：		
群落外貌特点	人工林		
小地形及样地周围环境描述	村边树林，杂草多		
分层及各层的特点	乔木层	高度	
	灌木层	高度	
	草本层	高度	
	层间植物	高度	
		高度	
备注（之前的土地利用状况）	鲜重 0.12 kg		

说明：数据尽可能填写全面，没有填无

乔木层植物群落调查表

群落名称：乌墨　样方面积：20 m × 20 m　野外编号：54
调查时间：　记录者：　室内编号：

编号	植物名称	高度（m）	胸径（cm）	冠幅（m）	物候期	生活力	备注
1	乌墨	16	50	6×8	叶	1	
2	乌墨	15	45	4×7	叶	1	
3	乌墨	12	25	4×4	叶	1	
4	乌墨	13	30	4×5	叶	1	
5	乌墨	12	30	4×4	叶	1	
6	乌墨	9	25	4×5	叶	1	
7	乌墨	10	35	3×4	叶	1	
8	乌墨	13	35	3×3	休眠	1	
9	乌墨	10	30	4×3	叶	2	
10	秋枫	12	35	4×5	叶	1	
11	海南菜豆树	15	18	2×2	叶	1	
12	菠萝蜜	15	25	4×3	叶果	1	
13	荔枝	10	18	3×3	叶	1	
14	菠萝蜜	14	30	3×3	叶果	1	
15	苦楝	15	20	3×3	叶	1	
16	木麻黄	14	15	3×3	叶	1	
17	苦楝	15	30	5×6	叶	1	
18	龙眼	10	12	4×4	叶	1	
19	龙眼	7	10	3×3	叶	1	
20	海南菜豆树	16	20	3×3	叶花	1	
21	海南菜豆树	12	20	2×2	叶	1	
22							
23							
24							
25							
26							
27							
28							
29							
30							
31							
32							
33							
34							
35							

灌丛层植物群落调查表

群落名称：雀梅藤-毛柿-鹊肾树　　样方面积：5 m × 5 m　　野外编号：54
调查时间：　　记录者：　　室内编号：

编号	植物名称	高度（cm）	冠径（cm）	物候期	生活力	盖度%	株数/丛树
1	白藤	60	50	叶	1	60	2
2	毛柿	160	140	叶	2	70	2
3	雀梅	300	200	叶	1	80	1
4							
5	鹊肾树	180	90	叶	2	70	1
6	破布叶	300	100	叶	1	40	1
7							
8	九节	60	40	叶	2	20	1
9	粗糠柴	100	40	叶	3	20	1
10							
11							
12							
13							
14							
15							
16							
17							
18							
19							
20							
21							
22							
23							
24							
25							
26							
27							
28							
29							
30							

草本层植物群落调查表

群落名称：金腰箭-绿萝-蔓生莠竹　样方面积 1 m × 1 m　　野外编号：54
调查时间：　　记录者：　　室内编号：

编号	植物名称	株高(cm)	盖度(%)	物候期	生活力	备注
1	厚叶崖爬藤	250	60	叶	1	
2	凤尾蕨	40	5	叶	2	
3						
4	麒麟尾	300	70	叶	1	
5	金腰箭	50	80	叶果	3	
6	绿萝	300	80	叶	2	
7						
8	抱树莲	400	40	叶	2	
9	吐烟花	5	70	叶	1	
10						
11	飞机草	20	5	叶	1	
12	薇甘菊	120	40	叶	1	
13						
14	蔓生莠竹	80	80	叶花	1	
15	飞机草	120	40	叶花果	1	
16	糞箕笃	20	5	叶	1	
17	络石	140	20	叶	1	
18						
19						
20						
21						
22						
23						
24						
25						
26						
27						
28						
29						
30						

说明：物候期：花、叶、果
生活力：1　良好 2　一般　3　较差

总表

<table>
<tr><td>群落名称
乔-灌-草
优势种</td><td colspan="3">荔枝-破布叶-飞机草</td><td>野外编号
(统一编
号)</td><td>55</td></tr>
<tr><td>记录者</td><td colspan="2"></td><td>日期</td><td></td><td>室内编号</td></tr>
<tr><td>样地面积</td><td colspan="2"></td><td>详细地
点</td><td colspan="2"></td></tr>
<tr><td>GPS 定位</td><td>N：19 °55.196´

E：110°12.742´</td><td>海
拔
高
度</td><td colspan="3">132 m</td></tr>
<tr><td>群落高度</td><td colspan="2"></td><td>群落的总
盖度</td><td colspan="2">90%</td></tr>
<tr><td>主要层优
势种</td><td colspan="5">乔木层：
灌木层：
草本层：</td></tr>
<tr><td>群落外貌
特点</td><td colspan="5">人工林</td></tr>
<tr><td>小地形及
样地周围
环境描述</td><td colspan="5">荔枝园 火山石众多 公路两旁</td></tr>
<tr><td rowspan="5">分层及各
层的特点</td><td>乔木层</td><td>高度</td><td colspan="3"></td></tr>
<tr><td>灌木层</td><td>高度</td><td colspan="3"></td></tr>
<tr><td>草本层</td><td>高度</td><td colspan="3"></td></tr>
<tr><td>层间植物</td><td>高度</td><td colspan="3"></td></tr>
<tr><td></td><td>高度</td><td></td><td></td><td></td></tr>
<tr><td>备注（之
前的土地
利用状
况）</td><td colspan="5">鲜重：0.12 kg</td></tr>
</table>

说明：数据尽可能填写全面，没有填无

乔木层植物群落调查表

群落名称：荔枝　　样方面积：20 m × 20 m　　野外编号：55

调查时间：2017.02.16.　10：35　　记录者：　　室内编号：

编号	植物名称	高度（m）	胸径（cm）	冠幅（m）	物候期	生活力	备注
1	荔枝	15	60	7×8	叶	3	
2	荔枝	12	50	6×6	叶	3	
3	荔枝	10	30	5×5	叶	2	
4	荔枝	13	40	6×5	叶	3	
5	荔枝	14	50	5×6	叶	3	
6	荔枝	15	60	7×8	叶	3	
7							
8							
9							
10	秋枫	17	80	6×5	叶	3	
11							
12							
13	橘子树	3	10	1×0.5	叶	1	
14	橘子树	1.5	6	1×0.5	叶	1	
15	橘子树	4	10	1×1	叶	1	
16							
17							
18							
19							
20							
21							
22							
23							
24							
25							
26							
27							
28							
29							
30							
31							
32							
33							
34							
35							

灌丛层植物群落调查表

群落名称：破布叶　　样方面积：1 m × 1 m　　野外编号：55
调查时间：2017.02.16.　10：35　　记录者：　　室内编号：

编号	植物名称	高度（cm）	冠径（cm）	物候期	生活力	盖度%	株数 / 丛树
1	毛柿	100	40	叶	1	10	
2	猪肚木	140	30	叶	2	10	
3	黄牛木	50	45	叶	2	20	
4							
5	九节	30	40	叶	2	30	
6	酒饼簕	50	30	叶	1	10	
7	潺槁木姜子	165	40	叶	2	30	
8	假杜鹃	50	30	花	1	20	
9							
10	破布叶	200	150	叶	1	40	
11	破布叶	200	150	叶	1	40	
12							
13							
14							
15							
16							
17							
18							
19							
20							
21							
22							
23							
24							
25							
26							
27							
28							
29							
30							

草本层植物群落调查表

群落名称：吐烟花-假蒟-飞机草　　样方面积 1 m × 1 m　　野外编号：55
调查时间：　　记录者：　　室内编号：

编号	植物名称	株高(cm)	盖度(%)	物候期	生活力	备注
1	薯莨	20	10	叶	2	
2	福建茶	50	30	叶	1	
3	吐烟花	5	70	叶	1	
4	薇甘菊	15	30	叶	2	
5	蔓生莠竹	60	40	叶	2	
6	翼茎白粉藤	50	20	叶	2	
7	厚叶崖爬藤	50	40	叶	2	
8						
9						
10	假蒟	20	60	叶	2	
11						
12	飞机草	80	60	叶	3	
13	槲蕨	30	20	叶	2	
14	麦冬	30	20	果	2	
15	线柱兰	30	10	叶花	3	
16						
17						
18						
19						
20						
21						
22						
23						
24						
25						
26						
27						
28						
29						
30						

说明：物候期：花、叶、果
生活力：1　良好 2　一般　3　较差

总表

群落名称<br>乔-灌-草<br>优势种	荔枝-番石榴-假蒟			野外编号<br>(统一编号)	56
记录者		日期		室内编号	56
样地面积	20 m×20 m	详细地点			
GPS 定位	N：19 °55.212′<br>E：110°13.317′	海拔高度	147 m		
群落高度		群落的总盖度			
主要层优势种	乔木层：<br>灌木层：<br>草本层：				
群落外貌特点	人工林				
小地形及样地周围环境描述	菠萝蜜林　小树苗				
分层及各层的特点	乔木层	高度			
	灌木层	高度			
	草本层	高度			
	层间植物	高度			
		高度			
备注（之前的土地利用状况）	鲜重：0.12 kg				

说明：数据尽可能填写全面，没有填无

乔木层植物群落调查表

群落名称：荔枝　　样方面积：　　野外编号：56

调查时间：2017.02.16　.9：15　　记录者：　　室内编号：56

编号	植物名称	高度（m）	胸径（cm）	冠幅（m）	物候期	生活力	备注
1	荔枝	12	60	7×6	叶	3	
2	荔枝	13	5	8×6	叶	3	
3	荔枝	15	60	10×10	叶	3	
4	荔枝	12	50	7×7	叶	3	
5	苦楝	10	30	5×6	叶	3	
6							
7							
8	菠萝蜜	12	50	6×6	叶	3	
9							
10							
11							
12							
13							
14							
15							
16							
17							
18							
19							
20							
21							
22							
23							
24							
25							
26							
27							
28							
29							
30							
31							
32							
33							
34							
35							

灌丛层植物群落调查表

群落名称：苦楝　　样方面积：1 m × 1 m　　野外编号：56
调查时间：2017.02.16　.9：15　　记录者：　　室内编号：

编号	植物名称	高度（cm）	冠径（cm）	物候期	生活力	盖度%	株数/丛树
1	番木瓜	150	35	叶	1	2	
2	番木瓜	170	70	果	1	4	
3							
4	杨桃	170	70	叶	2	4	
5							
6	潘石榴	180	60	叶	2	10	
7							
8	苦楝	50	40	叶	3	60	
9	梵天花	120	40	叶果	2	5	
10	鸦胆子	140	20	叶	2	5	
11	簕榄花椒	20	20	叶	2	5	
12							
13							
14							
15							
16							
17							
18							
19							
20							
21							
22							
23							
24							
25							
26							
27							
28							
29							
30							

草本层植物群落调查表

群落名称：假蒟　　样方面积 1 m × 1 m　　野外编号：56
调查时间：2017.02.16　9：22　　记录者：　　室内编号：

编号	植物名称	株高(cm)	盖度(%)	物候期	生活力	备注
1	金腰箭	60	20	叶花果	2	
2	藿香蓟	40	10	花	1	
3	芬花草	10	10	花	2	
4						
5	一年蓬	15	10	叶	1	
6	凤尾蕨	20	10	叶	1	
7	鸭趾草	30	20	叶	2	
8						
9	夜香牛	30	20	花	1	
10						
11	飞机草	140	10	叶	1	
12						
13	小花龙葵	40	20	花	2	
14	海芋	35	30	叶	1	
15	假蒟	45	60	叶	2	
16	吐烟花	15	20	叶	2	
17						
18						
19						
20						
21						
22						
23						
24						
25						
26						
27						
28						
29						
30						

说明：物候期：花、叶、果
生活力：1　良好 2　一般　3　较差

总表

群落名称乔-灌-草优势种	荔枝-油茶-边缘鳞盖蕨			野外编号(统一编号)	57
记录者		日期		室内编号	
样地面积		详细地点			
GPS 定位	N：19°55.405′<br>E：110°13.812′	海拔高度	129 m		
群落高度			群落的总盖度	95%	
主要层优势种	乔木层：<br>灌木层：<br>草本层：				
群落外貌特点	次生林				
小地形及样地周围环境描述	蕨类众多，荔枝园，道旁				
分层及各层的特点	乔木层	高度			
	灌木层	高度			
	草本层	高度			
	层间植物	高度			
		高度			
备注（之前的土地利用状况）	鲜重 0.12 kg				

说明：数据尽可能填写全面，没有填无

乔木层植物群落调查表

群落名称：荔枝　　样方面积：20 m × 20 m　　野外编号：57
调查时间：2017.02.16　11：20　　记录者：　　室内编号：57

编号	植物名称	高度（m）	胸径（cm）	冠幅（m）	物候期	生活力	备注
1	荔枝	10	40	5×5	叶	3	
2	荔枝	12	50	6×7	叶	2	
3	荔枝	10	35	5×6	叶	2	
4	荔枝	11	40	6×6	叶	2	
5							
6	黄皮	7	6	2×3	叶	3	
7							
8	鱼尾葵	7	4	2×2	叶	3	
9							
10	土坛树	6	10	2×2	叶	2	
11							
12							
13							
14							
15							
16							
17							
18							
19							
20							
21							
22							
23							
24							
25							
26							
27							
28							
29							
30							
31							
32							
33							
34							
35							

灌丛层植物群落调查表

群落名称：山黄麻-猪肚木　　样方面积：5 m × 5 m　　野外编号：57
调查时间：2017.02.16　11：20　　记录者：　　室内编号：

编号	植物名称	高度（cm）	冠径（cm）	物候期	生活力	盖度%	株数/丛树
1	水仙柯	190	40	叶	2	20	
2							
3	山黄麻	140	100	叶	2	30	
4	猪肚木	200	80	叶	2	30	
5							
6	油茶	310	150	叶	1	20	
7							
8	两面针	150	40	叶	2	20	
9	紫玉盘	130	50	叶	2	10	
10							
11							
12							
13							
14							
15							
16							
17							
18							
19							
20							
21							
22							
23							
24							
25							
26							
27							
28							
29							
30							

草本层植物群落调查表

群落名称：吐烟花-假蒟　　样方面积 1 m × 1 m　　野外编号：57
调查时间：2017.02.16　　记录者：　　室内编号：

编号	植物名称	株高(cm)	盖度(%)	物候期	生活力	备注
1	假蒟	20	50	叶	1	
2						
3	海芋	140	20	果	1	
4	薇甘菊	50	20	叶	2	
5						
6	吐烟花	5	60	叶	2	
7						
8	厚叶崖爬藤	15	20	叶	2	
9						
10	海金花	20	10	叶	2	
11	露兜	30	10	叶	2	
12	马瓞儿	30	5	叶花果	3	
13						
14						
15						
16						
17						
18						
19						
20						
21						
22						
23						
24						
25						
26						
27						
28						
29						
30						

说明：物候期：花、叶、果
生活力：1　良好 2　一般　3　较差

总表

群落名称<br>乔-灌-草<br>优势种	苦楝-白背叶-飞机草			野外编号<br>(统一编号)	58
记录者		日期	2017.2.17<br>8：02	室内编号	
样地面积	20×20 m	详细地点			
GPS 定位	N：19°54.704′<br>E：110°14.403′	海拔高度	107 m		
群落高度		群落的总盖度	85%		
主要层优势种	乔木层：<br>灌木层：<br>草本层：				
群落外貌特点	次生林				
小地形及样地周围环境描述	县道旁、乔木较少、灌木众多				
分层及各层的特点	乔木层	高度			
	灌木层	高度			
	草本层	高度			
	层间植物	高度			
		高度			
备注（之前的土地利用状况）	鲜重：0.12 kg				

说明：数据尽可能填写全面，没有填无

乔木层植物群落调查表

群落名称：苦楝-桉树　　样方面积：　　野外编号：58

调查时间：2017.02.17　8：02　　记录者：　　室内编号：

编号	植物名称	高度（m）	胸径（cm）	冠幅（m）	物候期	生活力	备注
1	桉树	6	15	1×1	叶	2	
2	桉树	6	15	1×1	叶	2	
3	桉树	5.5	12	1×1	叶	2	
4	桉树	7	15	1×1	叶	2	
5	桉树	5	10	1×1	叶	2	
6	桉树	5	10	1×1	叶	2	
7	苦楝	5	8	1×1.5	叶	2	
8	苦楝	7	10	1×2	叶	2	
9	苦楝	6.5	10	1×2	叶	2	
10	苦楝	5	10	1×1.5	叶	2	
11	苦楝	5	8	2×2.5	叶	2	
12	苦楝	6	10	2×1	叶	2	
13	苦楝	7	10	2×3	叶	2	
14	苦楝	5.5	8	2×1	叶	2	
15	苦楝	4.8	6	2×2	叶	2	
16	苦楝	5	10	2×2	叶	2	
17	苦楝	7	10	2×2	叶	2	
18	苦楝	8	15	2×2	叶	2	
19							
20							
21							
22							
23							
24							
25							
26							
27							
28							
29							
30							
31							
32							
33							
34							
35							

灌丛层植物群落调查表

群落名称：马樱丹-白楸　　样方面积：5 m × 5 m　　野外编号：58
调查时间：　2017.02.17　8：02　　记录者：　　室内编号：

编号	植物名称	高度（cm）	冠径（cm）	物候期	生活力	盖度%	株数 / 丛树
1	马樱丹	150	120	叶花	1	60	1
2	猪肚木	80	40	叶	1	20	1
3	酒饼簕	120	60	叶	1	20	1
4	破布叶	110	40	叶果	2	40	1
5	白楸	150	120	叶花	1	50	1
6	大青	100	20	叶	2	10	1
7	野牡丹	120	80	叶	1	40	1
8	簕欓花椒	80	120	叶	1	30	1
9	白藤	80	120	叶	1	40	1
10							
11							
12							
13							
14							
15							
16							
17							
18							
19							
20							
21							
22							
23							
24							
25							
26							
27							
28							
29							
30							

草本层植物群落调查表

群落名称：破铜钱-飞机草　　样方面积 1 m × 1 m　　野外编号：58
调查时间：2017.02.17　8：15　　记录者：　　室内编号：

编号	植物名称	株高(cm)	盖度(%)	物候期	生活力	备注
1	破铜钱	2	80	叶	1	
2	大叶油草	5	40	叶	1	
3	丰花草	16	5	叶	1	
4	破铜钱	3	40	叶	1	
5	一点红	10	5	叶花	1	
6	含羞草	15	10	叶花	1	
7	蛇婆子	15	5	叶	2	
8	墨苜蓿	5	10	叶花	2	
9	榛叶黄花稔	15	20	叶花	2	
10	三点金	3	40	叶	2	
11	酢浆草	6	20	叶花果	1	
12	臭矢菜	15	10	叶花果	1	
13	飞机草	120	60	叶花果	1	
14	决明	50	40	叶花果	1	
15						
16						
17						
18						
19						
20						
21						
22						
23						
24						
25						
26						
27						
28						
29						
30						

说明：物候期：花、叶、果
生活力：1　良好 2　一般　3　较差

总表

<table>
<tr><td>群落名称
乔-灌-草
优势种</td><td colspan="4">水仙柯-破布叶-斑茅</td><td>野外编号
(统一编号)</td><td>59</td></tr>
<tr><td>记录者</td><td colspan="2"></td><td>日期</td><td>2017.1.6
14：10</td><td>室内编号</td><td></td></tr>
<tr><td>样地面积</td><td colspan="2">20×20 m</td><td>详细地点</td><td colspan="3"></td></tr>
<tr><td>GPS 定位</td><td>N：19°55。184′
E：110°15.095′</td><td>海拔高度</td><td colspan="4">93 m</td></tr>
<tr><td>群落高度</td><td colspan="3"></td><td>群落的总盖度</td><td colspan="2">65%</td></tr>
<tr><td>主要层优势种</td><td colspan="6">乔木层：
灌木层：
草本层：</td></tr>
<tr><td>群落外貌特点</td><td colspan="6">次生林</td></tr>
<tr><td>小地形及样地周围环境描述</td><td colspan="6">乡野小山坡，杂草多</td></tr>
<tr><td rowspan="5">分层及各层的特点</td><td>乔木层</td><td>高度</td><td colspan="4"></td></tr>
<tr><td>灌木层</td><td>高度</td><td colspan="4"></td></tr>
<tr><td>草本层</td><td>高度</td><td colspan="4"></td></tr>
<tr><td>层间植物</td><td>高度</td><td colspan="4"></td></tr>
<tr><td></td><td>高度</td><td colspan="2"></td><td></td><td></td></tr>
<tr><td>备注（之前的土地利用状况）</td><td colspan="6">土壤鲜重：0.10 kg</td></tr>
</table>

说明：数据尽可能填写全面，没有填无

乔木层植物群落调查表

群落名称：水仙柯　　样方面积：20 m × 20 m　　野外编号：59
调查时间：2017.01.6　14：15　　记录者：　　室内编号：

编号	植物名称	高度（m）	胸径（cm）	冠幅（m）	物候期	生活力	备注
1	水仙柯	4.5	14	2×2	叶	2	
2	荔枝	7	23	3×4	叶	2	
3	水仙柯	7	25	4×4	叶	2	
4	黄花梨	3.5	6	2×2	叶	2	
5	麻楝	6.5	20	2×3	叶	2	
6	热櫾花椒	5	11	2×2	叶	1	
7							
8							
9							
10							
11							
12							
13							
14							
15							
16							
17							
18							
19							
20							
21							
22							
23							
24							
25							
26							
27							
28							
29							
30							
31							
32							
33							
34							
35							

灌丛层植物群落调查表

群落名称：山黄麻-粗糠柴-马樱丹　　样方面积：5 m × 5 m　　野外编号：59
调查时间：2017.01.06　14：20　　记录者：　　室内编号：

编号	植物名称	高度（cm）	冠径（cm）	物候期	生活力	盖度%	株数/丛树
1	粗糠柴	150	60	叶	2	25	3
2	九节	70	30	叶	2	20	1
3	黄牛木	60	20	叶	2	10	1
4	山黄皮	50	15	叶	2	10	1
5	异木患	55	10	叶	2	5	2
6							
7	破布叶	120	60	叶	2	20	2
8	马缨丹	30	16	花	1	25	1
9	斑鸠菊	12	8	花	2	15	1
10	毛柿	10	6	叶	1	5	2
11							
12	白楸	20	9	叶	2	5	1
13	山黄麻	150	14	叶	2	45	1
14							
15							
16							
17							
18							
19							
20							
21							
22							
23							
24							
25							
26							
27							
28							
29							
30							

草本层植物群落调查表

群落名称：斑茅-鬼针草　　样方面积 1 m × 1 m　　野外编号：59
调查时间：2017.01.06　14：20　　记录者：　　室内编号：

编号	植物名称	株高(cm)	盖度(%)	物候期	生活力	备注
1	鞘柄菝葜	40	30	叶	2	
2	飞机草	15	20	叶	1	
3						
4	锡叶藤	8	15	叶	2	
5	马交儿	10	10	叶	2	
6						
7	吐烟花	5	10	叶	2	
8	海南茄	20	30	叶	2	
9						
10	玉叶金花	15	10	叶	2	
11						
12	斑茅	80	60	花	2	
13	鬼针草	20	40	花	2	
14						
15						
16						
17						
18						
19						
20						
21						
22						
23						
24						
25						
26						
27						
28						
29						
30						

说明：物候期：花、叶、果
生活力：1　良好 2　一般　3　较差

总表

群落名称乔-灌-草优势种	构树-马缨丹-斑茅			野外编号(统一编号)	60
记录者		日期		室内编号	
样地面积		详细地点			
GPS 定位	N：19°55.121′<br>E：110°15.648′	海拔高度	86 m		
群落高度			群落的总盖度	80%	
主要层优势种	乔木层：<br>灌木层：<br>草本层：				
群落外貌特点	次生林				
小地形及样地周围环境描述	采石坑出，斑茅众多				
分层及各层的特点	乔木层	高度			
	灌木层	高度			
	草本层	高度			
	层间植物	高度			
		高度			
备注（之前的土地利用状况）	鲜重：0.12 kg				

说明：数据尽可能填写全面，没有填

乔木层植物群落调查表

群落名称：对叶榕-荔枝-构树　　样方面积：20 m × 20 m　　野外编号：60
调查时间：　　记录者：　　室内编号：

编号	植物名称	高度（m）	胸径（cm）	冠幅（m）	物候期	生活力	备注
1	荔枝	4	13	2×3	叶	3	
2	构树	6	15	2×3	叶	3	
3	对叶榕	2.5	12	3×2	叶	3	
4							
5							
6							
7							
8							
9							
10							
11							
12							
13							
14							
15							
16							
17							
18							
19							
20							
21							
22							
23							
24							
25							
26							
27							
28							
29							
30							
31							
32							
33							
34							
35							

灌丛层植物群落调查表

群落名称：斑茅　　　　样方面积：5 m × 5 m　　　　野外编号：60
调查时间：　　　　记录者：　　　　室内编号：

编号	植物名称	高度（cm）	冠径（cm）	物候期	生活力	盖度%	株数 / 丛树
1	簕欓花椒	30	30	叶	2	10	1
2	五色梅	50	30	叶花	2	15	1
3							
4	排钱树	60	40	叶	2	10	1
5	斑茅	70	80	叶	2	40	5
6							
7	猪肚木	60	25	花	2	20	2
8							
9	山黄麻	40	30	叶	2	20	2
10							
11							
12							
13							
14							
15							
16							
17							
18							
19							
20							
21							
22							
23							
24							
25							
26							
27							
28							
29							
30							

草本层植物群落调查表

群落名称：三点金-南美蟛蜞菊　　　　样方面积 1 m × 1 m　　　　野外编号：60
调查时间：　　　　记录者：　　　　室内编号：

编号	植物名称	株高(cm)	盖度(%)	物候期	生活力	备注
1	飞机草	15	10	叶	2	
2	含羞草	10	10	叶	2	
3	一年蓬	15	5	叶	2	
4						
5	丰花草	10	5	叶	2	
6	薇甘菊	8	5	叶	2	
7	蛇婆子	10	5	叶	2	
8						
9	墨苜蓿	2	10	叶花	2	
10	南美蟛蜞菊	5	30	叶花	2	
11						
12	野甘草	5	20	叶	2	
13	三点金	2	40	叶	2	
14						
15	红毛草	25	15	叶	2	
16	巴西含羞草	10	5	叶	2	
17						
18						
19						
20						
21						
22						
23						
24						
25						
26						
27						
28						
29						
30						

说明：物候期：花、叶、果
生活力：1　良好 2　一般　3　较差

总表

群落名称乔-灌-草优势种	龙眼-马缨丹-斑茅		野外编号(统一编号)	61
记录者		日期	2017.02.21 15：01	室内编号	
样地面积	20×20 m	详细地点		
GPS 定位	N：19°5.759′<br>E：110°10.506′	海拔高度	66 m	
群落高度		群落的总盖度	50%	
主要层优势种	乔木层：<br>灌木层：<br>草本层：			
群落外貌特点	次生林			
小地形及样地周围环境描述	植被大面积破坏，地表裸露			
分层及各层的特点	乔木层	高度		
	灌木层	高度		
	草本层	高度		
	层间植物	高度		
		高度		
备注（之前的土地利用状况）	土壤鲜重：0.10 kg			

说明：数据尽可能填写全面，没有填无

乔木层植物群落调查表

群落名称：龙眼　　样方面积：20 m × 20 m　　野外编号：61

调查时间：2017.02.21　15：01　　记录者：　　室内编号：

编号	植物名称	高度（m）	胸径（cm）	冠幅（m）	物候期	生活力	备注
1	龙眼	8	20	8×6	叶	1	
2							
3							
4							
5							
6							
7							
8							
9							
10							
11							
12							
13							
14							
15							
16							
17							
18							
19							
20							
21							
22							
23							
24							
25							
26							
27							
28							
29							
30							
31							
32							
33							
34							
35							

灌丛层植物群落调查表

群落名称：马樱丹-猪屎豆-地桃花　　样方面积：5 m × 5 m　　野外编号：61
调查时间：2017.02.21　15：01　　记录者：　　室内编号：

编号	植物名称	高度（cm）	冠径（cm）	物候期	生活力	盖度%	株数 / 丛树
1	猪屎豆	100	60	花	2	60	3
2							
3	马缨丹	120	80	花	2	70	2
4							
5	地桃花	110	120	花果	2	60	2
6							
7							
8							
9							
10							
11							
12							
13							
14							
15							
16							
17							
18							
19							
20							
21							
22							
23							
24							
25							
26							
27							
28							
29							
30							

草本层植物群落调查表

群落名称：斑茅-薇甘菊-蛇葡萄　　样方面积 1 m × 1 m　　野外编号：61
调查时间：2017.02.21　15：10　　记录者：　　室内编号：

编号	植物名称	株高(cm)	盖度(%)	物候期	生活力	备注
1	巴西含羞草	20	5	果	2	
2	薇甘菊	130	60	叶	2	
3						
4	羽芒菊	30	20	叶花	2	
5	假败酱	30	20	叶花	1	
6						
7	斑茅	230	80	叶	2	
8						
9	叶下珠	10	5	叶	2	
10	藿香蓟	20	5	叶花	2	
11						
12	蛇葡萄	40	60	叶	2	
13	巴西含羞草	60	20	叶花果	2	
14						
15	厚藤	30	10	叶	2	
16	红毛草	30	5	叶花果	2	
17						
18						
19						
20						
21						
22						
23						
24						
25						
26						
27						
28						
29						
30						

说明：物候期：花、叶、果
生活力：1　良好 2　一般　3　较差

总表

<table>
<tr><td>群落名称
乔-灌-草
优势种</td><td colspan="4">对叶榕-白饭树-鬼针草</td><td>野外编号
(统一编号)</td><td>62</td></tr>
<tr><td>记录者</td><td colspan="2"></td><td>日期</td><td></td><td>室内编号</td><td></td></tr>
<tr><td>样地面积</td><td colspan="2">20×20 m</td><td>详细地点</td><td colspan="3"></td></tr>
<tr><td>GPS 定位</td><td>N：19°54.710′
E：110°11.308′</td><td>海拔高度</td><td colspan="4">90 m</td></tr>
<tr><td>群落高度</td><td colspan="3"></td><td>群落的总盖度</td><td colspan="2">88%</td></tr>
<tr><td>主要层优势种</td><td colspan="6">乔木层：
灌木层：
草本层：</td></tr>
<tr><td>群落外貌特点</td><td colspan="6">较少乔木，杂草多</td></tr>
<tr><td>小地形及样地周围环境描述</td><td colspan="6">工地旁</td></tr>
<tr><td rowspan="5">分层及各层的特点</td><td>乔木层</td><td>高度</td><td colspan="4"></td></tr>
<tr><td>灌木层</td><td>高度</td><td colspan="4"></td></tr>
<tr><td>草本层</td><td>高度</td><td colspan="4"></td></tr>
<tr><td>层间植物</td><td>高度</td><td colspan="4"></td></tr>
<tr><td></td><td>高度</td><td colspan="2"></td><td></td><td></td></tr>
<tr><td>备注（之前的土地利用状况）</td><td colspan="6">0.12 kg</td></tr>
</table>

说明：数据尽可能填写全面，没有填无

乔木层植物群落调查表

群落名称：对叶榕　　样方面积：20 m × 20 m　　野外编号：62
调查时间：　　记录者：　　室内编号：

编号	植物名称	高度（m）	胸径（cm）	冠幅（m）	物候期	生活力	备注
1	对叶榕	7	6	4×5	叶果	1	
2	秋枫	6	9	6×5	叶	2	
3	对叶榕	6	5	4×5	叶果	1	
4	对叶榕	5	4	3×4	叶果	2	
5	对叶榕	6	7	5×4	叶果	1	
6	对叶榕	7	8	5×5	叶果	1	
7							
8							
9							
10							
11							
12							
13							
14							
15							
16							
17							
18							
19							
20							
21							
22							
23							
24							
25							
26							
27							
28							
29							
30							
31							
32							
33							
34							
35							

灌丛层植物群落调查表

群落名称：白饭树-马缨丹-龙血树　　样方面积：5 m × 5 m　　野外编号：62
调查时间：　　记录者：　　室内编号：

编号	植物名称	高度（cm）	冠径（cm）	物候期	生活力	盖度%	株数/丛树
1	倒吊笔	180	140	叶	2	50	1
2	鹊肾树	200	120	叶	2	50	1
3							
4	白饭树	200	150	叶	2	70	1
5	马缨丹	160	160	叶花	1	60	1
6	龙血树	300	150	叶	1	60	1
7							
8	潺槁木姜子	150	20	叶	2	40	2
9	牛筋果	140	60	叶	2	40	1
10	破布叶	170	40	叶	2	30	2
11	白藤	180	40	叶	2	10	2
12							
13							
14							
15							
16							
17							
18							
19							
20							
21							
22							
23							
24							
25							
26							
27							
28							
29							
30							

草本层植物群落调查表

群落名称：白花鬼针草-火炭母-飞机草　　样方面积 1 m × 1 m　　野外编号：62
调查时间：　　记录者：　　室内编号：

编号	植物名称	株高(cm)	盖度(%)	物候期	生活力	备注
1	龙珠果	5	60	叶	1	
2	火炭母	3	80	叶花果	1	
3						
4	酢浆草	3	20	叶	1	
5	凤尾蕨	30	5	叶	2	
6	白花鬼针草	120	90	叶花果	1	
7						
8	假蒟	30	60	叶	1	
9	海芋	60	40	叶	1	
10						
11	藿香蓟	30	10	叶花	1	
12	飞机草	160	80	叶花果	1	
13						
14	掌叶山猪菜	5	70	叶花	1	
15	斑茅	400	70	叶	3	
16						
17						
18						
19						
20						
21						
22						
23						
24						
25						
26						
27						
28						
29						
30						

说明：物候期：花、叶、果
生活力：1　良好 2　一般　3　较差

总表

群落名称<br>乔-灌-草<br>优势种	马占相思-破布叶-蔓生莠竹			野外编号(统一编号)	63
记录者		日期	2017.02.22 14：00	室内编号	
样地面积	20×20 m	详细地点			
GPS 定位	N：19°54.750′<br>E：110°11.540′	海拔高度	83m		
群落高度			群落的总盖度	99%	
主要层优势种	乔木层：<br>灌木层：<br>草本层：				
群落外貌特点	植物丰富，群落层次明显				
小地形及样地周围环境描述	乡村小路旁				
分层及各层的特点	乔木层	高度			
	灌木层	高度			
	草本层	高度			
	层间植物	高度			
		高度			
备注（之前的土地利用状况）	土壤鲜重：0.10 kg				

说明：数据尽可能填写全面，没有填无

乔木层植物群落调查表

群落名称：马占相思　　样方面积：20 m × 20 m　　野外编号：63
调查时间：2017.02.22　14：05　记录者：　　室内编号：

编号	植物名称	高度（m）	胸径（cm）	冠幅（m）	物候期	生活力	备注
1	荔枝	12	35	5×5	叶	1	
2	荔枝	15	50	5×5	叶	1	
3	荔枝	12	45	6×6	叶	1	
4	马占相思	12	40	3×3	叶	1	
5	马占相思	12	30	4×4	叶	1	
6	马占相思	10	25	2×2	叶	1	
7	马占相思	15	40	4×4	叶	1	
8	马占相思	10	25	3×3	叶	1	
9	苦楝	18	30	3×4	叶	1	
10	苦楝	18	35	4×3	叶	1	
11							
12							
13							
14							
15							
16							
17							
18							
19							
20							
21							
22							
23							
24							
25							
26							
27							
28							
29							
30							
31							
32							
33							
34							
35							

灌丛层植物群落调查表

群落名称：破布叶-毛柿　　样方面积：5 m × 5 m　　野外编号：63
调查时间：2017.02.22　14：00　　记录者：　　室内编号：

编号	植物名称	高度（cm）	冠径（cm）	物候期	生活力	盖度%	株数/丛树
1	毛柿	160	80	果	1	60	1
2	破布叶	180	160	叶	2	70	1
3							
4	白饭树	130	40	叶	2	20	2
5	两面针	170	60	叶	1	40	1
6							
7	越南悬钩子	160	40	叶	2	50	2
8	牛筋果	200	40	叶	1	50	1
9	华南省藤	180	30	叶果	2	40	1
10	大青	180	40	叶	2	20	1
11							
12							
13							
14							
15							
16							
17							
18							
19							
20							
21							
22							
23							
24							
25							
26							
27							
28							
29							
30							

草本层植物群落调查表

群落名称：蔓生莠竹-粪箕笃-蛇葡萄　　样方面积 1 m × 1 m　　野外编号：63
调查时间：2017.02.22　14：05　　记录者：　　室内编号：

编号	植物名称	株高(cm)	盖度(%)	物候期	生活力	备注
1	海芋	60	20	叶	1	
2	粪箕笃	100	85	叶	1	
3						
4	蔓生莠竹	40	80	叶	2	
5	假蒟	20	50	叶	1	
6	山牵牛	160	40	叶	1	
7						
8	乌蔹梅	40	40	叶	1	
9	蛇葡萄	50	75	叶	1	
10						
11	金腰箭	60	20	叶花果	2	
12	薇甘菊	170	60	叶	1	
13						
14	络石藤	2	40	叶	2	
15	蔓生莠竹	40	80	叶	1	
16						
17						
18						
19						
20						
21						
22						
23						
24						
25						
26						
27						
28						
29						
30						

说明：物候期：花、叶、果
生活力：1　良好 2　一般　3　较差

总表

<table>
<tr><td>群落名称
乔-灌-草
优势种</td><td colspan="3">乌墨-倒吊笔-吐烟花</td><td>野外编号
(统一编号)</td><td>64</td></tr>
<tr><td>记录者</td><td colspan="2"></td><td>日期</td><td></td><td>室内编号</td></tr>
<tr><td>样地面积</td><td colspan="2">20×20 m</td><td>详细地点</td><td colspan="2"></td></tr>
<tr><td>GPS 定位</td><td>N：19°54.771′
E：110°12.356′</td><td>海拔高度</td><td colspan="3">124 m</td></tr>
<tr><td>群落高度</td><td colspan="3"></td><td>群落的总盖度</td><td>95%</td></tr>
<tr><td>主要层优势种</td><td colspan="5">乔木层：
灌木层：
草本层：</td></tr>
<tr><td>群落外貌特点</td><td colspan="5">火山石众多，植被盖度大</td></tr>
<tr><td>小地形及样地周围环境描述</td><td colspan="5">林旁，风水林</td></tr>
<tr><td rowspan="5">分层及各层的特点</td><td>乔木层</td><td>高度</td><td colspan="3"></td></tr>
<tr><td>灌木层</td><td>高度</td><td colspan="3"></td></tr>
<tr><td>草本层</td><td>高度</td><td colspan="3"></td></tr>
<tr><td>层间植物</td><td>高度</td><td colspan="3"></td></tr>
<tr><td></td><td>高度</td><td></td><td></td><td></td></tr>
<tr><td>备注（之前的土地利用状况）</td><td colspan="5">0.10 kg</td></tr>
</table>

说明：数据尽可能填写全面，没有填无

乔木层植物群落调查表

群落名称：乌墨　　样方面积：20 m × 20 m　　野外编号：64
调查时间：　　记录者：　　室内编号：

编号	植物名称	高度（m）	胸径（cm）	冠幅（m）	物候期	生活力	备注
1	水仙柯	8	6	8×6	叶果	1	
2	秋枫	12	15	8×5	叶花	1	
3	乌墨	12	25	8×6	叶	1	
4	乌墨	10	15	6×7	叶	1	
5	乌墨	11	15	8×6	叶	1	
6	鱼尾葵	12	6	6×5	叶花	1	
7	榕树	12	25	10×12	叶	1	
8	毛八角枫	10	6	8×6	叶	1	
9							
10							
11							
12							
13							
14							
15							
16							
17							
18							
19							
20							
21							
22							
23							
24							
25							
26							
27							
28							
29							
30							
31							
32							
33							
34							
35							

灌丛层植物群落调查表

群落名称：八角枫-酒饼簕　　样方面积：5 m × 5 m　　野外编号：64
调查时间：　　记录者：　　室内编号：

编号	植物名称	高度（cm）	冠径（cm）	物候期	生活力	盖度%	株数/丛树
1	马缨丹	160	50	叶	1	40	1
2	破布叶	140	40	叶	2	30	1
3							
4	黄牛木	170	100	叶	2	40	1
5	毛柿	165	60	叶	2	40	1
6							
7	倒吊笔	200	60	叶	2	40	1
8	酒饼簕	150	70	叶	2	50	1
9	九节	140	40	叶	1	20	1
10	八角枫	200	160	叶	2	70	1
11	山黄皮	40	20	叶	1	10	1
12							
13							
14							
15							
16							
17							
18							
19							
20							
21							
22							
23							
24							
25							
26							
27							
28							
29							
30							

草本层植物群落调查表

群落名称：吐烟花-薇甘菊-飞机草　　样方面积 1 m × 1 m　　野外编号：64
调查时间：　　记录者：　　室内编号：

编号	植物名称	株高(cm)	盖度(%)	物候期	生活力	备注
1	吐烟花	3	80	叶	1	
2	海南茄	60	5	叶	1	
3						
4	蔓生莠竹	20	10	叶	2	
5	薇甘菊	190	70	叶	1	
6						
7	山牵牛	170	40	叶	1	
8	假蒟	20	60	叶	1	
9						
10	飞机草	160	70	叶	2	
11	吐烟花	5	80	叶	1	
12						
13	翼茎白粉藤	180	60	叶	1	
14	淡竹叶	15	60	叶	1	
15						
16						
17						
18						
19						
20						
21						
22						
23						
24						
25						
26						
27						
28						
29						
30						

说明：物候期：花、叶、果
生活力：1　良好 2　一般　3　较差

总表

群落名称<br>乔-灌-草<br>优势种	高山榕-毛柿-吐烟花			野外编号<br>(统一编号)	65
记录者		日期		室内编号	
样地面积	20 m×20 m	详细地点			
GPS 定位	N：19°54.770′<br>E：110 °12.906′	海拔高度	121 m		
群落高度		群落的总盖度	90%		
主要层优势种	乔木层：<br>灌木层：<br>草本层：				
群落外貌特点	次生林				
小地形及样地周围环境描述	小道旁火山石众多杂草纵生				
分层及各层的特点	乔木层	高度			
	灌木层	高度			
	草本层	高度			
	层间植物	高度			
		高度			
备注（之前的土地利用状况）	鲜重：0.10 kg				

说明：数据尽可能填写全面，没有填无

乔木层植物群落调查表

群落名称：毛柿　　样方面积：20 m × 20 m　　野外编号：65

调查时间：　2017.02.16　.10：00　记录者：　　室内编号：65

编号	植物名称	高度（m）	胸径（cm）	冠幅（m）	物候期	生活力	备注
1	毛柿	3	15	4×3	叶	2	
2							
3	凤凰木	7	10	2×3	叶	2	
4							
5							
6							
7							
8							
9							
10							
11							
12							
13							
14							
15							
16							
17							
18							
19							
20							
21							
22							
23							
24							
25							
26							
27							
28							
29							
30							
31							
32							
33							
34							
35							

灌丛层植物群落调查表

群落名称：毛柿　　样方面积：1 m × 1 m　　野外编号：65

调查时间：2017.02.16.　10：00　　记录者：　　室内编号：

编号	植物名称	高度（cm）	冠径（cm）	物候期	生活力	盖度%	株数/丛树
1	毛柿	190	120	果	2	40	
2	猪肚木	160	60	叶	2	30	
3	雀梅	170	80	叶	1	30	
4	假黄皮	170	50	叶	2	20	
5	马缨丹	200	100	花	1	10	
6							
7	五月茶	150	40	叶	2	10	
8	酒饼簕	100	60	叶	1	10	
9	黑面神	170	50	叶	2	20	
10							
11							
12							
13							
14							
15							
16							
17							
18							
19							
20							
21							
22							
23							
24							
25							
26							
27							
28							
29							
30							

草本层植物群落调查表

群落名称：斑茅-吐烟花　　样方面积 1 m × 1 m　　野外编号：65

调查时间：　　记录者：　　室内编号：

编号	植物名称	株高(cm)	盖度(%)	物候期	生活力	备注
1	吐烟花	10	60	叶	2	
2	肾蕨	20	30	叶	1	
3						
4	吐烟花	5	70	叶	1	
5						
6	野菊	210	60	叶	2	
7	薇甘菊	150	40	叶花	1	
8	斑茅	300	90	叶花	3	
9						
10	心叶黄花稔	20	30	叶	2	
11						
12						
13						
14						
15						
16						
17						
18						
19						
20						
21						
22						
23						
24						
25						
26						
27						
28						
29						
30						

说明：物候期：花、叶、果

生活力：1　良好 2　一般　3　较差

总表

群落名称乔-灌-草优势种	毛八角枫-细基丸-吐烟花			野外编号(统一编号)	66
记录者		日期	2017.02.17 9：45	室内编号	
样地面积		详细地点			
GPS 定位	N：19°54.693′<br>E：110°13.546′	海拔高度	139 m		
群落高度		群落的总盖度	95%		
主要层优势种	乔木层：<br>灌木层：<br>草本层：				
群落外貌特点	次生林				
小地形及样地周围环境描述	火山石众多				
分层及各层的特点	乔木层	高度			
	灌木层	高度			
	草本层	高度			
	层间植物	高度			
		高度			
备注（之前的土地利用状况）	鲜重：0.10 kg				

说明：数据尽可能填写全面，没有填无

乔木层植物群落调查表

群落名称：水仙柯-毛八角枫　　样方面积：　　野外编号：66
调查时间：　2017.02.17　9：45　　记录者：　　室内编号：

编号	植物名称	高度（m）	胸径（cm）	冠幅（m）	物候期	生活力	备注
1	水仙柯	6	8	2×3	叶果	2	
2	水仙柯	7	12	3×5	叶果	1	
3							
4	毛八角枫	8	20	4×4	休眠	3	
5	毛八角枫	8	25	5×7	叶	3	
6	毛八角枫	9	20	3×4	休眠	3	
7							
8	水仙柯	4	12	4×5	叶果	1	
9	水仙柯	5	15	3×4	果	1	
10							
11	簕欓花椒	3	8	3×2	叶	1	
12							
13							
14							
15							
16							
17							
18							
19							
20							
21							
22							
23							
24							
25							
26							
27							
28							
29							
30							
31							
32							
33							
34							
35							

灌丛层植物群落调查表

群落名称：白藤　　样方面积：5 m × 5 m　　野外编号：66
调查时间：2017.02.17　9：55　　记录者：　　室内编号：

编号	植物名称	高度（cm）	冠径（cm）	物候期	生活力	盖度%	株数 / 丛树
1	潺槁木姜子	120	10	叶	2	5	
2	九节	80	40	叶	1	10	
3							
4	细基丸	120	80	叶	2	40	
5							
6	土沉香	200	120	叶	2	40	
7	白藤	180	80	叶	1	60	
8							
9							
10							
11							
12							
13							
14							
15							
16							
17							
18							
19							
20							
21							
22							
23							
24							
25							
26							
27							
28							
29							
30							

草本层植物群落调查表

群落名称：吐烟花-海芋　　样方面积 1 m × 1 m　　野外编号：66
调查时间：2017.02.17　10：00　　记录者：　　室内编号：

编号	植物名称	株高(cm)	盖度(%)	物候期	生活力	备注
1	吐烟花	15	95	叶	1	
2	节毛乌蔹梅	220	40	叶	1	
3	粪箕笃	180	50	叶	1	
4	凤尾葵	30	20	叶	1	
5	海芋	120	80	叶花	1	
6	野葛	180	60	叶	1	
7						
8						
9						
10						
11						
12						
13						
14						
15						
16						
17						
18						
19						
20						
21						
22						
23						
24						
25						
26						
27						
28						
29						
30						

说明：物候期：花、叶、果
生活力：1　良好 2　一般　3　较差

总表

群落名称<br>乔-灌-草<br>优势种	荔枝-破布叶-吐烟花			野外编号<br>(统一编号)	67
记录者		日期	2017.2.17<br>9：15	室内编号	
样地面积	20×20 m	详细地点			
GPS 定位	N：19°54.632′<br>E：110°14.063′	海拔高度			
群落高度			群落的总盖度	90%	
主要层优势种	乔木层：<br>灌木层：<br>草本层：				
群落外貌特点	人工林				
小地形及样地周围环境描述	火山岩果园				
分层及各层的特点	乔木层	高度			
	灌木层	高度			
	草本层	高度			
	层间植物	高度			
		高度			
备注（之前的土地利用状况）	鲜重：0.10 kg				

说明：数据尽可能填写全面，没有填无

乔木层植物群落调查表

群落名称：荔枝-菠萝蜜-毛八角枫　　样方面积：　　野外编号：67

调查时间：　2017.02.17　9：15　　记录者：　　室内编号：

编号	植物名称	高度（m）	胸径（cm）	冠幅（m）	物候期	生活力	备注
1	荔枝	8	60	8×10	叶	1	
2	荔枝	9	70	9×10	叶	1	
3	菠萝蜜	8	20	4×5	叶	1	
4	菠萝蜜	8	15	4×5	果	1	
5	木棉	10	30	5×6	休眠	3	
6	毛八角枫	6	12	4×3	休眠	3	
7	黄皮	2	5	3×5	叶	1	
8	木瓜	4	8	2×2	果	2	
9	桉树	7	15	1×1	休眠	3	
10	毛八角枫	8	15	4×5	休眠	3	
11							
12							
13							
14							
15							
16							
17							
18							
19							
20							
21							
22							
23							
24							
25							
26							
27							
28							
29							
30							
31							
32							
33							
34							
35							

灌丛层植物群落调查表

群落名称：破布叶　　样方面积：1 m × 1 m　　野外编号：67
调查时间：2017.02.17　9：28　　记录者：　　室内编号：

编号	植物名称	高度（cm）	冠径（cm）	物候期	生活力	盖度%	株数/丛树
1	九节	30	20	叶	1	10	
2	黑面神	40	30	叶	1	20	
3	细基丸	60	40	叶	2	20	
4							
5	白藤	40	40	叶	2	30	
6	大青	80	40	叶	2	20	
7	毛柿	60	50	叶	2	20	
8	胡颓子	80	60	叶	2	30	
9							
10	山石榴	70	40	叶	1	20	
11	鸦胆子	100	40	叶	1	10	
12	黄牛木	80	20	叶	1	5	
13	破布叶	220	150	叶	1	40	
14							
15							
16							
17							
18							
19							
20							
21							
22							
23							
24							
25							
26							
27							
28							
29							
30							

草本层植物群落调查表

群落名称：吐烟花-金腰箭　　样方面积 1 m × 1 m　　野外编号：67
调查时间：2017.02.17　9：28　　记录者：　　室内编号：

编号	植物名称	株高(cm)	盖度(%)	物候期	生活力	备注
1	吐烟花	15	80	叶	1	
2	扭肚藤	80	20	叶	1	
3	凤尾葵	20	20	叶	1	
4	吐烟花	5	60	叶	1	
5	翼茎白粉藤	20	20	叶	2	
6	白粉藤	5	40	叶	2	
7	革命草	40	10	叶	1	
8	黄鹌菜	25	5	叶花	1	
9	一点红	15	5	叶花	1	
10	鬼针草	25	15	叶花果	2	
11	金腰箭	80	60	叶花	2	
12	威灵仙	15	20	叶	1	
13						
14						
15						
16						
17						
18						
19						
20						
21						
22						
23						
24						
25						
26						
27						
28						
29						
30						

说明：物候期：花、叶、果
生活力：1　良好 2　一般　3　较差

总表

群落名称<br>乔-灌-草<br>优势种	荔枝-油茶-吐烟花			野外编号<br>(统一编号)	68
记录者		日期	2017.02.17<br>8：45	室内编号	
样地面积	20×20 m	详细地点			
GPS 定位	N：19°54.704′<br>E：110°14.403′	海拔高度	131 m		
群落高度		群落的总盖度	95%		
主要层优势种	乔木层：<br>灌木层：<br>草本层：				
群落外貌特点	人工林				
小地形及样地周围环境描述	火山石众多				
分层及各层的特点	乔木层	高度			
	灌木层	高度			
	草本层	高度			
	层间植物	高度			
		高度			
备注（之前的土地利用状况）	鲜重：0.10 kg				

说明：数据尽可能填写全面，没有填无

乔木层植物群落调查表

群落名称：荔枝　　样方面积：　　野外编号：68

调查时间：2017.02.17　8：45　　记录者：　　室内编号：

编号	植物名称	高度（m）	胸径（cm）	冠幅（m）	物候期	生活力	备注
1	菠萝蜜	9	20	4×5	叶	1	
2	菠萝蜜	7.5	15	4×5	叶	1	
3	荔枝	10	80	6×10	叶	1	
4	荔枝	10	60	7×10	叶	1	
5	荔枝	9	40	6×10	叶	1	
6	银合欢	7	20	4×6	叶	2	
7	银合欢	6	20	4×6	叶	2	
8	银合欢	6	15	3×3	叶	2	
9	毛八角枫	10	15	3×3	休眠	3	
10	毛八角枫	7	10	3×3	休眠	3	
11	毛八角枫	9	15	3×3	休眠	3	
12							
13							
14							
15							
16							
17							
18							
19							
20							
21							
22							
23							
24							
25							
26							
27							
28							
29							
30							
31							
32							
33							
34							
35							

灌丛层植物群落调查表

群落名称：油茶-毛柿　样方面积：5 m × 5 m　野外编号：68
调查时间：2017.02.17 8：45　记录者：　室内编号：

编号	植物名称	高度（cm）	冠径（cm）	物候期	生活力	盖度%	株数 / 丛树
1	越南悬钩子	120	60	叶	1	40	3
2	九节	130	80	叶	2	30	1
3	酒饼簕	60	40	叶	2	20	1
4	破布叶	80	60	叶	2	40	1
5	胡颓子	120	60	叶	1	30	1
6	黑面神	80	40	叶	1	20	1
7	油茶	200	130	叶	1	80	1
8	毛柿	150	80	叶	1	50	1
9	鲫鱼胆	150	60	叶花	1	30	1
10							
11							
12							
13							
14							
15							
16							
17							
18							
19							
20							
21							
22							
23							
24							
25							
26							
27							
28							
29							
30							

草本层植物群落调查表

群落名称：吐烟花-三点金-海芋　样方面积 1 m × 1 m　野外编号：68
调查时间：2017.02.17　8：57　记录者：　室内编号：

编号	植物名称	株高(cm)	盖度(%)	物候期	生活力	备注
1	鸭跖草	20	40	叶	1	
2	三点金	5	60	叶	1	
3	微甘菊	15	25	叶	1	
4	飞机草	25	20	叶	1	
5	黄鹌菜	15	10	叶花果	2	
6	丰花草	15	10	叶花果	2	
7	藿香蓟	15	20	叶花	2	
8	一年蓬	20	10	叶	1	
9	鬼针草	25	40	叶花	1	
10	吐烟花	15	90	叶	1	
11	微白菊	20	20	叶	1	
12	海芋	60	60	叶	1	
13	酢浆草	5	40	叶	1	
14	铁草鞋	300	50	叶	1	
15	鞘柄菝葜	180	20	叶	1	
16						
17						
18						
19						
20						
21						
22						
23						
24						
25						
26						
27						
28						
29						
30						

说明：物候期：花、叶、果
生活力：1　良好 2 一般 3 较差

总表

<table>
<tr><td>群落名称
乔-灌-草
优势种</td><td colspan="4">菠萝蜜-海南菜豆树-飞机草</td><td>野外编号
(统一编号)</td><td colspan="2">69</td></tr>
<tr><td>记录者</td><td colspan="2"></td><td>日期</td><td></td><td>室内编号</td><td colspan="2"></td></tr>
<tr><td>样地面积</td><td colspan="2"></td><td>详细地点</td><td colspan="4"></td></tr>
<tr><td>GPS 定位</td><td>N：19°54.820′
E：110°15.145′</td><td>海拔高度</td><td colspan="5">88 m</td></tr>
<tr><td>群落高度</td><td colspan="3"></td><td>群落的总盖度</td><td colspan="3">70%</td></tr>
<tr><td>主要层优势种</td><td colspan="7">乔木层：
灌木层：
草本层：</td></tr>
<tr><td>群落外貌特点</td><td colspan="7">人工林</td></tr>
<tr><td>小地形及样地周围环境描述</td><td colspan="7">周围是果园，植被较多</td></tr>
<tr><td rowspan="5">分层及各层的特点</td><td>乔木层</td><td>高度</td><td colspan="5"></td></tr>
<tr><td>灌木层</td><td>高度</td><td colspan="5"></td></tr>
<tr><td>草本层</td><td>高度</td><td colspan="5"></td></tr>
<tr><td>层间植物</td><td>高度</td><td colspan="5"></td></tr>
<tr><td></td><td>高度</td><td colspan="2"></td><td></td><td colspan="2"></td></tr>
<tr><td>备注（之前的土地利用状况）</td><td colspan="7">鲜重：0.12 kg</td></tr>
</table>

说明：数据尽可能填写全面，没有填无

乔木层植物群落调查表

群落名称：黄皮-荔枝　样方面积：20 m × 20 m　野外编号：69
调查时间：　记录者：　室内编号：

编号	植物名称	高度（m）	胸径（cm）	冠幅（m）	物候期	生活力	备注
1	黄皮	4	12	2×2	叶	2	
2	黄皮	4	12	3×2	叶	2	
3	黄皮	4	10	3×2	叶	2	
4	黄皮	4	10	3×3	叶	2	
5	荔枝	3.5	12	3×3	叶	2	
6	荔枝	4	13	3×3	叶	2	
7	荔枝	3.5	12	3×3	叶	2	
8	莲雾	4.5	12	3×3	叶	2	
9	莲雾	4	13	3×2	叶	2	
10	菠萝蜜	10	35	4×5	果	2	
11	菠萝蜜	9	30	5×4	果	2	
12	菠萝蜜	9.5	32	5×5	果	2	
13	柚子	4	10	3×2	叶	2	
14	柚子	4	10	3×2	叶	2	
15	柚子	4	10	3×2	叶	2	
16	黄皮	4	10	3×2	叶	2	
17	荔枝	6.5	26	4×5	叶	2	
18	番石榴	3.5	13	3×2	叶	2	
19							
20							
21							
22							
23							
24							
25							
26							
27							
28							
29							
30							
31							
32							
33							
34							
35							

灌丛层植物群落调查表

群落名称：马樱丹-海南菜豆树　　样方面积：5 m × 5 m　　野外编号：69
调查时间：　　记录者：　　室内编号：

编号	植物名称	高度（cm）	冠径（cm）	物候期	生活力	盖度%	株数 / 丛树
1	马缨丹	40	30	花	2	30	1
2							
3	斑茅	60	10	叶	2	5	2
4	海南菜豆树	50	15	叶	2	10	2
5							
6	海南菜豆树	55	20	叶	2	10	1
7							
8							
9							
10							
11							
12							
13							
14							
15							
16							
17							
18							
19							
20							
21							
22							
23							
24							
25							
26							
27							
28							
29							
30							

草本层植物群落调查表

群落名称：荨麻-薇甘菊-飞机草　　样方面积 1 m × 1 m　　野外编号：69
调查时间：　　记录者：　　室内编号：

编号	植物名称	株高(cm)	盖度(%)	物候期	生活力	备注
1	薇甘菊	12	30	花	2	
2	飞机草	15	30	花	2	
3	水茄	15	10	叶	2	
4	丰花草	15	20	花	2	
5						
6	眼树莲	5	20	叶	2	
7	刺蒴麻	14	10	花	2	
8	梵天花	15	20	花	2	
9	假败酱	10	10	花	2	
10						
11	藿香蓟	20	20	花	2	
12	厚叶崖爬藤	5	20	叶	2	
13	黄花稔	15	20	花	2	
14	少花龙葵	10	20	花果	2	
15						
16	毒瓜	35	10	果	2	
17	丝瓜	40	20	果	2	
18	鬼针草	20	10	花	2	
19	火炭母	20	20	叶	2	
20	金腰箭	15	10	叶	2	
21						
22	芭蕉	12	6	叶	2	
23	荨麻	10	40	叶	2	
24	银胶菊	15	10	叶	2	
25	苋	10	15	叶	2	
26						
27						
28						
29						
30						

说明：物候期：花、叶、果
生活力：1　良好 2 一般 3 较差

总表

群落名称<br>乔-灌-草<br>优势种	荔枝-九节-菠萝			野外编号<br>(统一编号)	70
记录者		日期		室内编号	
样地面积		详细地点			
GPS 定位	N：19°54.669′<br>E：110°15.500′	海拔高度	85 m		
群落高度			群落的总盖度	80%	
主要层优势种	乔木层：<br>灌木层：<br>草本层：				
群落外貌特点	人工林				
小地形及样地周围环境描述	果园周围				
分层及各层的特点	乔木层	高度			
	灌木层	高度			
	草本层	高度			
	层间植物	高度			
		高度			
备注（之前的土地利用状况）	鲜重 0.1 kg				

说明：数据尽可能填写全面，没有填无

乔木层植物群落调查表

群落名称：荔枝-菠萝蜜　　样方面积：20 m × 20 m　　野外编号：70
调查时间：　　记录者：　　室内编号：

编号	植物名称	高度（m）	胸径（cm）	冠幅（m）	物候期	生活力	备注
1	荔枝	10	60	7×6	叶	1	
2	樫木	10	30	5×5	叶	2	
3	荔枝	7	43	5×5	叶	2	
4	荔枝	7	35	5×4	叶	2	
5	荔枝	7	38	5×4	叶	2	
6	菠萝蜜	8	32	5×4	叶	2	
7	降香黄檀	6	15	3×3	叶	2	
8	荔枝	6	10	3×2	叶	2	
9	荔枝	6	18	3×3	叶	2	
10	黄皮	7	19	3×3	叶	2	
11	黄皮	6	19	3×3	叶	2	
12	黄皮	6	18	3×3	叶	2	
13	荔枝	6	18	3×3	叶	2	
14	荔枝	6	16	3×3	叶	2	
15	荔枝	3	10	3×2	叶	2	
16	荔枝	3	10	3×2	叶	2	
17	荔枝	3	8	3×2	叶	2	
18	菠萝蜜	5	13	3×2	果	2	
19	菠萝蜜	5	15	2×3	果	2	
20	菠萝蜜	5	16	2×3	叶	2	
21	菠萝蜜	5	15	2×3	叶	2	
22							
23							
24							
25							
26							
27							
28							
29							
30							
31							
32							
33							
34							
35							

灌丛层植物群落调查表

群落名称：九节-鸦胆子　　样方面积：5 m × 5 m　　野外编号：70
调查时间：　　记录者：　　室内编号：

编号	植物名称	高度（cm）	冠径（cm）	物候期	生活力	盖度%	株数 / 丛树
1	九节	30	18	叶	2	20	2
2	酒饼簕	15	5	叶	2	10	1
3							
4	三桠苦	10	5	叶	2	10	2
5	鸦胆子	30	19	叶	2	15	1
6							
7	黄牛木	16	10	叶	2	10	1
8	鲫鱼胆	80	10	叶	2	10	2
9							
10							
11							
12							
13							
14							
15							
16							
17							
18							
19							
20							
21							
22							
23							
24							
25							
26							
27							
28							
29							
30							

草本层植物群落调查表

群落名称：菠萝-波罗蜜　　样方面积 1 m × 1 m　　野外编号：70
调查时间：　　记录者：　　室内编号：

编号	植物名称	株高(cm)	盖度(%)	物候期	生活力	备注
1	山黄皮	13	15	叶	2	
2	吐烟花	8	30	叶	2	
3	菠萝	25	60	叶	2	
4	菠萝蜜	20	40	叶	2	
5						
6	肾蕨	10	6	叶	2	
7	贴生石韦	5	5	叶	2	
8	马交儿	15	5	叶	2	
9	飞机草	15	20	叶	2	
10						
11	夜香牛	15	20	花	2	
12	藿香蓟	10	20	花	2	
13	金腰箭	15	20	叶	2	
14						
15	井栏边草	16	10	叶	2	
16	粪箕笃	10	5	叶	2	
17						
18	土人参	20	5	叶	2	
19	毛柿	6	10	叶	2	
20	华南忍冬	18	5	叶	2	
21						
22						
23						
24						
25						
26						
27						
28						
29						
30						

说明：物候期：花、叶、果
生活力：1　良好 2　一般　3　较差

总表

群落名称<br>乔-灌-草<br>优势种	簕竹-牛筋果-斑茅			野外编号<br>(统一编号)	71
记录者		日期	2017.02.21<br>14：20	室内编号	
样地面积	20×20 m	详细地点			
GPS 定位	N：19°54.266′<br>E：110°10.754′	海拔高度	68 m		
群落高度		群落的总盖度	89%		
主要层优势种	乔木层：<br>灌木层：<br>草本层：				
群落外貌特点	荒野				
小地形及样地周围环境描述	杂草、杂木多，有个竹林				
分层及各层的特点	乔木层	高度			
	灌木层	高度			
	草本层	高度			
	层间植物	高度			
		高度			
备注（之前的土地利用状况）	土壤鲜重：0.10 kg				

说明：数据尽可能填写全面，没有填无

乔木层植物群落调查表

群落名称：簕竹　　样方面积：20 m × 20 m　　野外编号：71

调查时间：2017.02.21　14：20　　记录者：　　室内编号：

编号	植物名称	高度（m）	胸径（cm）	冠幅（m）	物候期	生活力	备注
1	簕竹	12	4	1×1	叶	1	
2	簕竹	12	5	2×2	叶	1	
3	簕竹	10	5	1×2	叶	1	
4	簕竹	13	5	1×1	叶	1	
5	簕竹	12	5	2×2	叶	1	
6	簕竹	9	4	1×1	叶	1	
7							
8							
9							
10							
11							
12							
13							
14							
15							
16							
17							
18							
19							
20							
21							
22							
23							
24							
25							
26							
27							
28							
29							
30							
31							
32							
33							
34							
35							

灌丛层植物群落调查表

群落名称：破布叶-牛筋果-九节　　样方面积：5 m × 5 m　　野外编号：71
调查时间：2017.02.21　14：20　　记录者：　　室内编号：

编号	植物名称	高度（cm）	冠径（cm）	物候期	生活力	盖度%	株数 / 丛树
1	牛筋果	170	100	叶花	2	60	1
2	倒吊笔	180	70	叶	2	50	1
3							
4	裸实	100	40	叶	1	10	2
5	雀梅	160	60	果	2	15	3
6	土蜜树	120	60	叶	2	10	1
7							
8	鸦胆子	170	100	叶	2	50	1
9	九节	170	100	叶	2	60	1
10	破布叶	160	120	叶	2	70	1
11	黑面神	150	70	叶	2	40	1
12							
13							
14							
15							
16							
17							
18							
19							
20							
21							
22							
23							
24							
25							
26							
27							
28							
29							
30							

草本层植物群落调查表

群落名称：斑茅-蔓生莠竹　　样方面积 1 m × 1 m　　野外编号：71
调查时间：2017.02.21　14：20　　记录者：　　室内编号：

编号	植物名称	株高(cm)	盖度(%)	物候期	生活力	备注
1	一年蓬	20	15	叶	1	
2	金腰箭	20	10	叶花	1	
3						
4	蔓生莠竹	60	70	叶	3	
5	一年蓬	100	5	叶花	1	
6	桤叶黄花棯	15	5	叶花	1	
7						
8	猪屎豆	120	10	叶花	2	
9	飞机草	70	20	叶	1	
10						
11	羽芒菊	5	5	叶花	2	
12	飞扬草	15	5	叶花果	2	
13	地毯草	80	10	叶花果	3	
14						
15	斑茅	400	70	叶果	2	
16	链荚豆	5	20	叶	2	
17	半枝莲	5	20	叶	2	
18						
19						
20						
21						
22						
23						
24						
25						
26						
27						
28						
29						
30						

说明：物候期：花、叶、果
生活力：1　良好 2　一般　3　较差

总表

群落名称<br>乔-灌-草<br>优势种	马占相思-两面针-蔓生莠竹			野外编号<br>(统一编号)	72
记录者		日期	2017.02.21<br>13：43	室内编号	
样地面积	20×20 m	详细地点			
GPS 定位	N：19°54.283′<br>E：110°11.214′	海拔高度	77 m		
群落高度			群落的总盖度	89%	
主要层优势种	乔木层：<br>灌木层：<br>草本层：				
群落外貌特点	人工林				
小地形及样地周围环境描述	农田边 河边				
分层及各层的特点	乔木层	高度			
	灌木层	高度			
	草本层	高度			
	层间植物	高度			
		高度			
备注（之前的土地利用状况）	土壤鲜重：0.12 kg				

说明：数据尽可能填写全面，没有填无

乔木层植物群落调查表

群落名称：马占相思　　样方面积：20 m × 20 m　　野外编号：72

调查时间：2017.02.21　13：43　　记录者：　　室内编号：

编号	植物名称	高度（m）	胸径（cm）	冠幅（m）	物候期	生活力	备注
1	马占相思	15	25	5×6	叶	2	
2	马占相思	15	30	5×5	叶	1	
3	马占相思	15	20	4×4	叶	1	
4	马占相思	12	20	4×3	叶	1	
5	马占相思	15	35	5×5	叶	1	
6	马占相思	15	20	2×2	叶		
7	马占相思	12	35	2×2	叶	1	
8	马占相思	15	40	4×4	叶	1	
9	马占相思	15	35	4×4	叶	1	
10	马占相思	15	30	4×3	叶	1	
11	马占相思	12	30	3×3	叶	1	
12	马占相思	16	30	3×3	叶	1	
13	马占相思	15	20	4×4	叶	1	
14	马占相思	15	25	3×3	叶	1	
15	马占相思	15	30	4×4	叶	1	
16	马占相思	12	20	1×1	叶	2	
17	马占相思	14	30	2×2	叶	2	
18	马占相思	13	30	2×2	叶	2	
19	马占相思	15	30	3×3	叶	2	
20							
21							
22							
23							
24							
25							
26							
27							
28							
29							
30							
31							
32							
33							
34							
35							

灌丛层植物群落调查表

群落名称：马樱丹-鹊肾树　　样方面积：5 m × 5 m　　野外编号：72
调查时间：2017.02.21　　13：43　　记录者：　　室内编号：

编号	植物名称	高度（cm）	冠径（cm）	物候期	生活力	盖度%	株数 / 丛树
1	假杜鹃	30	30	花	1	20	3
2	马缨丹	160	120	花	1	60	2
3							
4	对叶榕	40	60	叶	1	20	2
5	潺槁木姜子	30	20	叶	2	20	1
6	鹊肾树	170	50	叶	1	60	1
7							
8	细基丸	40	20	叶	2	5	1
9	两面针	10	100	叶	1	20	1
10	大管	150	40	叶　花	1	10	1
11	牛筋果	160	20	叶	1	20	1
12							
13							
14							
15							
16							
17							
18							
19							
20							
21							
22							
23							
24							
25							
26							
27							
28							
29							
30							

草本层植物群落调查表

群落名称：蔓生莠竹-海芋-薇甘菊　　样方面积 1 m × 1 m　　野外编号：72
调查时间：2017.02.21　　13：53　　记录者：　　室内编号：

编号	植物名称	株高(cm)	盖度(%)	物候期	生活力	备注
1	革命菜	40	10	花	2	
2	少花龙葵	20	60	花果	1	
3						
4	金腰箭	40	5	花	2	
5	鸭趾草	5	50	叶	1	
6						
7	薇甘菊	5	70	叶	2	
8	斑茅	400	70	叶花	2	
9	蛇葡萄	30	40	叶	2	
10						
11	酢浆草	20	40	叶花	2	
12	叶下珠	5	5	叶	2	
13	丰花草	10	10	叶花	1	
14						
15	火炭母	20	20	叶	2	
16	蔓生莠竹	30	80	叶	2	
17	水茄	50	50	叶	2	
18	海芋	140	80	叶花果	1	
19						
20						
21						
22						
23						
24						
25						
26						
27						
28						
29						
30						

说明：物候期：花、叶、果
生活力：1　良好 2　一般 3　较差

总表

群落名称<br>乔-灌-草<br>优势种	秋枫-鹊肾树-斑茅			野外编号(统一编号)	73
记录者		日期	2017.02.21<br>13：20	室内编号	
样地面积	20×20 m	详细地点			
GPS 定位	N：19°54.276′<br>E：110°11.771′	海拔高度	96 m		
群落高度		群落的总盖度	90%		
主要层优势种	乔木层：<br>灌木层：<br>草本层：				
群落外貌特点	少乔木，多草本				
小地形及样地周围环境描述	荒废土地 斑茅多				
分层及各层的特点	乔木层	高度			
	灌木层	高度			
	草本层	高度			
	层间植物	高度			
		高度			
备注（之前的土地利用状况）	土壤鲜重：0.14 kg				

说明：数据尽可能填写全面，没有填无

乔木层植物群落调查表

群落名称：秋枫-番石榴　　样方面积：20 m × 20 m　　野外编号：73
调查时间：2017.02.21　13：20　　记录者：　　室内编号：

编号	植物名称	高度（m）	胸径（cm）	冠幅（m）	物候期	生活力	备注
1	秋枫	12	45	8×6	叶	2	
2	番石榴	5	4	2×3	叶	2	
3							
4							
5							
6							
7							
8							
9							
10							
11							
12							
13							
14							
15							
16							
17							
18							
19							
20							
21							
22							
23							
24							
25							
26							
27							
28							
29							
30							
31							
32							
33							
34							
35							

灌丛层植物群落调查表

群落名称：鹊肾树-黑面神-酒饼簕　　样方面积：5 m × 5 m　　野外编号：73
调查时间：2017.02.21　13：25　　记录者：　　室内编号：

编号	植物名称	高度（cm）	冠径（cm）	物候期	生活力	盖度%	株数/丛树
1	黄牛木	140	50	叶	1	20	1
2	鹊肾树	140	120	叶	2	80	2
3							
4	鹊肾树	160	130	叶	1	80	2
5	马缨丹	130	60	叶花	1	50	1
6							
7	酒饼簕	190	100	叶	2	60	1
8	黑面神	200	120	叶果	2	70	1
9	破布叶	170	100	叶	2	40	1
10							
11							
12							
13							
14							
15							
16							
17							
18							
19							
20							
21							
22							
23							
24							
25							
26							
27							
28							
29							
30							

草本层植物群落调查表

群落名称：蔓生莠竹-斑茅　　样方面积 1 m × 1 m　　野外编号：73
调查时间：2017.02.21　13：20　　记录者：　　室内编号：

编号	植物名称	株高(cm)	盖度(%)	物候期	生活力	备注
1	斑茅	500	70	花果	3	
2	藿香蓟	60	30	花果	2	
3						
4	蔓生莠竹	30	80	叶	2	
5	夜香牛	25	20	叶花	2	
6						
7	薇甘菊	30	40	叶	2	
8	鸭趾草	10	20	叶	2	
9	蛇葡萄	30	40	叶	2	
10						
11	珍珠菜	8	20	叶花	1	
12	鸡屎藤	20	40	叶	2	
13	海芋	130	20	叶花	2	
14						
15	丰花草	15	10	叶花	2	
16	火炭母	20	20	叶花	1	
17						
18						
19						
20						
21						
22						
23						
24						
25						
26						
27						
28						
29						
30						

说明：物候期：花、叶、果
生活力：1　良好 2　一般 3　较差

总表

群落名称乔-灌-草优势种	乌墨-鹊肾树-斑茅		野外编号（统一编号）	74
记录者		日期 2017.02.17	室内编号	
样地面积		详细地点		
GPS 定位	N：19 °54.108′<br>E：110°12.134′	海拔高度	116 m	
群落高度		群落的总盖度		
主要层优势种	乔木层：<br>灌木层：<br>草本层：			
群落外貌特点	次生林			
小地形及样地周围环境描述	农田 火山石多斑毛众多			
分层及各层的特点	乔木层	高度		
	灌木层	高度		
	草本层	高度		
	层间植物	高度		
		高度		
备注（之前的土地利用状况）	鲜重：0.10 kg			

说明：数据尽可能填写全面，没有填无

乔木层植物群落调查表

群落名称：乌墨　　样方面积：20 m × 20 m　　野外编号：74
调查时间：　2017.02.17.　15：00　　记录者：　　室内编号：

编号	植物名称	高度（m）	胸径（cm）	冠幅（m）	物候期	生活力	备注
1	乌墨	10	50	4×6	叶	1	
2	乌墨	10	50	4×6	叶	1	
3	乌墨	12	55	4×3	叶	1	
4	乌墨	15	60	3×6	叶	1	
5	龙眼	11	50	5×8	叶	1	
6	龙眼	12	50	4×3	叶	1	
7	苦楝	12	35	4×5	叶	3	
8	苦楝	8	20	3×4	休眠	3	
9							
10	秋枫	15	40	3×5	叶	1	
11							
12							
13							
14							
15							
16							
17							
18							
19							
20							
21							
22							
23							
24							
25							
26							
27							
28							
29							
30							
31							
32							
33							
34							
35							

灌丛层植物群落调查表

群落名称：鹊肾树-马樱丹-粗糠柴　　样方面积：11 m×1 m　　野外编号：74

调查时间：2017.02.17　15.：05　　记录者：　　室内编号：

编号	植物名称	高度（cm）	冠径（cm）	物候期	生活力	盖度%	株数/丛树
1							
2	酒饼簕	45	30	叶	2	15	
3	鹊肾树	300	100	叶	1	80	
4							
5							
6	马缨丹	150	120	叶	2	60	
7	鹊肾树	180	100	叶	1	45	
8							
9	破布叶	80	60	叶	2	30	
10	粗糖柴	180	120	叶	1	60	
11	大管	120	80	叶	1	40	
12							
13							
14							
15							
16							
17							
18							
19							
20							
21							
22							
23							
24							
25							
26							
27							
28							
29							
30							

草本层植物群落调查表

群落名称：斑茅-海芋　　样方面积 1 m × 1 m　　野外编号：74

调查时间：2017.02.17　15：00　　记录者：　　室内编号：

编号	植物名称	株高(cm)	盖度(%)	物候期	生活力	备注
1	鸭趾草	15	35	叶	1	
2	凤尾蕨	20	15	叶	1	
3	假蒟	25	10	叶	1	
4						
5	斑茅	250	80	叶花	1	
6						
7	假蒟	30	40	叶	2	
8	薜荔	5	40	叶	1	
9						
10	火炭母	25	25	叶	1	
11	海芋	65	65	叶	1	
12						
13	贴生石韦	20	20	叶	1	
14	夜香牛	20	20	叶花	1	
15						
16						
17						
18						
19						
20						
21						
22						
23						
24						
25						
26						
27						
28						
29						
30						

说明：物候期：花、叶、果

生活力：1　良好 2　一般　3　较差
