# Supplementary material for: Correction: Taxonomic and phylogenetic diversity of vascular plants at Ma'anling volcano urban park in tropical Haikou, China: Reponses to soil properties
Source: PLoS One. 2018 Dec 14;13(12):e0209520. doi: 10.1371/journal.pone.0209520 (PMC6294358; doi:10.1371/journal.pone.0209520)
Supplement: S5 File — (PDF) [file pone.0209520.s003.pdf]

总表

群落名称乔-灌-草优势种	荔枝-破布叶-吐烟花		野外编号(统一编号)	67
记录者		日期	2017.2.17 9：15	室内编号	
样地面积	20×20 m	详细地点		
GPS 定位	N：19°54.632′<br>E：110°14.063′	海拔高度		
群落高度		群落的总盖度	90%	
主要层优势种	乔木层：<br>灌木层：<br>草本层：			
群落外貌特点	人工林			
小地形及样地周围环境描述	火山岩果园			
分层及各层的特点	乔木层	高度		
	灌木层	高度		
	草本层	高度		
	层间植物	高度		
		高度		
备注（之前的土地利用状况）	鲜重：0.10 kg			

说明：数据尽可能填写全面，没有填无

乔木层植物群落调查表

群落名称：荔枝-菠萝蜜-毛八角枫　　样方面积：　　野外编号：67
调查时间：2017.02.17　9：15　　记录者：　　室内编号：

编号	植物名称	高度（m）	胸径（cm）	冠幅（m）	物候期	生活力	备注
1	荔枝	8	60	8×10	叶	1	
2	荔枝	9	70	9×10	叶	1	
3	菠萝蜜	8	20	4×5	叶	1	
4	菠萝蜜	8	15	4×5	果	1	
5	木棉	10	30	5×6	休眠	3	
6	毛八角枫	6	12	4×3	休眠	3	
7	黄皮	2	5	3×5	叶	1	
8	木瓜	4	8	2×2	果	2	
9	桉树	7	15	1×1	休眠	3	
10	毛八角枫	8	15	4×5	休眠	3	
11							
12							
13							
14							
15							
16							
17							
18							
19							
20							
21							
22							
23							
24							
25							
26							
27							
28							
29							
30							
31							
32							
33							
34							
35							

灌丛层植物群落调查表

群落名称：破布叶　　样方面积：1 m × 1 m　　野外编号：67
调查时间：2017.02.17　9：28　　记录者：　　室内编号：

编号	植物名称	高度（cm）	冠径（cm）	物候期	生活力	盖度%	株数 / 丛树
1	九节	30	20	叶	1	10	
2	黑面神	40	30	叶	1	20	
3	细基丸	60	40	叶	2	20	
4							
5	白藤	40	40	叶	2	30	
6	大青	80	40	叶	2	20	
7	毛柿	60	50	叶	2	20	
8	胡颓子	80	60	叶	2	30	
9							
10	山石榴	70	40	叶	1	20	
11	鸦胆子	100	40	叶	1	10	
12	黄牛木	80	20	叶	1	5	
13	破布叶	220	150	叶	1	40	
14							
15							
16							
17							
18							
19							
20							
21							
22							
23							
24							
25							
26							
27							
28							
29							
30							

草本层植物群落调查表

群落名称：吐烟花-金腰箭　　样方面积 1 m × 1 m　　野外编号：67
调查时间：2017.02.17　9：28　　记录者：　　室内编号：

编号	植物名称	株高(cm)	盖度(%)	物候期	生活力	备注
1	吐烟花	15	80	叶	1	
2	扭肚藤	80	20	叶	1	
3	凤尾葵	20	20	叶	1	
4	吐烟花	5	60	叶	1	
5	翼茎白粉藤	20	20	叶	2	
6	白粉藤	5	40	叶	2	
7	革命草	40	10	叶	1	
8	黄鹌菜	25	5	叶花	1	
9	一点红	15	5	叶花	1	
10	鬼针草	25	15	叶花果	2	
11	金腰箭	80	60	叶花	2	
12	威灵仙	15	20	叶	1	
13						
14						
15						
16						
17						
18						
19						
20						
21						
22						
23						
24						
25						
26						
27						
28						
29						
30						

说明：物候期：花、叶、果
生活力：1　良好 2　一般　3　较差

总表

群落名称<br>乔-灌-草<br>优势种	荔枝-油茶-吐烟花			野外编号<br>(统一编号)	68
记录者		日期	2017.02.17<br>8：45	室内编号	
样地面积	20×20 m	详细地点			
GPS 定位	N：19°54.704′<br>E：110°14.403′	海拔高度	131 m		
群落高度		群落的总盖度	95%		
主要层优势种	乔木层：<br>灌木层：<br>草本层：				
群落外貌特点	人工林				
小地形及样地周围环境描述	火山石众多				
分层及各层的特点	乔木层	高度			
	灌木层	高度			
	草本层	高度			
	层间植物	高度			
		高度			
备注（之前的土地利用状况）	鲜重：0.10 kg				

说明：数据尽可能填写全面，没有填无

乔木层植物群落调查表

群落名称：荔枝　　样方面积：　　野外编号：68

调查时间：2017.02.17　8：45　　记录者：　　室内编号：

编号	植物名称	高度（m）	胸径（cm）	冠幅（m）	物候期	生活力	备注
1	菠萝蜜	9	20	4×5	叶	1	
2	菠萝蜜	7.5	15	4×5	叶	1	
3	荔枝	10	80	6×10	叶	1	
4	荔枝	10	60	7×10	叶	1	
5	荔枝	9	40	6×10	叶	1	
6	银合欢	7	20	4×6	叶	2	
7	银合欢	6	20	4×6	叶	2	
8	银合欢	6	15	3×3	叶	2	
9	毛八角枫	10	15	3×3	休眠	3	
10	毛八角枫	7	10	3×3	休眠	3	
11	毛八角枫	9	15	3×3	休眠	3	
12							
13							
14							
15							
16							
17							
18							
19							
20							
21							
22							
23							
24							
25							
26							
27							
28							
29							
30							
31							
32							
33							
34							
35							

灌丛层植物群落调查表

群落名称：油茶-毛柿　　样方面积：5 m × 5 m　　野外编号：68
调查时间：　2017.02.17 8：45　　记录者：　　室内编号：

编号	植物名称	高度（cm）	冠径（cm）	物候期	生活力	盖度%	株数 / 丛树
1	越南悬钩子	120	60	叶	1	40	3
2	九节	130	80	叶	2	30	1
3	酒饼簕	60	40	叶	2	20	1
4	破布叶	80	60	叶	2	40	1
5	胡颓子	120	60	叶	1	30	1
6	黑面神	80	40	叶	1	20	1
7	油茶	200	130	叶	1	80	1
8	毛柿	150	80	叶	1	50	1
9	鲫鱼胆	150	60	叶花	1	30	1
10							
11							
12							
13							
14							
15							
16							
17							
18							
19							
20							
21							
22							
23							
24							
25							
26							
27							
28							
29							
30							

草本层植物群落调查表

群落名称：吐烟花-三点金-海芋　　样方面积 1 m × 1 m　　野外编号：68
调查时间：2017.02.17　8：57　　记录者：　　室内编号：

编号	植物名称	株高(cm)	盖度(%)	物候期	生活力	备注
1	鸭跖草	20	40	叶	1	
2	三点金	5	60	叶	1	
3	微甘菊	15	25	叶	1	
4	飞机草	25	20	叶	1	
5	黄鹌菜	15	10	叶花果	2	
6	丰花草	15	10	叶花果	2	
7	藿香蓟	15	20	叶花	2	
8	一年蓬	20	10	叶	1	
9	鬼针草	25	40	叶花	1	
10	吐烟花	15	90	叶	1	
11	微白菊	20	20	叶	1	
12	海芋	60	60	叶	1	
13	酢浆草	5	40	叶	1	
14	铁草鞋	300	50	叶	1	
15	鞘柄菝葜	180	20	叶	1	
16						
17						
18						
19						
20						
21						
22						
23						
24						
25						
26						
27						
28						
29						
30						

说明：物候期：花、叶、果
生活力：1　良好 2　一般　3　较差

总表

群落名称<br>乔-灌-草<br>优势种	菠萝蜜-海南菜豆树-飞机草			野外编号<br>(统一编号)	69
记录者		日期		室内编号	
样地面积		详细地点			
GPS 定位	N：19°54.820′<br>E：110°15.145′	海拔高度	88 m		
群落高度			群落的总盖度	70%	
主要层优势种	乔木层：<br>灌木层：<br>草本层：				
群落外貌特点	人工林				
小地形及样地周围环境描述	周围是果园，植被较多				
分层及各层的特点	乔木层	高度			
	灌木层	高度			
	草本层	高度			
	层间植物	高度			
		高度			
备注（之前的土地利用状况）	鲜重：0.12 kg				

说明：数据尽可能填写全面，没有填无

乔木层植物群落调查表

群落名称：黄皮-荔枝　　样方面积：20 m × 20 m　　野外编号：69
调查时间：　　记录者：　　室内编号：

编号	植物名称	高度（m）	胸径（cm）	冠幅（m）	物候期	生活力	备注
1	黄皮	4	12	2×2	叶	2	
2	黄皮	4	12	3×2	叶	2	
3	黄皮	4	10	3×2	叶	2	
4	黄皮	4	10	3×3	叶	2	
5	荔枝	3.5	12	3×3	叶	2	
6	荔枝	4	13	3×3	叶	2	
7	荔枝	3.5	12	3×3	叶	2	
8	莲雾	4.5	12	3×3	叶	2	
9	莲雾	4	13	3×2	叶	2	
10	菠萝蜜	10	35	4×5	果	2	
11	菠萝蜜	9	30	5×4	果	2	
12	菠萝蜜	9.5	32	5×5	果	2	
13	柚子	4	10	3×2	叶	2	
14	柚子	4	10	3×2	叶	2	
15	柚子	4	10	3×2	叶	2	
16	黄皮	4	10	3×2	叶	2	
17	荔枝	6.5	26	4×5	叶	2	
18	番石榴	3.5	13	3×2	叶	2	
19							
20							
21							
22							
23							
24							
25							
26							
27							
28							
29							
30							
31							
32							
33							
34							
35							

灌丛层植物群落调查表

群落名称：马樱丹-海南菜豆树　　样方面积：5 m × 5 m　　野外编号：69
调查时间：　　记录者：　　室内编号：

编号	植物名称	高度（cm）	冠径（cm）	物候期	生活力	盖度%	株数 / 丛树
1	马缨丹	40	30	花	2	30	1
2							
3	斑茅	60	10	叶	2	5	2
4	海南菜豆树	50	15	叶	2	10	2
5							
6	海南菜豆树	55	20	叶	2	10	1
7							
8							
9							
10							
11							
12							
13							
14							
15							
16							
17							
18							
19							
20							
21							
22							
23							
24							
25							
26							
27							
28							
29							
30							

草本层植物群落调查表

群落名称：荨麻-薇甘菊-飞机草　　样方面积 1 m × 1 m　　野外编号：69
调查时间：　　记录者：　　室内编号：

编号	植物名称	株高(cm)	盖度(%)	物候期	生活力	备注
1	薇甘菊	12	30	花	2	
2	飞机草	15	30	花	2	
3	水茄	15	10	叶	2	
4	丰花草	15	20	花	2	
5						
6	眼树莲	5	20	叶	2	
7	刺蒴麻	14	10	花	2	
8	梵天花	15	20	花	2	
9	假败酱	10	10	花	2	
10						
11	藿香蓟	20	20	花	2	
12	厚叶崖爬藤	5	20	叶	2	
13	黄花稔	15	20	花	2	
14	少花龙葵	10	20	花果	2	
15						
16	毒瓜	35	10	果	2	
17	丝瓜	40	20	果	2	
18	鬼针草	20	10	花	2	
19	火炭母	20	20	叶	2	
20	金腰箭	15	10	叶	2	
21						
22	芭蕉	12	6	叶	2	
23	荨麻	10	40	叶	2	
24	银胶菊	15	10	叶	2	
25	苋	10	15	叶	2	
26						
27						
28						
29						
30						

说明：物候期：花、叶、果
生活力：1　良好 2 一般 3 较差

总表

群落名称乔-灌-草优势种	荔枝-九节-菠萝			野外编号(统一编号)	70
记录者		日期		室内编号	
样地面积		详细地点			
GPS 定位	N：19°54.669′<br>E：110°15.500′	海拔高度	85 m		
群落高度		群落的总盖度	80%		
主要层优势种	乔木层：<br>灌木层：<br>草本层：				
群落外貌特点	人工林				
小地形及样地周围环境描述	果园周围				
分层及各层的特点	乔木层	高度			
	灌木层	高度			
	草本层	高度			
	层间植物	高度			
		高度			
备注（之前的土地利用状况）	鲜重 0.1 kg				

说明：数据尽可能填写全面，没有填无

乔木层植物群落调查表

群落名称：荔枝-菠萝蜜　　样方面积：20 m × 20 m　　野外编号：70
调查时间：　　记录者：　　室内编号：

编号	植物名称	高度（m）	胸径（cm）	冠幅（m）	物候期	生活力	备注
1	荔枝	10	60	7×6	叶	1	
2	樫木	10	30	5×5	叶	2	
3	荔枝	7	43	5×5	叶	2	
4	荔枝	7	35	5×4	叶	2	
5	荔枝	7	38	5×4	叶	2	
6	菠萝蜜	8	32	5×4	叶	2	
7	降香黄檀	6	15	3×3	叶	2	
8	荔枝	6	10	3×2	叶	2	
9	荔枝	6	18	3×3	叶	2	
10	黄皮	7	19	3×3	叶	2	
11	黄皮	6	19	3×3	叶	2	
12	黄皮	6	18	3×3	叶	2	
13	荔枝	6	18	3×3	叶	2	
14	荔枝	6	16	3×3	叶	2	
15	荔枝	3	10	3×2	叶	2	
16	荔枝	3	10	3×2	叶	2	
17	荔枝	3	8	3×2	叶	2	
18	菠萝蜜	5	13	3×2	果	2	
19	菠萝蜜	5	15	2×3	果	2	
20	菠萝蜜	5	16	2×3	叶	2	
21	菠萝蜜	5	15	2×3	叶	2	
22							
23							
24							
25							
26							
27							
28							
29							
30							
31							
32							
33							
34							
35							

灌丛层植物群落调查表

群落名称：九节-鸦胆子　　样方面积：5 m × 5 m　　野外编号：70
调查时间：　　记录者：　　室内编号：

编号	植物名称	高度（cm）	冠径（cm）	物候期	生活力	盖度%	株数 / 丛树
1	九节	30	18	叶	2	20	2
2	酒饼簕	15	5	叶	2	10	1
3							
4	三桠苦	10	5	叶	2	10	2
5	鸦胆子	30	19	叶	2	15	1
6							
7	黄牛木	16	10	叶	2	10	1
8	鲫鱼胆	80	10	叶	2	10	2
9							
10							
11							
12							
13							
14							
15							
16							
17							
18							
19							
20							
21							
22							
23							
24							
25							
26							
27							
28							
29							
30							

草本层植物群落调查表

群落名称：菠萝-波罗蜜　　样方面积 1 m × 1 m　　野外编号：70
调查时间：　　记录者：　　室内编号：

编号	植物名称	株高(cm)	盖度(%)	物候期	生活力	备注
1	山黄皮	13	15	叶	2	
2	吐烟花	8	30	叶	2	
3	菠萝	25	60	叶	2	
4	菠萝蜜	20	40	叶	2	
5						
6	肾蕨	10	6	叶	2	
7	贴生石韦	5	5	叶	2	
8	马交儿	15	5	叶	2	
9	飞机草	15	20	叶	2	
10						
11	夜香牛	15	20	花	2	
12	藿香蓟	10	20	花	2	
13	金腰箭	15	20	叶	2	
14						
15	井栏边草	16	10	叶	2	
16	粪箕笃	10	5	叶	2	
17						
18	土人参	20	5	叶	2	
19	毛柿	6	10	叶	2	
20	华南忍冬	18	5	叶	2	
21						
22						
23						
24						
25						
26						
27						
28						
29						
30						

说明：物候期：花、叶、果
生活力：1　良好 2 一般 3 较差

总表

<table>
<tr><td>群落名称
乔-灌-草
优势种</td><td colspan="4">簕竹-牛筋果-斑茅</td><td>野外编号
(统一编号)</td><td colspan="2">71</td></tr>
<tr><td>记录者</td><td colspan="2"></td><td>日期</td><td>2017.02.21
14：20</td><td>室内编号</td><td colspan="2"></td></tr>
<tr><td>样地面积</td><td colspan="2">20×20 m</td><td>详细地点</td><td colspan="4"></td></tr>
<tr><td>GPS 定位</td><td>N：19°54.266′
E：110°10.754′</td><td>海拔高度</td><td colspan="5">68 m</td></tr>
<tr><td>群落高度</td><td colspan="3"></td><td>群落的总盖度</td><td colspan="3">89%</td></tr>
<tr><td>主要层优势种</td><td colspan="7">乔木层：
灌木层：
草本层：</td></tr>
<tr><td>群落外貌特点</td><td colspan="7">荒野</td></tr>
<tr><td>小地形及样地周围环境描述</td><td colspan="7">杂草、杂木多，有个竹林</td></tr>
<tr><td rowspan="5">分层及各层的特点</td><td>乔木层</td><td>高度</td><td colspan="5"></td></tr>
<tr><td>灌木层</td><td>高度</td><td colspan="5"></td></tr>
<tr><td>草本层</td><td>高度</td><td colspan="5"></td></tr>
<tr><td>层间植物</td><td>高度</td><td colspan="5"></td></tr>
<tr><td></td><td>高度</td><td colspan="3"></td><td></td><td></td></tr>
<tr><td>备注（之前的土地利用状况）</td><td colspan="7">土壤鲜重：0.10 kg</td></tr>
</table>

说明：数据尽可能填写全面，没有填无

乔木层植物群落调查表

群落名称：簕竹　　样方面积：20 m × 20 m　　野外编号：71
调查时间：2017.02.21　14：20　　记录者：　　室内编号：

编号	植物名称	高度（m）	胸径（cm）	冠幅（m）	物候期	生活力	备注
1	簕竹	12	4	1×1	叶	1	
2	簕竹	12	5	2×2	叶	1	
3	簕竹	10	5	1×2	叶	1	
4	簕竹	13	5	1×1	叶	1	
5	簕竹	12	5	2×2	叶	1	
6	簕竹	9	4	1×1	叶	1	
7							
8							
9							
10							
11							
12							
13							
14							
15							
16							
17							
18							
19							
20							
21							
22							
23							
24							
25							
26							
27							
28							
29							
30							
31							
32							
33							
34							
35							

灌丛层植物群落调查表

群落名称：破布叶-牛筋果-九节　　样方面积：5 m × 5 m　　野外编号：71
调查时间：2017.02.21　14：20　　记录者：　　室内编号：

编号	植物名称	高度（cm）	冠径（cm）	物候期	生活力	盖度%	株数/丛树
1	牛筋果	170	100	叶花	2	60	1
2	倒吊笔	180	70	叶	2	50	1
3							
4	裸实	100	40	叶	1	10	2
5	雀梅	160	60	果	2	15	3
6	土蜜树	120	60	叶	2	10	1
7							
8	鸦胆子	170	100	叶	2	50	1
9	九节	170	100	叶	2	60	1
10	破布叶	160	120	叶	2	70	1
11	黑面神	150	70	叶	2	40	1
12							
13							
14							
15							
16							
17							
18							
19							
20							
21							
22							
23							
24							
25							
26							
27							
28							
29							
30							

草本层植物群落调查表

群落名称：斑茅-蔓生莠竹　　样方面积 1 m × 1 m　　野外编号：71
调查时间：2017.02.21　14：20　　记录者：　　室内编号：

编号	植物名称	株高(cm)	盖度(%)	物候期	生活力	备注
1	一年蓬	20	15	叶	1	
2	金腰箭	20	10	叶花	1	
3						
4	蔓生莠竹	60	70	叶	3	
5	一年蓬	100	5	叶花	1	
6	桤叶黄花棯	15	5	叶花	1	
7						
8	猪屎豆	120	10	叶花	2	
9	飞机草	70	20	叶	1	
10						
11	羽芒菊	5	5	叶花	2	
12	飞扬草	15	5	叶花果	2	
13	地毯草	80	10	叶花果	3	
14						
15	斑茅	400	70	叶果	2	
16	链荚豆	5	20	叶	2	
17	半枝莲	5	20	叶	2	
18						
19						
20						
21						
22						
23						
24						
25						
26						
27						
28						
29						
30						

说明：物候期：花、叶、果
生活力：1　良好 2　一般　3　较差

总表

群落名称<br>乔-灌-草<br>优势种	马占相思-两面针-蔓生莠竹			野外编号<br>(统一编号)	72
记录者		日期	2017.02.21<br>13：43	室内编号	
样地面积	20×20 m	详细地点			
GPS 定位	N：19°54.283′<br>E：110°11.214′	海拔高度	77 m		
群落高度			群落的总盖度	89%	
主要层优势种	乔木层：<br>灌木层：<br>草本层：				
群落外貌特点	人工林				
小地形及样地周围环境描述	农田边 河边				
分层及各层的特点	乔木层	高度			
	灌木层	高度			
	草本层	高度			
	层间植物	高度			
		高度			
备注（之前的土地利用状况）	土壤鲜重：0.12 kg				

说明：数据尽可能填写全面，没有填无

乔木层植物群落调查表

群落名称：马占相思　样方面积：20 m × 20 m　野外编号：72

调查时间：2017.02.21　13：43　记录者：　室内编号：

编号	植物名称	高度（m）	胸径（cm）	冠幅（m）	物候期	生活力	备注
1	马占相思	15	25	5×6	叶	2	
2	马占相思	15	30	5×5	叶	1	
3	马占相思	15	20	4×4	叶	1	
4	马占相思	12	20	4×3	叶	1	
5	马占相思	15	35	5×5	叶	1	
6	马占相思	15	20	2×2	叶		
7	马占相思	12	35	2×2	叶	1	
8	马占相思	15	40	4×4	叶	1	
9	马占相思	15	35	4×4	叶	1	
10	马占相思	15	30	4×3	叶	1	
11	马占相思	12	30	3×3	叶	1	
12	马占相思	16	30	3×3	叶	1	
13	马占相思	15	20	4×4	叶	1	
14	马占相思	15	25	3×3	叶	1	
15	马占相思	15	30	4×4	叶	1	
16	马占相思	12	20	1×1	叶	2	
17	马占相思	14	30	2×2	叶	2	
18	马占相思	13	30	2×2	叶	2	
19	马占相思	15	30	3×3	叶	2	
20							
21							
22							
23							
24							
25							
26							
27							
28							
29							
30							
31							
32							
33							
34							
35							

灌丛层植物群落调查表

群落名称：马樱丹-鹊肾树　　样方面积：5 m × 5 m　　野外编号：72
调查时间：2017.02.21　13：43　　记录者：　　室内编号：

编号	植物名称	高度（cm）	冠径（cm）	物候期	生活力	盖度%	株数/丛树
1	假杜鹃	30	30	花	1	20	3
2	马缨丹	160	120	花	1	60	2
3							
4	对叶榕	40	60	叶	1	20	2
5	潺槁木姜子	30	20	叶	2	20	1
6	鹊肾树	170	50	叶	1	60	1
7							
8	细基丸	40	20	叶	2	5	1
9	两面针	10	100	叶	1	20	1
10	大管	150	40	叶　花	1	10	1
11	牛筋果	160	20	叶	1	20	1
12							
13							
14							
15							
16							
17							
18							
19							
20							
21							
22							
23							
24							
25							
26							
27							
28							
29							
30							

草本层植物群落调查表

群落名称：蔓生莠竹-海芋-薇甘菊　　样方面积 1 m × 1 m　　野外编号：72
调查时间：2017.02.21　13：53　　记录者：　　室内编号：

编号	植物名称	株高(cm)	盖度(%)	物候期	生活力	备注
1	革命菜	40	10	花	2	
2	少花龙葵	20	60	花果	1	
3						
4	金腰箭	40	5	花	2	
5	鸭趾草	5	50	叶	1	
6						
7	薇甘菊	5	70	叶	2	
8	斑茅	400	70	叶花	2	
9	蛇葡萄	30	40	叶	2	
10						
11	酢浆草	20	40	叶花	2	
12	叶下珠	5	5	叶	2	
13	丰花草	10	10	叶花	1	
14						
15	火炭母	20	20	叶	2	
16	蔓生莠竹	30	80	叶	2	
17	水茄	50	50	叶	2	
18	海芋	140	80	叶花果	1	
19						
20						
21						
22						
23						
24						
25						
26						
27						
28						
29						
30						

说明：物候期：花、叶、果
生活力：1　良好 2　一般 3　较差

总表

群落名称<br>乔-灌-草<br>优势种	秋枫-鹊肾树-斑茅			野外编号(统一编号)	73
记录者		日期	2017.02.21 13：20	室内编号	
样地面积	20×20 m	详细地点			
GPS 定位	N：19°54.276′<br>E：110°11.771′	海拔高度	96 m		
群落高度		群落的总盖度	90%		
主要层优势种	乔木层：<br>灌木层：<br>草本层：				
群落外貌特点	少乔木，多草本				
小地形及样地周围环境描述	荒废土地 斑茅多				
分层及各层的特点	乔木层	高度			
	灌木层	高度			
	草本层	高度			
	层间植物	高度			
		高度			
备注（之前的土地利用状况）	土壤鲜重：0.14 kg				

说明：数据尽可能填写全面，没有填无

乔木层植物群落调查表

群落名称：秋枫-番石榴　　样方面积：20 m × 20 m　　野外编号：73
调查时间：2017.02.21　13：20　　记录者：　　室内编号：

编号	植物名称	高度（m）	胸径（cm）	冠幅（m）	物候期	生活力	备注
1	秋枫	12	45	8×6	叶	2	
2	番石榴	5	4	2×3	叶	2	
3							
4							
5							
6							
7							
8							
9							
10							
11							
12							
13							
14							
15							
16							
17							
18							
19							
20							
21							
22							
23							
24							
25							
26							
27							
28							
29							
30							
31							
32							
33							
34							
35							

灌丛层植物群落调查表

群落名称：鹊肾树-黑面神-酒饼簕　　样方面积：5 m × 5 m　　野外编号：73
调查时间：2017.02.21　13：25　　记录者：　　室内编号：

编号	植物名称	高度（cm）	冠径（cm）	物候期	生活力	盖度%	株数/丛树
1	黄牛木	140	50	叶	1	20	1
2	鹊肾树	140	120	叶	2	80	2
3							
4	鹊肾树	160	130	叶	1	80	2
5	马缨丹	130	60	叶花	1	50	1
6							
7	酒饼簕	190	100	叶	2	60	1
8	黑面神	200	120	叶果	2	70	1
9	破布叶	170	100	叶	2	40	1
10							
11							
12							
13							
14							
15							
16							
17							
18							
19							
20							
21							
22							
23							
24							
25							
26							
27							
28							
29							
30							

草本层植物群落调查表

群落名称：蔓生莠竹-斑茅　　样方面积 1 m × 1 m　　野外编号：73
调查时间：2017.02.21　13：20　　记录者：　　室内编号：

编号	植物名称	株高(cm)	盖度(%)	物候期	生活力	备注
1	斑茅	500	70	花果	3	
2	藿香蓟	60	30	花果	2	
3						
4	蔓生莠竹	30	80	叶	2	
5	夜香牛	25	20	叶花	2	
6						
7	薇甘菊	30	40	叶	2	
8	鸭跖草	10	20	叶	2	
9	蛇葡萄	30	40	叶	2	
10						
11	珍珠菜	8	20	叶花	1	
12	鸡屎藤	20	40	叶	2	
13	海芋	130	20	叶花	2	
14						
15	丰花草	15	10	叶花	2	
16	火炭母	20	20	叶花	1	
17						
18						
19						
20						
21						
22						
23						
24						
25						
26						
27						
28						
29						
30						

说明：物候期：花、叶、果
生活力：1　良好 2　一般　3　较差

总表

群落名称乔-灌-草优势种	秋枫-鹊肾树-斑茅		野外编号(统一编号)	73
记录者		日期	2017.02.21 13：20	室内编号	
样地面积	20×20 m	详细地点		
GPS 定位	N：19°54.276′<br>E：110°11.771′	海拔高度	96 m	
群落高度		群落的总盖度	90%	
主要层优势种	乔木层：<br>灌木层：<br>草本层：			
群落外貌特点	少乔木，多草本			
小地形及样地周围环境描述	荒废土地 斑茅多			
分层及各层的特点	乔木层	高度		
	灌木层	高度		
	草本层	高度		
	层间植物	高度		
		高度		
备注（之前的土地利用状况）	土壤鲜重：0.14 kg			

说明：数据尽可能填写全面，没有填无

乔木层植物群落调查表

群落名称：秋枫-番石榴　　样方面积：20 m × 20 m　　野外编号：73

调查时间：2017.02.21　13：20　　记录者：　　室内编号：

编号	植物名称	高度（m）	胸径（cm）	冠幅（m）	物候期	生活力	备注
1	秋枫	12	45	8×6	叶	2	
2	番石榴	5	4	2×3	叶	2	
3							
4							
5							
6							
7							
8							
9							
10							
11							
12							
13							
14							
15							
16							
17							
18							
19							
20							
21							
22							
23							
24							
25							
26							
27							
28							
29							
30							
31							
32							
33							
34							
35							

灌丛层植物群落调查表

群落名称：马樱丹-鹊肾树　　样方面积：5 m × 5 m　　野外编号：72
调查时间：2017.02.21　13：43　　记录者：　　室内编号：

编号	植物名称	高度（cm）	冠径（cm）	物候期	生活力	盖度%	株数/丛树
1	假杜鹃	30	30	花	1	20	3
2	马缨丹	160	120	花	1	60	2
3							
4	对叶榕	40	60	叶	1	20	2
5	潺槁木姜子	30	20	叶	2	20	1
6	鹊肾树	170	50	叶	1	60	1
7							
8	细基丸	40	20	叶	2	5	1
9	两面针	10	100	叶	1	20	1
10	大管	150	40	叶　花	1	10	1
11	牛筋果	160	20	叶	1	20	1
12							
13							
14							
15							
16							
17							
18							
19							
20							
21							
22							
23							
24							
25							
26							
27							
28							
29							
30							

草本层植物群落调查表

群落名称：蔓生莠竹-海芋-薇甘菊　　样方面积 1 m × 1 m　　野外编号：72
调查时间：2017.02.21　13：53　　记录者：　　室内编号：

编号	植物名称	株高(cm)	盖度(%)	物候期	生活力	备注
1	革命菜	40	10	花	2	
2	少花龙葵	20	60	花果	1	
3						
4	金腰箭	40	5	花	2	
5	鸭跖草	5	50	叶	1	
6						
7	薇甘菊	5	70	叶	2	
8	斑茅	400	70	叶花	2	
9	蛇葡萄	30	40	叶	2	
10						
11	酢浆草	20	40	叶花	2	
12	叶下珠	5	5	叶	2	
13	丰花草	10	10	叶花	1	
14						
15	火炭母	20	20	叶	2	
16	蔓生莠竹	30	80	叶	2	
17	水茄	50	50	叶	2	
18	海芋	140	80	叶花果	1	
19						
20						
21						
22						
23						
24						
25						
26						
27						
28						
29						
30						

说明：物候期：花、叶、果
生活力：1　良好 2　一般　3　较差

总表

<table>
<tr><td>群落名称
乔-灌-草
优势种</td><td colspan="3">马占相思-两面针-蔓生莠竹</td><td>野外编号
(统一编号)</td><td colspan="2">72</td></tr>
<tr><td>记录者</td><td colspan="2"></td><td>日期</td><td>2017.02.21
13：43</td><td>室内编号</td><td></td></tr>
<tr><td>样地面积</td><td colspan="2">20×20 m</td><td>详细地点</td><td colspan="3"></td></tr>
<tr><td>GPS 定位</td><td>N：19°54.283′
E：110°11.214′</td><td>海拔高度</td><td colspan="4">77 m</td></tr>
<tr><td>群落高度</td><td colspan="3"></td><td>群落的总盖度</td><td colspan="2">89%</td></tr>
<tr><td>主要层优势种</td><td colspan="6">乔木层：
灌木层：
草本层：</td></tr>
<tr><td>群落外貌特点</td><td colspan="6">人工林</td></tr>
<tr><td>小地形及样地周围环境描述</td><td colspan="6">农田边 河边</td></tr>
<tr><td rowspan="5">分层及各层的特点</td><td>乔木层</td><td>高度</td><td colspan="4"></td></tr>
<tr><td>灌木层</td><td>高度</td><td colspan="4"></td></tr>
<tr><td>草本层</td><td>高度</td><td colspan="4"></td></tr>
<tr><td>层间植物</td><td>高度</td><td colspan="4"></td></tr>
<tr><td></td><td>高度</td><td colspan="2"></td><td></td><td></td></tr>
<tr><td>备注（之前的土地利用状况）</td><td colspan="6">土壤鲜重：0.12 kg</td></tr>
</table>

说明：数据尽可能填写全面，没有填无

乔木层植物群落调查表

群落名称：马占相思　　样方面积：20 m × 20 m　　野外编号：72

调查时间：2017.02.21　13：43　　记录者：　　室内编号：

编号	植物名称	高度（m）	胸径（cm）	冠幅（m）	物候期	生活力	备注
1	马占相思	15	25	5×6	叶	2	
2	马占相思	15	30	5×5	叶	1	
3	马占相思	15	20	4×4	叶	1	
4	马占相思	12	20	4×3	叶	1	
5	马占相思	15	35	5×5	叶	1	
6	马占相思	15	20	2×2	叶		
7	马占相思	12	35	2×2	叶	1	
8	马占相思	15	40	4×4	叶	1	
9	马占相思	15	35	4×4	叶	1	
10	马占相思	15	30	4×3	叶	1	
11	马占相思	12	30	3×3	叶	1	
12	马占相思	16	30	3×3	叶	1	
13	马占相思	15	20	4×4	叶	1	
14	马占相思	15	25	3×3	叶	1	
15	马占相思	15	30	4×4	叶	1	
16	马占相思	12	20	1×1	叶	2	
17	马占相思	14	30	2×2	叶	2	
18	马占相思	13	30	2×2	叶	2	
19	马占相思	15	30	3×3	叶	2	
20							
21							
22							
23							
24							
25							
26							
27							
28							
29							
30							
31							
32							
33							
34							
35							

灌丛层植物群落调查表

群落名称：破布叶-牛筋果-九节　　样方面积：5 m × 5 m　　野外编号：71
调查时间：2017.02.21　14：20　　记录者：　　室内编号：

编号	植物名称	高度（cm）	冠径（cm）	物候期	生活力	盖度%	株数 / 丛树
1	牛筋果	170	100	叶花	2	60	1
2	倒吊笔	180	70	叶	2	50	1
3							
4	裸实	100	40	叶	1	10	2
5	雀梅	160	60	果	2	15	3
6	土蜜树	120	60	叶	2	10	1
7							
8	鸦胆子	170	100	叶	2	50	1
9	九节	170	100	叶	2	60	1
10	破布叶	160	120	叶	2	70	1
11	黑面神	150	70	叶	2	40	1
12							
13							
14							
15							
16							
17							
18							
19							
20							
21							
22							
23							
24							
25							
26							
27							
28							
29							
30							

草本层植物群落调查表

群落名称：斑茅-蔓生莠竹　　样方面积 1 m × 1 m　　野外编号：71
调查时间：2017.02.21　14：20　　记录者：　　室内编号：

编号	植物名称	株高(cm)	盖度(%)	物候期	生活力	备注
1	一年蓬	20	15	叶	1	
2	金腰箭	20	10	叶花	1	
3						
4	蔓生莠竹	60	70	叶	3	
5	一年蓬	100	5	叶花	1	
6	桤叶黄花棯	15	5	叶花	1	
7						
8	猪屎豆	120	10	叶花	2	
9	飞机草	70	20	叶	1	
10						
11	羽芒菊	5	5	叶花	2	
12	飞扬草	15	5	叶花果	2	
13	地毯草	80	10	叶花果	3	
14						
15	斑茅	400	70	叶果	2	
16	链荚豆	5	20	叶	2	
17	半枝莲	5	20	叶	2	
18						
19						
20						
21						
22						
23						
24						
25						
26						
27						
28						
29						
30						

说明：物候期：花、叶、果
生活力：1　良好 2　一般　3　较差

总表

群落名称乔-灌-草优势种	簕竹-牛筋果-斑茅			野外编号(统一编号)	71
记录者		日期	2017.02.21 14：20	室内编号	
样地面积	20×20 m	详细地点			
GPS 定位	N：19°54.266′<br>E：110°10.754′	海拔高度	68 m		
群落高度		群落的总盖度	89%		
主要层优势种	乔木层：<br>灌木层：<br>草本层：				
群落外貌特点	荒野				
小地形及样地周围环境描述	杂草、杂木多，有个竹林				
分层及各层的特点	乔木层	高度			
	灌木层	高度			
	草本层	高度			
	层间植物	高度			
		高度			
备注（之前的土地利用状况）	土壤鲜重：0.10 kg				

说明：数据尽可能填写全面，没有填无

乔木层植物群落调查表

群落名称：簕竹　　样方面积：20 m × 20 m　　野外编号：71
调查时间：2017.02.21　14：20　　记录者：　　室内编号：

编号	植物名称	高度（m）	胸径（cm）	冠幅（m）	物候期	生活力	备注
1	簕竹	12	4	1×1	叶	1	
2	簕竹	12	5	2×2	叶	1	
3	簕竹	10	5	1×2	叶	1	
4	簕竹	13	5	1×1	叶	1	
5	簕竹	12	5	2×2	叶	1	
6	簕竹	9	4	1×1	叶	1	
7							
8							
9							
10							
11							
12							
13							
14							
15							
16							
17							
18							
19							
20							
21							
22							
23							
24							
25							
26							
27							
28							
29							
30							
31							
32							
33							
34							
35							

灌丛层植物群落调查表

群落名称：海南破布叶-牛筋果-鹊肾树　　样方面积：1 m×1 m　　野外编号：75
调查时间：2017.02.17　11：05　　记录者：　　室内编号：

编号	植物名称	高度（cm）	冠径（cm）	物候期	生活力	盖度%	株数/丛树
1	假杜鹃	150	120	叶花	1	40	
2	牛筋果	450	180	叶	1	60	
3							
4	鹊肾树	250	80	叶	1	60	
5							
6	细基丸	150	60	叶果	1	20	
7							
8	海南破布叶	240	180	叶果	1	80	
9	白饭树	80	60	叶	1	20	
10							
11							
12							
13							
14							
15							
16							
17							
18							
19							
20							
21							
22							
23							
24							
25							
26							
27							
28							
29							
30							

草本层植物群落调查表

群落名称：蔓生莠竹-飞机草-芭蕉　　样方面积 1 m × 1 m　　野外编号：75
调查时间：　2017.02.17　11：10　记录者：　　室内编号：

编号	植物名称	株高(cm)	盖度(%)	物候期	生活力	备注
1	蔓生莠竹	40	90	叶花	1	
2						
3	蛇葡萄	90	50	叶	1	
4	假蒟	30	40	叶	1	
5						
6	飞机草	80	90	叶果	1	
7						
8	假蒟	20	80	叶	1	
9	芭蕉	400	90	叶	1	
10						
11	草	40	50	叶	1	
12	三叶崖爬藤	180	20	叶	1	
13						
14						
15						
16						
17						
18						
19						
20						
21						
22						
23						
24						
25						
26						
27						
28						
29						
30						

说明：物候期：花、叶、果
生活力：1　良好 2　一般　3　较差

总表

<table>
<tr><td>群落名称
乔-灌-草
优势种</td><td colspan="4">乌墨-牛筋果-蔓生莠竹</td><td>野外编号(统一编号)</td><td>75</td></tr>
<tr><td>记录者</td><td colspan="2"></td><td>日期</td><td>2017.02.17
11：05</td><td>室内编号</td><td></td></tr>
<tr><td>样地面积</td><td colspan="2">20×20 m</td><td>详细地点</td><td colspan="3"></td></tr>
<tr><td>GPS 定位</td><td>N：19°54.123′
E：110°12.909′</td><td>海拔高度</td><td colspan="4">124 m</td></tr>
<tr><td>群落高度</td><td colspan="3"></td><td>群落的总盖度</td><td colspan="2"></td></tr>
<tr><td>主要层优势种</td><td colspan="6">乔木层：
灌木层：
草本层：</td></tr>
<tr><td>群落外貌特点</td><td colspan="6">次生林</td></tr>
<tr><td>小地形及样地周围环境描述</td><td colspan="6">小山包、芭蕉众多、大树较多</td></tr>
<tr><td rowspan="5">分层及各层的特点</td><td>乔木层</td><td>高度</td><td colspan="4"></td></tr>
<tr><td>灌木层</td><td>高度</td><td colspan="4"></td></tr>
<tr><td>草本层</td><td>高度</td><td colspan="4"></td></tr>
<tr><td>层间植物</td><td>高度</td><td colspan="4"></td></tr>
<tr><td></td><td>高度</td><td colspan="2"></td><td></td><td></td></tr>
<tr><td>备注（之前的土地利用状况）</td><td colspan="6">鲜重：0.10 kg</td></tr>
</table>

说明：数据尽可能填写全面，没有填无

乔木层植物群落调查表

群落名称：乌墨　　样方面积：20 m × 20 m　　野外编号：75

调查时间：2017.02.17　11：05　　记录者：　　室内编号：

编号	植物名称	高度（m）	胸径（cm）	冠幅（m）	物候期	生活力	备注
1	乌墨	16	80	8×9	叶	1	
2	乌墨	15	80	8×8	叶	1	
3	乌墨	10	70	6×6	叶	1	
4	乌墨	10	75	4×6	叶	1	
5	乌墨	9	65	4×5	叶	2	
6							
7	龙眼	6	22	5×7	叶	1	
8							
9	菠萝蜜	8	20	5×8	叶	1	
10	菠萝蜜	7	20	5×7	叶	1	
11							
12	土蜜树	7	15	5×8	叶	1	
13							
14							
15							
16							
17							
18							
19							
20							
21							
22							
23							
24							
25							
26							
27							
28							
29							
30							
31							
32							
33							
34							
35							

灌丛层植物群落调查表

群落名称：鹊肾树-马樱丹-粗糠柴　　样方面积：11 m×1 m　　野外编号：74
调查时间：2017.02.17　15.：05　　记录者：　　室内编号：

编号	植物名称	高度（cm）	冠径（cm）	物候期	生活力	盖度%	株数/丛树
1							
2	酒饼簕	45	30	叶	2	15	
3	鹊肾树	300	100	叶	1	80	
4							
5							
6	马缨丹	150	120	叶	2	60	
7	鹊肾树	180	100	叶	1	45	
8							
9	破布叶	80	60	叶	2	30	
10	粗糖柴	180	120	叶	1	60	
11	大管	120	80	叶	1	40	
12							
13							
14							
15							
16							
17							
18							
19							
20							
21							
22							
23							
24							
25							
26							
27							
28							
29							
30							

草本层植物群落调查表

群落名称：斑茅-海芋　　样方面积 1 m × 1 m　　野外编号：74
调查时间：2017.02.17　15：00　　记录者：　　室内编号：

编号	植物名称	株高(cm)	盖度(%)	物候期	生活力	备注
1	鸭趾草	15	35	叶	1	
2	凤尾蕨	20	15	叶	1	
3	假蒟	25	10	叶	1	
4						
5	斑茅	250	80	叶花	1	
6						
7	假蒟	30	40	叶	2	
8	薜荔	5	40	叶	1	
9						
10	火炭母	25	25	叶	1	
11	海芋	65	65	叶	1	
12						
13	贴生石韦	20	20	叶	1	
14	夜香牛	20	20	叶花	1	
15						
16						
17						
18						
19						
20						
21						
22						
23						
24						
25						
26						
27						
28						
29						
30						

说明：物候期：花、叶、果
生活力：1　良好 2　一般　3　较差

总表

<table>
<tr><td>群落名称
乔-灌-草
优势种</td><td colspan="4">乌墨-鹊肾树-斑茅</td><td>野外编号
(统一编号)</td><td>74</td></tr>
<tr><td>记录者</td><td colspan="2"></td><td>日期</td><td>2017.02.17</td><td>室内编号</td><td></td></tr>
<tr><td>样地面积</td><td colspan="2"></td><td>详细地点</td><td colspan="3"></td></tr>
<tr><td>GPS 定位</td><td>N：19 °54.108′
E：110°12.134′</td><td>海拔高度</td><td colspan="4">116 m</td></tr>
<tr><td>群落高度</td><td colspan="3"></td><td>群落的总盖度</td><td colspan="2"></td></tr>
<tr><td>主要层优势种</td><td colspan="6">乔木层：
灌木层：
草本层：</td></tr>
<tr><td>群落外貌特点</td><td colspan="6">次生林</td></tr>
<tr><td>小地形及样地周围环境描述</td><td colspan="6">农田 火山石多斑毛众多</td></tr>
<tr><td rowspan="5">分层及各层的特点</td><td>乔木层</td><td>高度</td><td colspan="4"></td></tr>
<tr><td>灌木层</td><td>高度</td><td colspan="4"></td></tr>
<tr><td>草本层</td><td>高度</td><td colspan="4"></td></tr>
<tr><td>层间植物</td><td>高度</td><td colspan="4"></td></tr>
<tr><td></td><td>高度</td><td colspan="2"></td><td></td><td></td></tr>
<tr><td>备注（之前的土地利用状况）</td><td colspan="6">鲜重：0.10 kg</td></tr>
</table>

说明：数据尽可能填写全面，没有填无

乔木层植物群落调查表

群落名称：乌墨　　样方面积：20 m × 20 m　　野外编号：74

调查时间：　2017.02.17.　15：00　　记录者：　　室内编号：

编号	植物名称	高度（m）	胸径（cm）	冠幅（m）	物候期	生活力	备注
1	乌墨	10	50	4×6	叶	1	
2	乌墨	10	50	4×6	叶	1	
3	乌墨	12	55	4×3	叶	1	
4	乌墨	15	60	3×6	叶	1	
5	龙眼	11	50	5×8	叶	1	
6	龙眼	12	50	4×3	叶	1	
7	苦楝	12	35	4×5	叶	3	
8	苦楝	8	20	3×4	休眠	3	
9							
10	秋枫	15	40	3×5	叶	1	
11							
12							
13							
14							
15							
16							
17							
18							
19							
20							
21							
22							
23							
24							
25							
26							
27							
28							
29							
30							
31							
32							
33							
34							
35							

灌丛层植物群落调查表

群落名称：鹊肾树-黑面神-酒饼簕　　样方面积：5 m × 5 m　　野外编号：73
调查时间：2017.02.21　13：25　　记录者：　　室内编号：

编号	植物名称	高度（cm）	冠径（cm）	物候期	生活力	盖度%	株数/丛树
1	黄牛木	140	50	叶	1	20	1
2	鹊肾树	140	120	叶	2	80	2
3							
4	鹊肾树	160	130	叶	1	80	2
5	马缨丹	130	60	叶花	1	50	1
6							
7	酒饼簕	190	100	叶	2	60	1
8	黑面神	200	120	叶果	2	70	1
9	破布叶	170	100	叶	2	40	1
10							
11							
12							
13							
14							
15							
16							
17							
18							
19							
20							
21							
22							
23							
24							
25							
26							
27							
28							
29							
30							

草本层植物群落调查表

群落名称：蔓生莠竹-斑茅　　样方面积 1 m × 1 m　　野外编号：73
调查时间：2017.02.21　13：20　　记录者：　　室内编号：

编号	植物名称	株高(cm)	盖度(%)	物候期	生活力	备注
1	斑茅	500	70	花果	3	
2	藿香蓟	60	30	花果	2	
3						
4	蔓生莠竹	30	80	叶	2	
5	夜香牛	25	20	叶花	2	
6						
7	薇甘菊	30	40	叶	2	
8	鸭趾草	10	20	叶	2	
9	蛇葡萄	30	40	叶	2	
10						
11	珍珠菜	8	20	叶花	1	
12	鸡屎藤	20	40	叶	2	
13	海芋	130	20	叶花	2	
14						
15	丰花草	15	10	叶花	2	
16	火炭母	20	20	叶花	1	
17						
18						
19						
20						
21						
22						
23						
24						
25						
26						
27						
28						
29						
30						

说明：物候期：花、叶、果
生活力：1　良好 2　一般 3　较差

总表

群落名称<br>乔-灌-草<br>优势种	乌墨-马缨丹-鬼针草	野外编号<br>(统一编号)	78
记录者		日期	2017.02.21 9：54
		室内编号	
样地面积	20×20 m	详细地点	
GPS 定位	N：19°54.221′<br>E：110°14.524′	海拔高度	115 m
群落高度		群落的总盖度	65%
主要层优势种	乔木层：<br>灌木层：<br>草本层：		
群落外貌特点	人工林		
小地形及样地周围环境描述	火山石众多，荒废林		
分层及各层的特点	乔木层	高度	
	灌木层	高度	
	草本层	高度	
	层间植物	高度	
		高度	
备注（之前的土地利用状况）	土壤鲜重：0.10 kg		

说明：数据尽可能填写全面，没有填无

乔木层植物群落调查表

群落名称：乌墨-荔枝　　样方面积：20 m × 20 m　　野外编号：78

调查时间：2017.02.21　9：54　　记录者：　　室内编号：

编号	植物名称	高度（m）	胸径（cm）	冠幅（m）	物候期	生活力	备注
1	乌墨	12	60	10× 8	叶	2	
2	乌墨	12	60	8× 6	叶	2	
3	乌墨	10	45	8× 10	叶	2	
4	荔枝	6	20	6× 7	叶	1	
5	荔枝	7	15	5× 6	叶	1	
6	荔枝	8	25	6× 7	叶	1	
7							
8							
9							
10							
11							
12							
13							
14							
15							
16							
17							
18							
19							
20							
21							
22							
23							
24							
25							
26							
27							
28							
29							
30							
31							
32							
33							
34							
35							

灌丛层植物群落调查表

群落名称：马樱丹-羽叶山麻杆-假杜鹃-大花紫玉盘 样方面积：5 m × 5 m 野外编号：77
调查时间：2017.02.21 10：44 记录者： 室内编号：

编号	植物名称	高度（cm）	冠径（cm）	物候期	生活力	盖度%	株数/丛树
1	马缨丹	200	160	叶花	1	80	
2	毛柿	150	120	叶果	1	40	
3	赤楠	120	50	叶	2	40	
4	假杜鹃	160	120	叶花	2	60	
5							
6	大花紫玉盘	340	150	叶	1	60	
7	雀梅	170	20	叶	1	5	
8	簕欓花椒	1500	40	叶	1	10	
9	黄牛木	180	80	叶	2	40	
10							
11	大青	120	40	叶	2	10	
12	盐肤木	70	30	叶	1	5	
13	羽叶山麻杆	180	100	叶	2	70	
14	两面针	120	40	叶	2	20	
15							
16							
17							
18							
19							
20							
21							
22							
23							
24							
25							
26							
27							
28							
29							
30							

草本层植物群落调查表

群落名称：颠茄-鸭跖草-蔓生莠竹 样方面积 1 m × 1 m 野外编号：77
调查时间：2017.02.21 11：00 记录者： 室内编号：

编号	植物名称	株高(cm)	盖度(%)	物候期	生活力	备注
1	飞机草	140	40	叶果	2	
2	藿香蓟	20	30	叶花	1	
3	一年蓬	20	20	叶	1	
4						
5	络石藤	120	40	叶	2	
6	鸭趾草	20	70	叶	1	
7	天胡荽	2	10	叶	1	
8						
9	蔓生莠竹	80	70	叶	2	
10	假蒟	25	60	叶	2	
11						
12	颠茄	120	80	叶花果	1	
13	革命菜	40	20	叶花果	2	
14						
15	鬼针草	45	60	叶花果	2	
16	丰花草	15	5	叶花	2	
17	金腰箭	25	15	叶花	2	
18						
19						
20						
21						
22						
23						
24						
25						
26						
27						
28						
29						
30						

说明：物候期：花、叶、果
生活力：1 良好 2 一般 3 较差

总表

群落名称<br>乔-灌-草<br>优势种	桉树-山椒子-蔓生莠竹	野外编号<br>(统一编号)	77
记录者		日期	2017.02.21 10：44
		室内编号	
样地面积	20×20 m	详细地点	
GPS 定位	N：19°54.105′<br>E：110°14.109′	海拔高度	131 m
群落高度		群落的总盖度	95%
主要层优势种	乔木层：<br>灌木层：<br>草本层：		
群落外貌特点	桉树林		
小地形及样地周围环境描述	杂木多		
分层及各层的特点	乔木层	高度	
	灌木层	高度	
	草本层	高度	
	层间植物	高度	
		高度	
备注（之前的土地利用状况）	土壤鲜重：0.12 kg		

说明：数据尽可能填写全面，没有填无

乔木层植物群落调查表

群落名称：桉树　　样方面积：20 m × 20 m　　野外编号：77

调查时间：2017.02.21　10：44　　记录者：　　室内编号：

编号	植物名称	高度（m）	胸径（cm）	冠幅（m）	物候期	生活力	备注
1	桉树	10	6	2×2	叶	2	
2	桉树	13	8	3×2	叶	2	
3	桉树	10	6	2×2	叶	2	
4	桉树	12	8	2×3	叶	2	
5	桉树	9	6	2×2	叶	2	
6	桉树	10	6	2×3	叶	2	
7	桉树	11	6	2×3	叶	2	
8	桉树	12	6	2×2	叶	2	
9	桉树	10	5	2×2	叶	2	
10	番石榴	8	6	3×2	叶	3	
11	龙眼	6	6	4×5	叶	1	
12	破布叶	6	6	5×6	叶	1	
13							
14							
15							
16							
17							
18							
19							
20							
21							
22							
23							
24							
25							
26							
27							
28							
29							
30							
31							
32							
33							
34							
35							

灌丛层植物群落调查表

群落名称：榛叶黄花稔-两面针　　样方面积：1 m ×1 m　　野外编号：76
调查时间：2017.02.17　10：20　　记录者：　　室内编号：

编号	植物名称	高度（cm）	冠径（cm）	物候期	生活力	盖度%	株数/丛树
1	鹊肾树	50	80	叶	2	10	
2	苎麻	120	120	叶	1	20	
3	马樱丹	60	60	叶	1	20	
4	潺槁木姜子	160	60	叶	2	20	
5	两面针	60	40	叶	1	40	
6	榛叶黄花稔	180	80	叶花	1	60	
7							
8							
9							
10							
11							
12							
13							
14							
15							
16							
17							
18							
19							
20							
21							
22							
23							
24							
25							
26							
27							
28							
29							
30							

草本层植物群落调查表

群落名称：蔓生莠竹-节毛乌蔹莓　　样方面积 1 m × 1 m　　野外编号：76
调查时间：2017.02.17　10：31　　记录者：　　室内编号：

编号	植物名称	株高(cm)	盖度(%)	物候期	生活力	备注
1	飞机草	60	40	叶	1	
2	节毛乌蔹梅	120	50	叶花	1	
3		20	15	叶花	1	
4	丰花草	15	5	叶	1	
5	一点红	20	5	叶花	2	
6	落地生根	20	45	叶	1	
7	鸭跖草	15	5	叶	1	
8	蔓生莠竹	20	80	叶	1	
9	厚叶崖爬藤	40	40	叶	1	
10	三点金	5	40	叶	1	
11						
12						
13						
14						
15						
16						
17						
18						
19						
20						
21						
22						
23						
24						
25						
26						
27						
28						
29						
30						

说明：物候期：花、叶、果
生活力：1　良好 2　一般　3　较差

总表

群落名称<br>乔-灌-草<br>优势种	荔枝-苎麻-蔓生莠竹			野外编号(统一编号)	76
记录者		日期	2017.02.17<br>10：20	室内编号	
样地面积	20×20 m	详细地点			
GPS 定位	N：19°54.193′<br>E：110°13.346′	海拔高度	127 m		
群落高度		群落的总盖度	90%		
主要层优势种	乔木层：<br>灌木层：<br>草本层：				
群落外貌特点	人工林				
小地形及样地周围环境描述	火山石众多				
分层及各层的特点	乔木层	高度			
	灌木层	高度			
	草本层	高度			
	层间植物	高度			
		高度			
备注（之前的土地利用状况）					

说明：数据尽可能填写全面，没有填不

乔木层植物群落调查表

群落名称：荔枝-菠萝蜜-黄皮　样方面积：20 m × 20 m　野外编号：76

调查时间：2017.02.17　10：20　记录者：　室内编号：

编号	植物名称	高度（m）	胸径（cm）	冠幅（m）	物候期	生活力	备注
1	橘子	2.5	8	3×4	叶	1	
2	荔枝	7	20	6×8	叶	1	
3	荔枝	6	15	3×4	叶	1	
4	菠萝蜜	5	15	2×3	叶	1	
5	菠萝蜜	6	10	3×3	叶	1	
6	芒果	8	20	3×4	叶	2	
7	麻楝	8	22	3×5	叶	2	
8	苦楝	10	25	3×6	叶	2	
9	猫尾木	6	10	4×3	果	2	
10	黄皮	3.5	5	1×1	叶	2	
11	黄皮	4	7	1×2	叶	2	
12	黄皮	5	10	3×2	叶	2	
13							
14							
15							
16							
17							
18							
19							
20							
21							
22							
23							
24							
25							
26							
27							
28							
29							
30							
31							
32							
33							
34							
35							

灌丛层植物群落调查表

群落名称：毛柿-山石榴　　样方面积：5 m × 5 m　　野外编号：80
调查时间：2017.02.21　8：30　　记录者：　　室内编号：

编号	植物名称	高度（cm）	冠径（cm）	物候期	生活力	盖度%	株数 / 丛树
1	毛柿	160	150	叶果	2	60	1
2	马缨丹	200	40	叶花	2	20	1
3	山石榴	180	90	叶	3	60	1
4	大青	80	50	叶	1	20	1
5							
6	破布叶	60	50	叶	2	20	1
7	大管	70	40	叶	2	30	1
8							
9	两面针	170	70	叶	2	30	1
10							
11							
12							
13							
14							
15							
16							
17							
18							
19							
20							
21							
22							
23							
24							
25							
26							
27							
28							
29							
30							

草本层植物群落调查表

群落名称：斑茅-飞机草　　样方面积 1 m × 1 m　　野外编号：80
调查时间：　2017.02.21　8：30　　记录者：　　室内编号：

编号	植物名称	株高(cm)	盖度(%)	物候期	生活力	备注
1	假蒟	30	10	叶	2	
2	翼茎白粉藤	140	20	叶	2	
3	飞机草	70	50	叶	2	
4	吐烟花	5	60	叶	2	
5						
6	边缘鳞盖蕨	30	60	叶	1	
7	斑茅	300	80	叶花	3	
8						
9	夜香牛	130	20	叶花	2	
10	蜈蚣藤	200	50	叶	1	
11	抱树莲	200	40	叶	2	
12	贴生石韦	200	10	叶	2	
13						
14	飞机草	80	70	叶	2	
15	蔓生莠竹	60	50	叶	2	
16	土人参	20	20	叶	2	
17						
18	山牵牛	160	20	叶	2	
19	海芋	60	40	叶	2	
20	扭肚藤	100	15	叶	2	
21						
22						
23						
24						
25						
26						
27						
28						
29						
30						

说明：物候期：花、叶、果
生活力：1　良好 2 一般 3 较差

总表

<table>
<tr><td>群落名称
乔-灌-草
优势种</td><td colspan="4">荔枝-毛柿-飞机草</td><td>野外编号(统一编号)</td><td>80</td></tr>
<tr><td>记录者</td><td colspan="2"></td><td>日期</td><td>2017.02.21
8：30</td><td>室内编号</td><td></td></tr>
<tr><td>样地面积</td><td colspan="2">20×20 m</td><td>详细地点</td><td colspan="3"></td></tr>
<tr><td>GPS 定位</td><td>N：19°53.989′
E：110°。15.799′</td><td>海拔高度</td><td colspan="4">72 m</td></tr>
<tr><td>群落高度</td><td colspan="3"></td><td>群落的总盖度</td><td colspan="2">80%</td></tr>
<tr><td>主要层优势种</td><td colspan="6">乔木层：
灌木层：
草本层：</td></tr>
<tr><td>群落外貌特点</td><td colspan="6">次生林</td></tr>
<tr><td>小地形及样地周围环境描述</td><td colspan="6">火山石多，杂草丛生，荔枝多</td></tr>
<tr><td rowspan="5">分层及各层的特点</td><td>乔木层</td><td>高度</td><td colspan="4"></td></tr>
<tr><td>灌木层</td><td>高度</td><td colspan="4"></td></tr>
<tr><td>草本层</td><td>高度</td><td colspan="4"></td></tr>
<tr><td>层间植物</td><td>高度</td><td colspan="4"></td></tr>
<tr><td></td><td>高度</td><td colspan="2"></td><td></td><td></td></tr>
<tr><td>备注（之前的土地利用状况）</td><td colspan="6">土壤鲜重：0.10 kg</td></tr>
</table>

说明：数据尽可能填写全面，没有填无

乔木层植物群落调查表

群落名称：荔枝　　样方面积：20 m × 20 m　　野外编号：80
调查时间：2017.02.21　8：30　　记录者：　　室内编号：

编号	植物名称	高度（m）	胸径（cm）	冠幅（m）	物候期	生活力	备注
1	荔枝	12	50	8× 8	叶	1	
2	荔枝	10	45	6× 8	叶	1	
3	荔枝	11	40	8× 6	叶	1	
4	荔枝	12	30	6× 7	叶	1	
5	荔枝	9	35	8× 7	叶	1	
6	荔枝	10	50	9× 8	叶	1	
7	荔枝	12	45	8× 8	叶	1	
8	荔枝	13	49	8× 8	叶	1	
9	荔枝	11	48	8× 7	叶	1	
10	荔枝	10	45	9× 10	叶	1	
11	荔枝	12	40	8× 7	叶	1	
12	荔枝	10	50	8× 8	叶	1	
13	荔枝	9	43	8× 9	叶	1	
14	荔枝	10	45	9× 8	叶	1	
15	荔枝	8	0	10× 8	叶	1	
16	八角枫	6	6	4× 3	叶	2	
17							
18							
19							
20							
21							
22							
23							
24							
25							
26							
27							
28							
29							
30							
31							
32							
33							
34							
35							

灌丛层植物群落调查表

群落名称：假杜鹃-黑面神-大花紫玉盘　　样方面积：5 m × 5 m　　野外编号：79
调查时间：2017.02.21　9：21　　记录者：　　室内编号：

编号	植物名称	高度（cm）	冠径（cm）	物候期	生活力	盖度%	株数 / 丛树
1	毛柿	140	50	叶	2	40	1
2	假杜鹃	30	60	叶	3	70	1
3	黑面神	170	80	叶	2	70	1
4							
5	马缨丹	200	50	叶花	2	60	1
6	了哥王	60	30	叶	2	40	1
7	排钱草	100	70	叶	3	50	1
8							
9	赤楠	120	40	叶	1	20	1
10	大花紫玉盘	170	120	叶	2	70	1
11	九节	160	40	叶	3	40	1
12							
13							
14							
15							
16							
17							
18							
19							
20							
21							
22							
23							
24							
25							
26							
27							
28							
29							
30							

草本层植物群落调查表

群落名称：斑茅-蔓生莠竹　　样方面积 1 m × 1 m　　野外编号：79
调查时间：2017.02.21　9：21　　记录者：　　室内编号：

编号	植物名称	株高(cm)	盖度(%)	物候期	生活力	备注
1	蝙蝠草	20	20	叶花	2	
2	斑茅	300	80	叶花果	1	
3						
4	蔓生莠竹	150	80	叶	1	
5	藿香蓟	20	30	叶花	2	
6						
7	络石藤	5	20	叶	2	
8	飞机草	80	40	叶果	1	
9						
10	夜香牛	70	40	叶花	2	
11	一年蓬	60	20	叶	1	
12						
13	猫尾草	60	10	叶果	2	
14	鸭趾草	70	10	叶	2	
15						
16						
17						
18						
19						
20						
21						
22						
23						
24						
25						
26						
27						
28						
29						
30						

说明：物候期：花、叶、果
生活力：1　良好 2 一般 3 较差

总表

<table>
<tr><td>群落名称
乔-灌-草
优势种</td><td colspan="4">苦楝-山椒子-斑茅</td><td>野外编号(统一编号)</td><td>79</td></tr>
<tr><td>记录者</td><td colspan="2"></td><td>日期</td><td>2017.02.21
9：21</td><td>室内编号</td><td></td></tr>
<tr><td>样地面积</td><td colspan="2">20×20 m</td><td>详细地点</td><td colspan="3"></td></tr>
<tr><td>GPS 定位</td><td>N：19°54.160′
E：110°15.146′</td><td>海拔高度</td><td colspan="4">104m</td></tr>
<tr><td>群落高度</td><td colspan="3"></td><td>群落的总盖度</td><td colspan="2">85%</td></tr>
<tr><td>主要层优势种</td><td colspan="6">乔木层：
灌木层：
草本层：</td></tr>
<tr><td>群落外貌特点</td><td colspan="6">次生林</td></tr>
<tr><td>小地形及样地周围环境描述</td><td colspan="6">火山岩多，地表土少</td></tr>
<tr><td rowspan="5">分层及各层的特点</td><td>乔木层</td><td>高度</td><td colspan="4"></td></tr>
<tr><td>灌木层</td><td>高度</td><td colspan="4"></td></tr>
<tr><td>草本层</td><td>高度</td><td colspan="4"></td></tr>
<tr><td>层间植物</td><td>高度</td><td colspan="4"></td></tr>
<tr><td></td><td>高度</td><td colspan="2"></td><td></td><td></td></tr>
<tr><td>备注（之前的土地利用状况）</td><td colspan="6">土壤鲜重：0.08 kg</td></tr>
</table>

说明：数据尽可能填写全面，没有填无

乔木层植物群落调查表

群落名称：番石榴-苦楝　　样方面积：20 m × 20 m　　野外编号：79
调查时间：2017.02.21　9：21　　记录者：　　室内编号：

编号	植物名称	高度（m）	胸径（cm）	冠幅（m）	物候期	生活力	备注
1	番石榴	4	4	2×3	叶	2	
2	苦楝	8	6	6×5	叶	2	
3							
4							
5							
6							
7							
8							
9							
10							
11							
12							
13							
14							
15							
16							
17							
18							
19							
20							
21							
22							
23							
24							
25							
26							
27							
28							
29							
30							
31							
32							
33							
34							
35							

灌丛层植物群落调查表

群落名称：白背叶-马樱丹-番木瓜　　样方面积：5 m × 5 m　　野外编号：78
调查时间：2017.02.21　9：54　　记录者：　　室内编号：

编号	植物名称	高度（cm）	冠径（cm）	物候期	生活力	盖度%	株数 / 丛树
1	潺槁木姜子	50	20	叶	2	40	
2	簕欓花椒	80	40	叶	2	50	
3	油茶	130	100	叶	2	30	
4							
5	番木瓜	200	150	叶果	1	70	
6	白背叶	100	60	叶	2	80	
7							
8	毛柿	60	60	叶	2	30	
9	酒饼簕	70	50	叶	1	20	
10	马缨丹	150	155	叶	1	80	
11	破布叶	40	30	叶果	2	20	
12							
13							
14							
15							
16							
17							
18							
19							
20							
21							
22							
23							
24							
25							
26							
27							
28							
29							
30							

草本层植物群落调查表

群落名称：鬼针草-刺苋-夜香牛　　样方面积 1 m × 1 m　　野外编号：78
调查时间：2017.02.21　9：54　　记录者：　　室内编号：

编号	植物名称	株高(cm)	盖度(%)	物候期	生活力	备注
1	一年蓬	120	10	叶花	1	
2	鬼针草	40	70	叶花	1	
3	刺苋	80	60	叶花	2	
4						
5	青葙	150	20	叶花	2	
6	薇甘菊	10	40	叶	2	
7	藿香蓟	20	10	叶花	2	
8	苦蘵	5	10	叶花	2	
9						
10	皱子白花菜	5	20	叶	2	
11	蔓生莠竹	5	60	叶	1	
12	夜香牛	30	20	叶花	2	
13						
14	少花龙葵	20	20	叶花	2	
15	丰花草	10	10	叶花	2	
16	小茄	2	5	叶花	2	
17	一点红	15	10	叶花	2	
18						
19	羽芒菊	20	20	叶花	2	
20	墨苜蓿	3	5	叶	2	
21	铁草鞋	200	40	叶	2	
22						
23						
24						
25						
26						
27						
28						
29						
30						

说明：物候期：花、叶、果
生活力：1　良好 2 一般 3 较差

总表

群落名称 乔-灌-草 优势种	秋枫-鹊肾树-鬼针草			野外编号 (统一编号)	83
记录者	袁浪兴	日期	2017.02.17 11：10	室内编号	
样地面积	20×20 m	详细地点			
GPS 定位	N：19°53.696′ E：110°11.703′	海拔高度	94 m		
群落高度			群落的总盖度	95%	
主要层优势种	乔木层：<br>灌木层：<br>草本层：				
群落外貌特点	人工林				
小地形及样地周围环境描述	农田，苗圃旁。				
分层及各层的特点	乔木层	高度			
	灌木层	高度			
	草本层	高度			
	层间植物	高度			
		高度			
备注（之前的土地利用状况）	土壤鲜重：0.12 kg				

说明：数据尽可能填写全面，没有填无

乔木层植物群落调查表

群落名称：秋枫　样方面积：　野外编号：83
调查时间：2017.02.17　11：10　记录者：　室内编号：

编号	植物名称	高度（m）	胸径（cm）	冠幅（m）	物候期	生活力	备注
1	秋枫	9	20	4×5	叶	1	
2	秋枫	6	15	5×6	叶	1	
3	秋枫	5	6	2×3	叶	1	
4							
5	糖胶	8	18	2×2	叶	1	
6	糖胶	8	20	2×2	叶	1	
7							
8	幌伞枫	8	18	3×6	果	1	
9							
10	鸡冠刺桐	4.5	12	2×3	叶	2	
11							
12	大花紫薇	4	15	3×4	果	2	
13	大花紫薇	4	10	2×2	果	2	
14							
15	美丽梧桐	6	15	2×2	叶	2	
16							
17	五月茶	5	25	3×3	叶	1	
18							
19	大叶榄仁	7	30	2×3	休眠	3	
20	大叶榄仁	8	35	3×3	休眠	3	
21							
22	澳洲鸭脚木	7	10	1×1	叶	1	
23							
24	鱼尾葵	6	15	1×1	叶	1	
25							
26	金脉刺桐	7	20	3×2	叶	1	
27							
28							
29							
30							
31							
32							
33							
34							
35							

灌丛层植物群落调查表

群落名称：马樱丹　　样方面积：1 m × 1 m　　野外编号：82
调查时间：2017.02.17.　12：35　　记录者：　　室内编号：

编号	植物名称	高度（cm）	冠径（cm）	物候期	生活力	盖度%	株数/丛树
1	鹊肾树	250	180	叶	2	56	
2	马缨丹	80	40	叶	3	30	
3							
4	对叶榕	150	80	叶	1	40	
5	马缨丹	150	120	叶	1	58	
6							
7	鸦胆子	150	60	叶	2	30	
8	鸦胆子	120	180	叶	2	60	
9							
10	鸦胆子	180	120	叶	2	40	
11							
12							
13							
14							
15							
16							
17							
18							
19							
20							
21							
22							
23							
24							
25							
26							
27							
28							
29							
30							

草本层植物群落调查表

群落名称：鸭跖草-淡竹叶-斑茅　　样方面积 1 m × 1 m　　野外编号：82
调查时间：2017.02.17　　记录者：　　室内编号：

编号	植物名称	株高(cm)	盖度(%)	物候期	生活力	备注
1	鸭趾草	10	85	叶花	1	
2						
3	飞机草	80	30	叶	1	
4	白花鬼针草	40	20	叶花果	2	
5						
6	一点红	25	15	叶花	2	
7	白花鬼针草	15	60		2	
8	藿香蓟	15	5	叶花	3	
9						
10	淡竹叶	15	80	叶花果	1	
11	黄鹌菜	25	10	叶花	1	
12						
13	夜香牛	15	40	叶花	2	
14	鸭趾草	5	35	叶	1	
15	金腰箭	25	15	叶花	1	
16						
17	斑茅	230	80	叶花	1	
18						
19						
20						
21						
22						
23						
24						
25						
26						
27						
28						
29						
30						

说明：物候期：花、叶、果
生活力：1　良好 2 一般 3 较差

总表

群落名称<br>乔-灌-草<br>优势种	苦楝-鹊肾树-飞机草			野外编号<br>(统一编号)	82
记录者	袁浪兴	日期	2017.01.07<br>11：10	室内编号	82
样地面积	20×20 m	详细地点			
GPS 定位	N：19°53.556′<br>E：110°11.036′	海拔高度	87m		
群落高度			群落的总盖度	95%	
主要层优势种	乔木层：<br>灌木层：<br>草本层：				
群落外貌特点	人工林				
小地形及样地周围环境描述	槟榔园旁				
分层及各层的特点	乔木层	高度			
	灌木层	高度			
	草本层	高度			
	层间植物	高度			
		高度			
备注（之前的土地利用状况）	鲜重：0.12 kg				

说明：数据尽可能填写全面，没有填无

乔木层植物群落调查表

群落名称：苦楝-大叶榄仁　　样方面积：20 m × 20 m　　野外编号：82
调查时间：2017.02.17　　记录者：　　室内编号：

编号	植物名称	高度（m）	胸径（cm）	冠幅（m）	物候期	生活力	备注
1	苦楝	9	12	2×2	叶	2	
2	苦楝	15	20	2×3	叶	2	
3	苦楝	14	20	2×2	叶	2	
4	苦楝	14	20	2×2	叶	2	
5	苦楝	6	12	3×2	叶	2	
6							
7	木麻黄	17	30	5×4	叶	1	
8							
9	大叶榄仁	12	30	4×3	叶	1	
10	大叶榄仁	12	20	4×3	叶	1	
11	大叶榄仁	12	18	3×2	叶	1	
12	大叶榄仁	13	15	3×3	叶	1	
13							
14	槟榔	5	12	2×2	叶	1	
15	槟榔	4	15	2.5×3	叶	1	
16							
17							
18							
19							
20							
21							
22							
23							
24							
25							
26							
27							
28							
29							
30							
31							
32							
33							
34							
35							

灌丛层植物群落调查表

群落名称：两面针　　　样方面积：5 m × 5 m　　　野外编号：

调查时间：2017.02.17.　12：54　　　记录者：　　　室内编号：81

编号	植物名称	高度（cm）	冠径（cm）	物候期	生活力	盖度%	株数/丛树
1	两面针	60	30	叶	1	150	
2	越南悬钩子	80	60	叶	1	40	
3							
4	马樱丹	150	120	叶	2	50	
5	海南茄	80	20	叶	1	20	
6							
7	海南破布叶	210	120	叶	1	80	
8	大青	60	30	叶	1	10	
9	大青	45	30	叶	2	10	
10							
11							
12							
13							
14							
15							
16							
17							
18							
19							
20							
21							
22							
23							
24							
25							
26							
27							
28							
29							
30							

草本层植物群落调查表

群落名称：蔓生莠竹-假蒟　　　样方面积 1 m × 1 m　　　野外编号：

调查时间：　　　记录者：　　　室内编号：

编号	植物名称	株高(cm)	盖度(%)	物候期	生活力	备注
1	蔓生莠竹	80	90	叶花	1	
2	粪箕笃	80	5	叶花	1	
3						
4	火炭母	60	25	叶	1	
5	革命菜	15	5	叶	1	
6	假蒟	15	80	叶	1	
7	凤尾蕨	25	20	叶		
8						
9	鸡屎藤	40	15	叶	1	
10	鸭跖草	15	20	叶	1	
11	海芋	15	20	叶	1	
12						
13	海芋	80	60	叶	1	
14	菜蕨	30	40	叶	1	
15	地毯草	80	40	叶花	1	
16						
17						
18						
19						
20						
21						
22						
23						
24						
25						
26						
27						
28						
29						
30						

说明：物候期：花、叶、果

生活力：1　良好 2　一般　3　较差

总表

群落名称乔-灌-草优势种	苦楝-破布叶-蔓生莠竹			野外编号(统一编号)	81
记录者		日期	2017.02.17	室内编号	
样地面积		详细地点			
GPS 定位	N：19°53.522′<br>E：110°10.504′	海拔高度	80 m		
群落高度			群落的总盖度		
主要层优势种	乔木层：<br>灌木层：<br>草本层：				
群落外貌特点	人工林				
小地形及样地周围环境描述	农田				
分层及各层的特点	乔木层	高度			
	灌木层	高度			
	草本层	高度			
	层间植物	高度			
		高度			
备注（之前的土地利用状况）	鲜重：0.12 kg				

说明：数据尽可能填写全面，没有填无

乔木层植物群落调查表

群落名称：小叶榄仁　　样方面积：20 m × 20 m　　野外编号：
调查时间：　　记录者：　　室内编号：

编号	植物名称	高度（m）	胸径（cm）	冠幅（m）	物候期	生活力	备注
1	秋枫	8	20	3×2	叶	1	
2	秋枫	4	18	2×3	叶	1	
3	毛八角枫	6	15	3×3	叶	2	
4							
5	苦楝	14	30	4×5	叶	2	
6	苦楝	13	40	3×5	叶	2	
7							
8	榕树	15	20	2×2	叶	2	
9							
10	对叶榕	5	12	2×2	叶	2	
11							
12	小叶榄仁	12	10	2×2	叶	1	
13	小叶榄仁	12	10	2×2	叶	1	
14	小叶榄仁	10	10	2×2	叶	1	
15	小叶榄仁	12	18	3×4	叶	1	
16							
17							
18							
19							
20							
21							
22							
23							
24							
25							
26							
27							
28							
29							
30							
31							
32							
33							
34							
35							

灌丛层植物群落调查表

群落名称：破布叶　　样方面积：5 m × 5 m　　野外编号：85

调查时间：2017.02.21　12：45　　记录者：　　室内编号：

编号	植物名称	高度（cm）	冠径（cm）	物候期	生活力	盖度%	株数 / 丛树
1	赪桐	120	25	花果	3	5	1
2	山小橘	120	30	花果	1	10	1
3	海南茄	130	80	花	1	10	1
4							
5	鹊肾树	40	30	叶	2	40	1
6	两面针	200	40	叶	2	40	1
7	福建茶	40	20	叶	2	10	1
8							
9	白藤	30	50	叶	2	20	1
10	破布叶	160	70	叶	2	50	1
11	马缨丹	100	50	叶花	1	40	1
12							
13							
14							
15							
16							
17							
18							
19							
20							
21							
22							
23							
24							
25							
26							
27							
28							
29							
30							

草本层植物群落调查表

群落名称：鸭跖草-掌叶鱼黄草-海芋　样方面积 1 m × 1 m　野外编号：85

调查时间：2017.02.21　12：43　　记录者：　　室内编号：

编号	植物名称	株高(cm)	盖度(%)	物候期	生活力	备注
1	鸭趾草	15	70	叶	1	
2	海芋	30	60	叶花	1	
3						
4	藿香蓟	25	20	叶花	2	
5	蔓生莠竹	0	50	叶	1	
6						
7	掌叶鱼黄草	130	70	叶	1	
8	火炭母	20	50	叶	1	
9	假蒟	20	20	叶	2	
10						
11	海芋	120	70	叶花	1	
12	薯莨	130	20	叶	2	
13	飞机草	10	10	叶	2	
14						
15	夜香牛	20	5	叶花	2	
16	叶下珠	10	5	叶	1	
17	金腰箭	30	10	叶花	2	
18	丰花草	15	2	叶花	2	
19						
20						
21						
22						
23						
24						
25						
26						
27						
28						
29						
30						

说明：物候期：花、叶、果

生活力：1　良好 2　一般 3　较差

总表

群落名称乔-灌-草优势种	荔枝-两面针-海芋			野外编号(统一编号)	85
记录者		日期	2017.2.21 12：43	室内编号	
样地面积	20×20 m	详细地点			
GPS 定位	N：19°53.524′<br>E：110°12.934′	海拔高度	122 m		
群落高度		群落的总盖度	95%		
主要层优势种	乔木层：<br>灌木层：<br>草本层：				
群落外貌特点	人工林				
小地形及样地周围环境描述	荒废荔枝林村落旁				
分层及各层的特点	乔木层	高度			
	灌木层	高度			
	草本层	高度			
	层间植物	高度			
		高度			
备注（之前的土地利用状况）	土壤鲜重：0.12 kg				

说明：数据尽可能填写全面，没有填无

乔木层植物群落调查表

群落名称：荔枝　　样方面积：20 m × 20 m　　野外编号：85

调查时间：2017.02.21　12：43　　记录者：　　室内编号：

编号	植物名称	高度（m）	胸径（cm）	冠幅（m）	物候期	生活力	备注
1	山楝	8	20	3×3	叶	1	
2	荔枝	15	90	8×8	叶	1	
3	荔枝	15	90	8×8	叶	1	
4	荔枝	15	60	6×6	叶	1	
5	荔枝	12	50	4×4	叶	1	
6	苦楝	16	40	6×6	果	1	
7	苦楝	12	15	3×3	果	1	
8	猫尾木	12	30	2×2	果	1	
9	猫尾木	15	40	3×3	果	1	
10	乌墨	16	40	4×5	叶	1	
11							
12							
13							
14							
15							
16							
17							
18							
19							
20							
21							
22							
23							
24							
25							
26							
27							
28							
29							
30							
31							
32							
33							
34							
35							

灌丛层植物群落调查表

群落名称：鹊肾树-白饭树-牛筋藤　　样方面积：1 m × 1 m　　野外编号：84
调查时间：2017.02.17　11：30　　记录者：　袁浪兴　　室内编号：

编号	植物名称	高度（cm）	冠径（cm）	物候期	生活力	盖度%	株数/丛树
1	海南破布叶	120	40	叶	2	20	
2	鹊肾树	150	60	叶	1	40	
3							
4	细基丸	80	40	叶	2	20	
5	牛筋藤	300	120	叶	2	40	
6							
7	鸦胆子	120	40	叶	2	20	
8							
9	白饭树	140	80	叶	2	40	
10	山黄麻	60	20	叶	2	20	
11							
12							
13							
14							
15							
16							
17							
18							
19							
20							
21							
22							
23							
24							
25							
26							
27							
28							
29							
30							

草本层植物群落调查表

群落名称：白花鬼针草-斑茅　　样方面积 1 m × 1 m　　野外编号：84
调查时间：2017.2.17　11：44　　记录者：袁浪兴　　室内编号：

编号	植物名称	株高(cm)	盖度(%)	物候期	生活力	备注
1	飞机草	60	40	叶花	1	
2	少花龙葵	40	40	叶花果	1	
3						
4	白花鬼针草	60	90	叶花果	1	
5	毒瓜	40	20	叶	1	
6						
7	革命菜	20	15	叶	2	
8	斑茅	180	80	叶	1	
9						
10	藿香蓟	20	15	叶花	1	
11	金腰箭	30	40	叶花	2	
12						
13	一年蓬	20	40	叶	1	
14	蛇葡萄	40	20	叶	2	
15						
16						
17						
18						
19						
20						
21						
22						
23						
24						
25						
26						
27						
28						
29						
30						

说明：物候期：花、叶、果
生活力：1　良好 2　一般　3　较差

总表

群落名称乔-灌-草优势种	乌墨-牛筋果-鬼针草	野外编号(统一编号)	84
记录者	袁浪兴	日期	2017.02.17 11：30	室内编号	
样地面积	20×20 m	详细地点	
GPS 定位	N：19°53.770′<br>E：110°12.350′	海拔高度	109 m
群落高度		群落的总盖度	80%
主要层优势种	乔木层：<br>灌木层：<br>草本层：		
群落外貌特点	次生林		
小地形及样地周围环境描述	农田旁		
分层及各层的特点	乔木层	高度	
	灌木层	高度	
	草本层	高度	
	层间植物	高度	
		高度	
备注（之前的土地利用状况）	土壤鲜重 0.12 kg		

说明：数据尽可能填写全面，没有填无

乔木层植物群落调查表

群落名称：乌墨-龙眼　样方面积：　野外编号：84
调查时间：2017.02.17　11：35　记录者：袁浪兴　室内编号：

编号	植物名称	高度（m）	胸径（cm）	冠幅（m）	物候期	生活力	备注
1	乌墨	11	45	8×5	叶	2	
2	乌墨	12	50	7×9	叶	2	
3	乌墨	9	40	5×8	叶	2	
4							
5	龙眼	9	20	6×7	叶	1	
6	龙眼	9	30	7×8	叶	1	
7	龙眼	8	30	6×9	叶	1	
8							
9							
10							
11							
12							
13							
14							
15							
16							
17							
18							
19							
20							
21							
22							
23							
24							
25							
26							
27							
28							
29							
30							
31							
32							
33							
34							
35							

灌丛层植物群落调查表

群落名称：鹊肾树　　样方面积：1 m × 1 m　　野外编号：83
调查时间：2017.02.17　12：10　　记录者：　袁浪兴　　室内编号：

编号	植物名称	高度（cm）	冠径（cm）	物候期	生活力	盖度%	株数 / 丛树
1	酒饼簕	20	15	叶	1	5	
2	铁包金	35	20	叶	1	15	
3							
4	海南破布叶	50	40	叶	2	20	
5	海南破布叶	45	35	叶	3	15	
6							
7	油菜	80	40	叶	1	30	
8	麻风树	60	40	叶	3	15	
9	马樱丹	120	80	叶花	1	40	
10	鹊肾树	140	120	叶	1	60	
11							
12							
13							
14							
15							
16							
17							
18							
19							
20							
21							
22							
23							
24							
25							
26							
27							
28							
29							
30							

草本层植物群落调查表

群落名称：白花鬼针草-假蒟-飞机草-斑茅　　样方面积 1 m × 1 m　　野外编号：83
调查时间：2017.02.17　12：15　　记录者：　袁浪兴　　室内编号：

编号	植物名称	株高(cm)	盖度(%)	物候期	生活力	备注
1	黄鹌菜	40	45	叶花	1	
2	少花龙葵	15	5	叶	2	
3	假蒟	25	80	叶果	1	
4						
5	白花鬼针草	60	90	叶花果	1	
6						
7	飞机草	120	80	叶	1	
8						
9	大叶油草	5	60	花	1	
10	藿香蓟	20	15	叶花	2	
11						
12	斑茅	350	80	叶果	1	
13	海南山姜	80	45	叶	1	
14	蛇葡萄	30		叶	1	
15						
16						
17						
18						
19						
20						
21						
22						
23						
24						
25						
26						
27						
28						
29						
30						

说明：物候期：花、叶、果
生活力：1　良好 2　一般　3　较差

总表

群落名称乔-灌-草优势种	荔枝-角花胡颓子-假蒟			野外编号(统一编号)	88
记录者		日期	2017.02.18 10：30	室内编号	
样地面积	20 m × 20 m	详细地点			
GPS 定位	N：19°53.490′<br>E：110°14.436′	海拔高度	117 m		
群落高度		群落的总盖度	90%		
主要层优势种	乔木层：<br>灌木层：<br>草本层：				
群落外貌特点	人工林				
小地形及样地周围环境描述	乡村道路旁				
分层及各层的特点	乔木层	高度			
	灌木层	高度			
	草本层	高度			
	层间植物	高度			
		高度			
备注（之前的土地利用状况）	鲜重 0.10 kg				

说明：数据尽可能填写全面，没有填无

乔木层植物群落调查表

群落名称：荔枝　　样方面积：20 m × 20 m　　野外编号：88

调查时间：2017.02.18　10：30　　记录者：　　室内编号：

编号	植物名称	高度（m）	胸径（cm）	冠幅（m）	物候期	生活力	备注
1	荔枝	12	60	6×7	叶	1	
2	荔枝	12	80	6×6	叶	1	
3	荔枝	10	40	4×5	叶	1	
4							
5	幌伞枫	8	15	2×2	叶	1	
6	幌伞枫	10	15	2×2	叶	1	
7	幌伞枫	10	20	2×3	叶	1	
8							
9	母生	15	20	2×2	叶	2	
10	母生	15	20	2×2	叶	2	
11							
12	麻楝	8	8	2×2	叶	2	
13							
14	黄皮	7	10	2×2	叶	1	
15	黄皮	5	5	1×1	叶	1	
16	黄皮	4	5	1×2	叶	1	
17							
18							
19							
20							
21							
22							
23							
24							
25							
26							
27							
28							
29							
30							
31							
32							
33							
34							
35							

灌丛层植物群落调查表

群落名称：倒吊笔　　样方面积：5 m × 5 m　　野外编号：87
调查时间：2017.02.21　11：16　　记录者：　　室内编号：

编号	植物名称	高度（cm）	冠径（cm）	物候期	生活力	盖度%	株数/丛树
1	苎麻	120	40	叶	1	20	1
2	毛柿	100	30	叶果	1	10	1
3	破布叶	150	70	叶	2	40	1
4							
5	山石榴	200	70	叶	2	40	1
6	海南茄	100	10	叶	1	20	1
7	越南悬钩子	160	30	叶	2	20	1
8							
9	倒吊笔	200	180	叶	2	50	1
10	酒饼簕	120	40	叶	2	20	1
11	大管	120	40	叶	2	20	1
12	油茶	160	120	叶	1	40	1
13							
14							
15							
16							
17							
18							
19							
20							
21							
22							
23							
24							
25							
26							
27							
28							
29							
30							

草本层植物群落调查表

群落名称：蔓生莠竹-海芋-吐烟花-肾蕨　样方面积 1 m × 1 m　　野外编号：87
调查时间：2017.02.21　11：14　　记录者：　　室内编号：

编号	植物名称	株高(cm)	盖度(%)	物候期	生活力	备注
1	海芋	120	60	叶	1	
2	蔓生莠竹	70	80	叶	1	
3	吐烟花	5	60	叶	1	
4						
5	假蒟	10	10	叶	2	
6	粪箕笃	40	20	叶	2	
7	肾蕨	30	60	叶	1	
8						
9	红花酢浆草	3	10	叶花	2	
10	华南毛蕨	50	20	叶	1	
11						
12	叶下珠	5	3	叶	2	
13	眼树莲	2	10	叶	2	
14						
15	开唇兰	40	3	叶花	1	
16	吐烟花	3	40	叶	2	
17	红雾水葛	20	40	叶	1	
18	火龙果	120	20	叶	1	
19						
20						
21						
22						
23						
24						
25						
26						
27						
28						
29						
30						

说明：物候期：花、叶、果
生活力：1　良好 2　一般　3　较差

总表

群落名称乔-灌-草优势种	乌墨-倒吊笔-蔓生莠竹		野外编号(统一编号)	87
记录者		日期 2017.02.21 11：14	室内编号	
样地面积	20×20 m	详细地点		
GPS 定位	N：19°53.793′<br>E：110°14.019′	海拔高度	136 m	
群落高度		群落的总盖度	89%	
主要层优势种	乔木层：<br>灌木层：<br>草本层：			
群落外貌特点	人工林			
小地形及样地周围环境描述	果园边，杂木多			
分层及各层的特点	乔木层	高度		
	灌木层	高度		
	草本层	高度		
	层间植物	高度		
		高度		
备注（之前的土地利用状况）	土壤鲜重：0.08 kg			

说明：数据尽可能填写全面，没有填无

乔木层植物群落调查表

群落名称：荔枝-黄皮　　样方面积：20 m × 20 m　　野外编号：87
调查时间：2017.02.21　11：14　　记录者：　　室内编号：

编号	植物名称	高度（m）	胸径（cm）	冠幅（m）	物候期	生活力	备注
1	乌墨	18	50	8× 8	叶	1	
2	荔枝	15	35	6× 6	叶	1	
3	荔枝	15	50	6× 6	叶	1	
4	荔枝	12	50	3× 5	叶	1	
5	荔枝	12	30	3× 3	叶	1	
6	菠萝蜜	15	50	7× 7	叶果	1	
7	苦楝	16	50	6× 6	叶	1	
8	椰子	18	30	3× 3	叶	1	
9	黄皮	6	8	1× 2	叶	1	
10	黄皮	6	10	2× 2	叶	1	
11	黄皮	7	15	2× 2	叶	1	
12							
13							
14							
15							
16							
17							
18							
19							
20							
21							
22							
23							
24							
25							
26							
27							
28							
29							
30							
31							
32							
33							
34							
35							

灌丛层植物群落调查表

群落名称：柑橘-咖啡　　样方面积：5 m × 5 m　　野外编号：86
调查时间：2017.02.21　11：54　　记录者：　　室内编号：

编号	植物名称	高度（cm）	冠径（cm）	物候期	生活力	盖度%	株数 / 丛树
1	柑橘	200	20	叶	2	60	1
2	鹰爪花	70	20	叶	2	30	2
3							
4	破布叶	70	40	叶	2	30	1
5	咖啡	300	150	叶果	1	60	1
6	菜豆树	300	60	叶	2	30	1
7							
8	潺槁木姜子	50	30	叶	2	20	1
9	鹊肾树	60	30	叶	1	40	1
10							
11							
12							
13							
14							
15							
16							
17							
18							
19							
20							
21							
22							
23							
24							
25							
26							
27							
28							
29							
30							

草本层植物群落调查表

群落名称：假蒟-海芋-粪箕笃-麒麟尾　样方面积 1 m × 1 m　　野外编号：86
调查时间：2017.02.21　11：54　　记录者：　　室内编号：

编号	植物名称	株高(cm)	盖度(%)	物候期	生活力	备注
1	假蒟	20	80	叶	1	
2	海芋	80	60	叶	1	
3	飞机草	140	20	叶	1	
4						
5	耳叶马兜铃	400	40	叶	2	
6	乌蔹梅	120	50	叶	2	
7						
8	蔓生莠竹	70	40	叶	2	
9	粪箕笃	20	60	叶	1	
10	淡竹叶	30	5	叶	2	
11						
12	鸭跖草	20	20	叶	1	
13	土人参	15	10	叶	2	
14						
15	麒麟尾	300	60	叶	2	
16	露兜树	200	80	叶	2	
17	鬼针草	40	20	叶花	1	
18						
19						
20						
21						
22						
23						
24						
25						
26						
27						
28						
29						
30						

说明：物候期：花、叶、果
生活力：1　良好 2　一般 3　较差

总表

<table>
<tr><td>群落名称
乔-灌-草
优势种</td><td colspan="3">菠萝蜜-咖啡-假蒟</td><td>野外编号
(统一编号)</td><td colspan="2">86</td></tr>
<tr><td>记录者</td><td colspan="2"></td><td>日期</td><td>2017.02.21
11：54</td><td>室内编号</td><td></td></tr>
<tr><td>样地面积</td><td colspan="2">20×20 m</td><td>详细地点</td><td colspan="3"></td></tr>
<tr><td>GPS 定位</td><td>N：19°53.727′
E：110°13.407′</td><td>海拔高度</td><td colspan="4">124 m</td></tr>
<tr><td>群落高度</td><td colspan="3"></td><td>群落的总盖度</td><td colspan="2">98%</td></tr>
<tr><td>主要层优势种</td><td colspan="6">乔木层：
灌木层：
草本层：</td></tr>
<tr><td>群落外貌特点</td><td colspan="6">人工林</td></tr>
<tr><td>小地形及样地周围环境描述</td><td colspan="6">荒废果园，杂草众多</td></tr>
<tr><td rowspan="5">分层及各层的特点</td><td>乔木层</td><td>高度</td><td colspan="4"></td></tr>
<tr><td>灌木层</td><td>高度</td><td colspan="4"></td></tr>
<tr><td>草本层</td><td>高度</td><td colspan="4"></td></tr>
<tr><td>层间植物</td><td>高度</td><td colspan="4"></td></tr>
<tr><td></td><td>高度</td><td colspan="2"></td><td></td><td></td></tr>
<tr><td>备注（之前的土地利用状况）</td><td colspan="6">土壤鲜重：0.08 kg</td></tr>
</table>

说明：数据尽可能填写全面，没有填无

乔木层植物群落调查表

群落名称：菠萝蜜　　样方面积：20 m × 20 m　　野外编号：86
调查时间：2017.02.21　11：56　　记录者：　　室内编号：

编号	植物名称	高度（m）	胸径（cm）	冠幅（m）	物候期	生活力	备注
1	菠萝蜜	12	30	3× 3	叶果	1	
2	菠萝蜜	13	30	3× 3	叶果	1	
3	菠萝蜜	10	25	3× 3	叶	1	
4	菠萝蜜	9	20	2× 3	叶果	1	
5	荔枝	12	70	5× 5	叶	1	
6	黄皮	9	25	4× 4	叶	1	
7	黄皮	8	15	2× 2	叶	1	
8	毛八角枫	8	15	2× 2	叶	1	
9							
10							
11							
12							
13							
14							
15							
16							
17							
18							
19							
20							
21							
22							
23							
24							
25							
26							
27							
28							
29							
30							
31							
32							
33							
34							
35							

灌丛层植物群落调查表

群落名称：马樱丹　　　样方面积：1 m × 1 m　　　野外编号：90

调查时间：2017.02.18　12：12　　　记录者：　　　室内编号：

编号	植物名称	高度（cm）	冠径（cm）	物候期	生活力	盖度%	株数/丛树
1	苎麻	120	80	叶	1	60	
2	马樱丹	180	120	叶花	1	80	
3	马樱丹	120	60	叶	1	40	
4	木薯	120	40	叶	3	10	
5	大管	60	20	叶	2	20	
6	鹅掌藤	180	120	叶	1	80	
7							
8							
9							
10							
11							
12							
13							
14							
15							
16							
17							
18							
19							
20							
21							
22							
23							
24							
25							
26							
27							
28							
29							
30							

草本层植物群落调查表

群落名称：飞机草-白子菜　　　样方面积 1 m × 1 m　　　野外编号：90

调查时间：2017.02.18　12：20　　　记录者：　　　室内编号：

编号	植物名称	株高(cm)	盖度(%)	物候期	生活力	备注
1	白子菜	60	90	叶	1	
2	翼茎白粉藤	60	40	叶	1	
3	野菊	20	50	叶	1	
4	海芋	60	40	叶	1	
5	飞机草	40	20	叶花	1	
6	白粉藤	40	60	叶	1	
7	鸭跖草	20	15	叶	1	
8	飞机草	120	80	叶果	1	
9						
10						
11						
12						
13						
14						
15						
16						
17						
18						
19						
20						
21						
22						
23						
24						
25						
26						
27						
28						
29						
30						

说明：物候期：花、叶、果

生活力：1　良好 2　一般　3　较差

总表

群落名称乔-灌-草优势种	荔枝-马缨丹-柊叶			野外编号(统一编号)	90
记录者		日期	2017.02.18 12：18	室内编号	
样地面积	20×20 m	详细地点			
GPS 定位	N：19°53.499′<br>E：110°15.768′	海拔高度	101 m		
群落高度			群落的总盖度	60%	
主要层优势种	乔木层：<br>灌木层：<br>草本层：				
群落外貌特点	人工林				
小地形及样地周围环境描述	村旁				
分层及各层的特点	乔木层	高度			
	灌木层	高度			
	草本层	高度			
	层间植物	高度			
		高度			
备注（之前的土地利用状况）	鲜重：0.10 kg				

说明：数据尽可能填写全面，没有填无

乔木层植物群落调查表

群落名称：菠萝蜜　　样方面积：20 m × 20 m　　野外编号：90
调查时间：2017.02.18　12：10　　记录者：　　室内编号：

编号	植物名称	高度（m）	胸径（cm）	冠幅（m）	物候期	生活力	备注
1	番木瓜	7	20	1×1	果	1	
2	荔枝	16	80	8×8	叶	1	
3	荔枝	12	30	4×3	叶	1	
4	苦楝	10	20	3×3	叶	1	
5	菠萝蜜	10	20	2×2	果	1	
6	菠萝蜜	7	15	2×2	果	1	
7	菠萝蜜	15	30	3×3	叶	1	
8	菠萝蜜	10	20	3×2	叶	1	
9	鸡蛋果	8	18	3×3	果	1	
10							
11							
12							
13							
14							
15							
16							
17							
18							
19							
20							
21							
22							
23							
24							
25							
26							
27							
28							
29							
30							
31							
32							
33							
34							
35							

灌丛层植物群落调查表

群落名称：白藤　　样方面积：5 m × 5 m　　野外编号：89
调查时间：2017.02.18　11：00　　记录者：　　室内编号：

编号	植物名称	高度（cm）	冠径（cm）	物候期	生活力	盖度%	株数 / 丛树
1	黄牛木	120	80	叶	1	20	
2	酒饼簕	150	60	叶	1	20	
3	猬实	120	80	叶	1	40	
4							
5	细基丸	140	60	叶	1	20	
6	毛柿	150	120	叶	1	40	
7	黑面神	120	40	叶	1	20	
8							
9	白藤	180	120	叶	1	80	
10	水仙柯	160	80	叶	1	40	
11							
12							
13							
14							
15							
16							
17							
18							
19							
20							
21							
22							
23							
24							
25							
26							
27							
28							
29							
30							

草本层植物群落调查表

群落名称：露兜-海南山姜　　样方面积 1 m × 1 m　　野外编号：89
调查时间：2017.02.18　11：10　　记录者：　　室内编号：

编号	植物名称	株高(cm)	盖度(%)	物候期	生活力	备注
1	白花鬼针草	150	60	叶花	1	
2	飞机草	120	40	叶花果	1	
3						
4	海南山姜	250	80	叶	1	
5	吐烟花	15	40	叶	1	
6						
7	山牵牛	180	40	叶花	1	
8	翼茎白粉藤	150	20	叶	2	
9						
10	露兜	150	80	叶	1	
11						
12	肾蕨	20	40	叶	1	
13	翼茎白粉藤	250	20	叶果	1	
14						
15						
16						
17						
18						
19						
20						
21						
22						
23						
24						
25						
26						
27						
28						
29						
30						

说明：物候期：花、叶、果
生活力：1　良好 2　一般　3　较差

总表

群落名称<br>乔-灌-草<br>优势种	荔枝-毛柿-海南山姜		野外编号<br>(统一编号)	89
记录者		日期	2017.02.17<br>11：00	室内编号	
样地面积	20 m × 20 m	详细地点		
GPS 定位	N：19°53.489′<br>E：110°15.100′	海拔高度	102 m	
群落高度		群落的总盖度	90%	
主要层优势种	乔木层：<br>灌木层：<br>草本层：			
群落外貌特点	次生林			
小地形及样地周围环境描述	乡村边，火山石众多			
分层及各层的特点	乔木层	高度		
	灌木层	高度		
	草本层	高度		
	层间植物	高度		
		高度		
备注（之前的土地利用状况）	鲜重 0.10 kg			

说明：数据尽可能填写全面，没有填无

乔木层植物群落调查表

群落名称：荔枝　　样方面积：20 m × 20 m　　野外编号：89
调查时间：2017.02.18　11：00　　记录者：　　室内编号：

编号	植物名称	高度（m）	胸径（cm）	冠幅（m）	物候期	生活力	备注
1	荔枝	7	20	3×4	叶	1	
2	荔枝	8	35	4×4	叶	1	
3	荔枝	8	30	4×4	叶	1	
4							
5	山楝	7	15	1×1	叶	1	
6							
7	油茶	8	20	3×5	叶	1	
8							
9	黄牛木	7	10	2×2	叶	1	
10							
11							
12							
13							
14							
15							
16							
17							
18							
19							
20							
21							
22							
23							
24							
25							
26							
27							
28							
29							
30							
31							
32							
33							
34							
35							

灌丛层植物群落调查表

群落名称：角花胡颓子　　样方面积：5 m × 5 m　　野外编号：88
调查时间：2017.02.18　10：30　　记录者：　　室内编号：

编号	植物名称	高度（cm）	冠径（cm）	物候期	生活力	盖度%	株数/丛树
1	九节	120	80	叶	2	20	
2	毛柿	100	80	叶果	1	40	
3							
4	海南破布叶	120	60	叶	2	20	
5	潺槁木姜子	80	40	叶	1	20	
6							
7	角花胡颓子	150	80	叶	1	60	
8							
9							
10							
11							
12							
13							
14							
15							
16							
17							
18							
19							
20							
21							
22							
23							
24							
25							
26							
27							
28							
29							
30							

草本层植物群落调查表

群落名称：假蒟-吐烟花　　样方面积 1 m × 1 m　　野外编号：88
调查时间：2017.02.18　10：40　记录者：　　室内编号：

编号	植物名称	株高(cm)	盖度(%)	物候期	生活力	备注
1	假蒟	15	80	叶	1	
2						
3	凤尾蕨	25	20	叶	1	
4	假蒟	20	78	叶	1	
5						
6	海芋	60	75	叶	1	
7	假蒟	20	35	叶		
8						
9	吐烟花	5	80	叶	1	
10	鸭趾草	15	20	叶	1	
11						
12	露兜树	120	60	叶	1	
13	蔓生莠竹	30	50	叶花	1	
14						
15						
16						
17						
18						
19						
20						
21						
22						
23						
24						
25						
26						
27						
28						
29						
30						

说明：物候期：花、叶、果
生活力：1　良好 2　一般　3　较差

灌丛层植物群落调查表

群落名称：海南菜豆树　　样方面积：1 m ×1 m　　野外编号：92
调查时间：2017.02.17　14：00　　记录者：　　室内编号：

编号	植物名称	高度（cm）	冠径（cm）	物候期	生活力	盖度%	株数 / 丛树
1	菠萝蜜	150	20	叶	2	2	
2							
3	九节	120	40	叶	1	10	
4	潺槁木姜子	120	10	叶	2	5	
5	九节	100	60	叶	1	30	
6							
7	海南菜豆树	120	80	叶	1	40	
8	海南破布叶	80	20	叶	3	10	
9							
10							
11							
12							
13							
14							
15							
16							
17							
18							
19							
20							
21							
22							
23							
24							
25							
26							
27							
28							
29							
30							

草本层植物群落调查表

群落名称：假蒟-海芋　　样方面积 1 m × 1 m　　野外编号：92
调查时间：2017.02.17　14：10　　记录者：　　室内编号：

编号	植物名称	株高(cm)	盖度(%)	物候期	生活力	备注
1	假蒟	25	60	叶	1	
2	假蒟	30	40	叶	1	
3						
4	柊叶	60	80	叶	1	
5						
6	柊叶	50	60	叶	1	
7	假蒟	35	30	叶	1	
8						
9	海芋	120	75	叶	1	
10	假蒟	40	30	叶	1	
11						
12	落葵	120	10	叶	1	
13	海芋	140	80	叶	1	
14						
15						
16						
17						
18						
19						
20						
21						
22						
23						
24						
25						
26						
27						
28						
29						
30						

说明：物候期：花、叶、果
生活力：1　良好 2　一般　3　较差

总表

群落名称 乔-灌-草 优势种	山楝-九节-柊叶			野外编号 (统一编号)	92
记录者		日期	2017.02.17	室内编号	
样地面积	20 m×20 m	详细地点			
GPS 定位	N：19 °53.240′<br>E：110 °10.988′	海拔高度	m		
群落高度		群落的总盖度			
主要层优势种	乔木层：<br>灌木层：<br>草本层：				
群落外貌特点	次生林				
小地形及样地周围环境描述	湖边				
分层及各层的特点	乔木层	高度			
	灌木层	高度			
	草本层	高度			
	层间植物	高度			
		高度			
备注（之前的土地利用状况）	鲜重：0.12 kg				

说明：数据尽可能填写全面，没有填无

乔木层植物群落调查表

群落名称：波罗蜜　　样方面积：　　野外编号：92

调查时间：2017.02.17 .14：00　　记录者：　　室内编号：

编号	植物名称	高度（m）	胸径（cm）	冠幅（m）	物候期	生活力	备注
1	山楝	15	60		6×7	1	
2							
3	椰子	13	30		4×4	1	
4	椰子	8	50		4×4	1	
5							
6	菠萝蜜	7	15		3×3	1	
7	菠萝蜜	5	8		2×2	1	
8	菠萝蜜	5	10		2×2	1	
9							
10	簕竹	15	8		6×8	1	
11							
12							
13							
14							
15							
16							
17							
18							
19							
20							
21							
22							
23							
24							
25							
26							
27							
28							
29							
30							
31							
32							
33							
34							
35							

灌丛层植物群落调查表

群落名称：山小橘　　样方面积：1 m ×1 m　　野外编号：91
调查时间：2017.02.17　　记录者：　　室内编号：

编号	植物名称	高度（cm）	冠径（cm）	物候期	生活力	盖度%	株数/丛树
1	山小橘	200	150	叶	1	85	
2							
3	倒吊笔	210	140	叶	2	60	
4	对叶榕	150	35	叶	3	15	
5							
6	对叶榕	180	120	叶	2	40	
7	鹊肾树	250	120	叶	1	50	
8							
9							
10							
11							
12							
13							
14							
15							
16							
17							
18							
19							
20							
21							
22							
23							
24							
25							
26							
27							
28							
29							
30							

草本层植物群落调查表

群落名称：假蒟　　样方面积 1 m × 1 m　　野外编号：91
调查时间：2017.02.17　1:35　　记录者：　　室内编号：

编号	植物名称	株高(cm)	盖度(%)	物候期	生活力	备注
1	海芋	60	80	叶花	1	
2	假蒟	40	60	叶	1	
3						
4	假蒟	40	90	叶	1	
5						
6	火炭母	30	15	叶	1	
7	假蒟	40	80	叶	1	
8						
9	假蒟	35	75	叶	1	
10	美人蕉	65	10	叶花	1	
11						
12	假蒟	45	85	叶	1	
13	薜荔	50	10	叶	1	
14						
15						
16						
17						
18						
19						
20						
21						
22						
23						
24						
25						
26						
27						
28						
29						
30						

说明：物候期：花、叶、果
生活力：1　良好 2　一般　3　较差

总表

群落名称乔-灌-草优势种	麻楝-鹊肾树-假蒟			野外编号(统一编号)	91
记录者		日期	2017.02.17	室内编号	
样地面积	20 m×20 m	详细地点			
GPS 定位	N：19°53.220′<br>E：110°10.55′	海拔高度	91 m		
群落高度			群落的总盖度		
主要层优势种	乔木层：<br>灌木层：<br>草本层：				
群落外貌特点	次生林				
小地形及样地周围环境描述	村旁				
分层及各层的特点	乔木层	高度			
	灌木层	高度			
	草本层	高度			
	层间植物	高度			
		高度			
备注（之前的土地利用状况）	鲜重：0.12 kg				

说明：数据尽可能填写全面，没有填无

乔木层植物群落调查表

群落名称：麻楝　　样方面积：　　野外编号：91
调查时间：2017.02.17. 13：30　　记录者：　　室内编号：

编号	植物名称	高度（m）	胸径（cm）	冠幅（m）	物候期	生活力	备注
1	山楝	12	40	6×8	叶	1	
2	山楝	14	50	7×8	叶	1	
3							
4	对叶榕	7	10	3×4	叶	2	
5							
6	苦楝	15	40	4×5	叶	3	
7	苦楝						
8	麻楝	12	30	3×5	叶	2	
9	麻楝	12	40	3×5	叶	2	
10	麻楝	12	30	3×5	叶	2	
11							
12	乌墨	10	25	2×3	叶	2	
13							
14	秋枫	9	20	2×2	叶	2	
15							
16	五月茎	8	25	3×3	叶	1	
17							
18	菠萝密	6	18	2×2	叶	1	
19							
20							
21							
22							
23							
24							
25							
26							
27							
28							
29							
30							
31							
32							
33							
34							
35							

总表

<table>
<tr><td>群落名称
乔-灌-草
优势种</td><td colspan="4">麻楝-鹊肾树-假蒟</td><td>野外编号
(统一编号)</td><td colspan="2">91</td></tr>
<tr><td>记录者</td><td colspan="2"></td><td>日期</td><td>2017.02.17</td><td>室内编号</td><td colspan="2"></td></tr>
<tr><td>样地面积</td><td colspan="2">20 m×20 m</td><td>详细地点</td><td colspan="4"></td></tr>
<tr><td>GPS 定位</td><td>N：19°53.220′
E：110°10.55′</td><td>海拔高度</td><td colspan="5">91 m</td></tr>
<tr><td>群落高度</td><td colspan="3"></td><td>群落的总盖度</td><td colspan="3"></td></tr>
<tr><td>主要层优势种</td><td colspan="7">乔木层：
灌木层：
草本层：</td></tr>
<tr><td>群落外貌特点</td><td colspan="7">次生林</td></tr>
<tr><td>小地形及样地周围环境描述</td><td colspan="7">村旁</td></tr>
<tr><td rowspan="5">分层及各层的特点</td><td>乔木层</td><td>高度</td><td colspan="5"></td></tr>
<tr><td>灌木层</td><td>高度</td><td colspan="5"></td></tr>
<tr><td>草本层</td><td>高度</td><td colspan="5"></td></tr>
<tr><td>层间植物</td><td>高度</td><td colspan="5"></td></tr>
<tr><td></td><td>高度</td><td colspan="3"></td><td></td><td></td></tr>
<tr><td>备注（之前的土地利用状况）</td><td colspan="7">鲜重：0.12 kg</td></tr>
</table>

说明：数据尽可能填写全面，没有填无

乔木层植物群落调查表

群落名称：麻楝　　样方面积：　　野外编号：91
调查时间：2017.02.17. 13：30　　记录者：　　室内编号：

编号	植物名称	高度（m）	胸径（cm）	冠幅（m）	物候期	生活力	备注
1	山楝	12	40	6×8	叶	1	
2	山楝	14	50	7×8	叶	1	
3							
4	对叶榕	7	10	3×4	叶	2	
5							
6	苦楝	15	40	4×5	叶	3	
7	苦楝						
8	麻楝	12	30	3×5	叶	2	
9	麻楝	12	40	3×5	叶	2	
10	麻楝	12	30	3×5	叶	2	
11							
12	乌黑	10	25	2×3	叶	2	
13							
14	秋枫	9	20	2×2	叶	2	
15							
16	五月茎	8	25	3×3	叶	1	
17							
18	菠萝密	6	18	2×2	叶	1	
19							
20							
21							
22							
23							
24							
25							
26							
27							
28							
29							
30							
31							
32							
33							
34							
35							

灌丛层植物群落调查表

群落名称：马樱丹　　样方面积：1 m × 1 m　　野外编号：90
调查时间：2017.02.18　12：12　　记录者：　　室内编号：

编号	植物名称	高度（cm）	冠径（cm）	物候期	生活力	盖度%	株数 / 丛树
1	苎麻	120	80	叶	1	60	
2	马樱丹	180	120	叶花	1	80	
3	马樱丹	120	60	叶	1	40	
4	木薯	120	40	叶	3	10	
5	大管	60	20	叶	2	20	
6	鹅掌藤	180	120	叶	1	80	
7							
8							
9							
10							
11							
12							
13							
14							
15							
16							
17							
18							
19							
20							
21							
22							
23							
24							
25							
26							
27							
28							
29							
30							

草本层植物群落调查表

群落名称：飞机草-白子菜　　样方面积 1 m × 1 m　　野外编号：90
调查时间：　2017.02.18　12：20　　记录者：　　室内编号：

编号	植物名称	株高(cm)	盖度(%)	物候期	生活力	备注
1	白子菜	60	90	叶	1	
2	翼茎白粉藤	60	40	叶	1	
3	野菊	20	50	叶	1	
4	海芋	60	40	叶	1	
5	飞机草	40	20	叶花	1	
6	白粉藤	40	60	叶	1	
7	鸭跖草	20	15	叶	1	
8	飞机草	120	80	叶果	1	
9						
10						
11						
12						
13						
14						
15						
16						
17						
18						
19						
20						
21						
22						
23						
24						
25						
26						
27						
28						
29						
30						

说明：物候期：花、叶、果
生活力：1　良好 2　一般　3　较差

总表

群落名称<br>乔-灌-草<br>优势种	荔枝-破布叶-假蒟			野外编号<br>(统一编号)	95
记录者		日期	2017.02.18<br>8：40	室内编号	
样地面积	20×20 m	详细地点			
GPS 定位	N：19°53.203′<br>E：110°12.943′	海拔高度	106 m		
群落高度			群落的总盖度	75%	
主要层优势种	乔木层：<br>灌木层：<br>草本层：				
群落外貌特点	人工林				
小地形及样地周围环境描述	荒废果园，杂草多				
分层及各层的特点	乔木层	高度			
	灌木层	高度			
	草本层	高度			
	层间植物	高度			
		高度			
备注（之前的土地利用状况）	土壤鲜重：0.12 kg				

说明：数据尽可能填写全面，没有填无

乔木层植物群落调查表

群落名称：荔枝　　样方面积：20 m × 20 m　　野外编号：95
调查时间：2017.02.18　8：40　　记录者：　　室内编号：

编号	植物名称	高度（m）	胸径（cm）	冠幅（m）	物候期	生活力	备注
1	菠萝蜜	9	25	1×2	叶	1	
2	菠萝蜜	9	28	2×3	叶	1	
3	荔枝	8	30	3×3	叶	1	
4	荔枝	9	22	2×3	叶	1	
5	荔枝	9	30	2×3	叶	1	
6	黄皮	8	12	3×4	叶	1	
7	簕竹	12	8	4×4	叶	1	
8							
9							
10							
11							
12							
13							
14							
15							
16							
17							
18							
19							
20							
21							
22							
23							
24							
25							
26							
27							
28							
29							
30							
31							
32							
33							
34							
35							

灌丛层植物群落调查表

群落名称：破布叶-鹊肾树-黄牛木　　样方面积：5 m × 5 m　　野外编号：94
调查时间：2017.02.18　8：15　　记录者：　　室内编号：

编号	植物名称	高度（cm）	冠径（cm）	物候期	生活力	盖度%	株数 / 丛树
1	破布叶	150	120	叶	2	40	1
2	鹊肾树	180	80	叶	1	40	1
3	猪肚木	230	90	叶	2	36	1
4							
5	斜叶榕	120	60	叶	1	30	1
6	酒饼簕	80	60	叶	1	20	1
7	细基丸	80	20	叶	2	10	1
8							
9	赤楠	60	60	叶	1	35	1
10	九节	80	20	叶	1	10	1
11	潺槁木姜子	60	40	叶	1	30	1
12	黄牛木	160	80	叶	2	40	1
13							
14							
15							
16							
17							
18							
19							
20							
21							
22							
23							
24							
25							
26							
27							
28							
29							
30							

草本层植物群落调查表

群落名称：吐烟花　　样方面积 1 m × 1 m　　野外编号：94
调查时间：2017.02.18　8：20　　记录者：　　室内编号：

编号	植物名称	株高(cm)	盖度(%)	物候期	生活力	备注
1	吐烟花	15	80	叶	1	
2	鸭趾草	30	20	叶	1	
3						
4	麦冬	20	30	叶	1	
5	落地生根	45	20	叶	1	
6						
7	鸭趾草	20	40	叶	1	
8						
9	吐烟花	5	70	叶	1	
10						
11	十万错	25	40	叶	1	
12	吐烟花	5	40	叶	1	
13						
14						
15						
16						
17						
18						
19						
20						
21						
22						
23						
24						
25						
26						
27						
28						
29						
30						

说明：物候期：花、叶、果
生活力：1　良好 2　一般 3　较差

灌丛层植物群落调查表

群落名称：破布叶-鹊肾树-黄牛木　　样方面积：5 m × 5 m　　野外编号：94
调查时间：　2017.02.18　8：15　　记录者：　　室内编号：

编号	植物名称	高度（cm）	冠径（cm）	物候期	生活力	盖度%	株数/丛树
1	破布叶	150	120	叶	2	40	1
2	鹊肾树	180	80	叶	1	40	1
3	猪肚木	230	90	叶	2	36	1
4							
5	斜叶榕	120	60	叶	1	30	1
6	酒饼簕	80	60	叶	1	20	1
7	细基丸	80	20	叶	2	10	1
8							
9	赤楠	60	60	叶	1	35	1
10	九节	80	20	叶	1	10	1
11	瀑槁木姜子	60	40	叶	1	30	1
12	黄牛木	160	80	叶	2	40	1
13							
14							
15							
16							
17							
18							
19							
20							
21							
22							
23							
24							
25							
26							
27							
28							
29							
30							

草本层植物群落调查表

群落名称：吐烟花　　样方面积 1 m × 1 m　　野外编号：94
调查时间：　2017.02.18　8：20　　记录者：　　室内编号：

编号	植物名称	株高(cm)	盖度(%)	物候期	生活力	备注
1	吐烟花	15	80	叶	1	
2	鸭趾草	30	20	叶	1	
3						
4	麦冬	20	30	叶	1	
5	落地生根	45	20	叶	1	
6						
7	鸭趾草	20	40	叶	1	
8						
9	吐烟花	5	70	叶	1	
10						
11	十万错	25	40	叶	1	
12	吐烟花	5	40	叶	1	
13						
14						
15						
16						
17						
18						
19						
20						
21						
22						
23						
24						
25						
26						
27						
28						
29						
30						

说明：物候期：花、叶、果
生活力：1　良好 2 一般 3 较差

灌丛层植物群落调查表

群落名称：马樱丹-牛筋果　　样方面积：1 m × 1 m　　野外编号：93

调查时间：2017.02.17　14：00　　记录者：　　室内编号：

编号	植物名称	高度（cm）	冠径（cm）	物候期	生活力	盖度%	株数 / 丛树
1	鹊肾树	60	40	叶	3	10	
2	山麻黄	75	40	叶	1	20	
3							
4	潺槁木姜子	4	20	叶	1	15	
5	两面针	45	25	叶	1	20	
6	鹊肾树	85	40	叶	2	40	
7							
8	梵天花	80	30	叶	2	40	
9	马缨丹	120	120	叶	1	80	
10							
11	牛筋果	350	180	叶	1	75	
12	粗糠柴	150	60	叶	1	60	
13							
14							
15							
16							
17							
18							
19							
20							
21							
22							
23							
24							
25							
26							
27							
28							
29							
30							

草本层植物群落调查表

群落名称：假蒟-火炭母　　样方面积 1 m × 1 m　　野外编号：93

调查时间：2017.02.17.　14：35　　记录者：　　室内编号：

编号	植物名称	株高(cm)	盖度(%)	物候期	生活力	备注
1						
2	假蒟	15	80	叶花	1	
3	鬼针草	20	40	叶花	2	
4						
5	藿香蓟	15	10	叶花	2	
6	丰花草	15	15	叶花	1	
7	含羞草	20	15	叶花	1	
8						
9	黄鹌菜	25	15	叶花	1	
10	酢浆草	5	10	叶花	1	
11	火炭母	15	60	叶	1	
12						
13	夜香牛	15	10	叶花	1	
14	鸭趾草	10	30	叶	2	
15	粪箕笃	15	20	叶	1	
16						
17	落葵	15	40	叶花	1	
18	黄花稔	20	15	叶花	1	
19	蛇葡萄	15	25	叶	1	
20						
21						
22						
23						
24						
25						
26						
27						
28						
29						
30						

说明：物候期：花、叶、果

生活力：1　良好 2　一般　3　较差

总表

群落名称乔-灌-草优势种	龙眼-猪肚木-吐烟花		野外编号(统一编号)	94
记录者		日期 2017.02.18 8：15	室内编号	
样地面积	20×20 m	详细地点		
GPS 定位	N：19°53.020′<br>E：110°12.150′	海拔高度	94 m	
群落高度		群落的总盖度	80%	
主要层优势种	乔木层：<br>灌木层：<br>草本层：			
群落外貌特点	人工林			
小地形及样地周围环境描述	火山石多			
分层及各层的特点	乔木层	高度		
	灌木层	高度		
	草本层	高度		
	层间植物	高度		
		高度		
备注（之前的土地利用状况）	土壤鲜重：0.12 kg			

说明：数据尽可能填写全面，没有填无

乔木层植物群落调查表

群落名称：龙眼　样方面积：20 m × 20 m　野外编号：94
调查时间：2017.02.18　8：15　记录者：　室内编号：

编号	植物名称	高度（m）	胸径（cm）	冠幅（m）	物候期	生活力	备注
1	龙眼	7	20	4×4	叶	1	
2	龙眼	5	15	3×3	叶	1	
3	龙眼	6	30	4×6	叶	1	
4	龙眼	6	20	5×5	叶	1	
5	毛八角枫	5	10	1×1	叶	2	
6							
7							
8							
9							
10							
11							
12							
13							
14							
15							
16							
17							
18							
19							
20							
21							
22							
23							
24							
25							
26							
27							
28							
29							
30							
31							
32							
33							
34							
35							

灌丛层植物群落调查表

群落名称：马樱丹-牛筋果　　样方面积：1 m × 1 m　　野外编号：93

调查时间：2017.02.17　14：00　　记录者：　　室内编号：

编号	植物名称	高度（cm）	冠径（cm）	物候期	生活力	盖度%	株数/丛树
1	鹊肾树	60	40	叶	3	10	
2	山麻黄	75	40	叶	1	20	
3							
4	潺槁木姜子	4	20	叶	1	15	
5	两面针	45	25	叶	1	20	
6	鹊肾树	85	40	叶	2	40	
7							
8	梵天花	80	30	叶	2	40	
9	马缨丹	120	120	叶	1	80	
10							
11	牛筋果	350	180	叶	1	75	
12	粗糠柴	150	60	叶	1	60	
13							
14							
15							
16							
17							
18							
19							
20							
21							
22							
23							
24							
25							
26							
27							
28							
29							
30							

草本层植物群落调查表

群落名称：假蒟-火炭母　　样方面积 1 m × 1 m　　野外编号：93

调查时间：2017.02.17.　14：35　　记录者：　　室内编号：

编号	植物名称	株高(cm)	盖度(%)	物候期	生活力	备注
1						
2	假蒟	15	80	叶花	1	
3	鬼针草	20	40	叶花	2	
4						
5	藿香蓟	15	10	叶花	2	
6	丰花草	15	15	叶花	1	
7	含羞草	20	15	叶花	1	
8						
9	黄鹌菜	25	15	叶花	1	
10	酢浆草	5	10	叶花	1	
11	火炭母	15	60	叶	1	
12						
13	夜香牛	15	10	叶花	1	
14	鸭趾草	10	30	叶	2	
15	粪箕笃	15	20	叶	1	
16						
17	落葵	15	40	叶花	1	
18	黄花稔	20	15	叶花	1	
19	蛇葡萄	15	25	叶	1	
20						
21						
22						
23						
24						
25						
26						
27						
28						
29						
30						

说明：物候期：花、叶、果

生活力：1　良好 2　一般　3　较差

总表

群落名称乔-灌-草优势种	苦楝-牛筋果-假蒟			野外编号(统一编号)	93
记录者		日期	217.02.17	室内编号	
样地面积	20 m×20 m	详细地点			
GPS 定位	N：19 °53.096′<br>E：110°11.674′	海拔高度	93 m		
群落高度		群落的总盖度			
主要层优势种	乔木层：<br>灌木层：<br>草本层：				
群落外貌特点	次生林				
小地形及样地周围环境描述	湖边				
分层及各层的特点	乔木层	高度			
	灌木层	高度			
	草本层	高度			
	层间植物	高度			
		高度			
备注（之前的土地利用状况）	鲜重：0.14 kg				

说明：数据尽可能填写全面，没有填无

乔木层植物群落调查表

群落名称：菠萝蜜-苦楝　样方面积：　野外编号：93
调查时间：2017.02.17　记录者：　室内编号：

编号	植物名称	高度（m）	胸径（cm）	冠幅（m）	物候期	生活力	备注
1	菠萝蜜	10	60	6×9	叶	1	
2	菠萝蜜	9	15	3×3	叶	1	
3	菠萝蜜	7	15	2×3	叶	1	
4	菠萝蜜	8	15	2×2	叶	1	
5							
6	潘石榴	9	20	3×5	叶	2	
7							
8	发财树	5	10	2×3	叶	1	
9	发财树	5	8	3×3	叶	2	
10							
11	苦楝	14	40	5×4	叶	2	
12	苦楝	12	40	3×5	叶	2	
13	苦楝	12	40	4×6	叶	2	
14	苦楝	15	30	3×2	叶	2	
15							
16							
17							
18							
19							
20							
21							
22							
23							
24							
25							
26							
27							
28							
29							
30							
31							
32							
33							
34							
35							

灌丛层植物群落调查表

群落名称：破布叶-毛柿　　样方面积：5 m × 5 m　　野外编号：95
调查时间：2017.02.18　8：40　　记录者：　　室内编号：

编号	植物名称	高度（cm）	冠径（cm）	物候期	生活力	盖度%	株数 / 丛树
1	破布叶	120	80	叶果	2	20	
2	破布叶	250	120	叶	2	40	
3	九节	120	60	叶	1	20	
4							
5	黑面神	120	40	叶	1	20	
6	毛柿	150	120	叶	1	60	
7	破布叶	160	80	叶	2	40	
8							
9	九节	120	80	叶	1	40	
10	山小橘	120	20	叶	1	20	
11							
12							
13							
14							
15							
16							
17							
18							
19							
20							
21							
22							
23							
24							
25							
26							
27							
28							
29							
30							

草本层植物群落调查表

群落名称：假蒟-蔓生莠竹　　样方面积 1 m × 1 m　　野外编号：95
调查时间：2017.02.18　8：50　　记录者：　　室内编号：

编号	植物名称	株高(cm)	盖度(%)	物候期	生活力	备注
1	假蒟	35	90	叶	1	
2						
3	蔓生莠竹	60	85	叶花	1	
4						
5	络石	5	60	叶	1	
6	丰花草	15	10	叶花	1	
7	野葛	350	60	叶	1	
8						
9	掌叶鱼黄草	80	40	叶	1	
10	厚叶崖爬藤	20	40	叶	1	
11						
12	一点红	20	40	叶花	1	
13	酢浆草	5	45	叶花	1	
14						
15						
16						
17						
18						
19						
20						
21						
22						
23						
24						
25						
26						
27						
28						
29						
30						

说明：物候期：花、叶、果
生活力：1　良好 2　一般　3　较差

总表

群落名称<br>乔-灌-草<br>优势种	龙眼-毛柿-蔓生莠竹			野外编号<br>(统一编号)	96
记录者		日期	2017.02.18<br>9：00	室内编号	
样地面积	20×20 m	详细地点			
GPS 定位	N：19°52.954′<br>E：110°13.312′	海拔高度	111 m		
群落高度			群落的总盖度	98%	
主要层优势种	乔木层：<br>灌木层：<br>草本层：				
群落外貌特点	人工林				
小地形及样地周围环境描述	荒废果园，火山石较多				
分层及各层的特点	乔木层	高度			
	灌木层	高度			
	草本层	高度			
	层间植物	高度			
		高度			
备注（之前的土地利用状况）	土壤鲜重：0.10 kg				

说明：数据尽可能填写全面，没有填无

乔木层植物群落调查表

群落名称：龙眼　　样方面积：20 m × 20 m　　野外编号：96

调查时间：2017.02.18　9：00　　记录者：　　室内编号：

编号	植物名称	高度（m）	胸径（cm）	冠幅（m）	物候期	生活力	备注
1	龙眼	9	40	4×5	叶	1	
2	龙眼	9	40	3× 4	叶	1	
3	龙眼	8	25	3× 4	叶	1	
4	龙眼	8	40	3× 6	叶	1	
5	荔枝	9	50	7× 9	叶	1	
6	乌墨	10	30	3× 5	叶	1	
7							
8							
9							
10							
11							
12							
13							
14							
15							
16							
17							
18							
19							
20							
21							
22							
23							
24							
25							
26							
27							
28							
29							
30							
31							
32							
33							
34							
35							

灌丛层植物群落调查表

群落名称：山石榴-牛筋果　　样方面积：5 m × 5 m　　野外编号：96
调查时间：2017.02.18　9：05　　记录者：　　室内编号：

编号	植物名称	高度（cm）	冠径（cm）	物候期	生活力	盖度%	株数 / 丛树
1	破布叶	80	80	叶	2	40	
2	毛柿	120	80	叶果	1	45	
3							
4	大花紫玉盘	120	80	叶	1	35	
5	倒吊笔	80	60	叶	2	25	
6	酒饼簕	80	20	叶	1	10	
7							
8	牛筋果	40	120	叶	1	60	
9	白藤	120	80	叶	1	35	
10	山石榴	250	120	叶	1	70	
11							
12							
13							
14							
15							
16							
17							
18							
19							
20							
21							
22							
23							
24							
25							
26							
27							
28							
29							
30							

草本层植物群落调查表

群落名称：蔓生莠竹-吐烟花-飞机草　　样方面积 1 m × 1 m　　野外编号：96
调查时间：2017.02.18　9：10　　记录者：　　室内编号：

编号	植物名称	株高(cm)	盖度(%)	物候期	生活力	备注
1	吐烟花	10	80	叶	1	
2	掌叶鱼黄草	40	20	叶花	1	
3						
4	假蒟	15	40	叶	1	
5	翼茎白粉藤	15	30	叶	1	
6						
7	飞机草	230	80	叶花	1	
8						
9	蔓生莠竹	230	90	叶花	1	
10	厚叶崖爬藤	120	20	叶	1	
11						
12	野葛	230	60	叶	1	
13						
14						
15						
16						
17						
18						
19						
20						
21						
22						
23						
24						
25						
26						
27						
28						
29						
30						

说明：物候期：花、叶、果
生活力：1　良好 2　一般　3　较差

总表

群落名称<br>乔-灌-草<br>优势种	荔枝-马缨丹-吐烟花			野外编号<br>(统一编号)	97
记录者		日期	2017.02.18<br>9：30	室内编号	
样地面积	20 m × 20 m	详细地点			
GPS 定位	N：19°52.867′<br>E：110°13.746′	海拔高度	107m		
群落高度			群落的总盖度	90%	
主要层优势种	乔木层：<br>灌木层：<br>草本层：				
群落外貌特点	人工林				
小地形及样地周围环境描述	火山石众多				
分层及各层的特点	乔木层	高度			
	灌木层	高度			
	草本层	高度			
	层间植物	高度			
		高度			
备注（之前的土地利用状况）	鲜重 0.10 kg				

说明：数据尽可能填写全面，没有填无

乔木层植物群落调查表

群落名称：荔枝-水仙柯　　样方面积：20 m × 20 m　　野外编号：97

调查时间：2017.02.18　9：30　　记录者：　　室内编号：

编号	植物名称	高度（m）	胸径（cm）	冠幅（m）	物候期	生活力	备注
1	乌墨	10	30	3×5	叶	2	
2							
3	水仙柯	8	12	2×1	叶	1	
4	水仙柯	8	10	2×1	叶	1	
5	水仙柯	6	10	1×1	叶	1	
6	水仙柯	7	10	1×1	果	1	
7	荔枝	10	70	6×7	叶	1	
8	荔枝	10	45	3×3	叶	1	
9	荔枝	9	40	6×4	叶	1	
10	荔枝	12	75	6×5	花	1	
11							
12							
13							
14							
15							
16							
17							
18							
19							
20							
21							
22							
23							
24							
25							
26							
27							
28							
29							
30							
31							
32							
33							
34							
35							

灌丛层植物群落调查表

群落名称：马樱丹-油茶-假九节　　样方面积：5 m × 5 m　　野外编号：97

调查时间：2017.02.18　9：30　　记录者：　　室内编号：

编号	植物名称	高度（cm）	冠径（cm）	物候期	生活力	盖度%	株数 / 丛树
1	马缨丹	180	120	叶花果	1	80	
2							
3	斑鸠菊	80	40	叶	1	20	
4							
5	假九节	250	160	叶	1	85	
6							
7	毛柿	60	40	叶	2	20	
8	油茶	120	80	叶	2	60	
9							
10							
11							
12							
13							
14							
15							
16							
17							
18							
19							
20							
21							
22							
23							
24							
25							
26							
27							
28							
29							
30							

草本层植物群落调查表

群落名称：吐烟花　　样方面积 1 m × 1 m　　野外编号：97

调查时间：　2017.02.18　9：40　　记录者：　　室内编号：

编号	植物名称	株高(cm)	盖度(%)	物候期	生活力	备注
1	野葛	250	40	叶	1	
2						
3	海芋	160	60	叶花	1	
4						
5	三叶崖爬藤	240	40	叶	1	
6						
7	吐烟花	15	90	叶	1	
8						
9	翼茎白粉藤	80	60	叶	1	
10						
11	眼树莲	60	20	叶	1	
12						
13						
14						
15						
16						
17						
18						
19						
20						
21						
22						
23						
24						
25						
26						
27						
28						
29						
30						

说明：物候期：花、叶、果

生活力：1　良好 2　一般 3　较差

总表

<table>
<tr><td>群落名称乔-灌-草优势种</td><td colspan="3">菠萝蜜-簕欓花椒-斑茅</td><td>野外编号(统一编号)</td><td>98</td></tr>
<tr><td>记录者</td><td colspan="2"></td><td>日期</td><td>2017.02.18 10：00</td><td>室内编号</td></tr>
<tr><td>样地面积</td><td colspan="2">20 m × 20 m</td><td>详细地点</td><td colspan="2"></td></tr>
<tr><td>GPS 定位</td><td>N：19°53.081′
E：110°14.314′</td><td>海拔高度</td><td colspan="3">111 m</td></tr>
<tr><td>群落高度</td><td colspan="3"></td><td>群落的总盖度</td><td>95%</td></tr>
<tr><td>主要层优势种</td><td colspan="5">乔木层：
灌木层：
草本层：</td></tr>
<tr><td>群落外貌特点</td><td colspan="5">次生林</td></tr>
<tr><td>小地形及样地周围环境描述</td><td colspan="5">乡村道路旁，杂草丛生</td></tr>
<tr><td rowspan="5">分层及各层的特点</td><td>乔木层</td><td>高度</td><td colspan="3"></td></tr>
<tr><td>灌木层</td><td>高度</td><td colspan="3"></td></tr>
<tr><td>草本层</td><td>高度</td><td colspan="3"></td></tr>
<tr><td>层间植物</td><td>高度</td><td colspan="3"></td></tr>
<tr><td></td><td>高度</td><td></td><td></td><td></td></tr>
<tr><td>备注（之前的土地利用状况）</td><td colspan="5">鲜重 0.10 kg</td></tr>
</table>

说明：数据尽可能填写全面，没有填无

乔木层植物群落调查表

群落名称：毛八角枫-菠萝蜜　　样方面积：20 m × 20 m　　野外编号：98
调查时间：2017.02.18　10：00　　记录者：　　室内编号：

编号	植物名称	高度（m）	胸径（cm）	冠幅（m）	物候期	生活力	备注
1	菠萝蜜	8	18	1×1	叶	2	
2	菠萝蜜	10	35	3×5	叶	1	
3	菠萝蜜	9	30	3×4	果	1	
4							
5	毛八角枫	9	30	2×2	叶	2	
6	毛八角枫	9	25	2×1	叶	2	
7	毛八角枫	8	20	2×2	叶	2	
8	毛八角枫	6	20	1×1	休眠	3	
9							
10	麻楝	9	20	3×3	叶	1	
11							
12	秋枫	9	25	2×2	叶	2	
13							
14							
15							
16							
17							
18							
19							
20							
21							
22							
23							
24							
25							
26							
27							
28							
29							
30							
31							
32							
33							
34							
35							

灌丛层植物群落调查表

群落名称：簕欓花椒　　　　样方面积：5 m × 5 m　　　野外编号：98
调查时间：2017.02.18　10：00　　　记录者：　　　室内编号：

编号	植物名称	高度（cm）	冠径（cm）	物候期	生活力	盖度%	株数 / 丛树
1	土蜜树	150	60	叶	1	40	
2	马缨丹	120	80	叶花	1	35	
3							
4	假杜鹃	60	40	叶花	2	20	
5	两面针	160	80	叶	1	40	
6							
7	簕欓花椒	310	250	叶	1	80	
8							
9	越南悬钩子	180	40	叶	1	40	
10							
11							
12							
13							
14							
15							
16							
17							
18							
19							
20							
21							
22							
23							
24							
25							
26							
27							
28							
29							
30							

草本层植物群落调查表

群落名称：斑茅-吐烟花-节毛乌蔹莓　样方面积 1 m × 1 m　　野外编号：98
调查时间：2017.02.18　10：05　　记录者：　　　室内编号：

编号	植物名称	株高(cm)	盖度(%)	物候期	生活力	备注
1	吐烟花	15	80	叶	1	
2						
3	海芋	60	25	叶	1	
4						
5	掌叶山猪菜	120	40	叶	1	
6	野葛	240	45	叶	1	
7						
8	节毛乌蔹莓	230	80	叶	1	
9						
10	飞机草	120	40	叶花	1	
11	斑茅	280	85	叶果	1	
12						
13						
14						
15						
16						
17						
18						
19						
20						
21						
22						
23						
24						
25						
26						
27						
28						
29						
30						

说明：物候期：花、叶、果
生活力：1　良好 2　一般　3　较差

总表

<table>
<tr><td>群落名称
乔-灌-草
优势种</td><td colspan="4">荔枝-破布叶-柊叶</td><td>野外编号
(统一编号)</td><td>99</td></tr>
<tr><td>记录者</td><td colspan="2"></td><td>日期</td><td>2017.02.18
11：30</td><td>室内编号</td><td></td></tr>
<tr><td>样地面积</td><td colspan="2">20 m × 20 m</td><td>详细地点</td><td colspan="3"></td></tr>
<tr><td>GPS 定位</td><td>N：19°53.241′
E：110°15.212′</td><td>海拔高度</td><td colspan="4">111 m</td></tr>
<tr><td>群落高度</td><td colspan="3"></td><td>群落的总盖度</td><td colspan="2">100%</td></tr>
<tr><td>主要层优势种</td><td colspan="6">乔木层：
灌木层：
草本层：</td></tr>
<tr><td>群落外貌特点</td><td colspan="6">人工林</td></tr>
<tr><td>小地形及样地周围环境描述</td><td colspan="6">火山石众多，乡道旁</td></tr>
<tr><td rowspan="5">分层及各层的特点</td><td>乔木层</td><td>高度</td><td colspan="4"></td></tr>
<tr><td>灌木层</td><td>高度</td><td colspan="4"></td></tr>
<tr><td>草本层</td><td>高度</td><td colspan="4"></td></tr>
<tr><td>层间植物</td><td>高度</td><td colspan="4"></td></tr>
<tr><td></td><td>高度</td><td colspan="2"></td><td></td><td></td></tr>
<tr><td>备注（之前的土地利用状况）</td><td colspan="6">鲜重 0.12 kg</td></tr>
</table>

说明：数据尽可能填写全面，没有填无

乔木层植物群落调查表

群落名称：荔枝-菠萝蜜　　样方面积：20 m × 20 m　　野外编号：99
调查时间：2017.02.18　11：30　　记录者：　　室内编号：

编号	植物名称	高度（m）	胸径（cm）	冠幅（m）	物候期	生活力	备注
1	菠萝蜜	12	30	2×2	果	1	
2	菠萝蜜	8	20	1×1	叶	2	
3	菠萝蜜	10	40	3×3	果	1	
4							
5	荔枝	12	50	6×6	叶	1	
6	荔枝	12	30	3×3	叶	1	
7	荔枝	13	40	4×4	叶	1	
8	荔枝	15	40	4×5	叶	1	
9							
10	母生	16	20	2×2	叶	1	
11							
12	椰子	15	30	2×2	果	1	
13							
14	黄皮	8	15	2×3	花	1	
15							
16	山楝	15	40	4×3	叶	1	
17							
18							
19							
20							
21							
22							
23							
24							
25							
26							
27							
28							
29							
30							
31							
32							
33							
34							
35							

灌丛层植物群落调查表

群落名称：海南破布叶　　样方面积：5 m × 5 m　　野外编号：99
调查时间：2017.02.18　11：30　　记录者：　　室内编号：

编号	植物名称	高度（cm）	冠径（cm）	物候期	生活力	盖度%	株数 / 丛树
1	海南破布叶	120	100	叶	2	60	
2							
3	海南破布叶	150	120	叶	2	65	
4	海南破布叶	120	80	叶	1	40	
5							
6	毛柿	120	80	叶	2	30	
7	幌伞枫	150	120	叶	1	60	
8	山石榴	80	60	叶	2	20	
9							
10							
11							
12							
13							
14							
15							
16							
17							
18							
19							
20							
21							
22							
23							
24							
25							
26							
27							
28							
29							
30							

草本层植物群落调查表

群落名称：吊竹梅-柊叶　　样方面积 1 m × 1 m　　野外编号：99
调查时间：2017.02.18　11：35　　记录者：　　室内编号：

编号	植物名称	株高(cm)	盖度(%)	物候期	生活力	备注
1	吊竹梅	15	95	叶花	1	
2						
3	吊竹梅	10	60	叶	1	
4	海芋	60	40	叶	1	
5						
6	鳄嘴花	80	20	叶	1	
7	翼茎白粉藤	50	20	叶	3	
8						
9	飞机草	80	40	叶	1	
10	金腰箭	60	20	叶	2	
11	狗肝菜	40	40	叶花	2	
12						
13	一年蓬	120	60	叶花	1	
14						
15	柊叶	80	95	叶	1	
16						
17						
18						
19						
20						
21						
22						
23						
24						
25						
26						
27						
28						
29						
30						

说明：物候期：花、叶、果
生活力：1　良好 2　一般　3　较差

总表

群落名称<br>乔-灌-草<br>优势种	荔枝-山石榴-假蒟				野外编号<br>(统一编号)	100
记录者			日期	2017.02.18<br>11：50	室内编号	
样地面积	20×20 m		详细地点			
GPS 定位	N：19°53.077′<br>E：110°15.608′	海拔高度	108 m			
群落高度				群落的总盖度	90%	
主要层优势种	乔木层：<br>灌木层：<br>草本层：					
群落外貌特点	人工林					
小地形及样地周围环境描述	乡村旁					
分层及各层的特点	乔木层	高度				
	灌木层	高度				
	草本层	高度				
	层间植物	高度				
		高度				
备注（之前的土地利用状况）	鲜重：0.14 kg					

说明：数据尽可能填写全面，没有填无

乔木层植物群落调查表

群落名称：荔枝　　样方面积：　　野外编号：100

调查时间：2017.02.18　11：50　　记录者：　　室内编号：

编号	植物名称	高度（m）	胸径（cm）	冠幅（m）	物候期	生活力	备注
1	荔枝	12	40	4×4	叶	1	
2	荔枝	10	30	3×3	叶	1	
3	荔枝	12	30	3×3	叶	1	
4	荔枝	12	45	3×3	叶	1	
5	龙眼	8	20	2×2	叶	1	
6	石榴	6	5	2×2	叶	1	
7	山楝	6	15	2×2	叶	1	
8	苦楝	15	30	4×3	叶	2	
9	苦楝	15	20	2×2	叶	2	
10	菠萝蜜	6	10	2×2	叶	1	
11	菠萝蜜	8	15	2×2	叶	1	
12							
13							
14							
15							
16							
17							
18							
19							
20							
21							
22							
23							
24							
25							
26							
27							
28							
29							
30							
31							
32							
33							
34							
35							

灌丛层植物群落调查表

群落名称：雀梅藤-海南破布叶-山石榴　　样方面积：5 m × 5 m　　野外编号：100

调查时间：2017.02.18　11：48　　记录者：　　室内编号：

编号	植物名称	高度（cm）	冠径（cm）	物候期	生活力	盖度%	株数 / 丛树
1	赪桐	120	20	叶	3	10	1
2	潺槁木姜子	60	60	叶	2	20	1
3	马樱丹	80	120	叶花	1	60	1
4	雀梅	180	190	叶	1	90	1
5	海南破布叶	200	120	叶	2	90	1
6	山石榴	250	180	叶	2	80	1
7	酒饼簕	120	40	叶	2	30	1
8	两面针	180	120	叶	1	40	1
9							
10							
11							
12							
13							
14							
15							
16							
17							
18							
19							
20							
21							
22							
23							
24							
25							
26							
27							
28							
29							
30							

草本层植物群落调查表

群落名称：假蒟-斑茅　　样方面积 1 m × 1 m　　野外编号：100

调查时间：　2017.02.18　11：55　记录者：　　室内编号：

编号	植物名称	株高(cm)	盖度(%)	物候期	生活力	备注
1	假蒟	15	90	叶	1	
2	淡竹叶	20	10	叶	1	
3	鸭跖草	15	20	叶	1	
4	蔓生莠竹	120	85	叶	1	
5	飞机草	120	80	叶花果	1	
6	海芋	20	60	叶果	1	
7	斑茅	350	90	叶果	1	
8						
9						
10						
11						
12						
13						
14						
15						
16						
17						
18						
19						
20						
21						
22						
23						
24						
25						
26						
27						
28						
29						
30						

说明：物候期：花、叶、果

生活力：1　良好 2　一般　3　较差
